# Dynamic Covalent Radical Recombination for the Assembly of Tuneable Responsive Porous Organic Cages

**DOI:** 10.1002/anie.7638565

**Published:** 2026-05-09

**Authors:** Yannic Hartmann, Robert Oestreich, Yuki Wada, Philippe de Bary, Masaki Kawano, Christoph Janiak, Bernd M. Schmidt

**Affiliations:** ^1^ Institut für Organische Chemie und Makromolekulare Chemie Heinrich‐Heine‐Universität Düsseldorf Düsseldorf Germany; ^2^ Institut für Anorganische Chemie und Strukturchemie Heinrich‐Heine‐Universität Düsseldorf Universitätsstraße 1 Düsseldorf Germany; ^3^ Department of Chemistry Institute of Science Tokyo Meguro‐ku Tokyo Japan; ^4^ Lehrstuhl für Bioanorganische Chemie Heinrich‐Heine‐Universität Düsseldorf Universitätsstraße 1 Düsseldorf Germany

**Keywords:** dynamic covalent chemistry, gas sorption, porous organic cages, radicals, stimuli‐responsive

## Abstract

The construction of discrete organic cages via radical recombination offers a powerful yet underexplored route toward *stimuli*‐responsive, C─C‐linked molecular architectures. Here, we introduce aryldicyanomethyl radical dynamic covalent chemistry as a general strategy for the controlled assembly of porous organic cages. Systematic variation of a single substituent governs both radical and σ‐bond stability as well as the resulting cage geometry, enabling precise, substituent‐dependent control over cage topology and responsiveness. A thiophenoxy‐substituted monomer **S** selectively affords a discrete Tri^2^ dimer in 99% yield, whereas the *N*‐methylaniline‐substituted analogue **N** forms the tetrahedral Tri^4^ tetramer in 83% yield. **N^4^** possesses permanent porosity and pronounced selectivity for CO_2_ and H_2_ over CH_4_ and N_2_, as confirmed by gas sorption experiments, arising from narrow pore apertures and strong host–guest interactions. Both cages display reversible mechano‐ and thermochromic behaviour. Moreover, the combination of a highly dynamic bond formation process with three‐dimensional preorganisation of the cage enables efficient self‐healing, which is markedly accelerated upon exposure to THF vapour. Collectively, these results establish radical recombination as an unexplored dynamic covalent motif for the synthesis of responsive organic cage architectures, enabling substituent‐dependent fine‐tuning of topology, stability, and material function through simple substituent modification.

## Graphical abstract

Dynamic covalent chemistry (DCC) based on reversible radical recombination enables the assembly of responsive, C─C‐linked organic cages. Substituent‐controlled aryldicyanomethyl radicals form discrete, porous architectures with tuneable topology of Tri^2^ and Tri^4^ cages, gas selectivity, chromism, and self‐healing behaviour, establishing radical‐based DCC as a versatile platform for adaptive porous materials.

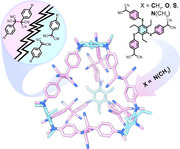

Dynamic covalent chemistry (DCC) [1] serves as a powerful platform for the construction of discrete molecular architectures as well as extended frameworks [2, 3]. In particular, reversible imine and boronate ester condensation reactions have enabled the synthesis of porous materials such as covalent organic frameworks (COFs) and porous organic cages (POCs) [4, 5, 6, 7, 8, 9], in which reversible bond formation facilitates error correction during self‐assembly, allowing kinetically formed defects to equilibrate and converge toward the thermodynamically favoured architectures. While these heteroatom‐based dynamic reactions have been explored extensively, C–C linkages remain largely untouched, even though the C–C single bond is one of the most ubiquitous covalent connections in organic and biological matter and is fundamental to molecular structure and function [10, 11]. But the high bond dissociation energy of C–C single bonds has made their incorporation into dynamic regimes particularly challenging. A promising strategy to overcome this limitation is the use of persistent carbon‐centred radicals that exist in equilibrium with their recombined σ‐bonded dimers, without relying on classical heterolytic condensation chemistry. Such homolytic processes feature low energetic barriers, proceed without catalysts, and generate no byproducts, thus enabling rapid, well‐behaved equilibria under mild conditions [12, 13, 14]. Beyond their fundamental appeal, dynamic radical‐based linkages provide access to intrinsically responsive motifs that translate directly into macroscopic function. By integrating responsiveness and dynamic covalent behaviour within a single bond, this approach eliminates the need for responsive subunits and renders such systems attractive for sensory applications [15], magnetic frameworks [16] and as molecular platforms for information storage and encryption [17].

One of the most thoroughly investigated radical‐based dynamic covalent systems is the reversible recombination of air‐stable aryldicyanomethyl radicals to their corresponding σ‐dimers [18, 19, 20]. A *para*‐substituent is essential to suppress unproductive head‐to‐tail dimerisation and, simultaneously, governs the stability of both the carbon‐centred radical and the σ‐bonded dimer through the captodative effect [21, 22, 23]. This effect is most pronounced when electron‐donating *para*‐substituents synergise with the electron‐withdrawing cyano‐groups, resulting in enhanced spin delocalisation and increased radical persistence [24]. As demonstrated by the fundamental works of Winter et al., the introduction of *para*‐substituents with systematically varied electronic properties enables the C–C bond dissociation energy to be tuned over a broad range, from 54 to 117 kJ mol^−1^, providing a straightforward handle to modulate the dynamic behaviour of the equilibrium [25, 26, 27, 28, 29]. However, except for strongly electron‐donating amino‐derivatives, the equilibrium overwhelmingly favours the dimeric species at ambient temperature. Efficient error correction and selective formation of thermodynamic products via DCC require sufficiently rapid equilibration, which is achieved only in systems exhibiting pronounced spin delocalisation [30]. This constraint has necessitated the use of either strongly electron‐donating substituents or extended conjugated scaffolds. Consequently, all supramolecular assemblies reported to date have been limited to amino‐substituted or highly conjugated aryldicyanomethyl radical systems [31, 32, 33, 34].

Herein, we introduce the aryldicyanomethyl radical‐based DCC as a strategy for the controlled assembly of porous organic cages. By employing an established, three‐dimensional preorganised 1,3,5‐triethylbenzene scaffold [35, 36], we envisaged systematic variation of the 2,4,6‐substituents would govern both radical and σ‐bond stability, as well as the resulting cage geometry, enabling fine‐tuned, inherently responsive cage systems (Figure 1).

**FIGURE 1:**
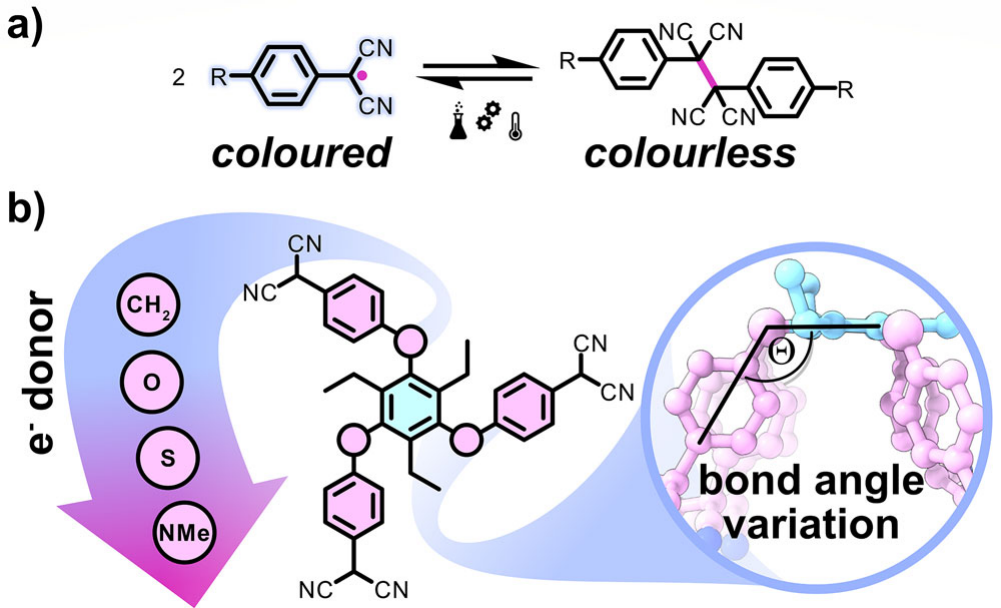
(a) Reversible dimerisation of aryldicyanomethyl radicals. (b) Schematical representation of the building block design.

The phenoxy‐substituted building block **O** was synthesised via an Ullmann‐type etherification of phenol with 1,3,5‐tribromo‐2,4,6‐triethylbenzene, followed by iodination using *N*‐iodosuccinimide and subsequent Takahashi coupling, affording **O** in four steps and an overall yield of 44% (Scheme S2). The benzyl‐ (**C**) and the thiophenoxy‐substituted (**S**) derivatives were obtained through Takahashi coupling of the corresponding iodinated or brominated precursors, which were prepared following modified literature procedures reported by Moore et al., in overall yields of 50% and 57%, respectively (Schemes S1,S3) [37]. The *N*‐methylaniline‐substituted building block **N** was synthesised by palladium‐catalysed Buchwald–Hartwig amination of 1,3,5‐tribromo‐2,4,6‐triethylbenzene with aniline, followed by *N*‐methylation, iodination with NIS, and final Takahashi coupling, yielding **N** over five steps in 75% overall yield (Scheme S4). Oxidation of the building blocks with DDQ generated the corresponding triradicals, which displayed substituent‐dependent assembly behaviour. The thiophenoxy derivative **S** selectively formed a discrete Tri^2^ species in near quantitative yield at elevated temperatures of 110 °C, consistent with the reduced radical stabilisation of the thioether substituent, whereas the *N*‐methylaniline analogue **N** afforded the tetrahedral Tri^4^ cage in 83% yield under significantly milder conditions. Notably, the ^1^H NMR spectrum of **N^4^** exhibited an additional set of aromatic proton signals of equal intensity, which can be attributed to restricted rotation of the aniline moiety within the cage framework (Figure 2).

**FIGURE 2:**
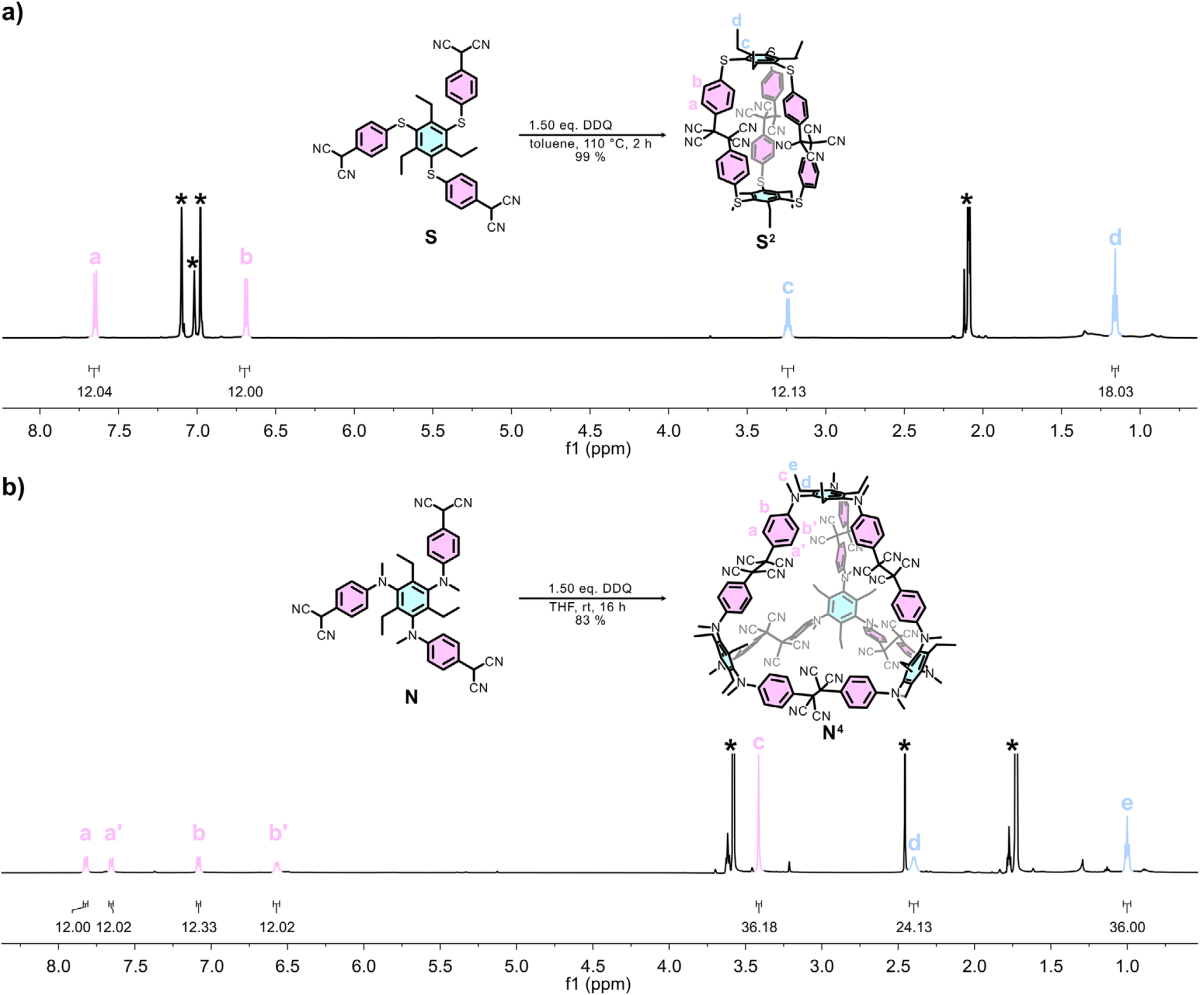
Synthesis and corresponding ^1^H NMR spectrum (600 MHz, 298 K) of (a) thioether‐based organic cage **S^2^** in toluene‐d_8_ and (b) amine‐based organic cage **N^4^** in THF‐d_8_ (* residual solvent signals).

Size determination using ^1^H DOSY experiments gave solvodynamic radii of r_solv_ = 7.25 Å (D = 5.37 · 10^−10^ m^2^ s^−1^ in toluene‐d_8_) for **S^2^** (Figure S2) and r_solv_ = 11.7 Å (D = 4.07 · 10^−10^ m^2^ s^−1^ in THF‐d_8_) for **N^4^** (Figure S4), respectively, consistent with their discrete dimeric and tetrameric architectures. Notably, **S^2^** could be detected by HRMS (ESI), reflecting the increased stability of the recombined σ‐bonds arising from the comparatively weaker electron‐donating character of the thioether substituent (Figure S92). In contrast, the phenoxy derivative **O**, which requires higher temperatures to undergo sufficient exchange, failed to form discrete assemblies and instead decomposed at elevated temperatures, highlighting the inability of phenoxy‐substituted aryldicyanomethyl radicals to support supramolecular structure formation. Similarly, the less electron‐rich benzyl‐substituted system **C** yielded only oligomeric species and underwent side reactions at elevated temperatures, likely due to the increased susceptibility of the benzylic position towards irreversible radical pathways, leading to unproductive recombination and oligomer formation. Collectively, these observations underscore the critical requirement for sufficient radical stabilisation to enable reversible C–C bond formation and, consequently, controlled cage assembly.

Octahedral‐like crystals of **N^4^** suitable for single‐crystal x‐ray diffraction (SC‐XRD) analysis were obtained by slow evaporation of a THF solution at 4°C (Figure 3a). **N^4^** crystallises in the monoclinic space group *P*2_1_/c and adopts a truncated tetrahedral cage geometry. The structure features an all‐*anti* arrangement of the recombined C–C σ‐bonds, enforced by the three‐dimensional organisation of the tritopic building blocks, with an average dihedral angle of 175.5°, resulting in a slight bending along the cage axis. This enforced *anti*‐configuration introduces pronounced structural directionality, thereby increasing architectural predictability, a key requirement for reliable cage self‐assembly (Figure 3b). The average angle Θ between the aniline edges and the central triethylbenzene core amounts to 121.9°, slightly lower than the ideal value of 125.2° for a perfect tetrahedron, highlighting the conformational flexibility of the dynamically formed C–C bonds [35]. Remarkably, the non‐constrained σ‐bonds exhibit an average C–C bond length of approximately 1.55 Å, comparable to conventional C–C single bonds, indicative of a potentially thermodynamically stabilised cage architecture [34]. In the crystalline solid‐state, the cages of **N^4^** pack through a network of weak intermolecular N–H and N–C interactions, giving rise to a three‐dimensional, potentially porous lattice that is filled with THF solvent molecules (Figure 3c).

**FIGURE 3:**
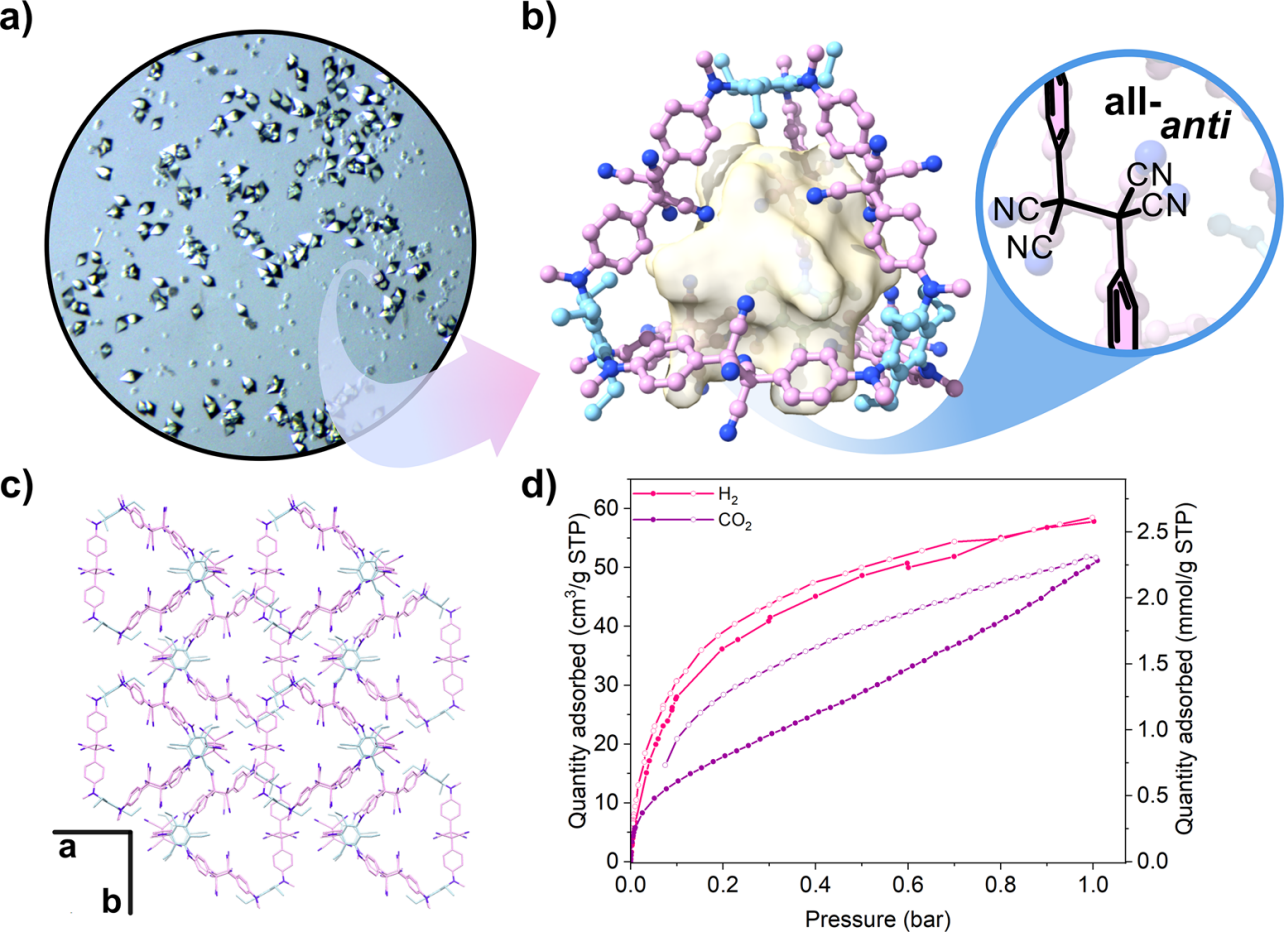
(a) Image of the octahedral‐like crystals of **N^4^**. (b) Structure of **N^4^** obtained from SC‐XRD with internal pore volume and inlet showing the all‐*anti* configuration (cage cavity volume of 549 Å^3^ calculated and visualised with CageCavityCalc) [43]. (c) View of the crystal packing of **N^4^** along the crystallographic *c*‐axis, with solvent molecules omitted for clarity. (d) Gas adsorption (filled symbols) and desorption (open symbols) isotherms of **N^4^** for H_2_ at 77 K, CO_2_ at 195K.

The crystalline packing motif suggests permanent porosity, motivating a detailed investigation of the gas sorption properties of **N^4^**. Especially dynamic imine‐based POCs [2, 4, 5, 7, 8, 9], as well as discrete boronate‐ester‐based cages [2, 7, 38, 39], have emerged as discrete, solution‐processable porous hosts [40] with finely tuneable topology and function that can also be incorporated into complex networks [3, 41, 42]. Gas adsorption measurements reveal that **N^4^** behaves as an ultramicroporous material with a pronounced size‐ and polarity‐selective sorption profile. No measurable uptake of N_2_ at 77 K or CH_4_ at 273 K was observed (Figure S12–S13), consistent with pore apertures that kinetically exclude larger, less polar gases. In contrast, **N^4^** exhibits a significant CO_2_ uptake between 195–293 K (Figure S10). At 195 K and 1 bar, a CO_2_ uptake of 2.30 mmol g^−1^ (9.2 wt%) was reached (Figure 3d), corresponding to approximately six CO_2_ molecules per cage molecule. Overall, the isotherm appears as a composite of Type I and II, with a H4 isotherm. This is in line with aggregated crystals of a microporous material where the Type II adsorption branch at higher pressure stems from the meso‐ to macroporous interparticle voids. From temperature‐dependent CO_2_ adsorption isotherms, an isosteric enthalpy of adsorption of −41 kJ mol^−1^ at a loading of 0.01 mmol g^−1^ was derived (Figure S15). This enhanced affinity is attributed to the favourable match between the kinetic diameter of CO_2_ (3.30 Å) and the ultramicroporous dimensions, combined with attractive host–guest interactions arising from the presence of 36 Lewis‐basic nitrogen atoms in one cage molecule decorating the inner pore surface. Notably, **N^4^** also shows H_2_ uptake at 77 K, reaching 2.58 mmol g^−1^ (0.52 wt%) at 1 bar (Figure 3d), further corroborating the presence of permanent ultramicroporousity. Consistent with these observations, grand canonical Monte Carlo (GCMC) calculations estimate an intrinsic limiting pore window of 3.4 Å with a narrow pore size distribution, in good agreement with the value of 3.6 Å extracted from the SC‐XRD analysis (Figure S16). The calculations further corroborate the presence of an extrinsic pore with a diameter of 5.2 Å, as observed in the crystal structure. The small pore apertures further enable reversible water vapour uptake, reaching 3.6 mmol g^−1^ (6.1 wt%) at 99% relative humidity and 293 K, corresponding to approximately 10 water molecules per cage molecule (Figure S14).

To investigate the reversible C–C bond dissociation and the resulting responsive behaviour of the cages, variable‐temperature UV/Vis‐ (VT‐UV/Vis) and electron paramagnetic resonance (VT)‐EPR spectroscopy were performed in solution and in the solid‐state. VT‐UV/Vis spectra of **N^4^** in toluene revealed a temperature‐dependent population of dissociated aryldicyanomethyl radicals, in close analogy to previously reported triphenylamine‐based dicyanomethyl systems [31]. At lower temperatures, no absorption attributable to the radical species was observed in the visible region. Upon increasing the temperature, a characteristic radical band at around 638 nm emerged and intensified, accompanied by a pronounced blue colouration of the solution. Concurrently, the absorption band at 369 nm increased, while the band at around 302 nm, assigned to the diphenylmethylamine moieties, decreased in intensity, consistent with a thermally induced shift of the radical‐dimer equilibrium (Figure 4a). An analogous temperature‐dependent behaviour was observed for **S^2^** in toluene, featuring a radical absorption band at around 616 nm. However, due to the substantially lower radical population arising from the enhanced stability of the recombined σ‐bonds, measurement required a 1000‐fold higher concentration (Figure S17). These trends were corroborated by VT‐EPR spectroscopy, which showed a pronounced increase in radical signal intensity at elevated temperatures (**N^4^**: g_e_ = 2.0034; **S^2^**: g_e_ = 2.0040), whereas at room temperature the radical population in solution was nearly undetectable (Figures 4b and S19). In the solid‐state, **S^2^** exhibited a highly temperature‐sensitive increase in radical signal intensity (g_e_ = 2.0040), highlighting its thermoresponsive behaviour (Figure 4c). In contrast, **N^4^** (g_e_ = 2.0033) showed a markedly attenuated temperature dependence (Figure 4d), which we attribute to strong local preorganisation within the tetrahedral cage architecture. This preorganisation enforces short initial fragment distances and promotes ultrafast geminate recombination, even in the absence of long‐range crystalline order [44]. Comparison of the solid‐state EPR spectra of the two organic cages revealed a substantially lower radical population for **S^2^** relative to **N^4^**, reflecting the higher thermodynamic stability of its recombined σ‐bonds. Mechanical grinding disrupts this local organisation, increasing structural heterogeneity and reducing the efficiency of geminate encounters, thereby leading to a pronounced increase in the detectable radical population. Owing to the three‐dimensional preorganisation of the cages, the radicals slowly recombine over time. This effect suffers from prolonged regeneration time because of the limited solid‐state mobility. Exposure to THF vapour accelerates this recombination effect, resulting in total recovery of the original EPR signal intensity and full decolouration of the material, thus demonstrating a vapour‐induced self‐healing of the material (Figures 4f,g and S23) [16, 45].

**FIGURE 4:**
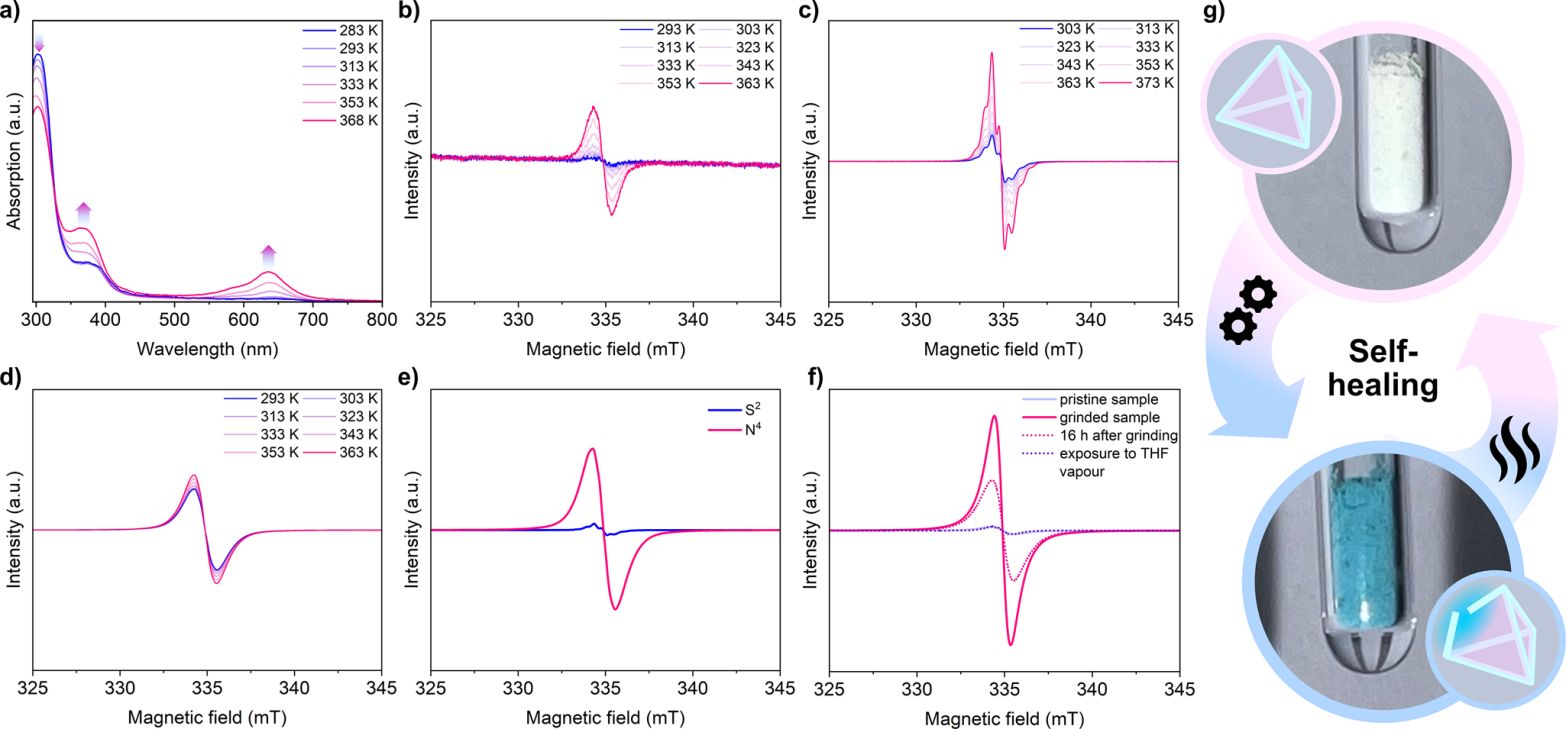
(a) VT‐UV/Vis absorption spectrum of **N^4^** in toluene (1·10^−6^ M). VT‐EPR spectra of (b) **N^4^** in toluene (1·10^−4^ M), (c) **S^2^** in solid‐state, (d) **N^4^** in solid‐state. (e) Comparison of EPR spectra of **S^2^** and **N^4^** in solid‐state at 293 K. (f) EPR spectrum of **N^4^** (9:1 mixture of NaCl:**N^4^**) in the solid‐state, after grinding, 16 h after grinding, and after exposure to THF vapour. (g) Images of **N^4^** (9:1 mixture of NaCl:**N^4^**) after grinding (bottom) and after THF vapour treatment (top), with a schematic representation of the reversible association/dissociation behaviour.

In summary, we introduced radical recombination of carbon‐centred radicals as a new dynamic covalent strategy for the controlled assembly of C–C‐linked porous organic cages. Systematic variation of a single substituent within a preorganised tritopic building block enables precise tuning of radical stability, σ‐bond strength, and cage topology, allowing selective access to discrete Tri^2^ and Tri^4^ architectures. Structural and spectroscopic analyses confirm well‐defined cage formation and permanent porosity in the tetrahedral Tri^4^ system. The narrow pore apertures, combined with the intrinsic presence of numerous Lewis‐basic binding sites, give rise to a highly CO_2_‐ and H_2_‐selective sorption profile. The reversible homolytic C–C linkage embedded within the cage framework imparts intrinsic thermo‐ and mechano‐responsiveness, including vapour‐assisted self‐healing, without the need for additional responsive subunits, enabling architectures that are both self‐correcting and functionally adaptive by design, ultimately paving the way for responsive molecular capsules and frameworks for targeted guest release, selective sorption and beyond.

## Author Contributions

**Yannic Hartmann**: conceptualization, investigation, writing – original draft, methodology, visualization, writing – review and editing. **Robert Oestreich**: investigation. **Yuki Wada**: methodology, formal analysis, data curation, resources, writing – review and editing. **Philippe de Bary**: investigation. **Masaki Kawano**: funding acquisition, resources, supervision. **Christoph Janiak**: writing – review and editing, resources, supervision. **Bernd M. Schmidt**: conceptualization, investigation, funding acquisition, writing – original draft, methodology, validation, visualization, writing – review and editing, project administration, supervision, resources, data curation.

## Conflicts of Interest

The authors declare no conflicts of interest.

## Supporting information




**Supporting File 1**: The authors have cited additional references within the Supporting Information [47, 48, 49, 50, 51, 52, 53].

**Supporting File 2**: anie72547‐sup‐0002‐cif.zip.

## Data Availability

The data that supports the findings of this study are available in the supporting information of this article. Obtained ‐X‐ray data was deposited in the CCDC [46].
