# Supplementary material for: Dynamic Covalent Radical Recombination for the Assembly of Tuneable Responsive Porous Organic Cages
Source: Angew Chem Int Ed Engl. 2026 May 9;65(26):e7638565. doi: 10.1002/anie.7638565 (PMC13285465; doi:10.1002/anie.7638565)
Supplement: Supplementary file 1 — Supporting File 1: The authors have cited additional references within the Supporting Information [47, 48, 49, 50, 51, 52, 53]. [file ANIE-65-e7638565-s001.pdf]

Contents

Contents .... **I**

1 General considerations **1**

2 Experimental details **3**

2.1 Synthesis of C **3**

2.1.1 Synthesis of 1,3,5-tris(bromomethyl)-2,4,6-triethylbenzene **1** 3

2.1.2 Synthesis of (((2,4,6-triethylbenzene-1,3,5-triyl)tris(methylene))tris(benzene-4,1-diyl))tris(trimethylsilane) **2** 4

2.1.3 Synthesis of 4,4',4''-((2,4,6-triethylbenzene-1,3,5-triyl)tris(methylene))tris(iodobenzene) **3** 5

2.1.4 Synthesis of 2,2',2''-(((2,4,6-triethylbenzene-1,3,5-triyl)tris(methylene))tris(benzene-4,1-diyl))trimalononitrile **C** 5

2.2 Synthesis of O **7**

2.2.1 Synthesis of 1,3,5-tribromo-2,4,6-triethylbenzene **4** 7

2.2.2 Synthesis of ((2,4,6-triethylbenzene-1,3,5-triyl)tris(oxy))tribenzene **5** 8

2.2.3 Synthesis of 4,4',4''-((2,4,6-triethylbenzene-1,3,5-triyl)tris(oxy))tris(iodobenzene) **6** 9

2.2.4 Synthesis of 2,2',2''-(((2,4,6-triethylbenzene-1,3,5-triyl)tris(oxy))tris(benzene-4,1-diyl))trimalononitrile **O** 10

2.3 Attempted Syntheses of C- and O-based Cage Systems **11**

2.4 Synthesis of S **12**

2.4.1 Synthesis of (2,4,6-triethylbenzene-1,3,5-triyl)tris(phenylsulfane) **7** 12

2.4.2 Synthesis of (2,4,6-triethylbenzene-1,3,5-triyl)tris((4-bromophenyl)sulfane) **8** 13

2.4.3 Synthesis of 2,2',2''-(((2,4,6-triethylbenzene-1,3,5-triyl)tris(sulfanediyl))tris(benzene-4,1-diyl))trimalononitrile **S** 13

2.5 Synthesis of S^2 .... **15**

2.6 Synthesis of N **16**

2.6.1 Synthesis of 2,4,6-triethyl-N^1,N^3,N^5-triphenylbenzene-1,3,5-triamine **9** 17

2.6.2 Synthesis of 2,4,6-triethyl-N^1,N^3,N^5-trimethyl-N^1,N^3,N^5-triphenylbenzene-1,3,5-triamine **10** 18

2.6.3 Synthesis of 2,4,6-triethyl-*N*[1],*N*[3],*N*[5]-tris(4-iodophenyl)-*N*[1],*N*[3],*N*[5]-trimethylbenzene-1,3,5-triamine **11** 19

2.6.4 Synthesis of 2,2',2''-(((2,4,6-triethylbenzene-1,3,5-triyl)tris(methylazanediyl))tris(benzene-4,1-diyl))trimalononitrile **N** 20

2.7 Synthesis of N⁴ 22

3 ¹H DOSY experiments 23

4 Crystallographic details 26

5 PXRD measurements 29

6 Gas adsorption measurements 30

7 UV/Vis spectroscopy 34

8 EPR spectroscopy 35

9 VT-NMR spectra 40

10 Stability Investigation 44

11 Thermogravimetric Analysis 47

12 Spectra 48

13 References 100

1 General considerations

Chemicals and solvents were purchased from BLD Pharmatech Ltd., Fisher Scientific International, Inc., Sigma-Aldrich Chemie GmbH, Tokyo Chemical Industry Co., Ltd., and VWR International GmbH and were used without further purification. Dry solvents were obtained from an MBraun Inertgas-Systeme GmbH MB-SPS-800 solvent purification system. Reactions were monitored by thin-layer chromatography using silica-coated aluminium plates F_{254} from Macherey-Nagel (ALUGRAM® Xtra SIL G/UV254). Purification *via* column chromatography was performed with silica gel from Macherey-Nagel (Silica 60M, 0.040–0.063 mm). The corresponding eluents are stated individually for each reaction. The solvents were removed under reduced pressure by using a rotary evaporator at a 50 °C water bath temperature, if not stated otherwise.

NMR spectroscopy

All NMR spectra were measured on a Bruker Avance III – 300, a Bruker Avance III – 600, and a Bruker Avance NEO Evo – 600 spectrometer at 293 K using deuterated solvents with their residual protonated solvent signals as internal standards for ^{1}H and $^{13}C[^{1}H]$ spectra: $CDCl_3$ (δ_H 7.26 ppm, δ_C 77.16 ppm), THF-d_8 (δ_H 3.58 ppm, δ_H 1.72 ppm, δ_C 67.21 ppm, δ_C 25.31 ppm), and toluene-d_8 (δ_H 7.09 ppm, δ_H 7.01 ppm, δ_H 6.97 ppm, δ_H 2.08 ppm, δ_C 137.48 ppm, δ_C 128.87 ppm, δ_C 127.96 ppm, δ_C 125.13 ppm, δ_C 20.43 ppm). The multiplicity is abbreviated as follows: singlet (s), doublet (d), triplet (t), quartet (q), multiplet (m), and broad (br).

IR spectroscopy

Infrared spectra were measured with a Shimadzu IR Affinity-1 with ATR technique, and the intensities were characterised as strong (s), middle (m) and weak (w).

Mass spectrometry

EI mass spectra were measured with a Triple-Quadrupol-spectrometer TSQ 7000 of the company Finnigan MAT. MALDI spectra were measured with a MALDI/TOF UltrafleXtreme of the company Bruker Daltonics GmbH & Co. KG. ESI mass spectrometry was performed on a UHR-QTOF maXis 4G (Bruker Daltonics GmbH & Co. KG).

UV/Vis spectroscopy

UV/vis spectroscopy was performed on a Cary 60 UV-Vis spectrophotometer of the company Agilent Technologies. Temperature regulation was achieved via a Cary Single Cell Peltier Accessory by Agilent Technologies. Samples were allowed to equilibrate for 10 minutes at the desired measurement temperature.

EPR spectroscopy

EPR measurements were performed using a Bruker Magnettech ESR5000 X-band spectrometer (≈ 9.4 GHz). Spectra were recorded at various temperatures using a liquid nitrogen-based evaporation cooling system. Data processing was conducted using the Magnettech software, which automatically corrected the baseline, smoothed the spectra, and determined the double integral. Liquid samples were purged with argon prior to the measurement. Samples were allowed to equilibrate for 5 minutes at the desired measurement temperature. Magnetic field sweeps were recorded in the range of 325–345 mT. Spectra were acquired using a modulation frequency of 100 kHz and a modulation amplitude of 0.2 mT. The microwave power was set to 10 mW. The number of data points was 60000 for each measurement. Sweep times (60 s or 90 s) and the number of accumulations (1–5) were adjusted as required and are reported for each measurement.

Gas sorption measurements

The gas sorption measurements of $\mathbf{N^4}$ were performed on a BELSorp-max II by MicrotracBEL Corporation. The crystals obtained by slow evaporation of a THF solution at 4 °C were activated by repeated solvent exchange with n-pentane and subsequent activation at room temperature at 10^{-2} mbar for about 16 hours prior to the measurement.

PXRD

PXRD measurements were obtained using a Rigaku MiniFlex 600 powder diffractometer (600 W, 40 kV, 15 mA) in θ/2θ geometry with Cu-K_α radiation (λ = 1.54184 A) at 293 K and equipped with a rotating low-background silicon sample holder. The highest reflex was normalised to 1.

TGA

Thermogravimetric analysis was carried out under nitrogen using a NETZSCH TG 209 F3 Tarsus in a temperature range of 30 °C to 500 °C at a step rate of 30 °C/min.

2 Experimental details

2.1 Synthesis of C

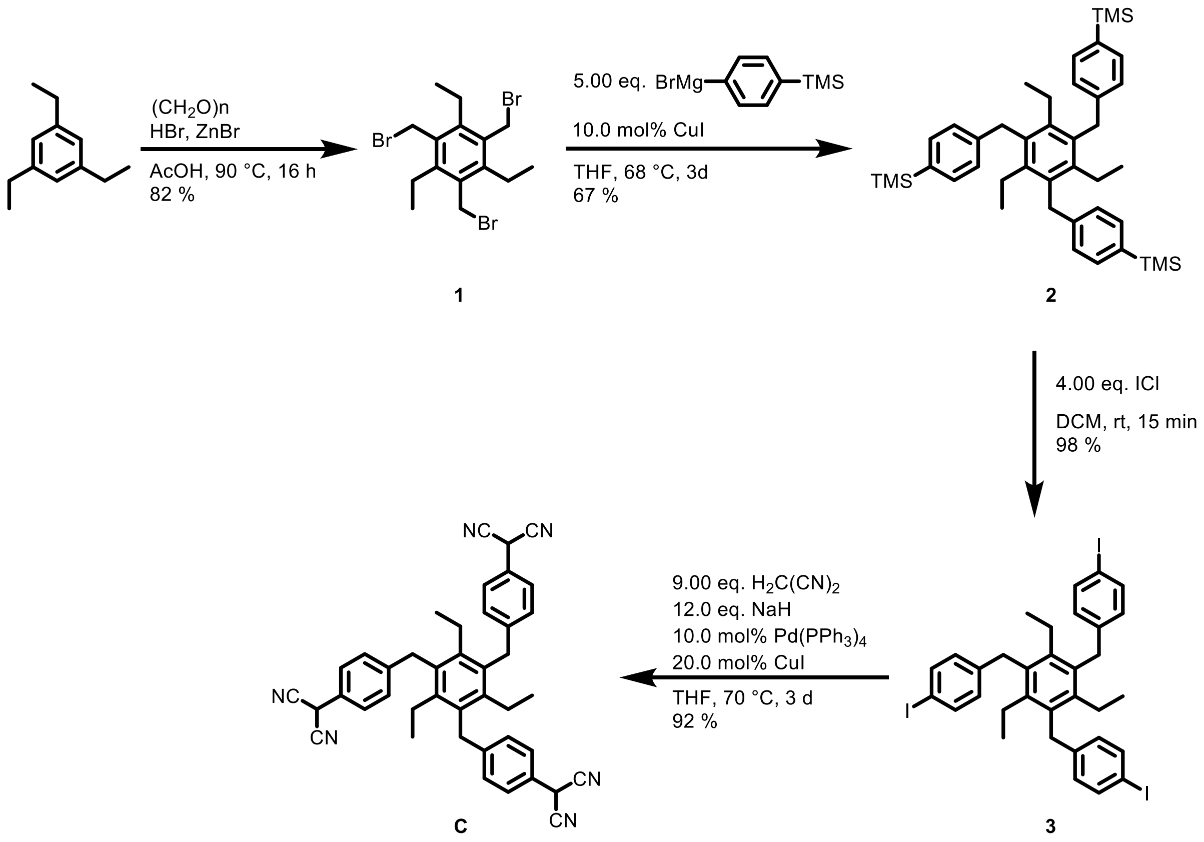

Scheme S1: Synthetic overview for **C**.

2.1.1 Synthesis of 1,3,5-tris(bromomethyl)-2,4,6-triethylbenzene 1

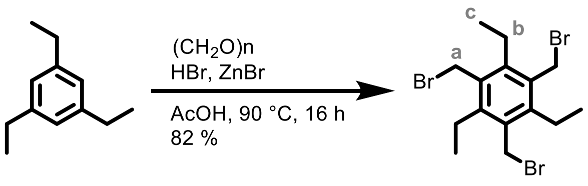

1

At room temperature, hydrobromic acid (100 mL, 33 % in acetic acid) was added to 1,3,5-triethylbenzene (10.0 mL, 53.1 mmol, 1.00 eq.) and paraformaldehyde (16.7 g, 558 mmol, 10.5 eq.). Under rigorous stirring, $ZnBr_2$ (19.7 g, 87.6 mmol, 1.65 eq.) was slowly added. After complete addition, the mixture was stirred vigorously at 90 °C for 60 hours. The reaction mixture was then cooled to room temperature, and the colourless solid was filtered off, washed with acetic acid (100 mL) and water (300 mL), and dried *in vacuo* to yield **1** (19.1 g, 43.3 mmol, 82 %) as a colourless solid.

^{1}H NMR (300 MHz, CDCl$_3$): δ = 4.58 (s, 6 H, **H$_a$**), 2.94 (q, ^3J = 7.6 Hz, 6 H, **H$_b$**), 1.34 (t, ^3J = 7.6 Hz, 9 H, **H$_c$**).

All the analytical data obtained was in accordance with the literature.[1]

2.1.2 Synthesis of (((2,4,6-triethylbenzene-1,3,5-triyl)tris(methylene))tris(benzene-4,1-diyl))tris(trimethylsilane) 2

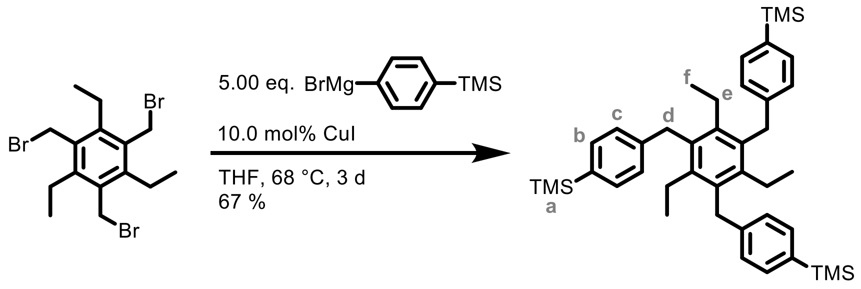

1 **2**

To a 50 mL two-neck round-bottom flask charged with activated magnesium turnings (0.729 g, 30.0 mmol, 10.0 eq.) equipped with a reflux condenser was slowly added 1-bromo-4-(trimethylsilyl)benzene (2.93 mL, 15.0 mmol, 5.00 eq.) in 15 mL dry THF under nitrogen. After stirring for 1.5 h at room temperature, the mixture was diluted with an additional 15 mL dry THF and transferred to a separate 100 mL two-neck round-bottom flask containing **1** (1.32 g, 3.00 mmol, 1.00 eq.), CuI (57 mg, 0.30 mmol, 10.0 mol%), and 10 mL dry THF. The mixture was then stirred under nitrogen for 3 days at 70 °C. After cooling to room temperature, the reaction was quenched by the addition of 10 mL of saturated aqueous NH_4Cl solution. A further of 50 mL water was added, and the mixture was extracted with 3x30 mL dichloromethane and subsequently washed with 3x30 mL brine. The organic phase was dried using $MgSO_4$, the solvent was removed under reduced pressure, and the product was purified *via* column chromatography using *n*-hexane and then a 94/6 *n*-hexane/dichloromethane mixture as eluent to yield **2** (1.30 g, 2.00 mmol, 67 %) as a colourless solid.**^{1}H NMR (600 MHz, CDCl$_3$):** δ = 7.42 (d, ^3J = 7.9 Hz, 6 H, **H$_b$**), 7.02 (d, ^3J = 7.8 Hz, 6 H, **H$_c$**), 4.14 (s, 6 H, **H$_d$**), 2.45 (q, ^3J = 7.5 Hz, 6 H, **H$_e$**), 1.11 (t, ^3J = 7.5 Hz, 9 H, **H$_f$**), 0.26 (s, 27 H, **H$_a$**).

All the analytical data obtained was in accordance with the literature.[2]

2.1.3 Synthesis of 4,4',4''-((2,4,6-triethylbenzene-1,3,5-triyl)tris(methylene))tris(iodobenzene) 3

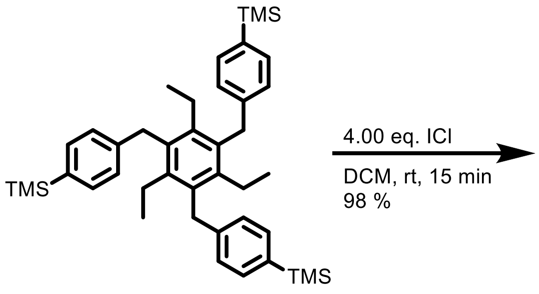

2 **3**

To a solution of **2** (1.23 g, 1.90 mmol, 1.00 eq.) in 120 mL dry dichloromethane in a 250 mL Schlenk round-bottom flask was dropwise added a 1M solution of ICl in dichloromethane (7.6 mL, 7.60 mmol, 4.00 eq.) at 0 °C and stirred for 15 minutes. The reaction was quenched by the addition of a saturated aqueous Na_2SO_3 solution, and the reaction mixture was extracted with 2x10 mL dichloromethane and subsequently washed with 20 mL saturated aqueous Na_2SO_3 solution. The organic phase was dried with $MgSO_4$, and the solvent was removed under reduced pressure. This yielded **3** (1.51 g, 1.87 mmol, 98 %) as a colourless foam.

^{1}H NMR (600 MHz, CDCl$_3$): δ = 7.57 (d, ^3J = 7.8 Hz, 6 H, **H$_a$**), 6.72 (d, ^3J = 7.8 Hz, 6 H, **H$_b$**), 4.06 (s, 6 H, **H$_c$**), 2.38 (q, ^3J = 7.5 Hz, 6 H, **H$_d$**), 1.04 (t, ^3J = 7.4 Hz, 9 H, **H$_e$**).

All the analytical data obtained was in accordance with the literature.[2]

2.1.4 Synthesis of 2,2',2''-(((2,4,6-triethylbenzene-1,3,5-triyl)tris(methylene))tris(benzene-4,1-diyl))trimalononitrile C

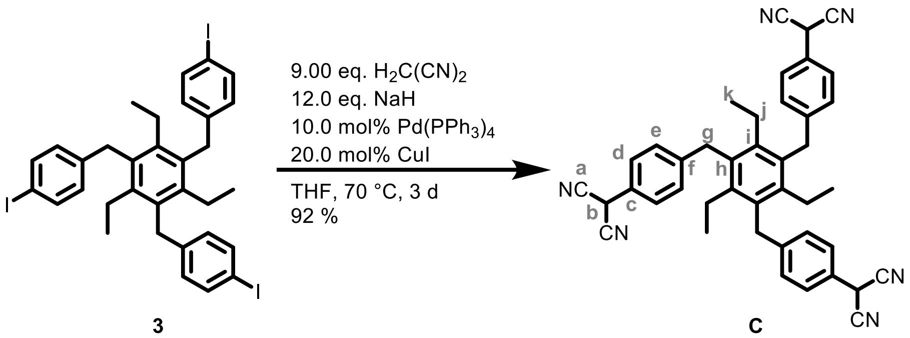

3 **C**

In a 10 mL Schlenk tube were added NaH (0.096 g, 2.40 mmol, 12.0 eq.; 60 % in mineral oil) and 1.5 mL dry THF under nitrogen. The mixture was then cooled to 0 °C and malononitrile (0.119 g, 1.80 mmol, 9.00 eq.) in 0.5 mL dry THF was slowly added under vigorous stirring.

After the addition, the mixture was stirred for another 30 minutes at room temperature. Finally, **3** (0.162 g, 0.200 mmol, 1.00 eq.), $Pd(PPh_3)_4$ (0.023 g, 0.020 mmol, 10.0 mol%) and CuI (0.008 g, 0.040 mmol, 20.0 mol%) were added, and the mixture was stirred at 70 °C for 3 days, after which the reaction was quenched by the addition of 1 mL 3M HCl at 0 °C. The mixture was extracted with 3 x 10 mL of dichloromethane, washed with 10 mL of water, and the organic phase was dried with $MgSO_4$. After removal of the solvent under reduced pressure, the mixture was suspended in 2 mL methanol, further precipitated by addition of 10 mL water and filtrated. The solid was suspended once more in 2 mL methanol and 10 mL water were added again to precipitate **C** (0.115 g, 0.184 mmol, 92 %) as a brown solid.

^{1}H NMR (600 MHz, $CDCl_3$): δ = 7.41 (d, ^3J = 8.0 Hz, 6 H, **H_d**), 7.12 (d, ^3J = 7.9 Hz, 6 H, **H_e**), 5.03 (s, 3 H, **H_b**), 4.18 (s, 6 H, **H_g**), 2.40 (q, ^3J = 7.7 Hz, 6 H, **H_j**), 1.07 (t, ^3J = 7.5 Hz, 9 H, **H_k**).

^{13}C{^{1}H} NMR (151 MHz, $CDCl_3$): δ = 144.16 (**C_c**), 141.78 (**C_i**), 133.51 (**C_h**), 129.36 (**C_e**), 127.52 (**C_d**), 123.91 (**C_f**), 111.97 (**C_a**), 34.45 (**C_g**), 27.90 (**C_b**), 23.92 (**C_j**), 15.25 (**C_k**).

HRMS (ESI): m/z calculated for $[C_{42}H_{36}N_6+Na]^+$: 647.2894, found: 647.2890.

IR $\tilde{\nu}$ [cm^{-1}]: 2964.59 (w), 2929.87 (w), 2895.15 (w), 2872.01 (w), 2833.43 (w), 2358.94 (w), 2339.65 (w), 2331.94 (w), 2256.71 (w), 1681.93 (w), 1608.63 (w), 1579.70 (w), 1508.33 (s), 1456.26 (w), 1436.97 (w), 1415.75 (m), 1375.25 (w), 1315.45 (w), 1253.73 (w), 1226.73 (w), 1192.01 (w), 1170.79 (w), 1020.34 (m), 921.97 (s), 839.03 (w), 800.46 (m), 767.67 (m), 754.17 (m), 734.88 (w) 694.37 (w).

2.2 Synthesis of O

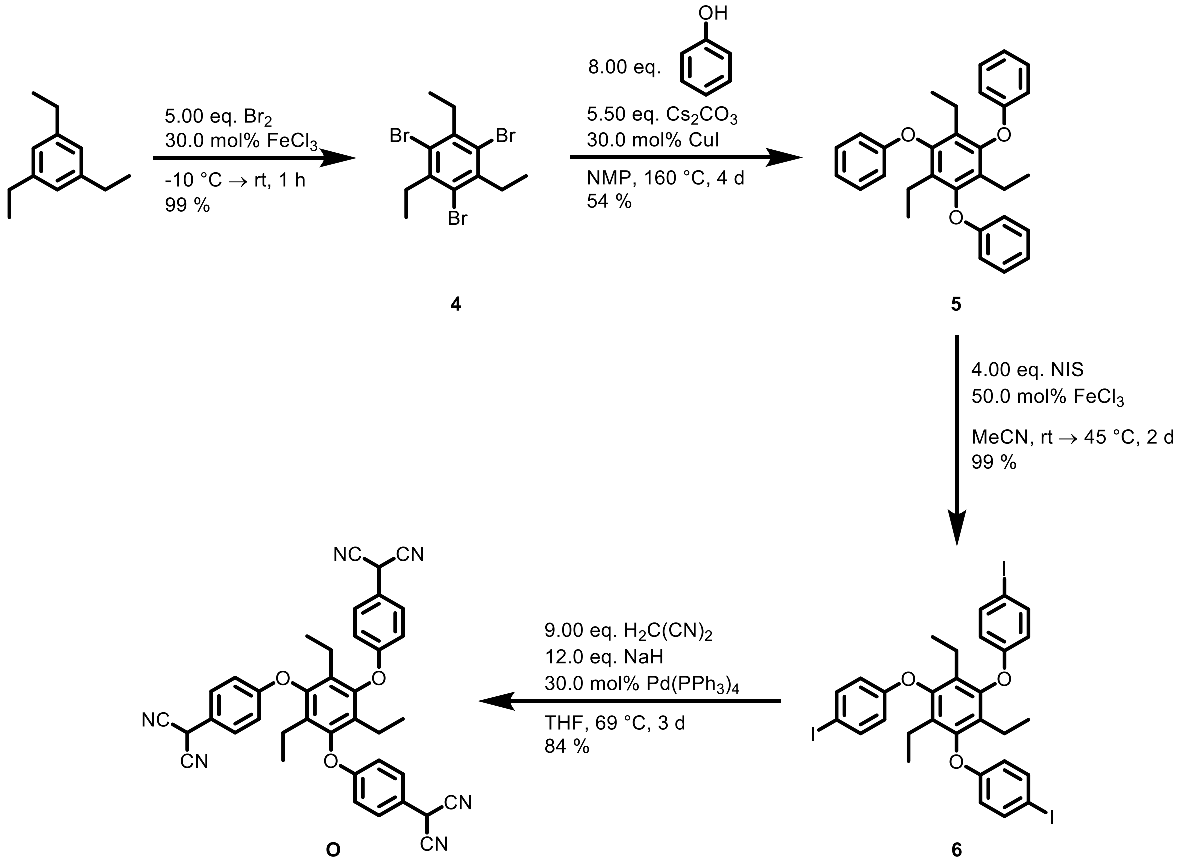

Scheme S2: Synthetic overview for **O**.

2.2.1 Synthesis of 1,3,5-tribromo-2,4,6-triethylbenzene 4

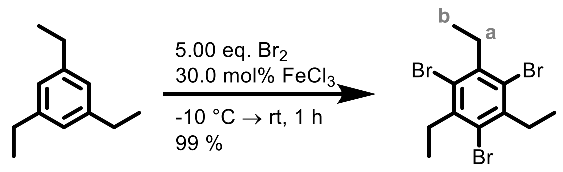

4

To a 25 mL two-neck round-bottom flask equipped with a bubbler were added iron powder (0.209 g, 3.75 mmol, 30.0 mol%) and bromine (3.20 mL, 62.5 mmol, 5.00 eq.). The mixture was cooled to -20 °C and 1,3,5-triethylbenzene (2.35 mL, 12.5 mmol, 1.00 eq.) was added dropwise under rigorous stirring over the course of 45 minutes. During the addition the bubbler was dipped into a 10 wt% aqueous KOH solution. The mixture was then stirred for an additional 30 minutes at room temperature and quenched by the addition of first 10 wt% aqueous KOH solution and then aqueous KOH/NaS_2O_4 solution. The reaction mixture was extracted with 3x30 mL dichloromethane, washed once with 30 mL brine, and the organic phase was dried with $MgSO_4$ to yield **4** (4.96 g, 12.4 mmol, 99 %) as a colourless solid.

^{1}H NMR (600 MHz, $CDCl_3$): δ = 3.13 (q, ^3J = 7.5 Hz, 6 H, **H_a**), 1.17 (t, ^3J = 7.5 Hz, 9 H, **H_b**).

All the analytical data obtained was in accordance with the literature.[3]

2.2.2 Synthesis of ((2,4,6-triethylbenzene-1,3,5-triyl)tris(oxy))tribenzene 5

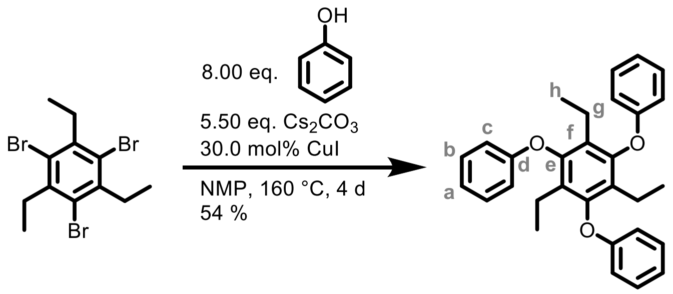

4 **5**

To a 50 mL Schlenk round-bottom flask were added **4** (2.00 g, 5.00 mmol, 1.00 eq.), Cs_2CO_3 (8.96 g, 27.5 mmol, 5.50 eq.), and CuI (0.286 g, 1.50 mmol, 30.0 mol%) under nitrogen. 10 mL NMP and finally phenol (3.76 g, 40.0 mmol, 8.00 eq.) were added, and the mixture was rigorously stirred at 160 °C for 4 days. Subsequently the reaction mixture was allowed to cool to room temperature, and 20 mL of 3M aqueous NaOH solution were added. The reaction mixture was then extracted with 4x30 mL cold diethyl ether, and the organic phase was dried with Na_2SO_4. Further purification *via* column chromatography with *n*-hexane as eluent afforded **5** (1.19 g, 2.71 mmol, 54 %) as a colourless oil which crystallised upon cooling to 4 °C.

^{1}H NMR (600 MHz, $CDCl_3$): δ = 7.31–7.25 (m, 6 H, **H_b**), 6.99 (tt, ^3J = 7.4 Hz, ^4J = 1.1 Hz, 3 H, **H_a**), 6.85–6.82 (m, 6 H, **H_c**), 2.37 (q, ^3J = 7.5 Hz, 6 H, **H_g**), 0.98 (t, ^3J = 7.5 Hz, 9 H, **H_h**).

$^{13}C\{^1H\}$ NMR (151 MHz, $CDCl_3$): δ = 158.74 (**C_d**), 150.44 (**C_e**), 129.75 (**C_b**), 129.39 (**C_f**), 121.68 (**C_a**), 114.87 (**C_c**), 18.73 (**C_g**), 13.92 (**C_h**).

HRMS (ESI): m/z calculated for $[C_{30}H_{30}O_3+H]^+$: 439.2268, found: 439.2264.

IR $\tilde{\nu}$ [cm^{-1}]: 3078.39 (w), 2970.38 (w), 2937.59 (w), 2877.79 (w), 2422.59 (w), 1581.63 (w), 1489.05 (m), 1456.26 (w), 1427.32 (m), 1371.39 (w), 1330.88 (w), 1317.38 (w), 1288.45 (w), 1257.59 (w), 1209.37 (s), 1163.08 (m), 1099.43 (s), 1074.35 (w), 1049.28 (w), 1022.27 (w), 997.20 (w), 974.05 (w), 867.97 (m), 840.96 (w), 823.60 (w), 785.03 (w), 746.45 (s), 718.45 (w), 688.59 (s), 659.66 (w), 615.29 (w).

2.2.3 Synthesis of 4,4',4"-((2,4,6-triethylbenzene-1,3,5-triyl)tris(oxy))tris(iodobenzene) 6

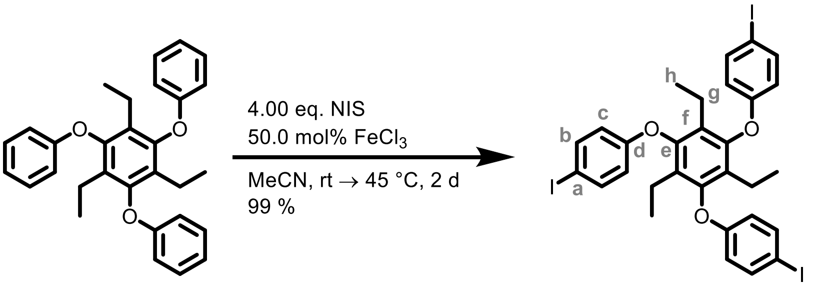

5 6

To a 10 mL Schlenk flask were added **5** (0.439 g, 1.00 mmol, 1.00 eq.), $FeCl_3$ (0.081 g, 0.500 mmol, 50.0 mol%) and 3.0 mL dry acetonitrile. The mixture was then stirred at 0 °C for 20 minutes until NIS (0.900 g, 4.00 mmol, 4.00 eq.) was added all at once and the mixture was slowly warmed to room temperature. After stirring at room temperature for two days, the mixture was stirred for an additional 4 hours at 45 °C. The reaction mixture was then poured into 50 mL water and extracted with 3 x 20 mL diethyl ether. The organic phase was dried with $MgSO_4$ and the crude product was purified by column chromatography with *n*-hexane as eluent to yield **6** (0.804 g, 0.985 mmol, 99 %) as a colourless crystalline solid.

^{1}H NMR (600 MHz, $CDCl_3$): δ = 7.58–7.54 (m, 6 H, **H_b**), 6.60–6.57 (m, 6 H, **H_a**), 2.31 (q, ^3J = 7.4 Hz, 6 H, **H_g**), 0.96 (t, ^3J = 7.5 Hz, 9 H, **H_h**).

^{13}C{^{1}H} NMR (151 MHz, $CDCl_3$): δ 158.50 (**C_d**), 150.16 (**C_e**), 138.69 (**C_b**), 129.42 (**C_a**), 117.15 (**C_c**), 84.17 (**C_f**), 18.66 (**C_g**), 13.91 (**C_h**).

EI + MS (70 eV, *m/z* (%)): 817 ($[C_{30}H_{28}I_3O_3]^+$, 84), 547 ($[C_{30}H_{28}I_3O_3]^+$, 40), 471 ($[C_{24}H_{24}IO_2]^+$, 76), 470 ($[C_{24}H_{23}IO_2]^+$, 76), 420 (16), 328 (32), 299 (31), 253 (37), 231 (31), 165 (15) , 115 (20), 76 ($[C_6H_4]^+$, 65).

IR $\tilde{\nu}$ [cm^{-1}]: 3867.28 (w), 3574.10 (w), 2723.49 (w), 2495.89 (w), 2422.59 (w), 2208.49 (w), 2160.27 (w), 2144.84 (w), 2073.48 (w), 2019.47 (w), 2005.97 (w), 1986.68 (w), 1975.11 (w), 1919.17 (w), 1874.81 (w), 1857.45 (w), 1573.91 (w), 1477.47 (s), 1463.97 (w), 1429.25 (m), 1398.39 (w), 1371.39 (w), 1321.24 (w), 1296.16 (w), 1273.02 (w), 1261.45 (w), 1211.30 (s), 1165.00 (m), 1099.43 (m), 1002.98 (m), 975.98 (w), 950.91 (w), 931.62 (w), 869.90 (w), 817.82 (m), 786.96 (w), 763.81 (w), 748.38 (w), 690.52 (w), 642.30 (w), 630.72 (w).

2.2.4 Synthesis of 2,2',2''-(((2,4,6-triethylbenzene-1,3,5-triyl)tris(oxy))tris(benzene-4,1-diyl))trimalononitrile O

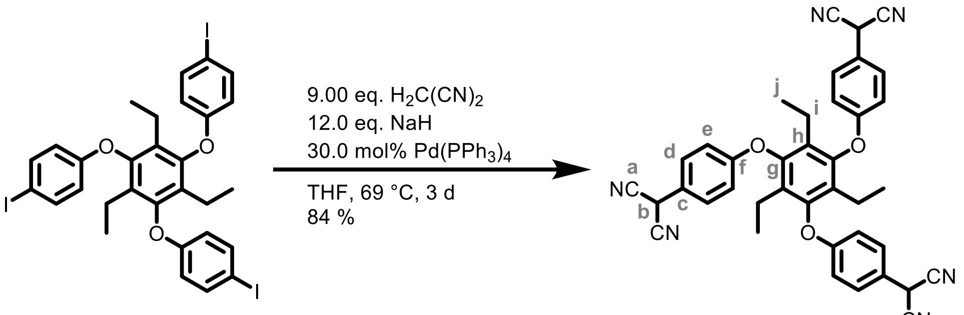

6 **O**

In a 25 mL Schlenk tube were added NaH (0.159 g, 3.96 mmol, 12.0 eq.; 60 % in mineral oil) and 3 mL dry THF under nitrogen. The mixture was then cooled to 0 °C and malononitrile (0.197 g, 2.97 mmol, 9.00 eq.) in 1 mL dry THF was slowly added under vigorous stirring. After the addition, the mixture was stirred for another 30 minutes at room temperature. Finally, **6** (0.270 g, 0.330 mmol, 1.00 eq.) and $Pd(PPh_3)_4$ (0.115 g, 0.099 mmol, 30.0 mol%) were added, and the mixture was heated to 69 °C for 3 days, after which the reaction was quenched by the addition of 2.7 mL 2M HCl at 0 °C. The mixture was extracted with 3 x 10 mL of dichloromethane, the organic phase was dried with Na_2SO_4, and the crude product was purified by column chromatography with cyclohexane/ethyl acetate 2/1 as eluent. The product was further suspended in methanol and precipitated by the addition of water to yield **O** (0.175 g, 0.277 mmol, 84 %) as a colourless solid after filtration.

^{1}H NMR (600 MHz, $CDCl_3$): δ = 7.45 (d, ^3J = 8.7 Hz, 6 H, $\mathbf{H_d}$), 6.97–6.92 (m, 6 H, $\mathbf{H_e}$), 5.02 (s, 3 H, $\mathbf{H_b}$), 2.32 (q, ^3J = 7.4 Hz, 6 H, $\mathbf{H_i}$), 0.99 (t, ^3J = 7.4 Hz, 9 H, $\mathbf{H_j}$).

^{13}C{^{1}H} NMR (151 MHz, $CDCl_3$): δ = 159.85 ($\mathbf{C_c}$), 150.05 ($\mathbf{C_g}$), 129.65 ($\mathbf{C_h}$), 129.21 ($\mathbf{C_e}$), 119.53 ($\mathbf{C_f}$), 116.26 ($\mathbf{C_d}$), 111.90 ($\mathbf{C_a}$), 27.62 ($\mathbf{C_b}$), 18.75 ($\mathbf{C_i}$), 13.90 ($\mathbf{C_j}$).

HRMS (ESI): m/z calculated for $[C_{39}H_{30}N_6O_3+Na]^+$: 653.2272, found: 653.2267.

IR $\tilde{\nu}$ [cm^{-1}]: 2978.09 (w), 2937.59 (w), 2877.79 (w), 1608.63 (w), 1587.42 (w), 1504.48 (s), 1460.11 (w), 1431.18 (m), 1319.31 (w), 1303.88 (w), 1224.80 (s), 1172.72 (m), 1099.43 (m), 1012.63 (w), 977.91 (w), 920.05 (w), 871.82 (w), 835.18 (m), 777.31 (w), 754.17 (w), 690.52 (w), 603.72 (w).

2.3 Attempted Syntheses of C- and O-based Cage Systems

From a geometric perspective, both the O- and C-based building blocks appear in principle suitable for cage formation, as their bond angles are comparable to those of the successful N-based system and should allow formation of a Tri^4 topology. Moreover, the inherent flexibility of the σ-bond provides a certain degree of geometric adaptability. However, experimentally, both systems failed to yield discrete cage structures. At lower temperatures, only oligomeric species were observed, while at elevated temperatures decomposition of the building blocks and intermediates occurred. A range of reaction conditions was explored, and the corresponding results have been summarised in the table below.

Table S1: Reaction conditions for the attempted synthesis of O- and C-based cage systems.

Building Block	Oxidizing agent	Solvent	Temperature	Time	Comment
**O**	DDQ	Acetone	rt	10 min	Oligomers
**O**	DDQ	Acetone	50 °C	2 h	Oligomers
**O**	DDQ	DCM	rt	2 h	Oligomers
**O**	$Pb(IV)O_2$	$CHCl_3$	rt	4 h	Oligomers
**O**	$K_3[Fe(CN)_6]$ / KOH	DCM/H_2O	rt	2 h	Decomposition
**O**	$K_3[Fe(CN)_6]$ / KOH	DCM/H_2O	50 °C	2 h	Decomposition
**O**	DDQ	Toluene	110 °C	16 h	Oligomers
**O**	DDQ	Toluene	110 °C	2 w	Oligomers
**O**	DDQ	*p*-Xylene	130 °C	3 h	Decomposition
**O**	DDQ	NMP	125 °C	3 h	Decomposition
**C**	DDQ	DCM	rt	16 h	Oligomers
**C**	DDQ	Toluene	110 °C	3 h	Decomposition
**C**	DDQ	*p*-Xylene	140 °C	3 h	Decomposition
**C**	DDQ	$CHCl_3$	65	2 w	Oligomers

2.4 Synthesis of S

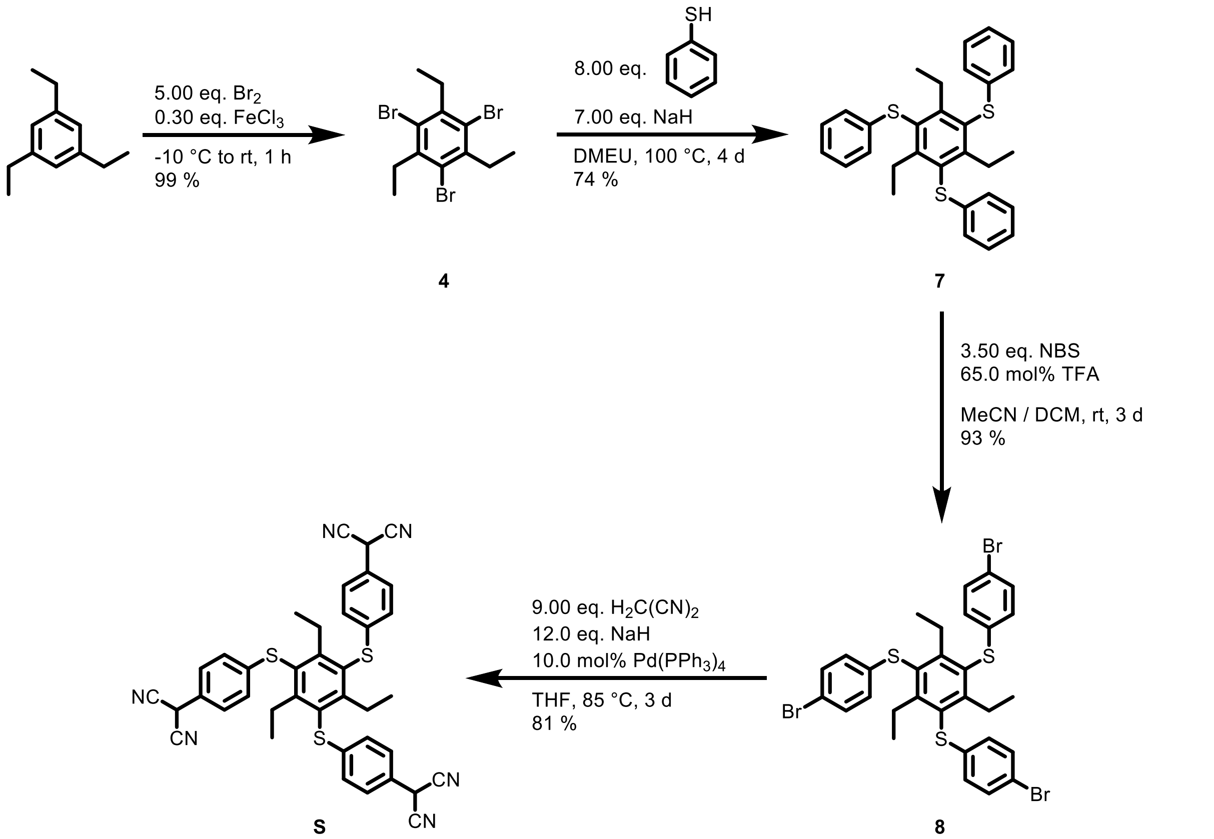

Scheme S3: Synthetic overview for **S**.

2.4.1 Synthesis of (2,4,6-triethylbenzene-1,3,5-triyl)tris(phenylsulfane) 7

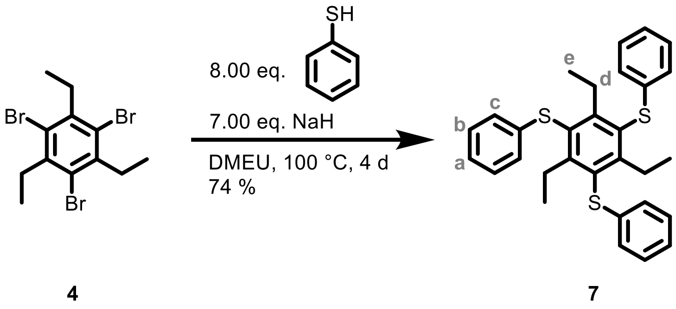

In a 100 mL Schlenk round-bottom flask were added NaH (1.40 g, 35.0 mmol, 7.00 eq.; 60 % in mineral oil) and 40 mL DMEU. Then thiophenol was added carefully (4.08 g, 40.0 mmol, 8.00 eq.) and subsequently **4** (2.00 g, 5.00 mmol, 1.00 eq.). The round-bottom flask was sealed and heated to 100 °C for 4 days. The reaction mixture was then quenched by the addition of aqueous NaOH solution and extracted with 4 x 30 mL cold diethyl ether. The organic phase was dried with Na_2SO_4, and the crude product was purified by column chromatography with *n*-hexane as eluent to yield **7** (1.79 g, 3.68 mmol, 74 %) as a colourless crystalline solid.

^{1}H NMR (600 MHz, $CDCl_3$): δ = 7.21 (t, ^3J = 7.6 Hz, 6 H, **H$_b$**), 7.08 (t, ^3J = 7.4 Hz, 3 H, **H$_a$**), 6.93–6.90 (m, 6 H, **H$_c$**), 3.16 (q, ^3J = 7.4 Hz, 6 H, **H$_d$**), 1.11 (t, ^3J = 7.4 Hz, 9 H, **H$_e$**).

All the analytical data obtained was in accordance with the literature.[2]

2.4.2 Synthesis of (2,4,6-triethylbenzene-1,3,5-triyl)tris((4-bromophenyl)sulfane) 8

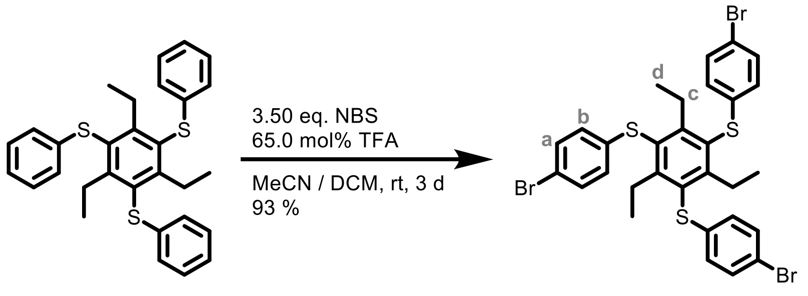

7 8

To a 100 mL Schlenk round-bottom flask were added **7** (0.974 g, 2.00 mmol, 1.00 eq.), 40 mL of an acetonitrile/dichloromethane 1/2 mixture and trifluoroacetic acid (0.100 mL, 1.30 mmol, 65.0 mol%). The mixture was cooled to 0 °C before NBS (1.25 g, 7.00 mmol, 3.50 eq.) was added all at once. After the addition, the mixture was stirred at room temperature for 3 days before 40 mL of an aqueous NaOH / $Na_2S_2O_3$ solution were added. The reaction mixture was extracted with 3 x 30 mL dichloromethane, and the organic phase was dried with Na_2SO_4 to yield **8** (1.34 g, 1.86 mmol, 93 %) as a colourless crystalline solid.

^{1}H NMR (600 MHz, $CDCl_3$): δ = 7.35–7.30 (m, 6 H, **H$_a$**), 6.77–6.72 (m, 6 H, **H$_b$**), 3.10 (q, ^3J = 7.4 Hz, 6 H, **H$_c$**), 1.08 (t, ^3J = 7.4 Hz, 9 H, **H$_d$**).

All the analytical data obtained was in accordance with the literature.[2]

2.4.3 Synthesis of 2,2',2''-(((2,4,6-triethylbenzene-1,3,5-triyl)tris(sulfanediyl))tris(benzene-4,1-diyl))trimalononitrile S

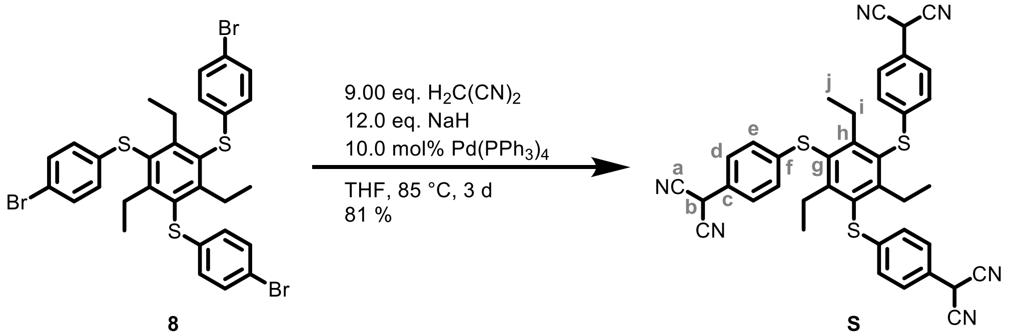

8 S

In a 50 mL Schlenk round-bottom flask were added NaH (1.44 g, 36.0 mmol, 12.0 eq.; 60 % in mineral oil) and 20 mL dry THF under nitrogen. The mixture was then cooled to 0 °C and malononitrile (1.78 g, 27.0 mmol, 9.00 eq.) in 10 mL dry THF was slowly added under vigorous stirring. After the addition, the mixture was stirred for another 30 minutes at room temperature. Finally, **8** (2.17 g, 3.00 mmol, 1.00 eq.) and $Pd(PPh_3)_4$ (0.347 g, 0.300 mmol, 30.0 mol%) were added, and the mixture was heated to reflux at 85 °C for 3 days after which the reaction was quenched by addition of 8.6 mL 2M HCl at 0 °C. The mixture was extracted with 3 x 30 mL of dichloromethane, the organic phase was dried with Na_2SO_4, and the crude product was purified *via* column chromatography with dichloromethane/methanol 99/1 as eluent. The product was further suspended in methanol and precipitated by the addition of water to yield **S** (1.65 g, 2.43 mmol, 81 %) as a grey-green solid after filtration.

^{1}H NMR (600 MHz, $CDCl_3$): δ = 7.37–7.34 (m, 6 H, **H_d**), 7.01–6.97 (m, 6 H, **H_e**), 5.02 (s, 3 H, **H_b**), 3.08 (q, ^3J = 7.3 Hz, 6 H, **H_i**), 1.13 (t, ^3J = 7.4 Hz, 9 H, **H_j**).

^{13}C NMR{^{1}H} (151 MHz, $CDCl_3$): δ = 158.99 (**C_h**), 142.80 (**C_c**), 130.09 (**C_g**), 128.03 (**C_e**), 126.42 (**C_d**), 122.95 (**C_f**), 111.79 (**C_a**), 29.34 (**C_i**), 27.72 (**C_b**), 15.77 (**C_j**).

HRMS (ESI): m/z calculated for $[C_{39}H_{30}N_6S_3+Na]^+$: 701.1587, found: 701.1583.

IR $\tilde{\nu}$ [cm^{-1}]: 3666.68 (w), 3643.53 (w), 3055.24 (w), 2962.66 (w), 2926.01 (w), 2887.44 (w), 2870.08 (w), 2754.35 (w), 2596.19 (w), 2441.88 (w), 2254.79 (w), 1903.74 (w), 1666.50 (w), 1595.13 (w), 1490.47 (s), 1448.54 (w), 1406.11 (w), 1369.46 (w), 1359.82 (w), 1317.38 (w), 1263.37 (w), 1246.02 (w), 1080.14 (m), 1055.06 (w), 1014.56 (m), 921.97 (w), 835.18 (m), 821.68 (m), 779.24 (s), 719.45 (w), 698.23 (w).

2.5 Synthesis of S^2

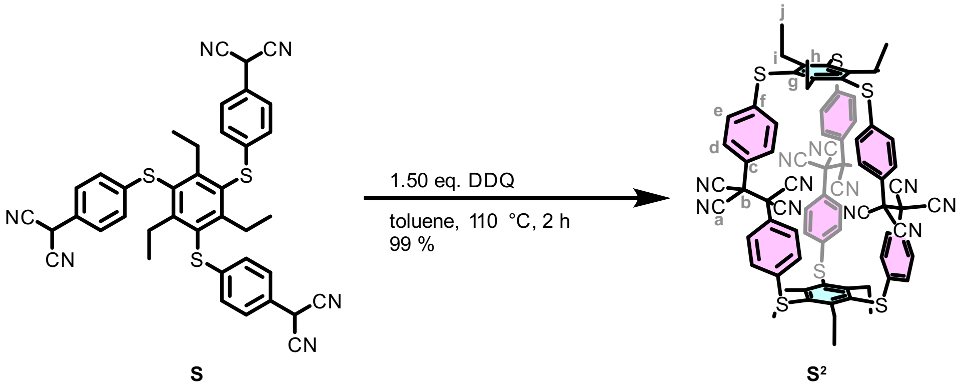

In a 25 mL Schlenk round-bottom flask was **S** (33.9 mg, 0.050 mmol, 1.00 eq.) dissolved in 7.5 mL dry toluene. Then DDQ (17.0 mg, 0.075 mmol, 1.50 eq.) in 2.5 mL dry toluene was added dropwise at room temperature. After addition, the mixture was stirred at 110 °C for 2 h. The reaction mixture was filtered through a silica plug and washed with toluene to yield **S^2** (33.4 mg, 0.0247 mmol, 99 %) as a colourless solid.

^{1}H NMR (600 MHz, toluene-d$_8$): δ = 7.67–7.63 (m, 12 H, **H$_d$**), 6.70-6.67 (m, 12 H, **H$_e$**), 3.24 (q, ^3J = 7.3 Hz, 12 H, **H$_i$**), 1.16 (t, ^3J = 7.3 Hz, 18 H, **H$_j$**).

^{13}C NMR{^{1}H} (151 MHz, toluene-d$_8$): δ = 160.06 (**C$_h$**), 144.96 (**C$_c$**), 131.69 (**C$_g$**), 128.46 (**C$_d$**), 127.16 (**C$_e$**), 124.13 (**C$_f$**), 110.50 (**C$_a$**), 54.52 (**C$_b$**), 29.69 (**C$_i$**), 16.24 (**C$_j$**).

HRMS (ESI): m/z calculated for $[C_{78}H_{54}N_{12}S_6+Na]^+$: 1373.2811, found: 1373.2798; m/z calculated for $[C_{78}H_{54}N_{12}S_6+NH_4]^+$: 1368.3257, found: 1368.3236.

IR $\tilde{\nu}$ [cm^{-1}]: 3061.03 (w), 2960.73 (w), 2927.94 (w), 2870.08 (w), 2858.51 (w), 2252.86 (w), 1901.91 (w), 1716.65 (w), 1672.28 (w), 1591.27 (w), 1514.12 (w), 1490.97 (w), 1456.26 (w), 1406.11 (w), 1371.39 (w), 1359.82 (w), 1315.45 (w), 1265.30 (m), 1190.08 (w), 1172.72 (w), 1114.86 (w), 1089.78 (w), 1078.21 (w), 1053.13 (w), 1014.56 (m), 954.76 (w), 941.26 (w), 896.90 (w), 873.75 (w), 794.67 (s), 731.02 (m), 694.37 (w), 665.44 (w).

2.6 Synthesis of N

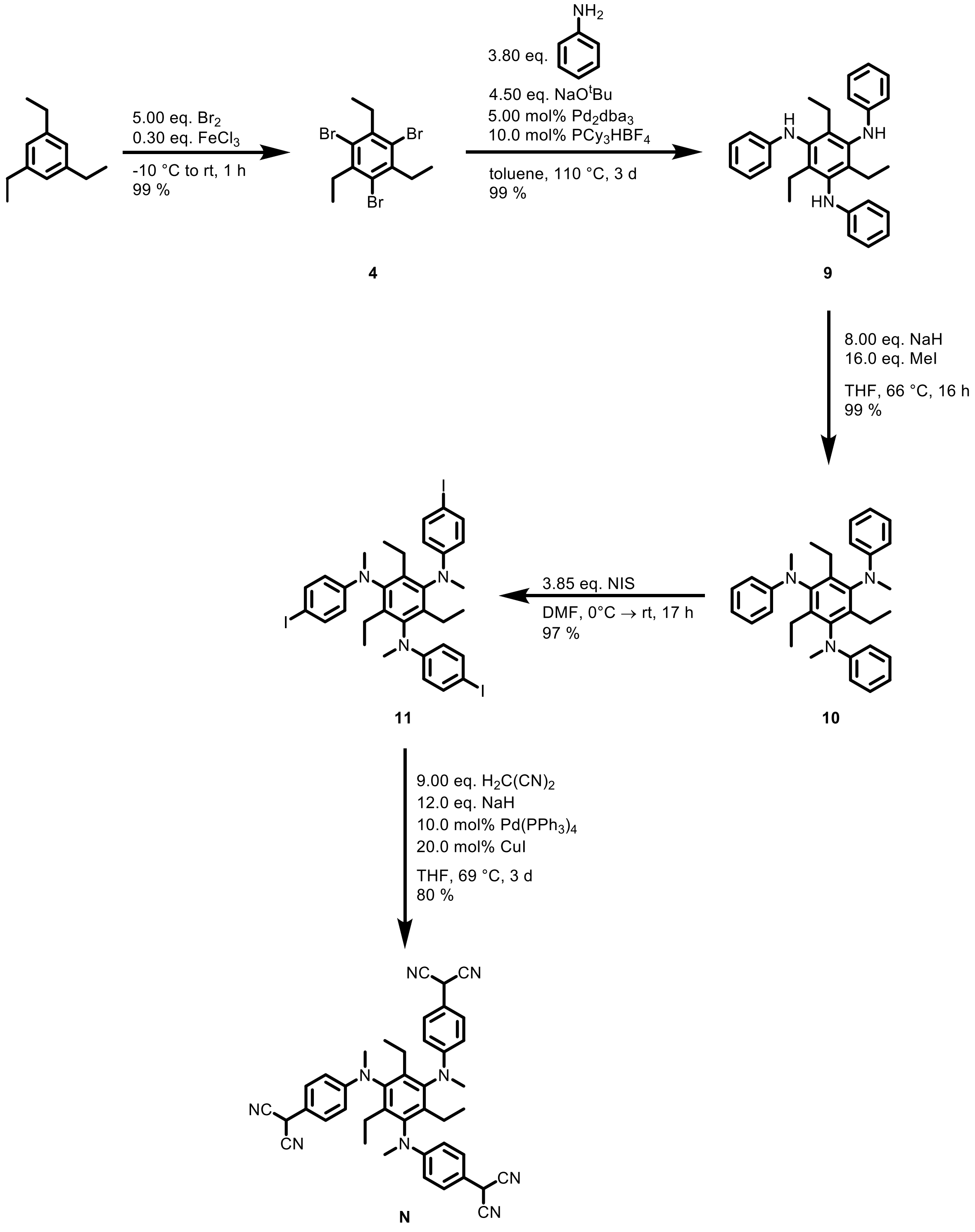

Scheme S4: Synthetic overview for **N**.

2.6.1 Synthesis of 2,4,6-triethyl-*N*[1],*N*[3],*N*[5]-triphenylbenzene-1,3,5-triamine 9

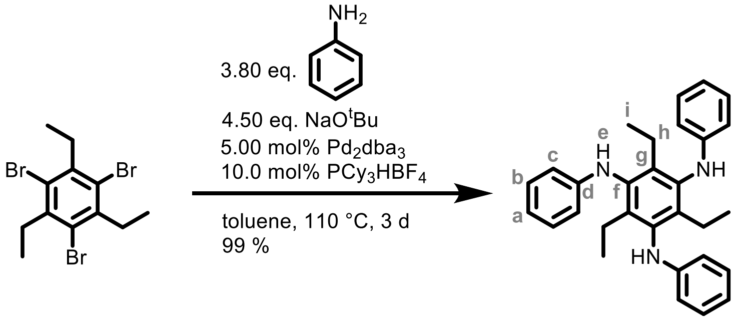

4 **9**

In a 50 mL Schlenk round-bottom flask were added **4** (1.40 g, 3.50 mmol, 1.00 eq.), NaO^tBu (1.51 g, 15.8 mmol, 4.50 eq.), Pd_2dba_3 (0.160 g, 0.180 mmol, 5.00 mol%), PCy_3HBF_4 (0.129 g, 0.350 mmol, 10.0 mol%) and dissolved in 21 mL dry toluene. Aniline (1.21 mL, 13.3 mmol, 3.80 eq.) was added, and the mixture was stirred at 110 °C for 3 days. 30 mL water were added, and the mixture was extracted with 3 x 30 mL ethyl acetate. The crude product was purified by column chromatography with *n*-hexane/ethyl acetate 9/1 as eluent to yield **9** (1.52 g, 3.48 mmol, 99 %) as a light pink crystalline solid.

^{1}H NMR (600 MHz, $CDCl_3$): δ = 7.20–7.15 (m, 6 H, **H$_b$**), 6.74 (t, ^3J = 7.2 Hz, 3 H, **H$_a$**), 6.53 (d, ^3J = 7.8 Hz, 6 H, **H$_c$**), 5.22 (s, 3 H, **H$_e$**), 2.56 (q, ^3J = 7.5 Hz, 6 H, **H$_h$**), 1.01 (t, ^3J = 7.5 Hz, 9 H, **H$_i$**).

^{13}C{^{1}H} NMR (151 MHz, $CDCl_3$): δ = 147.67 (**C$_d$**), 141.21 (**C$_e$**), 137.07 (**C$_b$**), 129.38 (**C$_f$**), 118.11 (**C$_a$**), 113.22 (**C$_c$**), 21.58 (**C$_g$**), 15.10 (**C$_h$**).

HRMS (ESI): m/z calculated for $[C_{30}H_{30}O_3+H]^+$: 439.2268, found: 439.2264.

IR $\tilde{\nu}$ [cm^{-1}]: 3078.39 (w), 2970.38 (w), 2937.59 (w), 2877.79 (w), 2422.59 (w), 1581.63 (w), 1489.05 (m), 1456.26 (w), 1427.32 (m), 1371.39 (w), 1330.88 (w), 1317.38 (w), 1288.45 (w), 1257.59 (w), 1209.37 (s), 1163.08 (m), 1099.43 (s), 1074.35 (w), 1049.28 (w), 1022.27 (w), 997.20 (w), 974.05 (w), 867.97 (m), 840.96 (w), 823.60 (w), 785.03 (w), 746.45 (s), 718.45 (w), 688.59 (s), 659.66 (w), 615.29 (w).

2.6.2 Synthesis of 2,4,6-triethyl-*N*[1],*N*[3],*N*[5]-trimethyl-*N*[1],*N*[3],*N*[5]-triphenylbenzene-1,3,5-triamine 10

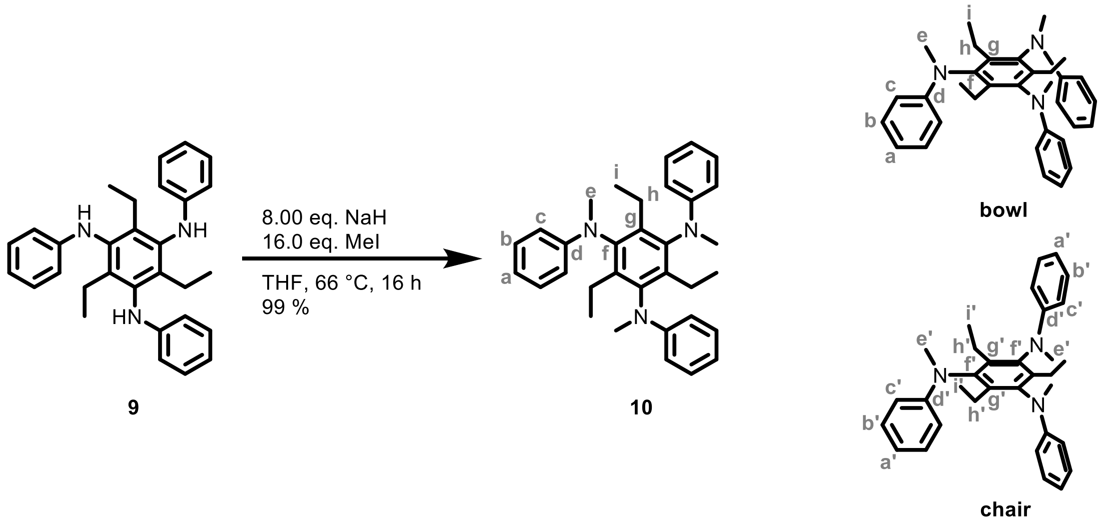

In a 100 mL Schlenk round-bottom flask were added NaH (1.12 g, 28.0 mmol, 8.00 eq.; 60 % in mineral oil) and 25 mL dry THF under nitrogen. Subsequently, **9** (1.53 g, 3.50 mmol, 1.00 eq.) in 20 mL dry THF was added dropwise at room temperature and further stirred at 66 °C for 2 hours until MeI (3.49 mL, 56.0 mmol, 16.0 eq.) was added at room temperature. The reaction vessel was then sealed and stirred at 66 °C for additional 14 hours. The reaction was quenched by the addition of 10 mL water, the reaction mixture was extracted with 3 x 30 mL ethyl acetate, and the organic phase was dried with Na_2SO_4 to yield **10** (1.66 g, 3.47 mmol, 99 %) as a pink solid after removal of the solvent under reduced pressure.

^{1}H NMR (600 MHz, $CDCl_3$): δ = 7.45–6.99 (m, 6 H, **H_b/$H_{b'}$**), 6.89–6.61 (m, 6 H, **H_a/$H_{a'}$+H_c**), 6.11 (br s, **$H_{c'}$**), 3.33–3.27 (m, 9 H, **H_e/$H_{e'}$**), 2.40–2.22 (m, 6 H, **H_h/$H_{h'}$**), 1.02–0.94 (m, 4.5 H, **$H_{i'}$**), 0.88–0.83 (m, 4.5 H, **H_i**).

^{13}C{^{1}H} NMR (151 MHz, $CDCl_3$): δ = 148.97 (**$C_{d'}$(chair)**), 148.79 (**C_d(bowl)**), 148.61 (**$C_{d'}$(chair)**), 145.33 (**$C_{f'}$(chair)**), 145.02 (**C_f(bowl)**), 145.00 (**$C_{f'}$(chair)**), 144.73 (**2 x $C_{g'}$(chair)**, 144.25 (**C_g(bowl)**), 129.20 (**br**, **C_b(bowl)/$C_{b'}$(chair)**), 116.28 (**$C_{a'}$(chair)**), 116.21 (**C_a(bowl)**), 116.16 (**$C_{a'}$(chair)**), 113.07 (**br**, **C_c(bowl)**), 109.57 (**br**, **$C_{c'}$(chair)**), 40.34 (**$C_{e'}$(chair)**), 40.23 (**C_e(bowl)**), 40.22 (**$C_{e'}$(chair)**), 21.70 (**C_h(bowl)**), 21.65 (**$C_{h'}$(chair)**), 21.54 (**$C_{h'}$(chair)**), 15.29 (**$C_{i'}$(chair)**), 15.04 (**$C_{i'}$(chair)**), 14.79 (**C_i(bowl)**).

HRMS (ESI): m/z calculated for $[C_{33}H_{39}N_3+H]^+$: 478.3217, found: 478.3212.

IR $\tilde{\nu}$ [cm^{-1}]: 3086.11 (w), 3049.46 (w), 3024.38 (w), 2954.95 (w), 2924.09 (w), 2872.01 (w), 2852.72 (w), 2812.21 (w), 2762.06 (w), 2725.42 (w), 2630.91 (w), 1907.60 (w), 1666.50 (w), 1597.06 (m), 1571.99 (w), 1558.48 (w), 1496.76 (s), 1477.47 (w), 1462.04 (w), 1435.04 (w),

1417.68 (w), 1369.46 (w), 1328.95 (w), 1317.38 (w), 1300.02 (w), 1288.45 (w), 1263.37 (w), 1215.15 (w), 1186.22 (w), 1157.29 (w), 1138.00 (w), 1112.93 (m), 1095.57 (w), 1060.85 (w), 1029.99 (w), 987.55 (w), 968.27 (w), 947.05 (w), 912.33 (w), 862.18 (w), 810.10 (w), 785.03 (w), 744.52 (s), 715.59 (w), 688.59 (s), 617.22 (w).

Due to the rotational barriers introduced by *N*-methylation, multiple rotamers are present in solution, giving rise to a large number of signals and partial signal broadening in the NMR spectra. The most prominent isomers corresponding to the two most stable conformers were assigned. The conformers are referred to as **bowl** (all *N*-phenyl substituents pointing downwards) and **chair** (one *N*-phenyl substituent pointing upwards) and the corresponding signals are marked as **x** and **x'** respectively.[2]

2.6.3 Synthesis of 2,4,6-triethyl-*N*1,*N*3,*N*5-tris(4-iodophenyl)-*N*1,*N*3,*N*5-trimethylbenzene-1,3,5-triamine 11

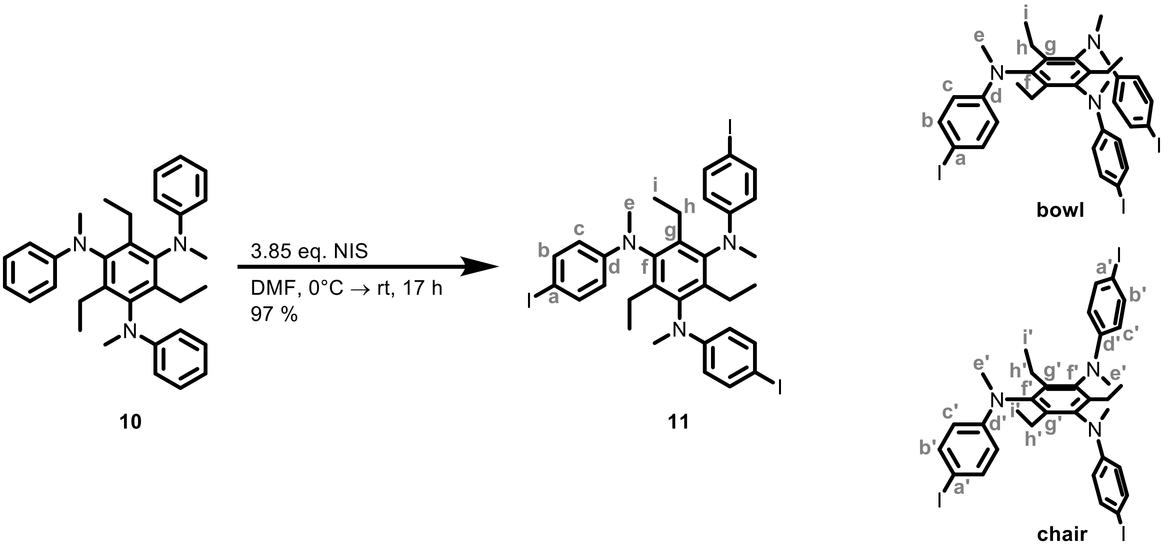

In a 100 mL Schlenk round-bottom flask were added **10** (1.62 g, 3.40 mmol, 1.00 eq) in 60 mL dry DMF under nitrogen and stirred at 0 °C. Subsequently NIS (2.94 g, 13.1 mmol, 3.85 eq.) was added in three portions after 0, 15 and 30 minutes. Afterwards the mixture was slowly warmed to room temperature and stirred for an additional 17 hours. The reaction was then quenched by the addition of 60 mL aqueous saturated $Na_2S_2O_3$ solution. The precipitate was filtered and washed with 100 mL water to yield **11** (2.83 g, 3.31 mmol, 97 %) as a brown solid.

^{1}H NMR (600 MHz, $CDCl_3$): δ = 7.67–7.27 (m, 6 H, **H$_b$/H$_{b'}$**), 6.55 (br s, **H$_c$**), 5.79 (br s, **H$_{c'}$**), 3.27 –3.22 (m, 9 H, **H$_e$/H$_{e'}$**), 2.33–2.16 (m, 6 H, **H$_h$/H$_{h'}$**), 0.98–0.90 (m, 4.5 H, **H$_{i'}$**), 0.84 (t, ^3J = 7.5 Hz, 4.5 H, **H$_i$**).

$^{13}C\{^1H\}$ NMR (151 MHz, $CDCl_3$): δ = 148.43 (**$C_{d'}$(chair)**), 148.25 (**C_d(bowl)**), 148.09 (**$C_{d'}$(chair)**), 144.99 (**$C_{f'}$(chair)**), 144.68 (**C_f(bowl)**), 144.64 (**$C_{f'}$(chair)**), 144.62 (**$C_{g'}$(chair)**, 144.55 (**$C_{g'}$(bowl)**), 144.18 (**C_g(bowl)**), 138.14 (**br, C_b(bowl)/$C_{b'}$(chair)**), 115.31 (**br, $C_{c'}$(chair)**), 112.15 (**br, C_c(bowl)**), 77.67 (**$C_{a'}$(chair)**), 77.56 (**C_a(bowl)**), 77.47 (**$C_{a'}$(chair)**), 40.28 (**$C_{e'}$(chair)**), 40.26 (**$C_{e'}$(bowl)**), 40.23 (**C_e(chair)**), 21.64 (**C_h(bowl)**), 21.58 (**$C_{h'}$(chair)**), 21.48 (**$C_{h'}$(chair)**), 15.18 (**$C_{i'}$(chair)**), 14.98 (**$C_{i'}$(chair)**), 14.81 (**C_i(bowl)**).

HRMS (ESI): m/z calculated for $[C_{33}H_{36}I_3N_3+H]^+$: 856.0117, found: 856.0098.

IR $\tilde{\nu}$ [cm^{-1}]: 3076.46 (w), 2968.45 (w), 2931.80 (w), 2897.08 (w), 2873.94 (w), 2812.21 (w), 2357.01 (w), 2333.87 (w), 2050.33 (w), 1863.24 (w), 1585.49 (m), 1554.63 (w), 1485.19 (s), 1462.04 (w), 1431.18 (w), 1402.25 (w), 1367.53 (w), 1330.88 (m), 1311.59 (w), 1290.38 (w), 1278.81 (w), 1261.45 (w), 1211.30 (w), 1186.22 (w), 1143.79 (w), 1111.00 (m), 1058.92 (w), 1031.92 (w), 991.41 (w), 954.76 (w), 923.90 (w), 866.04 (w), 804.32 (m), 783.10 (w), 731.02 (w), 692.44 (w), 663.51 (w), 628.79 (w).

2.6.4 Synthesis of 2,2',2''-(((2,4,6-triethylbenzene-1,3,5-triyl)tris(methylazanediyl))tris(benzene-4,1-diyl))trimalononitrile N

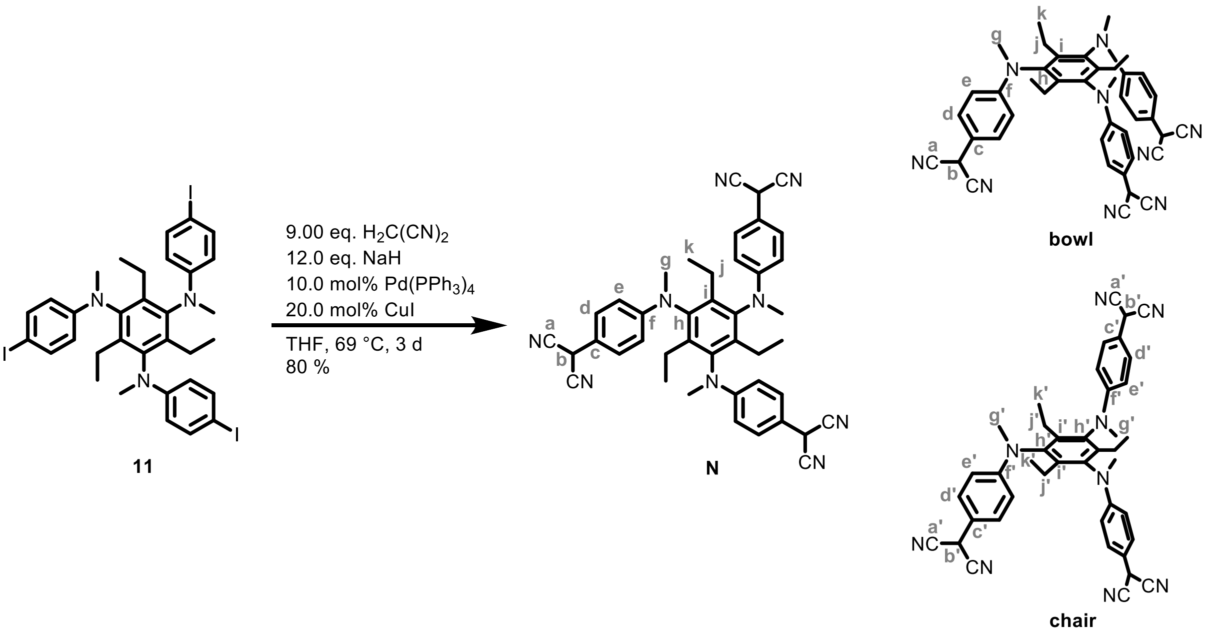

In a 10 mL Schlenk tube were added NaH (0.072 g, 1.80 mmol, 12.0 eq.; 60 % in mineral oil) and 1.0 mL dry THF under nitrogen. The mixture was then cooled to 0 °C and malononitrile (0.089 g, 1.35 mmol, 9.00 eq.) in 0.5 mL dry THF was slowly added under vigorous stirring. After the addition, the mixture was stirred for another 30 minutes at room temperature. Finally, **11** (0.128 g, 0.150 mmol, 1.00 eq.), $Pd(PPh_3)_4$ (0.017 g, 0.015 mmol, 10.0 mol%) and CuI

(0.006 g, 0.030 mmol, 20.0 mol%) were added, and the mixture was stirred at 69 °C for 3 days, after which the reaction was quenched by the addition of 0.8 mL 3M HCl at 0 °C. The mixture was extracted with 3 x 10 mL of dichloromethane, washed with 10 mL of water, and the organic phase was dried with $MgSO_4$. After removal of the solvent under reduced pressure, the mixture was suspended in 2 mL methanol, filtered, and subsequently 10 mL water were added to the filtrate to precipitate **N** (0.080 g, 0.119 mmol, 80 %) as a green solid.

^{1}H NMR (600 MHz, $CDCl_3$): δ = 7.40 (br s, 3 H, **H_d**), 7.17 (br s, 3 H, **$H_{d'}$**), 6.84 (br s, 3 H, **H_e**), 6.08 (br s, 3 H, **$H_{e'}$**), 4.95 (s, 3 H, **H_b/$H_{b'}$**), 3.37–3.29 (m, 9 H, **H_g/$H_{g'}$**), 2.40–2.18 (m, 6 H, **H_j/$H_{j'}$**), 1.01-0.93 (m, 4.5 H, **$H_{k'}$**), 0.86 (t, ^3J = 7.5 Hz, 4.5 H, **H_k**).

^{13}C{^{1}H} NMR (151 MHz, $CDCl_3$): δ = 149.99 (**$C_{f'}$(chair)**), 149.84 (**C_f(bowl)**), 149.68 (**$C_{f'}$(chair)**), 144.91 (**$C_{h'}$(chair)**), 144.61 (**C_h(bowl)**), 144.60 (**$C_{h'}$(chair)**), 144.54 (**$C_{i'}$(chair**), 144.53 (**$C_{i'}$(bowl)**), 144.25 (**C_i(bowl)**), 128.91 (**br, C_d(bowl)**),128.34 (**br, $C_{d'}$(chair)**), 128.11 (**br, $C_{d'}$(chair)**), 127.89 (**br, $C_{d'}$(chair)**), 114.19 (**br, $C_{e'}$(chair)**), 113.77 (**br, $C_{e'}$(chair)**), 113.40 (**br, $C_{e'}$(chair)**), 113.36 (**$C_{c'}$(chair)**), 113.26 (**C_c(bowl)**),113.16 (**$C_{c'}$(chair)**), 112.48 **C_a(bowl)**), 112.44 (**$C_{a'}$(chair)**), 112.43 (**$C_{a'}$(chair)**), 110.74 (**br**, **C_e(bowl)**), 40.50 (**$C_{g'}$(chair)**), 40.48 (**$C_{g'}$(bowl)**), 40.47 (**C_g(chair)**), 27.61 (**$C_{b'}$(chair)**), 27.58 (**C_b(bowl)**), 27.57 (**$C_{b'}$(chair)**), 21.68 (**C_j(bowl)**), 21.62 (**$C_{j'}$(chair)**), 21.54 (**$C_{j'}$(chair)**), 15.09 (**$C_{k'}$(chair)**), 14.92 (**$C_{k'}$(chair)**), 14.79 (**C_k(bowl)**).

HRMS (ESI): m/z calculated for $[C_{42}H_{39}N_9+NH_4]^+$: 687.3667, found: 687.3667.

IR $\tilde{\nu}$ [cm^{-1}]: 2972.31 (w), 2937.59 (w), 2900.94 (w), 2885.51 (w), 1649.21 (w), 1589.34 (s), 1546.91 (w), 1514.12 (s), 1475.54 (w), 1433.11 (w), 1417.68 (w), 1350.17 (w), 1332.81 (w), 1274.95 (m), 1230.58 (w), 1209.37 (w), 1180.44 (m), 1153.43 (w), 1111.00 (w), 1076.28 (w), 1056.99 (w), 1028.06 (w), 1004.91 (w), 968.27 (w), 954.76 (w), 914.26 (w), 867.97 (w), 831.32 (w), 777.31 (w), 752.24 (w), 723.31 (w), 692.44 (w).

2.7 Synthesis of N^4

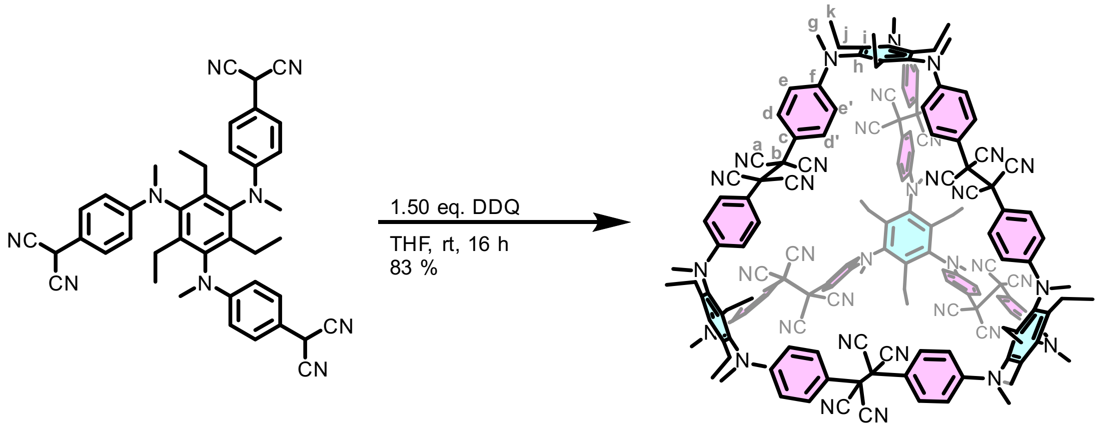

N **N^4**

In a 100 mL Schlenk round-bottom flask was **N** (201 mg, 0.300 mmol, 1.00 eq.) dissolved in 40 mL dry THF. Then DDQ (102 mg, 0.450 mmol, 1.50 eq.) in 10 mL dry THF was slowly added, and the mixture was stirred for another 16 hours at room temperature. The THF was removed under reduced pressure to about 5 mL. Afterwards methanol was added to precipitate the product, which was collected by filtration. Another treatment of the filtrate with water (CAUTION: reacts with residual DDQ by formation of HCN!) precipitated residual product. That way **N^4** (166 mg, 0.0622 mmol, 83 %) was obtained in the form of a blue solid.

Due to the instability of the σ-bond formed by the recombination of the aryldicyanomethyl radicals, mass spectrometry of the compounds only yielded the corresponding monomers after ionisation in our hands.

^{1}H NMR (600 MHz, $CDCl_3$): δ = 7.82 (dd, ^3J = 8.9 Hz, ^4J = 2.8 Hz, 12 H, $\mathbf{H_d}$), 7.65 (dd, ^3J = 9.0 Hz, ^4J = 2.8 Hz, 12 H, $\mathbf{H_{d'}}$), 7.08 (dd, ^3J = 9.1 Hz, ^4J = 2.9 Hz, 12 H, $\mathbf{H_e}$), 6.57 (dd, ^3J = 9.0 Hz, ^4J = 2.9 Hz, 12 H, $\mathbf{H_{e'}}$), 3.41 (s, 36 H, $\mathbf{H_g}$), 2.40 (q, ^3J = 7.6 Hz, 24 H, $\mathbf{H_j}$), 1.00 (t, ^3J = 7.6 Hz, 36 H, $\mathbf{H_k}$).

^{13}C{^{1}H} NMR (151 MHz, $CDCl_3$): δ = 152.12 ($\mathbf{C_f}$), 145.67 ($\mathbf{C_h}$), 145.27 ($\mathbf{C_i}$), 130.74 ($\mathbf{C_d}$), 130.31 ($\mathbf{C_{d'}}$), 114.60 ($\mathbf{C_{e'}}$), 114.44 ($\mathbf{C_c}$), 112.02 ($\mathbf{C_a}$), 111.26 ($\mathbf{C_e}$), 55.82 ($\mathbf{C_b}$), 41.31 ($\mathbf{C_g}$), 22.48 ($\mathbf{C_j}$), 15.02 ($\mathbf{C_k}$).

IR $\tilde{\nu}$ [cm^{-1}]: 2937.59 (w), 2877.79 (w), 2856.58 (w), 2823.79 (w), 2191.13 (w), 1606.70 (s), 1556.55 (w), 1514.12 (s), 1477.47 (w), 1435.04 (w), 1417.68 (w), 1373.32 (w), 1346.31 (w), 1330.88 (w), 1307.74 (w), 1265.30 (w), 1190.08 (m), 1153.43 (w), 1141.86 (w), 1112.93 (m), 1085.92 (w), 1060.85 (w), 1037.70 (w), 1004.91 (w), 979.84 (w), 950.91 (w), 935.48 (w), 867.97 (w), 808.17 (m), 785.03 (w), 723.31 (w).

3 ^{1}H DOSY experiments

^{1}H DOSY experiments were recorded at 298 K and calibrated using known values for the solvents used (D_{solv}). The hydrodynamic radii were estimated using the unmodified Stokes-Einstein equation. The equation was solved for r_H, using values for η from the literature.[4] The viscosity of neat THF was used.[5]

$$D = \frac{k_B T}{6\pi\eta r_H} \quad \Rightarrow \quad r_H = \frac{k_B T}{6\pi\eta D}$$

D is the measured diffusion coefficient [$m^2\,s^{-1}$]

k_B is the *Boltzmann* constant ($1.38 \cdot 10^{-23}$ m^2 kg s^{-2} K^{-1})

T is the temperature [K]

r_H is the hydrodynamic radius of the analyte [m]

η is the viscosity of the solvent at temperature T [kg m^{-1} s^{-1}]

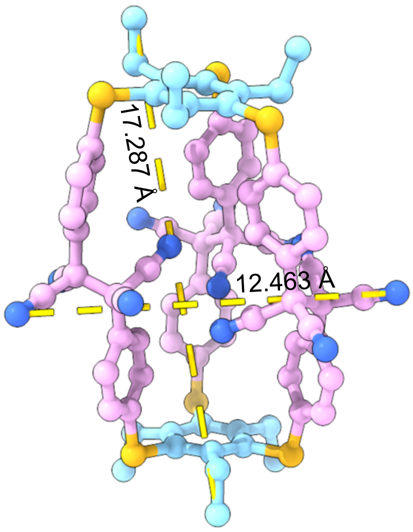

Figure S1: Distances between atoms of **S²** (structure optimised by force field) marked in yellow and corresponding distances noted in black.

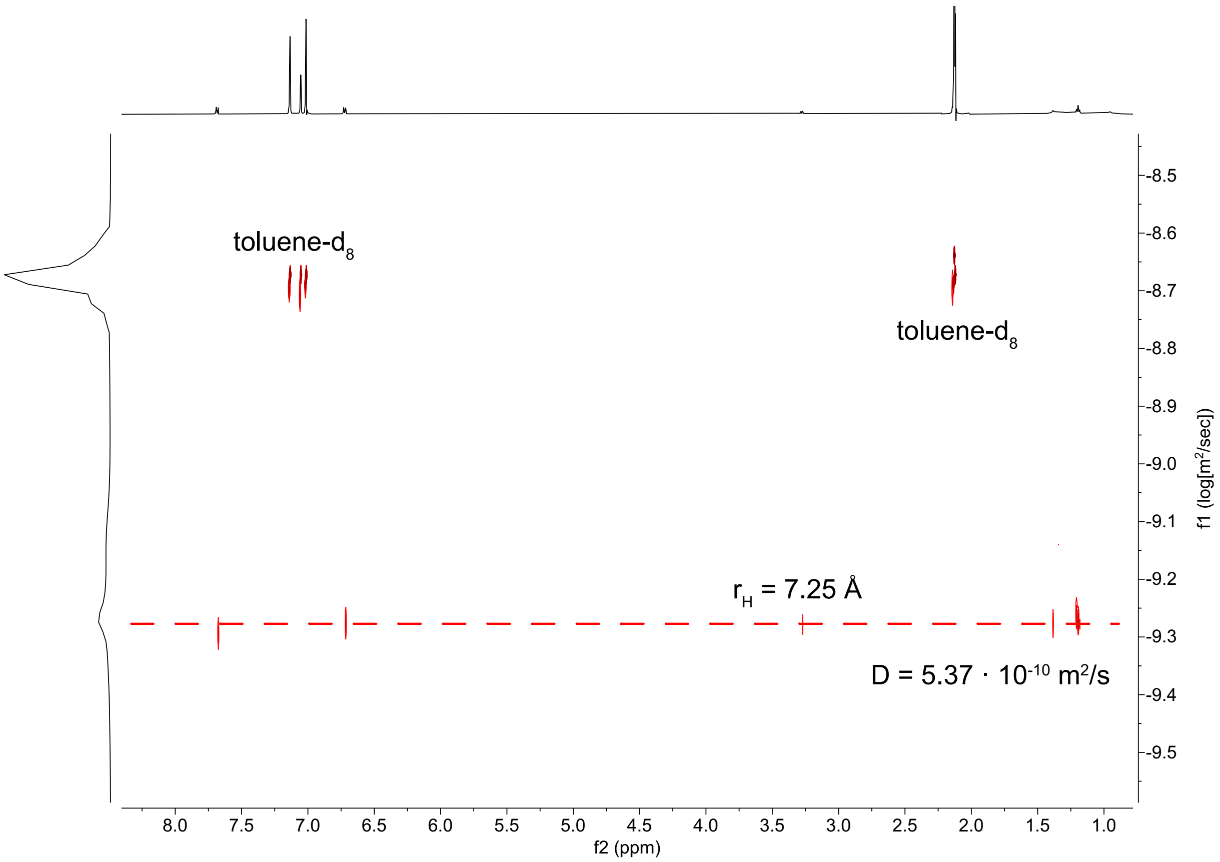

Figure S2: ^{1}H DOSY NMR spectrum (600 MHz, toluene-d$_8$, 298 K) of **S^2**.

The hydrodynamic diameter of **S^2** determined by ^{1}H DOSY NMR (14.5 Å) is in good agreement with the dimensions extracted from a force-field-optimised cage structure.

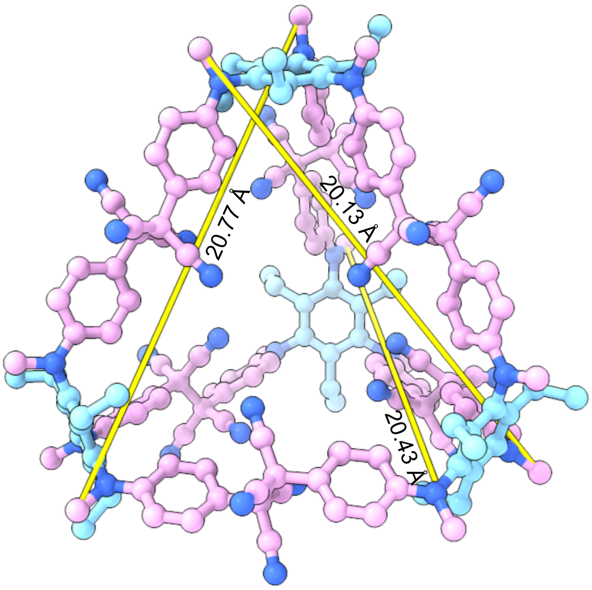

Figure S3: Distances between atoms of **N^4** (structure obtained by SC-XRD) marked in yellow and corresponding distances noted in black.

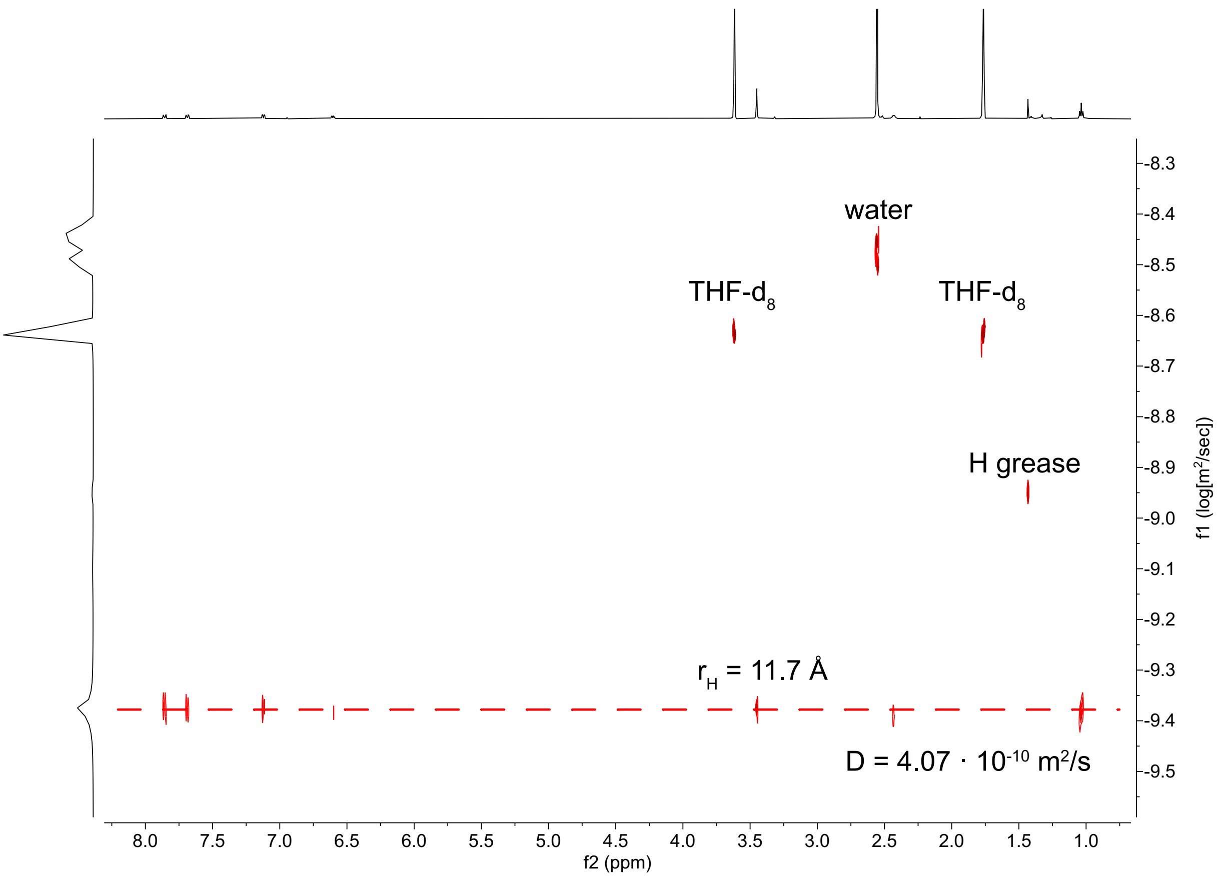

Figure S4: ^{1}H DOSY NMR spectrum (600 MHz, THF-d8, 298 K) of **N⁴**.

The hydrodynamic diameter of **N⁴** determined by ^{1}H DOSY NMR (23.4 Å) is in good agreement with the dimensions extracted from the crystal structure.

4 Crystallographic details

The analysis of **N⁴** was performed on a diffractometer at beamline PF BL-5A of KEK (the High Energy Accelerator Research Organization, Japan) with a Pilatus3 S6M detector (synchrotron radiation, λ = 0.7500 Å, T = 95 K). The crystal was kept at 95.00 K during data collection. Using Olex2,[6] the structure was solved with the SHELXT structure solution program[7] using intrinsic phasing and refined with the SHELXL refinement package[8] using least squares minimisation. Colourless crystals of **N⁴** were grown by slow evaporation of a THF solution in the fridge at 8 °C over the course of several days. Crystals of **N⁴** are heavily solvated, and seven THF molecules were successfully modelled with varying occupancies, then, heavily disordered solvent molecules were removed using the SQUEEZE procedure implemented in Platon software.[9] Framework disorder was observed and treated accordingly, geometric restraints were applied where appropriate to maintain chemically reasonable bond lengths and angles.
CCDC 2515476 (**N⁴**) contains the supplementary crystallographic data for this paper. Copies of the data can be obtained free of charge via http://www.ccdc.cam.ac.uk/conts/retrieving.html (or from the Cambridge Crystallographic Data Centre, 12, Union Road, Cambridge, CB2 1EZ, UK; Fax: +44 1223 336033; e-mail: deposit@ccdc.cam.ac.uk).

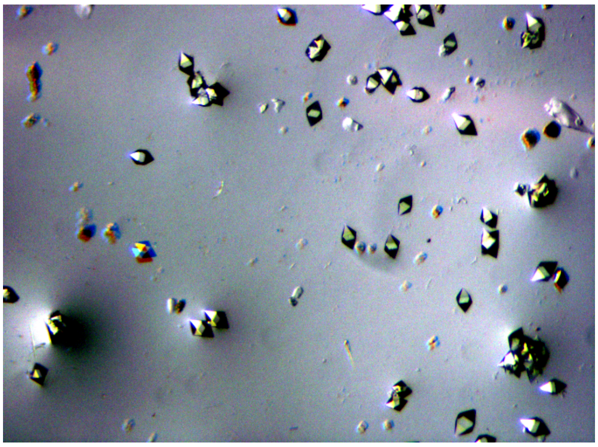

Figure S5: Colourless octahedral-like crystal of **N⁴** grown from THF.

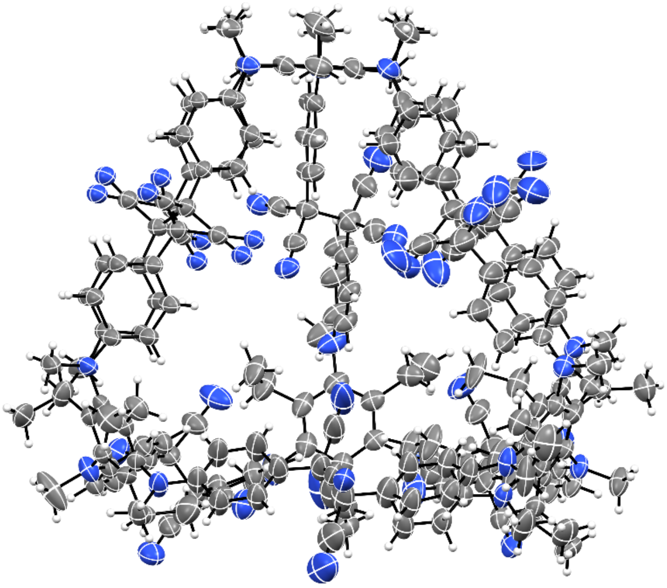

Figure S6: Data set of **N⁴** showing the asymmetric unit bearing one **N⁴** cage, THF solvent molecules are omitted for clarity, thermal ellipsoids set at 30 % probability. The structure was measured at 95 K and solved in the monoclinic space group $P2_1/c$ with Rint = 0.046, R1 = 0.138, and wR2 = 0.425.

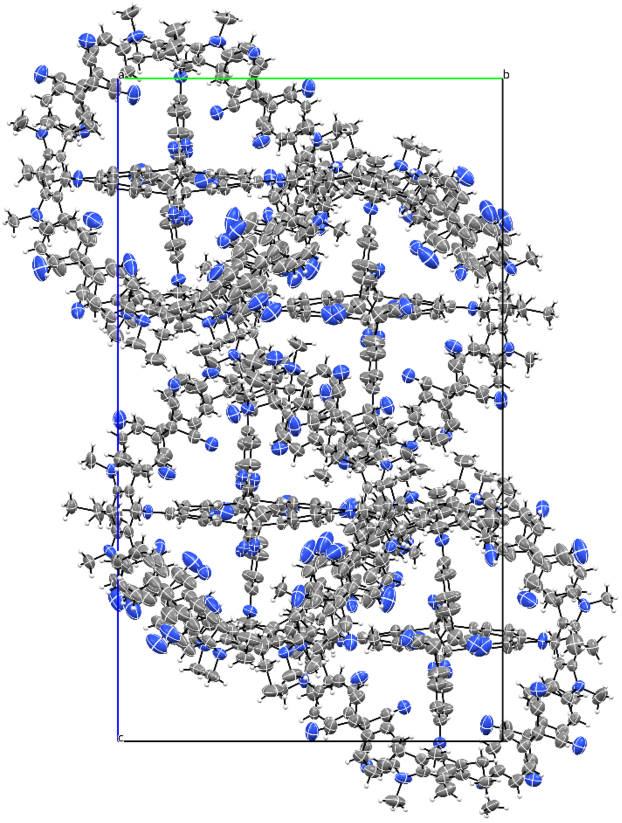

Figure S7: View of the unit cell of **N⁴** along the crystallographic *a* axis, THF solvent molecules omitted for clarity.

Table S2: Crystal data and structure refinement of N[4].

Identification code	2515476
Empirical formula	$C_{187.72}H_{183.62}N_{36}O_{4.93}$
Formula weight	3022.81
Temperature/K	95.00
Crystal system	monoclinic
Space group	$P2_1/c$
a/Å	22.173(6)
b/Å	22.947(4)
c/Å	40.895(8)
α/°	90
β/°	104.848(4)
γ/°	90
Volume/Å^3	20113(8)
Z	4
ρ_{calc}g/cm^3	0.998
μ/mm^{-1}	0.070
F(000)	6405.0
Crystal size/mm^3	0.075 × 0.075 × 0.05
Radiation	synchrotron (λ = 0.750)
2Θ range for data collection/°	2.006 to 50.084
Index ranges	$-21 \leqslant h \leqslant 22$, $-24 \leqslant k \leqslant 24$, $-43 \leqslant l \leqslant 44$
Reflections collected	84552
Independent reflections	27279 [R_{int} = 0.0463, R_{sigma} = 0.0510]
Data/restraints/parameters	27279/5580/2600
Goodness-of-fit on F^2	1.301
Final R indexes [I>=2σ (I)]	R_1 = 0.1380, wR_2 = 0.3873
Final R indexes [all data]	R_1 = 0.2018, wR_2 = 0.4245
Largest diff. peak/hole / e Å^{-3}	0.81/-0.31

5 PXRD measurements

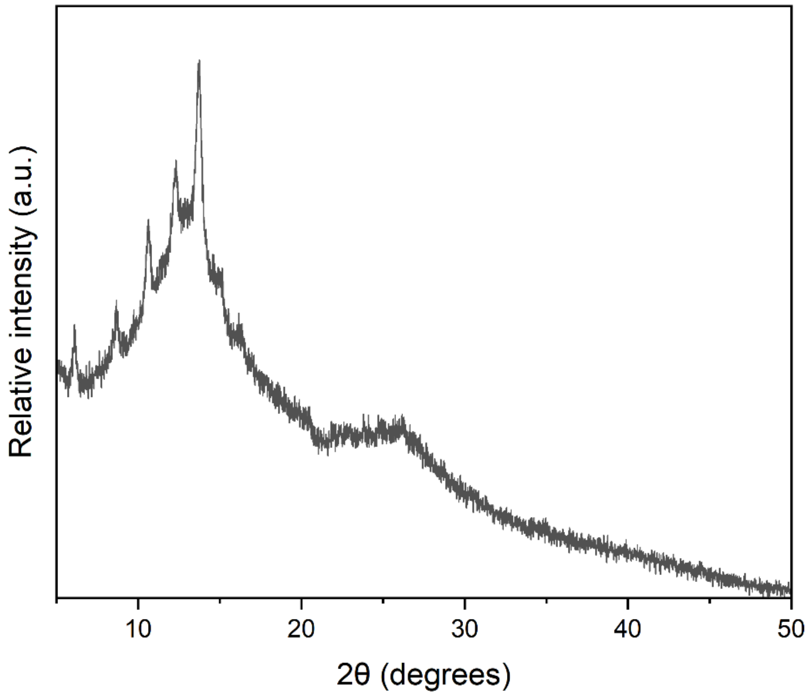

Figure S8: PXRD pattern of **N**^{4} before gas sorption measurement.

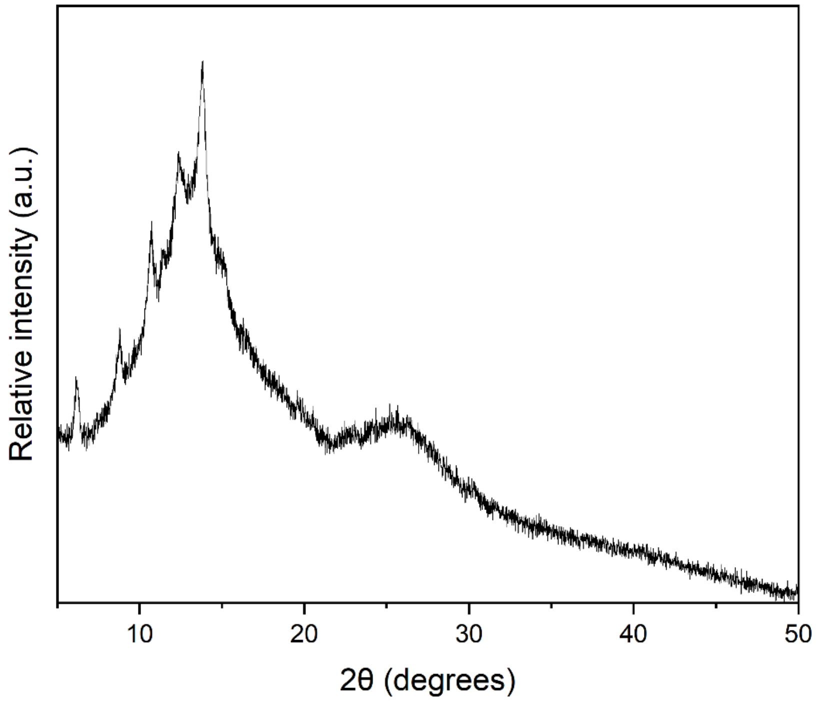

Figure S9: PXRD pattern of **N**^{4} after gas sorption measurement.

6 Gas adsorption measurements

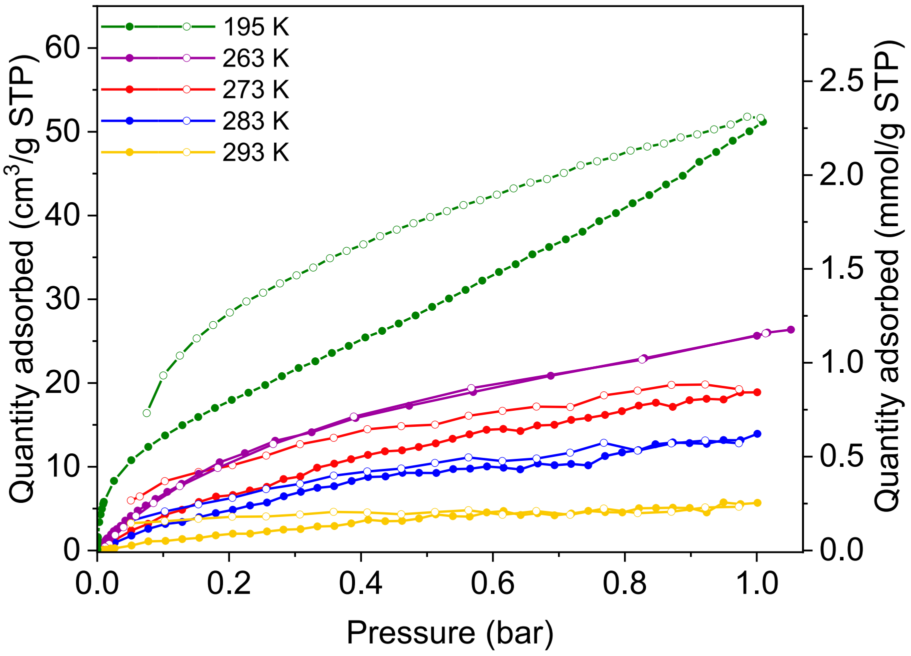

Figure S10: CO_2 adsorption (filled symbol) and desorption (hollow symbol) of **N**[4].

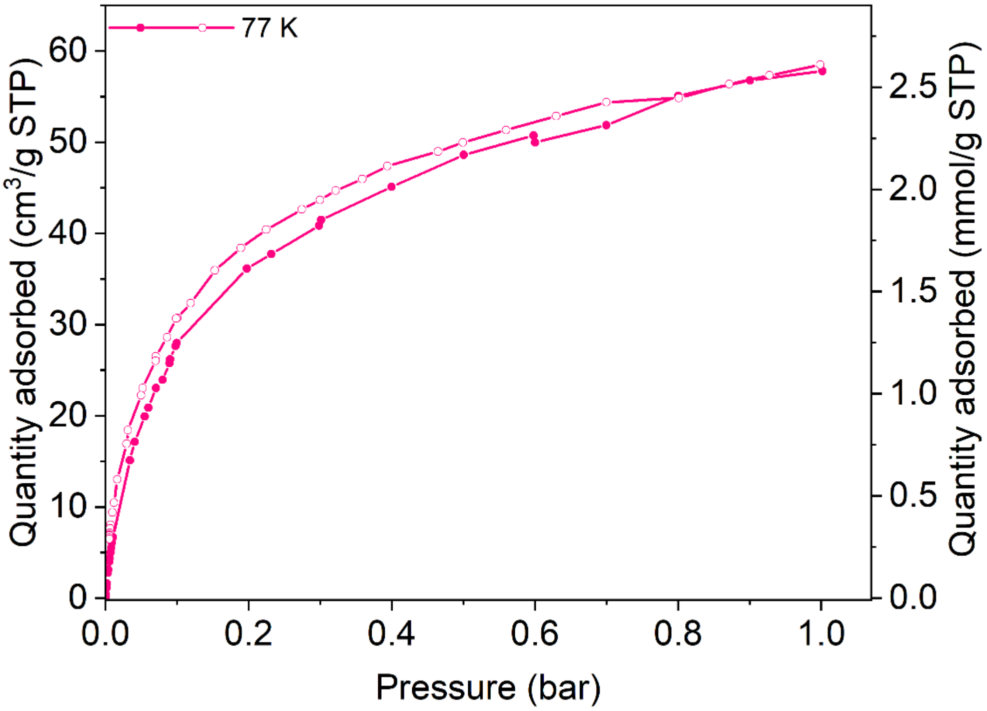

Figure S11: H_2 adsorption (filled symbol) and desorption (hollow symbol) of **N**[4].

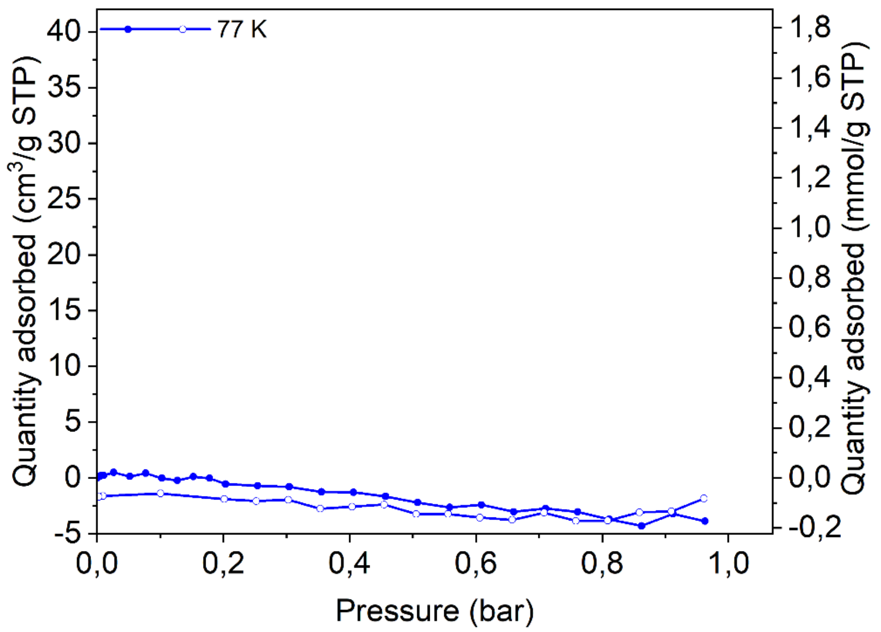

Figure S12: N_2 adsorption (filled symbol) and desorption (hollow symbol) of $\mathbf{N^4}$.

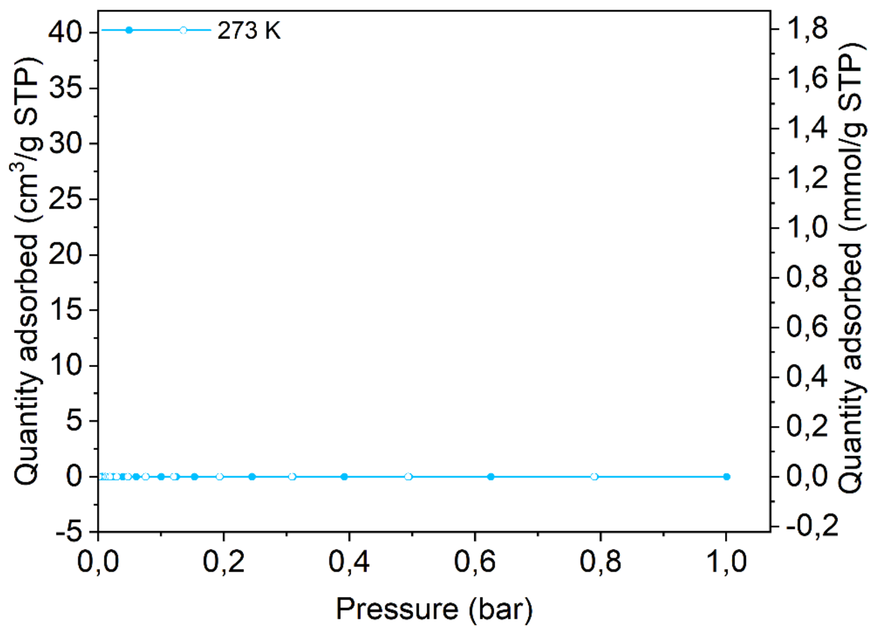

Figure S13: CH_4 adsorption (filled symbol) and desorption (hollow symbol) of $\mathbf{N^4}$.

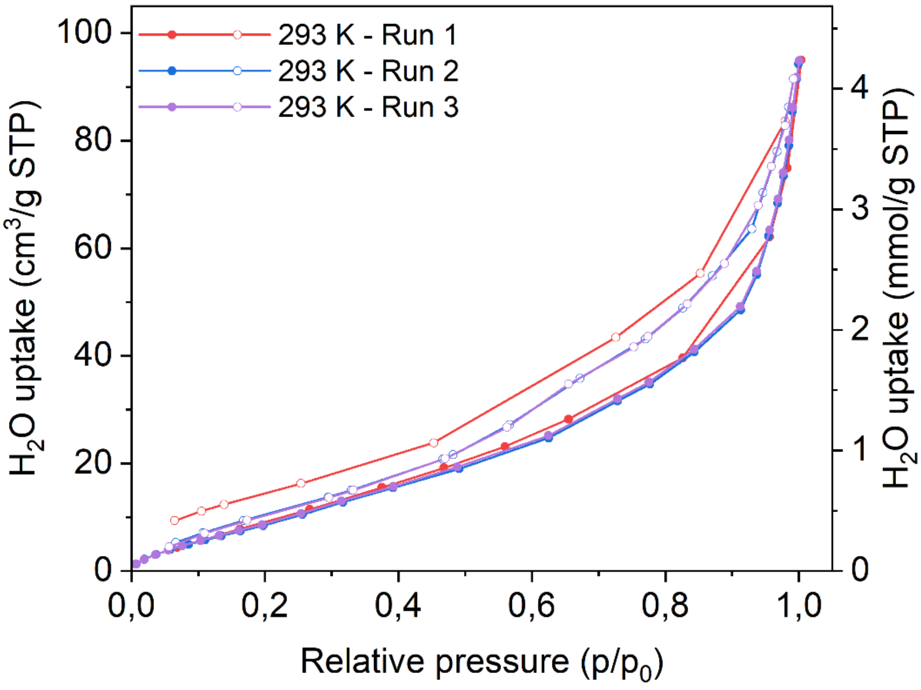

Figure S14: Repeated H_2O adsorption (filled symbol) and desorption (hollow symbol) of **N[4]**.

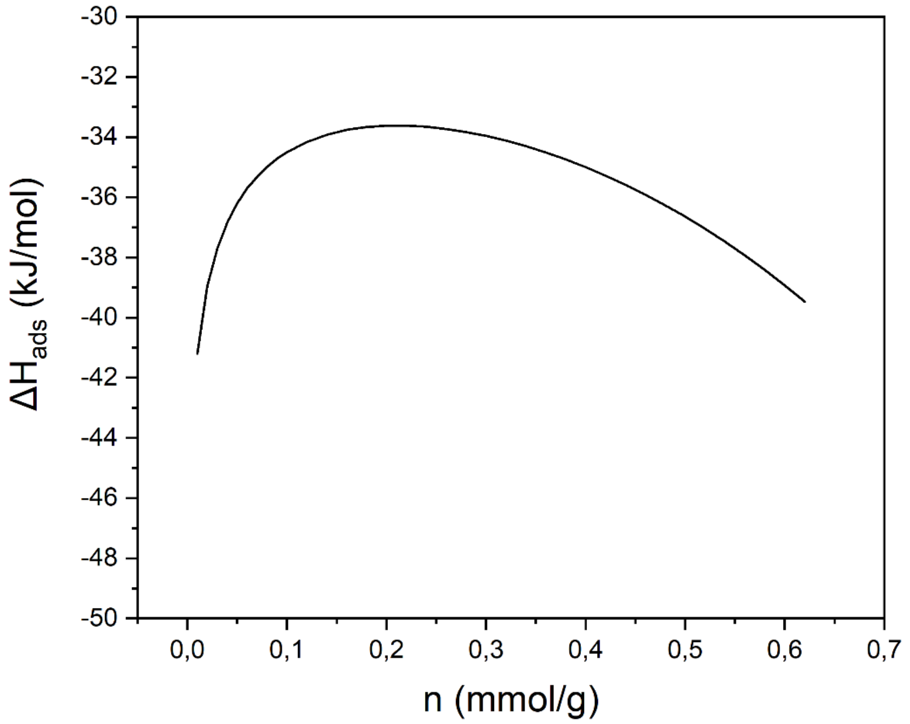

Figure S15: CO_2 adsorption enthalpy of **N[4]** determined from isotherms at 263, 273, and 283 K.

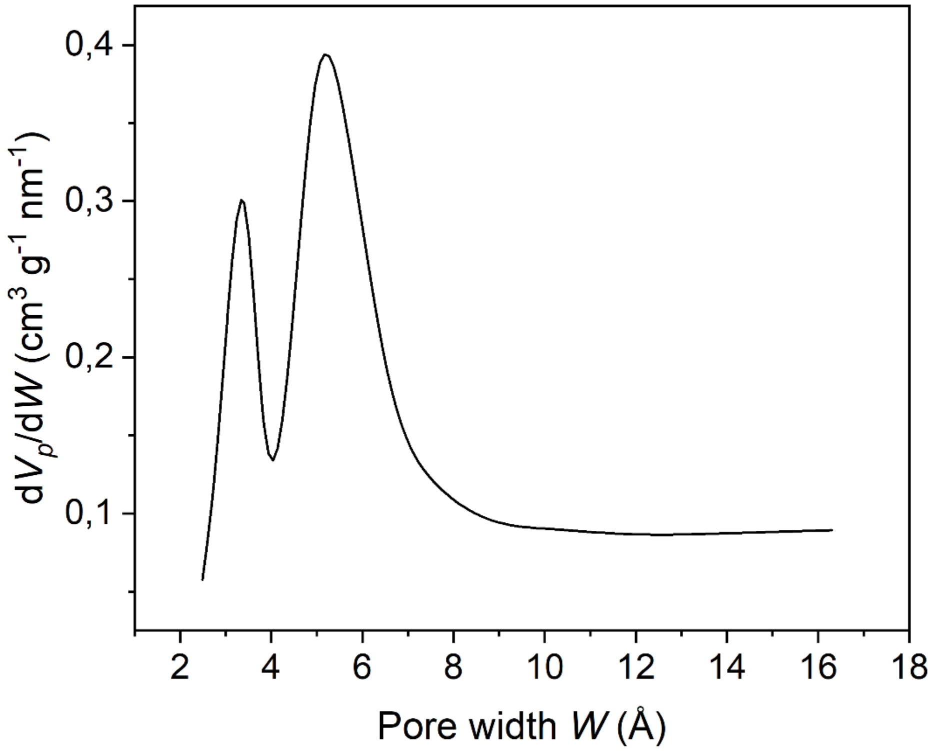

Figure S16: Grand canonical Monte Carlo (GCMC, slit pore shape) pore size distribution curves for **N[4]** obtained from the CO_2 adsorption isotherm at 273 K.

7 UV/Vis spectroscopy

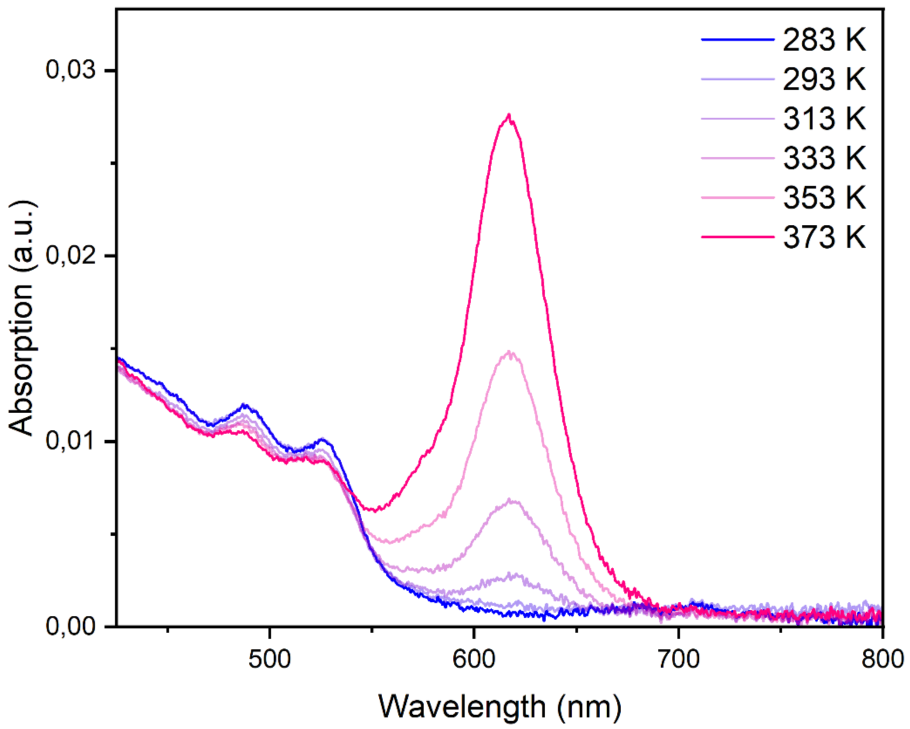

Figure S17: VT-UV/Vis absorption spectrum of **S²** in toluene (1·10⁻³ M).

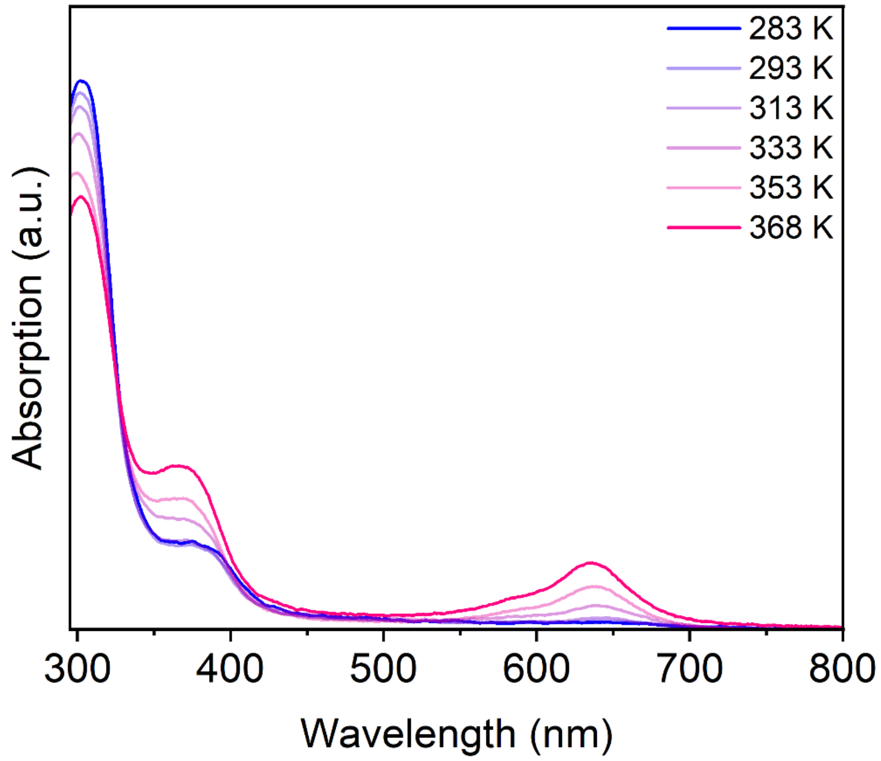

Figure S18: VT-UV/Vis absorption spectrum of **N⁴** in toluene (1·10⁻⁶ M).

8 EPR spectroscopy

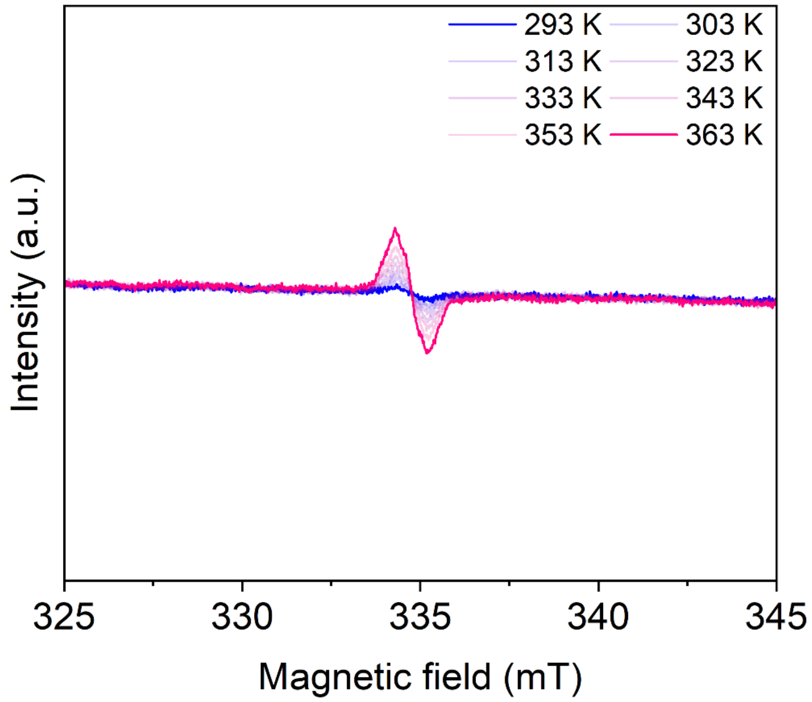

Figure S19: VT-EPR spectrum of **S²** in toluene ($1 \cdot 10^{-3}$ M; 90 s sweep time; 5 accumulations).

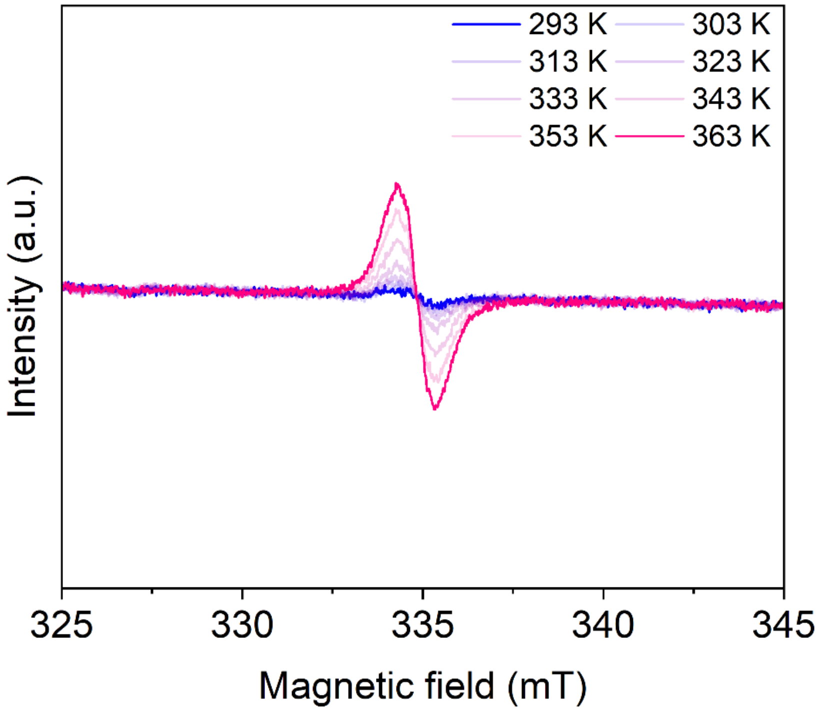

Figure S20: VT-EPR spectrum of **N⁴** in toluene ($1 \cdot 10^{-4}$ M; 90 s sweep time; 3 accumulations).

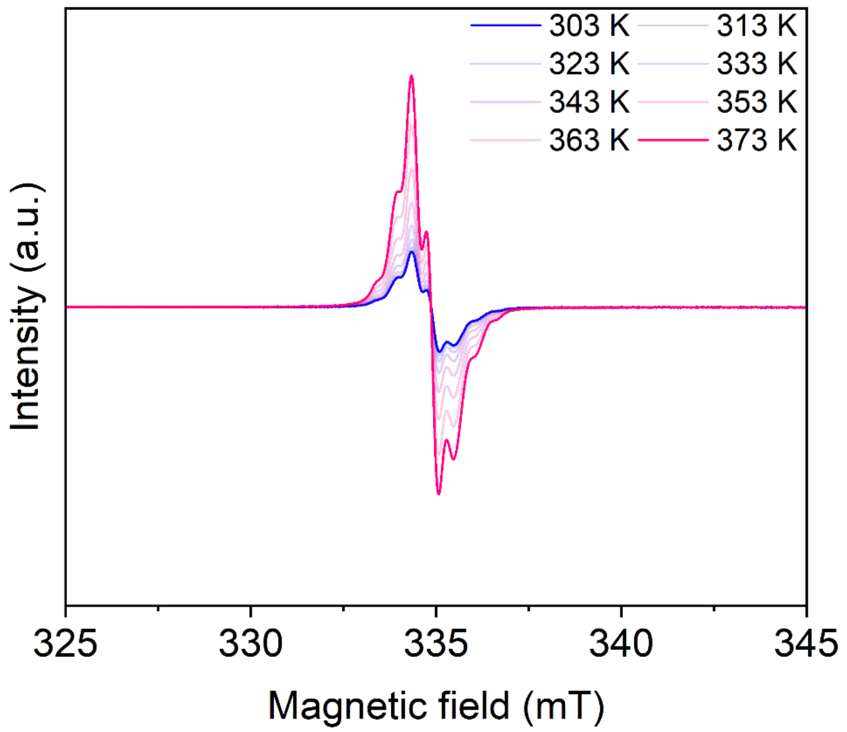

Figure S21: VT-EPR spectrum of **S²** in solid state (60 s sweep time; 3 accumulations).

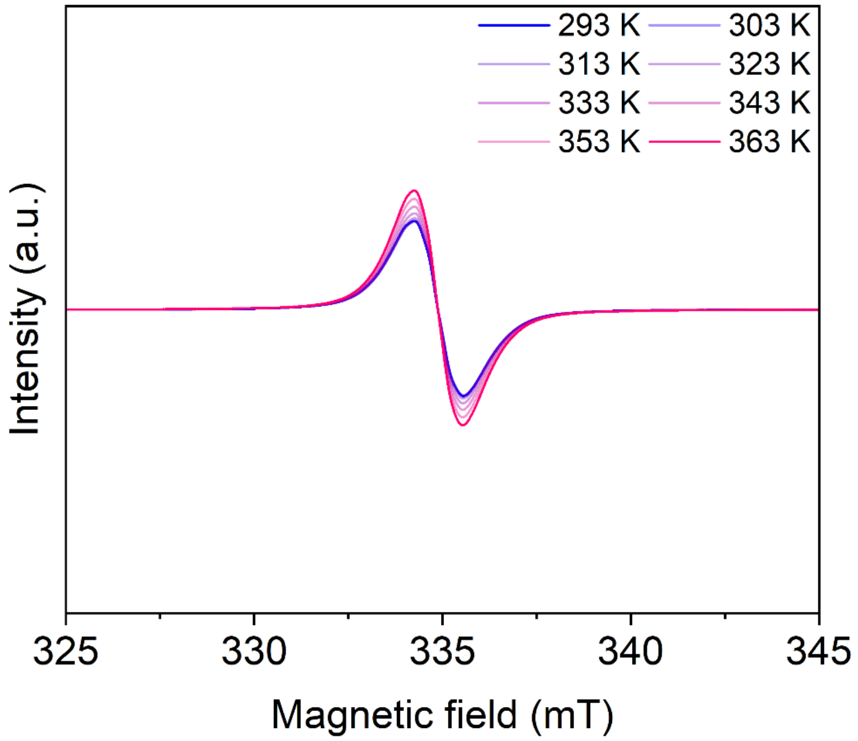

Figure S22: VT-EPR spectrum of **N⁴** in solid state (90 s sweep time; 3 accumulations).

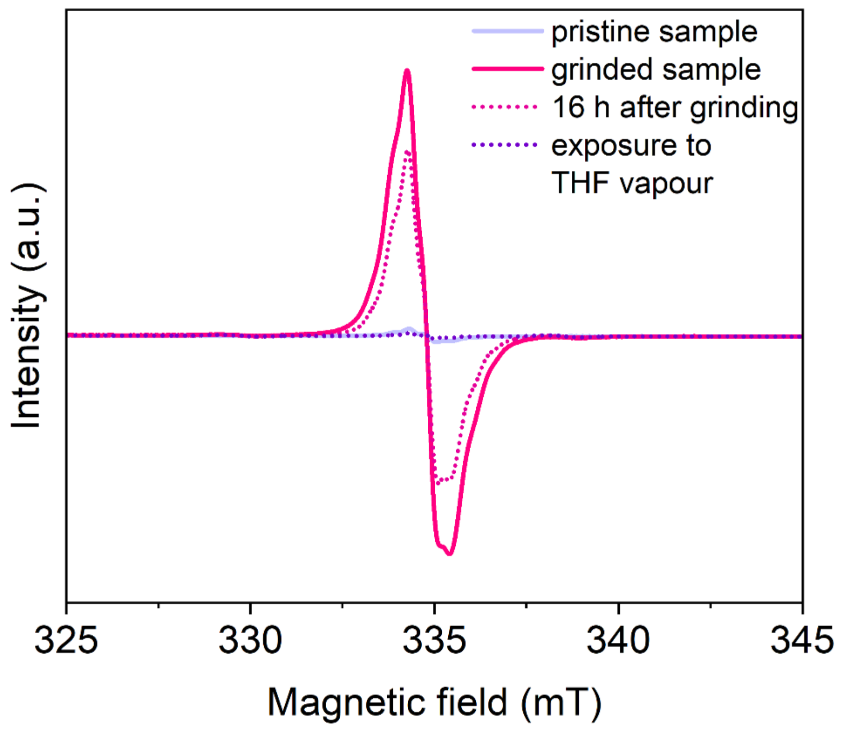

Figure S23: EPR spectrum of $\mathbf{S^2}$ (4:1 mixture of NaCl:$\mathbf{S^2}$) in the solid-state, after grinding, 16 hours after grinding, and after exposure to THF vapour (90 s sweep time; 1 accumulation).

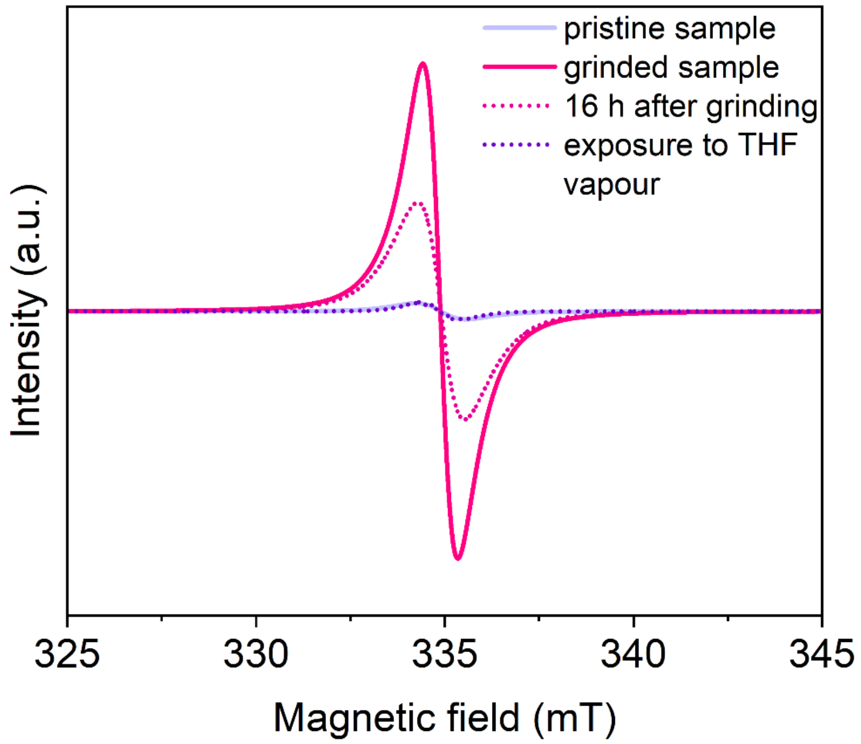

Figure S24: EPR spectrum of $\mathbf{N^4}$ (9:1 mixture of NaCl:$\mathbf{N^4}$) in the solid-state, after grinding, 16 hours after grinding, and after exposure to THF vapour (60 s sweep time; 1 accumulation).

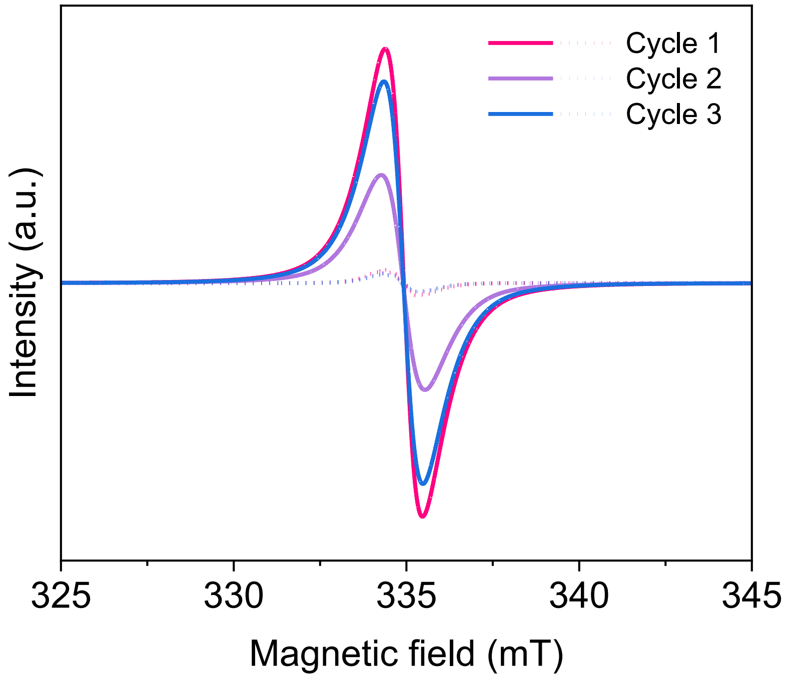

Figure S25: EPR spectra of **N⁴** (9:1 mixture of NaCl:**N⁴**) in the solid state over 3 grinding/healing cycles (60 s sweep time; 3 accumulation).

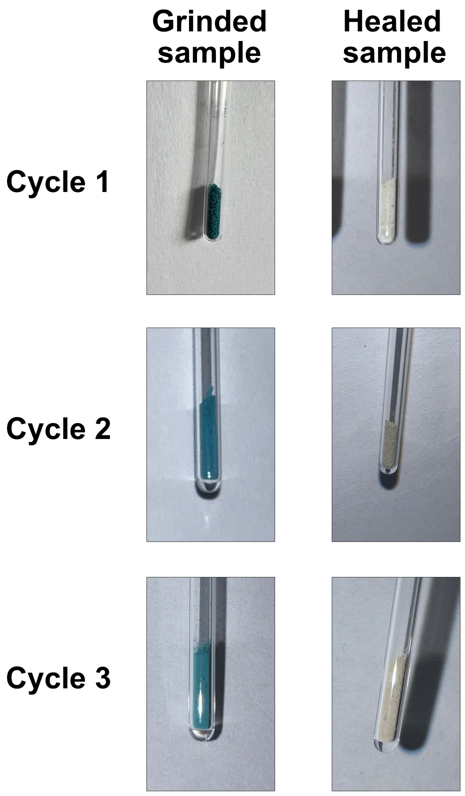

Figure S26: Images of **N⁴** (9:1 NaCl:**N⁴**) after grinding and self-healing during the three performed cycles.

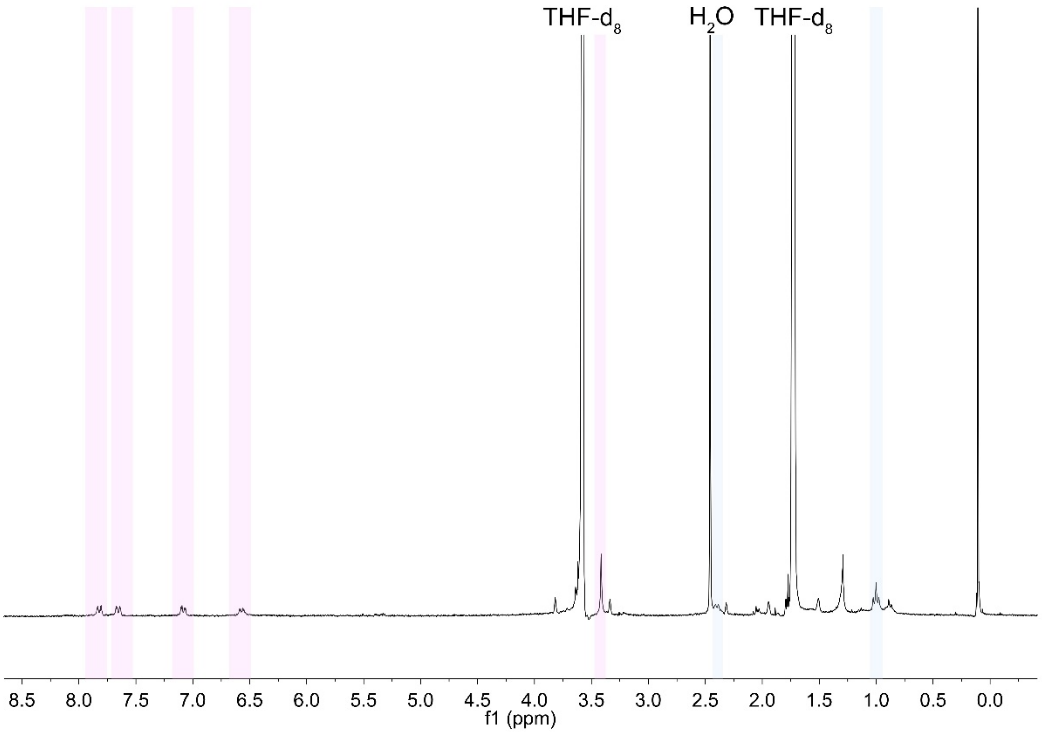

Figure S27: ^{1}H NMR spectrum (THF-d$_8$, 298 K, 300 MHz) of **N**^{4} after grinding/healing cycles.

The band at 2190 cm^{-1}, corresponds to a CN stretching, *v*(CN), observed in nonconjugated nitriles, which is equivalent to the σ-dimer situation of the equilibrium. This band intensifies again after the self-healing *via* THF vapour exposure, which supports the successful self-healing of the material.[10]

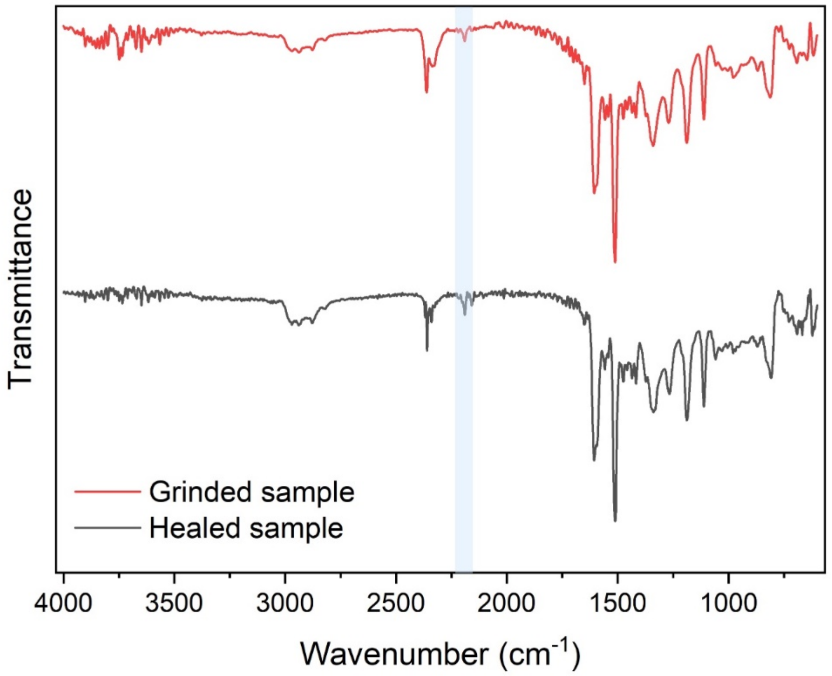

Figure S28: FTIR spectra of **N**^{4} (9:1 NaCl:**N**^{4}) after grinding and after self-healing with marked band in blue at 2190 cm^{-1}.

9 VT-NMR spectra

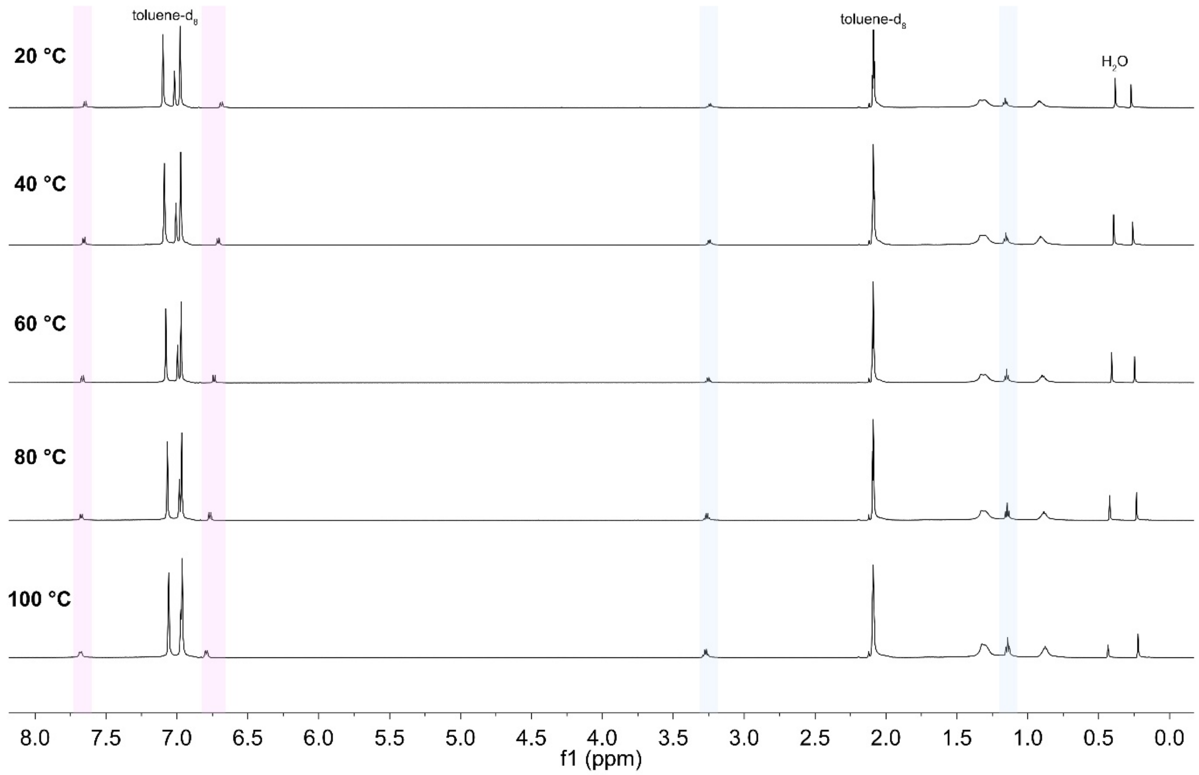

Figure S29: VT-^{1}H NMR spectra (toluene-d$_8$, 600 MHz) of $\mathbf{S^2}$ at different temperatures.

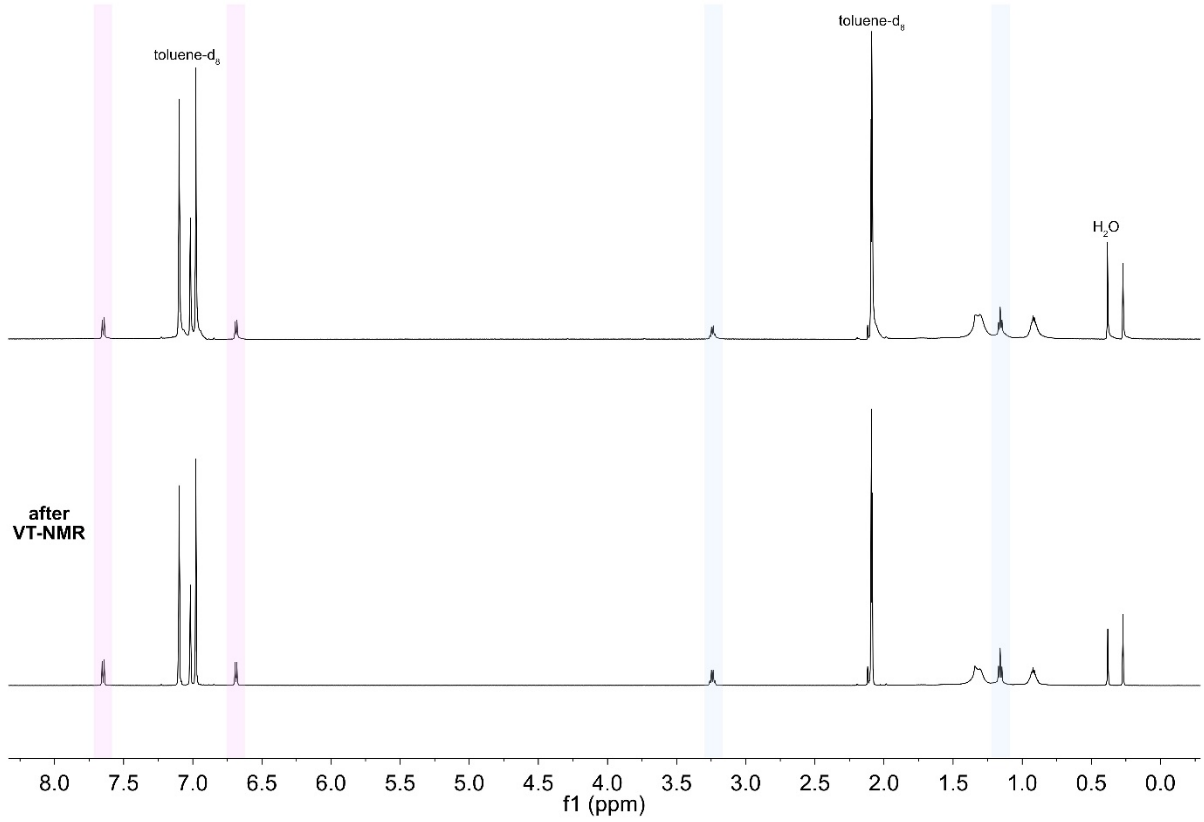

Figure S30: ^{1}H NMR spectra (toluene-d$_8$, 298 K, 600 MHz) of $\mathbf{S^2}$ before and after VT-NMR study.

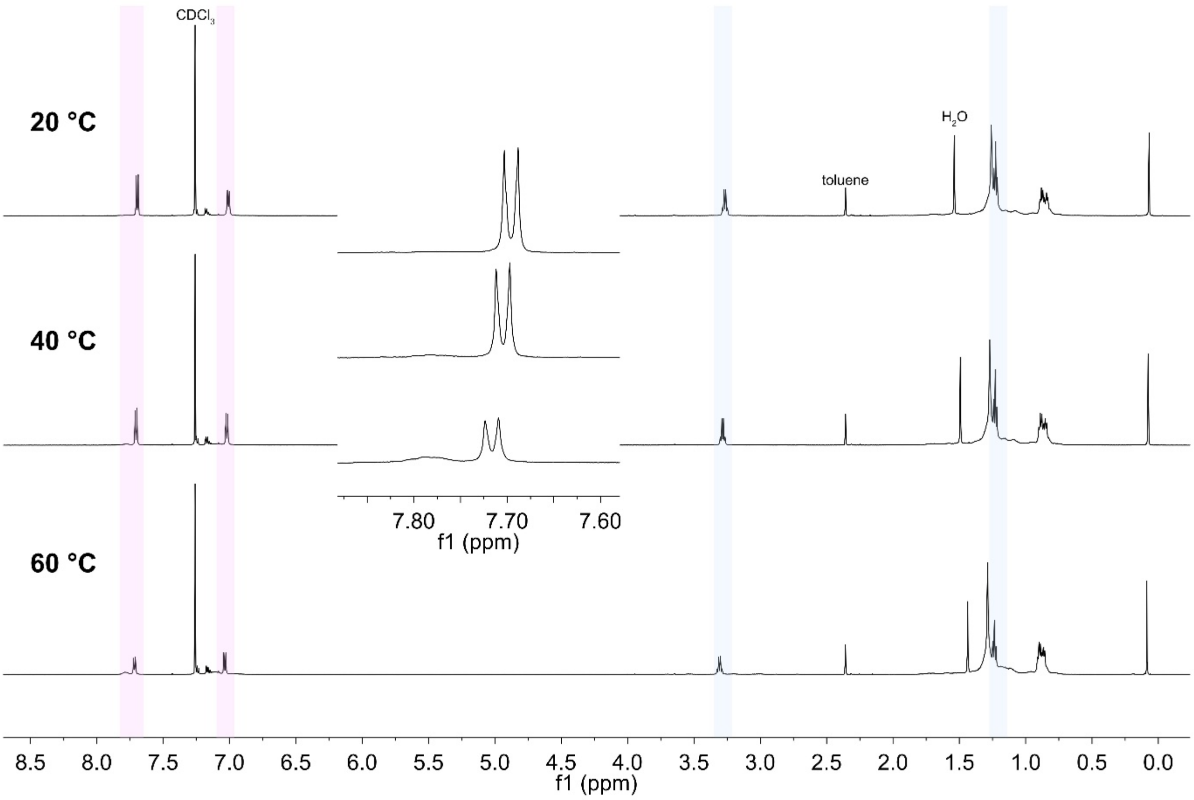

Figure S31: VT-^{1}H NMR spectra ($CDCl_3$, 600 MHz) of **S^2** at different temperatures.

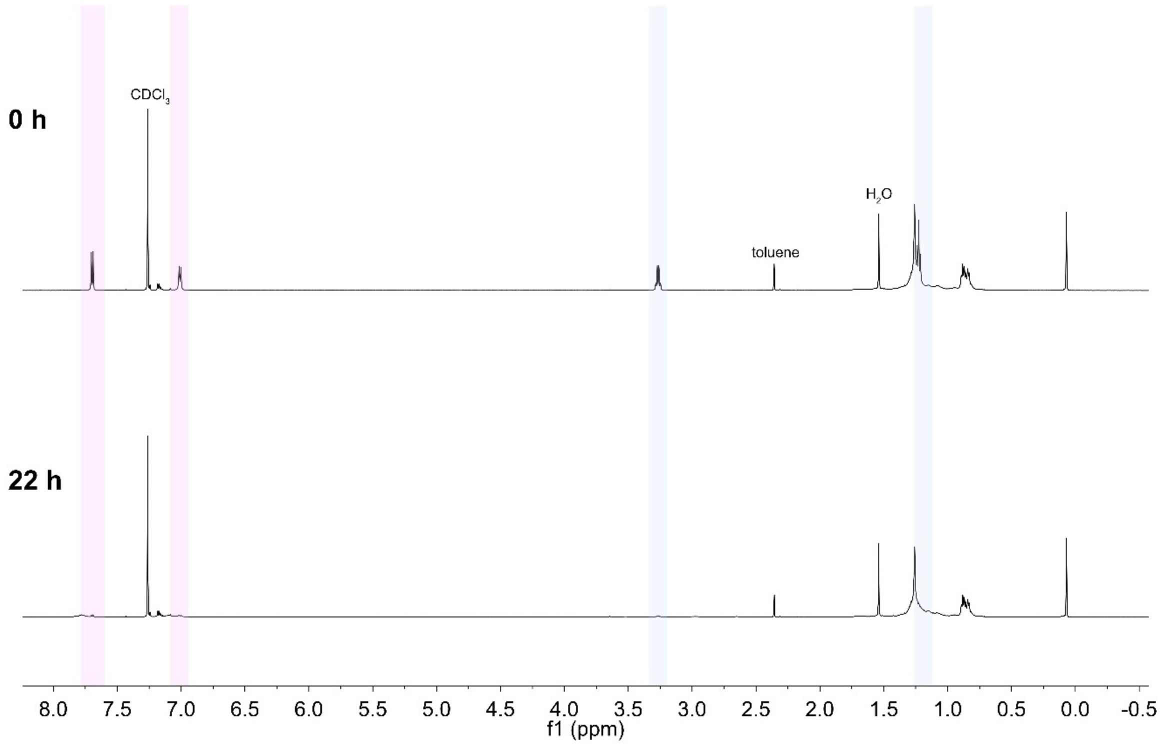

Figure S32: ^{1}H NMR spectra ($CDCl_3$, 298 K, 600 MHz) of **S^2** before and after VT-NMR study.

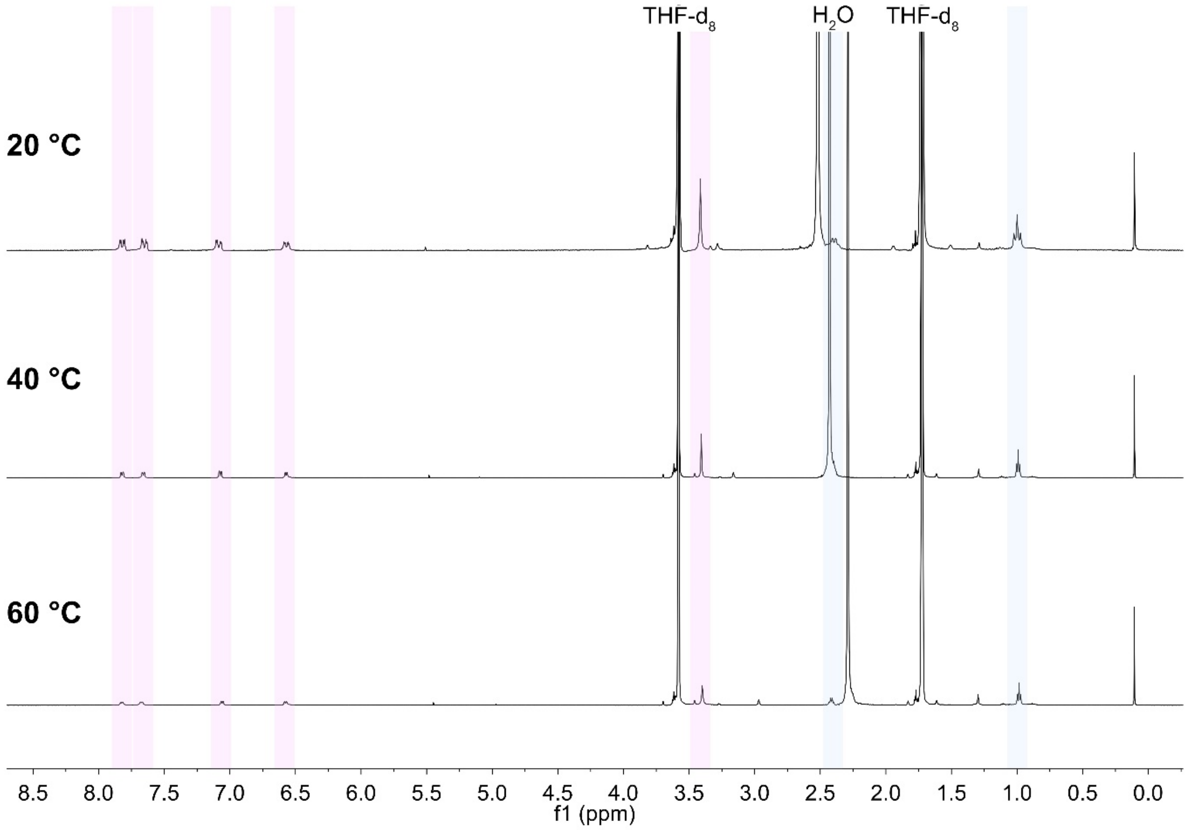

Figure S33: VT-^{1}H NMR spectra (THF-d8, 600 MHz) of $\mathbf{N^4}$ at different temperatures.

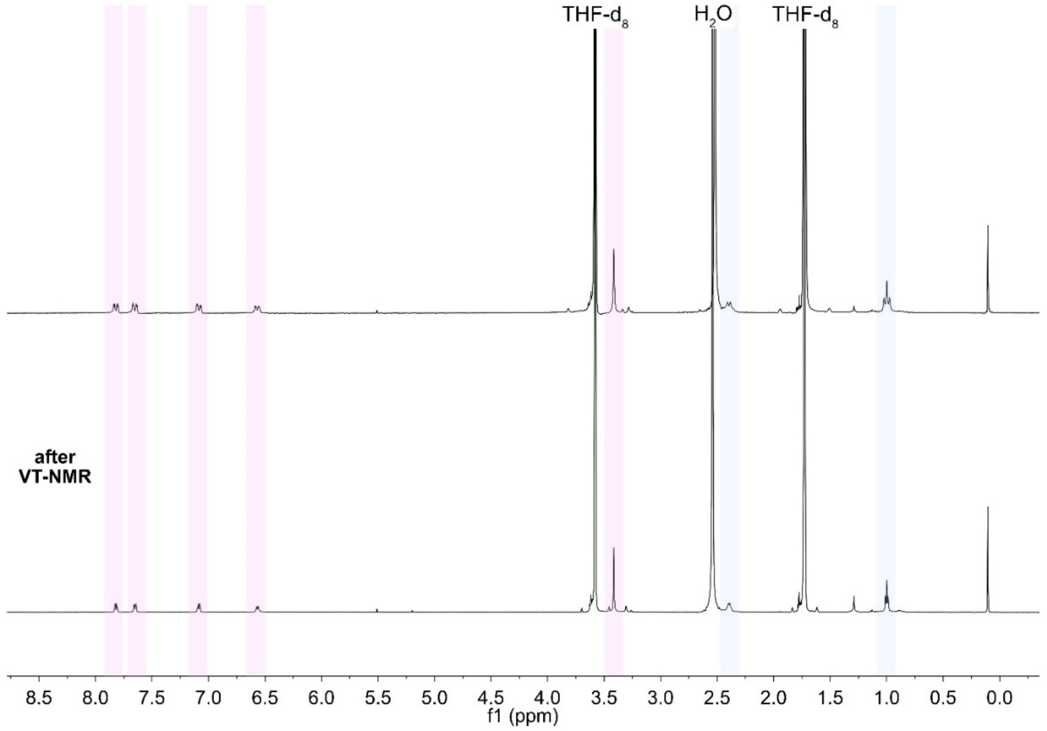

Figure S34: ^{1}H NMR spectra (THF-d8, 298 K, 600 MHz) of $\mathbf{N^4}$ before and after VT-NMR study.

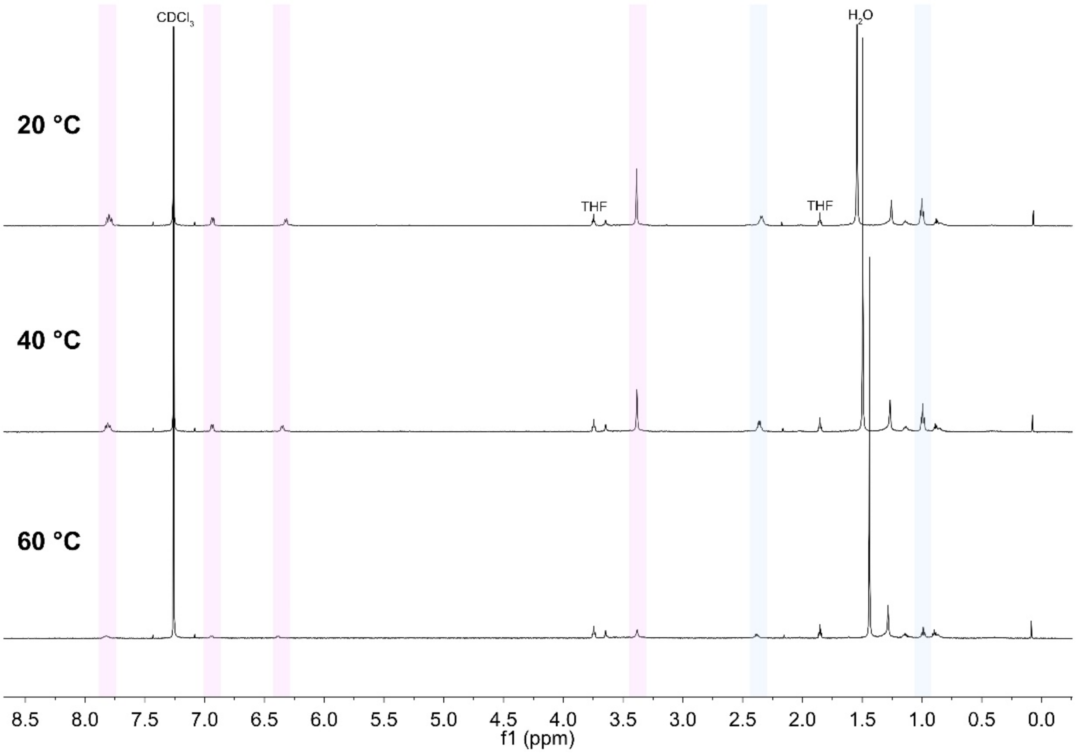

Figure S35: VT-^{1}H NMR spectra ($CDCl_3$, 600 MHz) of **N[4]** at different temperatures.

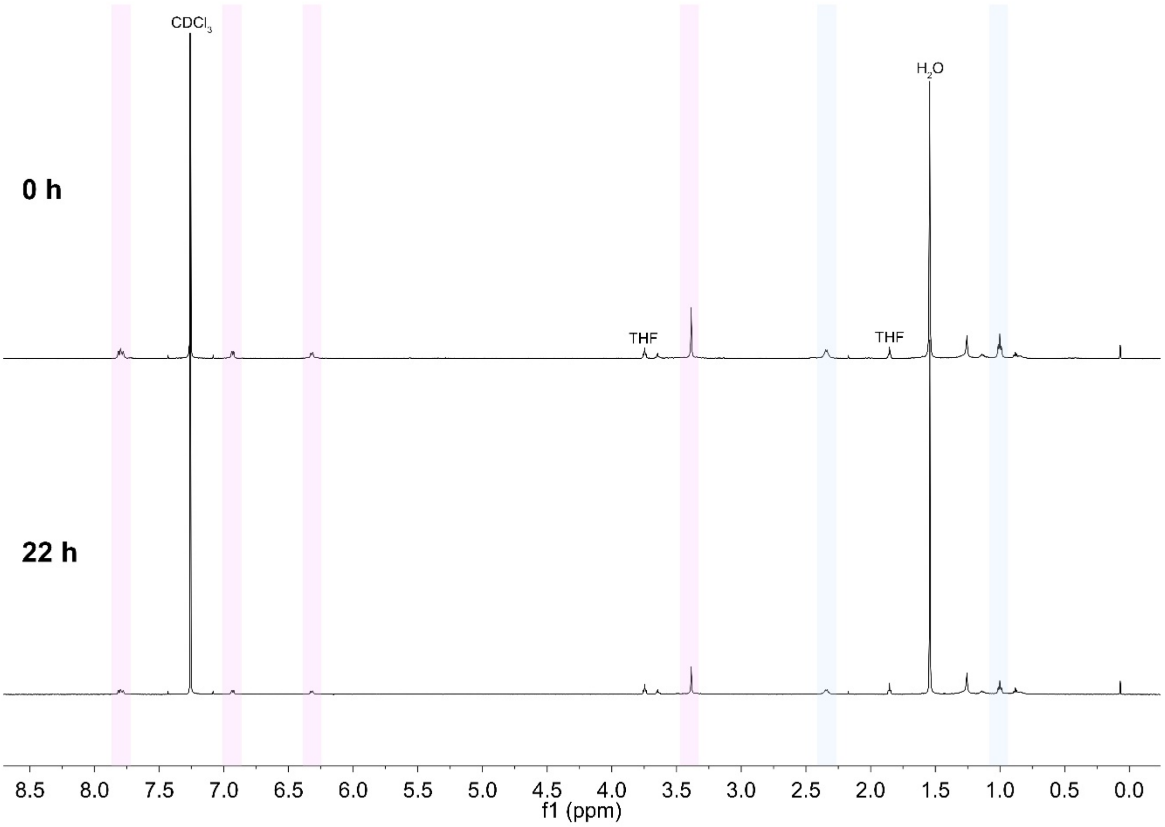

Figure S36: ^{1}H NMR spectra ($CDCl_3$, 298 K, 600 MHz) of **N[4]** before and after VT-NMR study.

10 Stability Investigation

To evaluate the chemical stability of the cages, **N^4** (2.8 mg and 2.5 mg) and **S^2** (2.6 mg and 3.1 mg) were submerged in acidic and basic aqueous solutions (1M HCl and 1M NaOH) for 24 h. After filtration and redissolution in deuterated solvents, both systems were fully recovered and showed no indication of decomposition by ^{1}H NMR spectroscopy, demonstrating good stability under these conditions. In addition, the stability under visible light was investigated over a period of two weeks. **S^2** showed no detectable signs of decomposition. For **N^4**, minor changes in the ^{1}H NMR spectra were observed, accompanied by a slight colour change, indicating a certain sensitivity toward light. We therefore recommend storage under exclusion of light. These results demonstrate that both cage systems exhibit good chemical stability under a range of conditions, with only minor limitations for **N^4** under prolonged light exposure

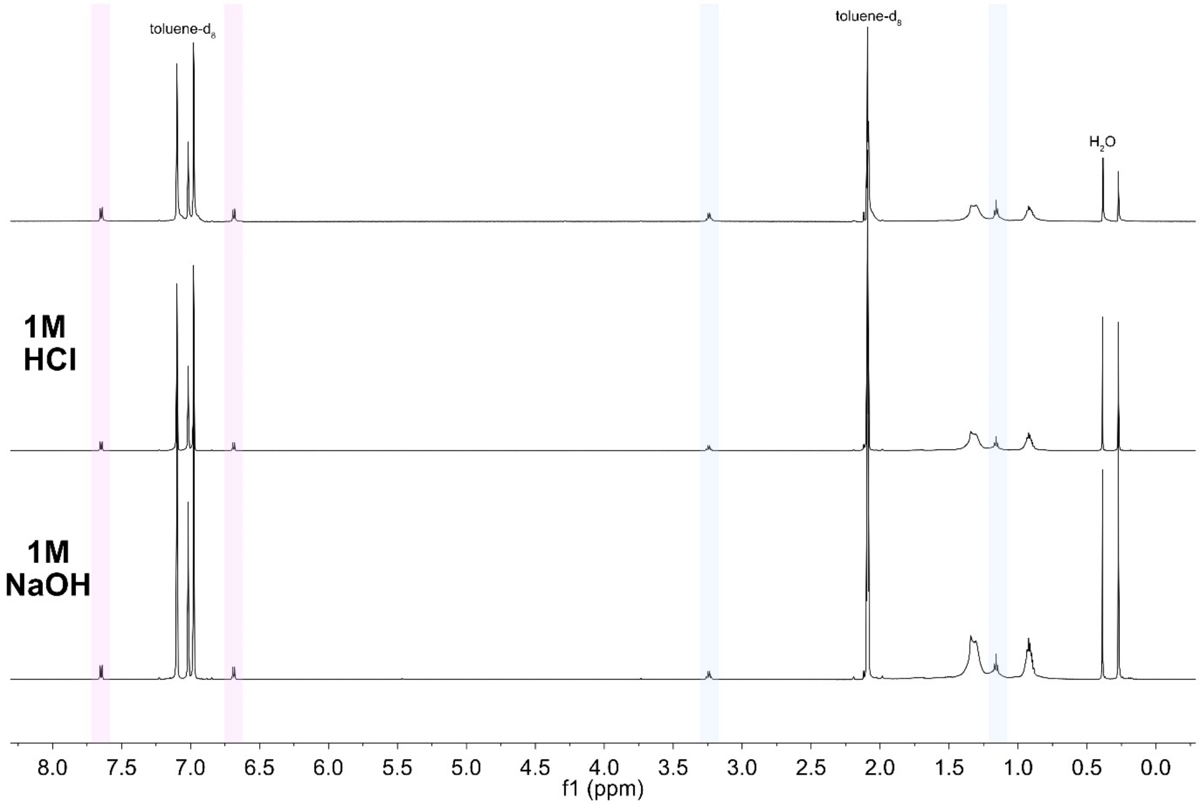

Figure S37: ^{1}H NMR spectra (toluene-d$_8$, 298 K, 600 MHz) of **S^2** before and after 24 h long exposure to aqueous 1M HCl and 1M NaOH solution.

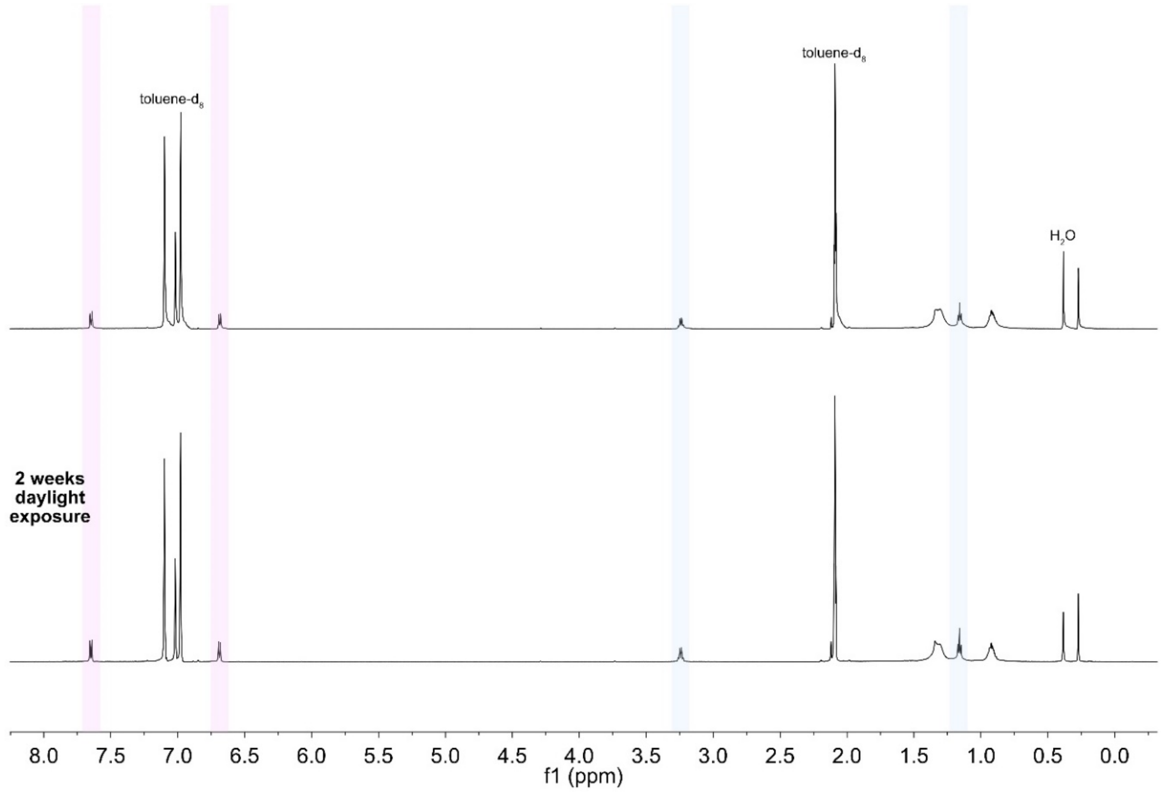

Figure S38: ^{1}H NMR spectra (toluene-d$_8$, 298 K, 600 MHz) of $\mathbf{S^2}$ before and after 2 w long exposure to daylight.

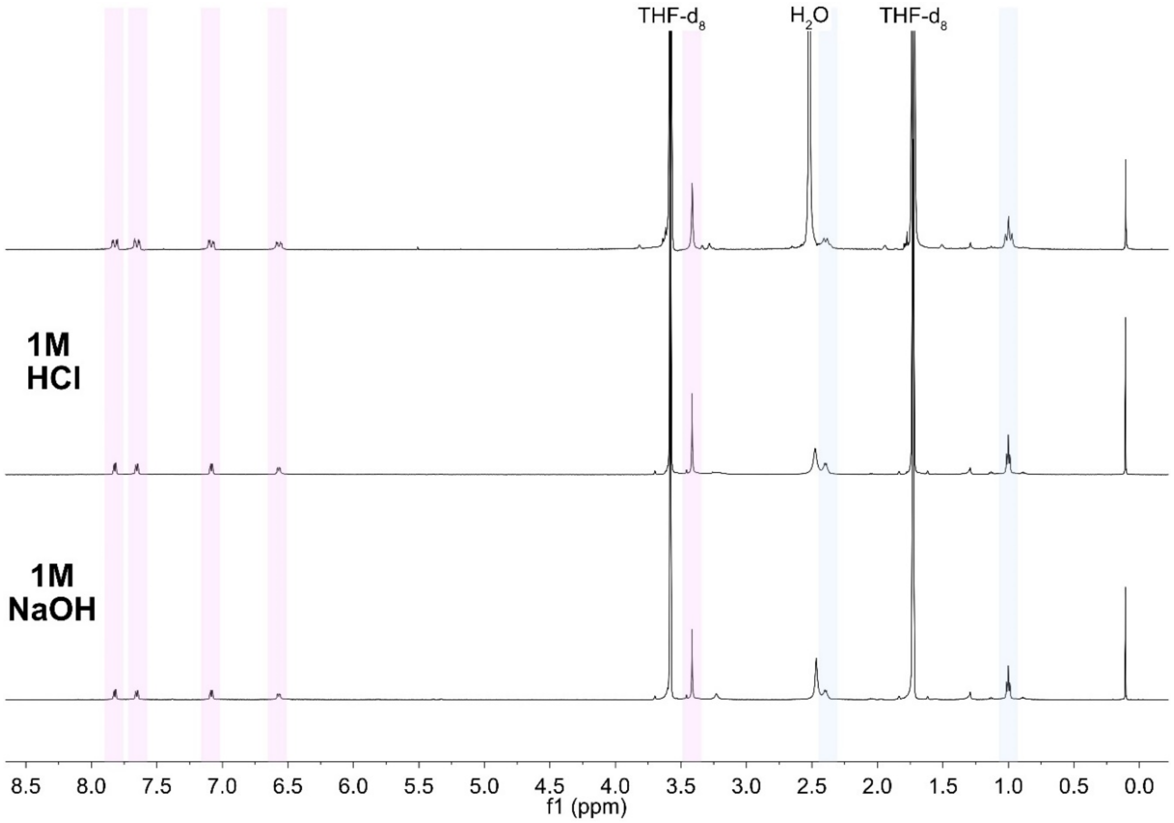

Figure S39: ^{1}H NMR spectra (THF-d$_8$, 298 K, 600 MHz) of $\mathbf{N^4}$ before and after 24 h long exposure to aqueous 1M HCl and 1M NaOH solution.

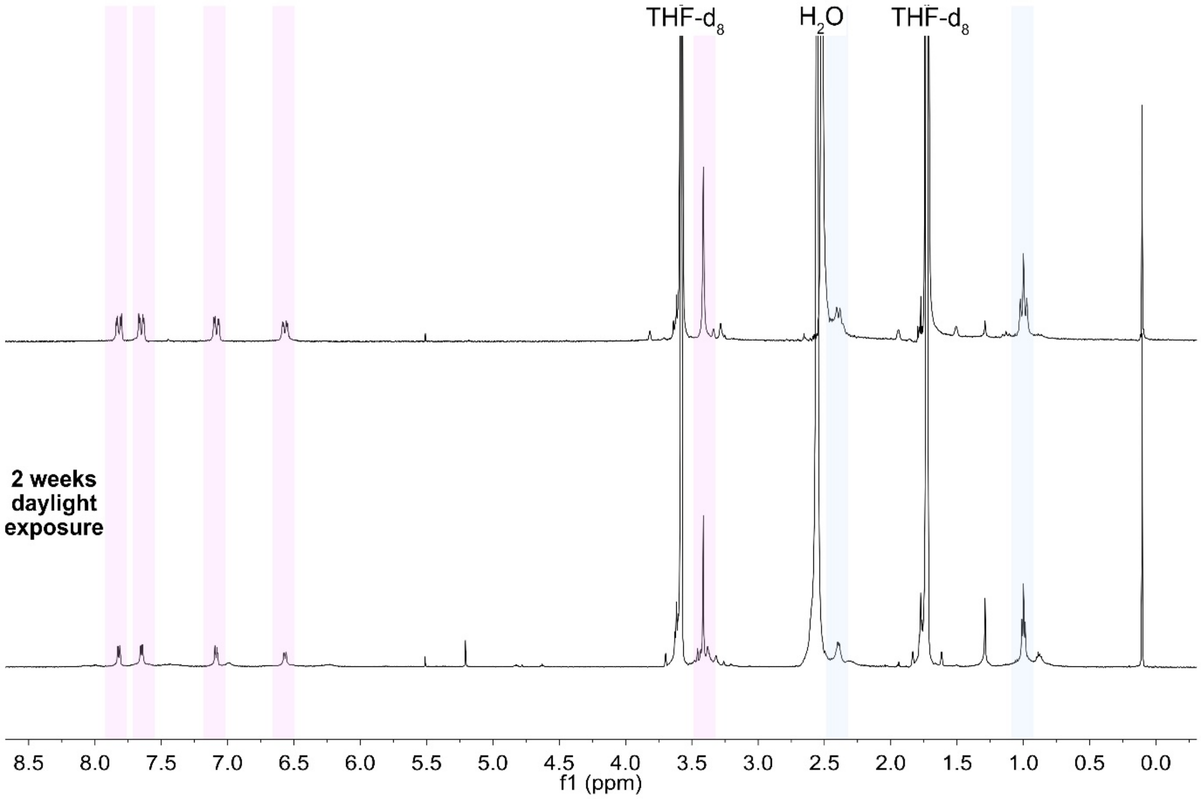

Figure S40: ^{1}H NMR spectra (THF-d8, 298 K, 600 MHz) of **N[4]** before and after 2 w long exposure to daylight.

11 Thermogravimetric Analysis

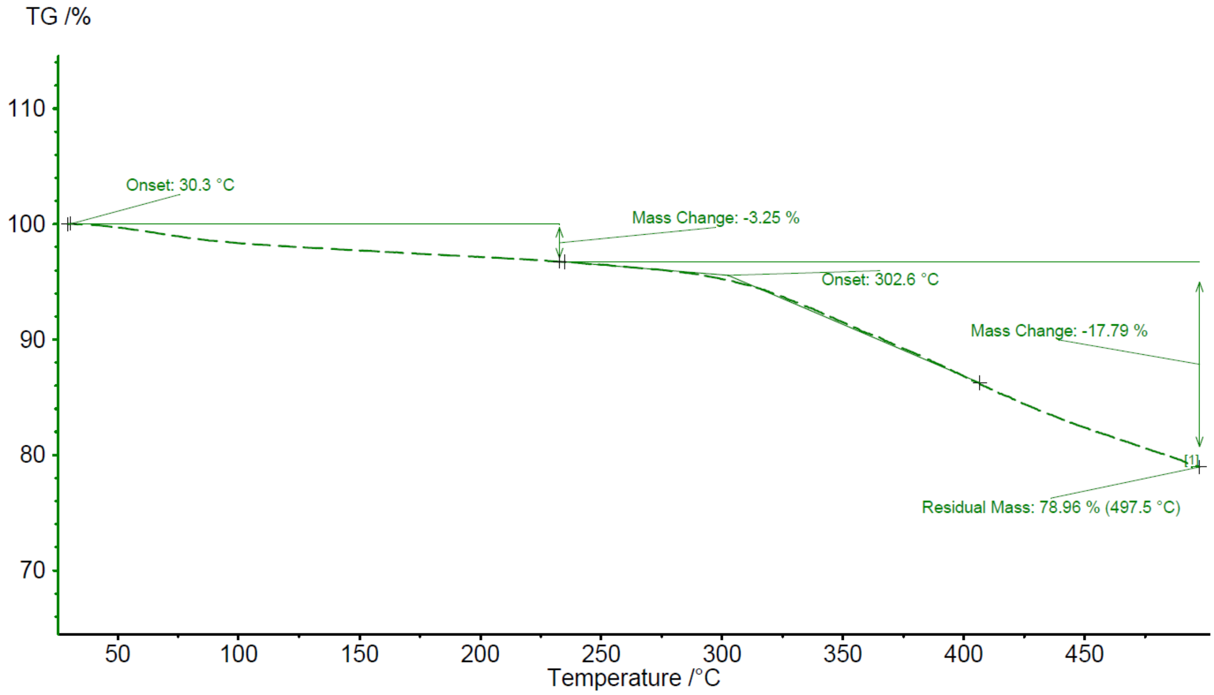

Figure S41: Thermogravimetric analysis of **N⁴**.

12 Spectra

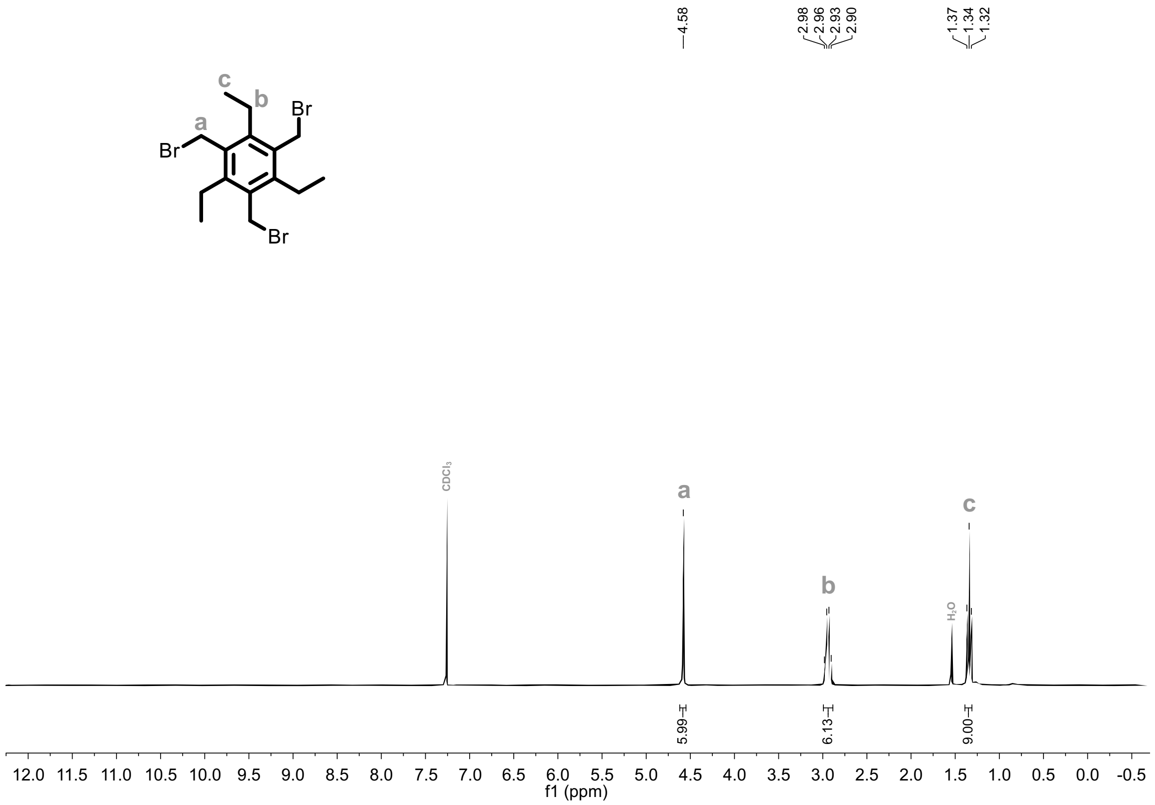

Figure S42: ^{1}H NMR spectrum (300 MHz, $CDCl_3$, 298 K) of **1**.

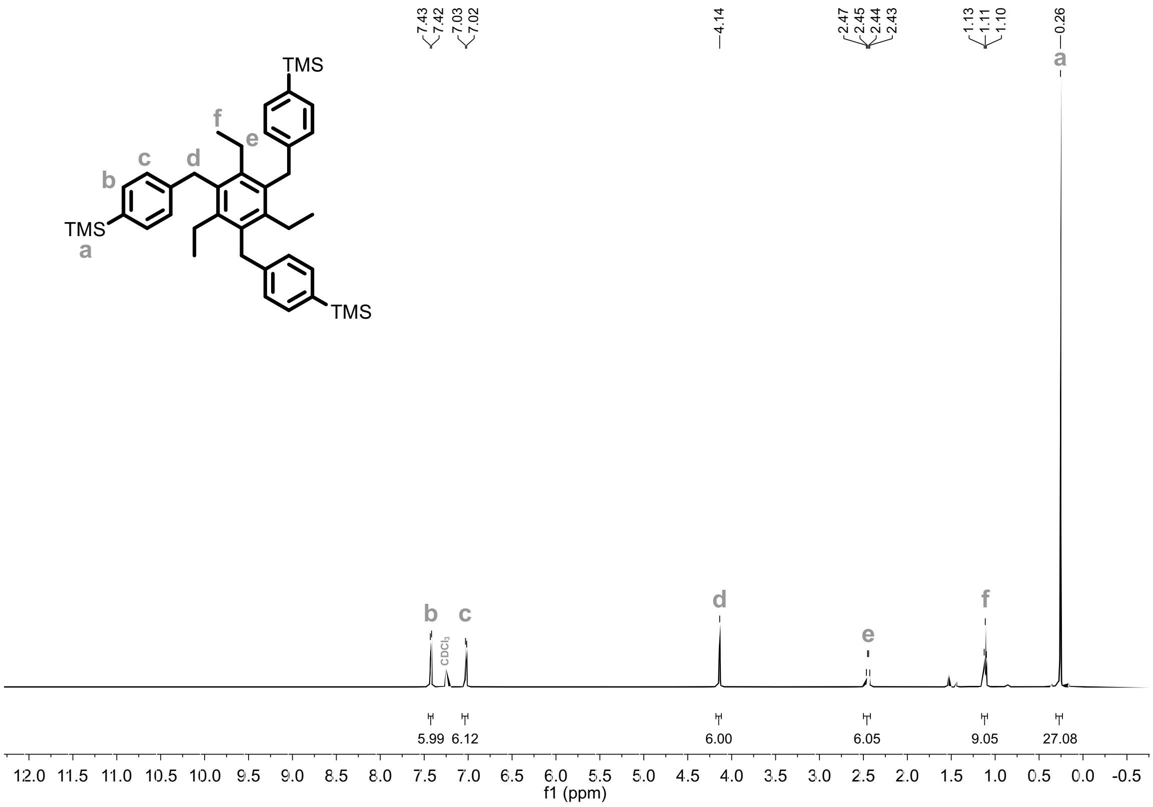

Figure S43: ^{1}H NMR spectrum (600 MHz, $CDCl_3$, 298 K) of **2**.

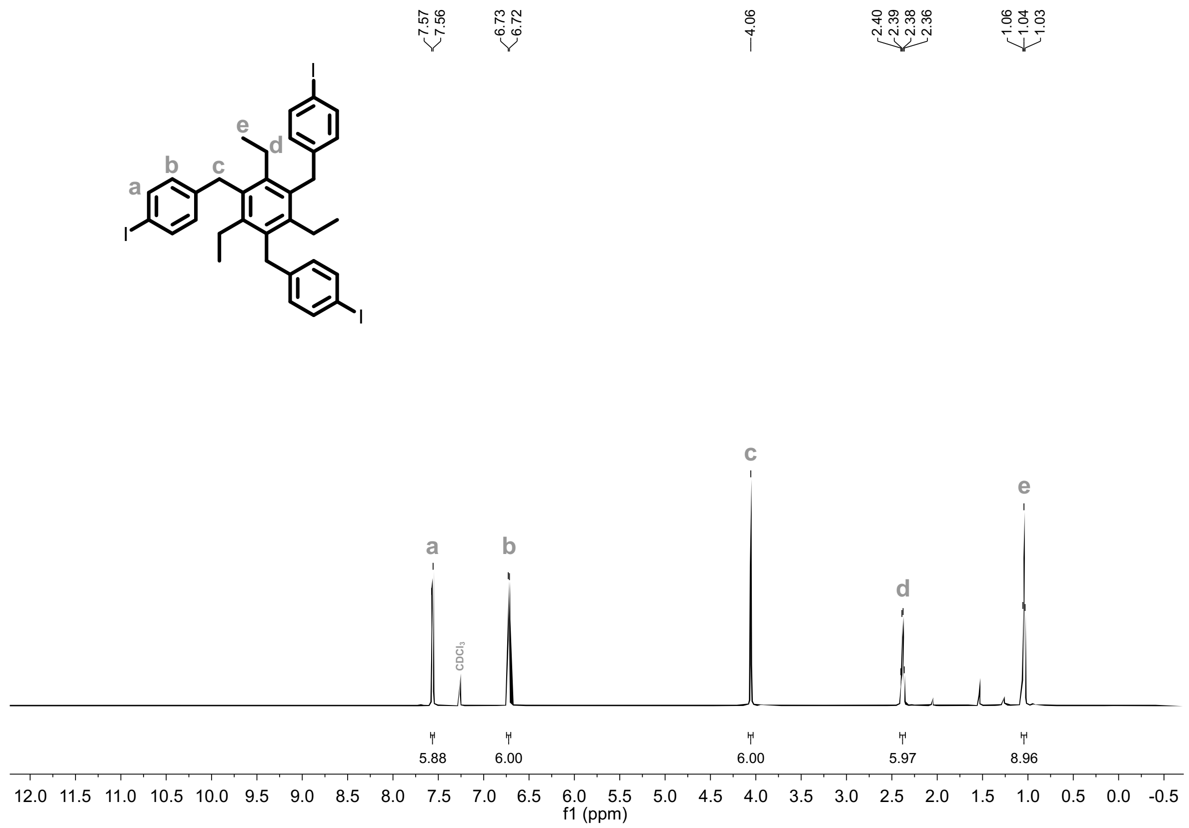

Figure S44: ^{1}H NMR spectrum (600 MHz, $CDCl_3$, 298 K) of **3**.

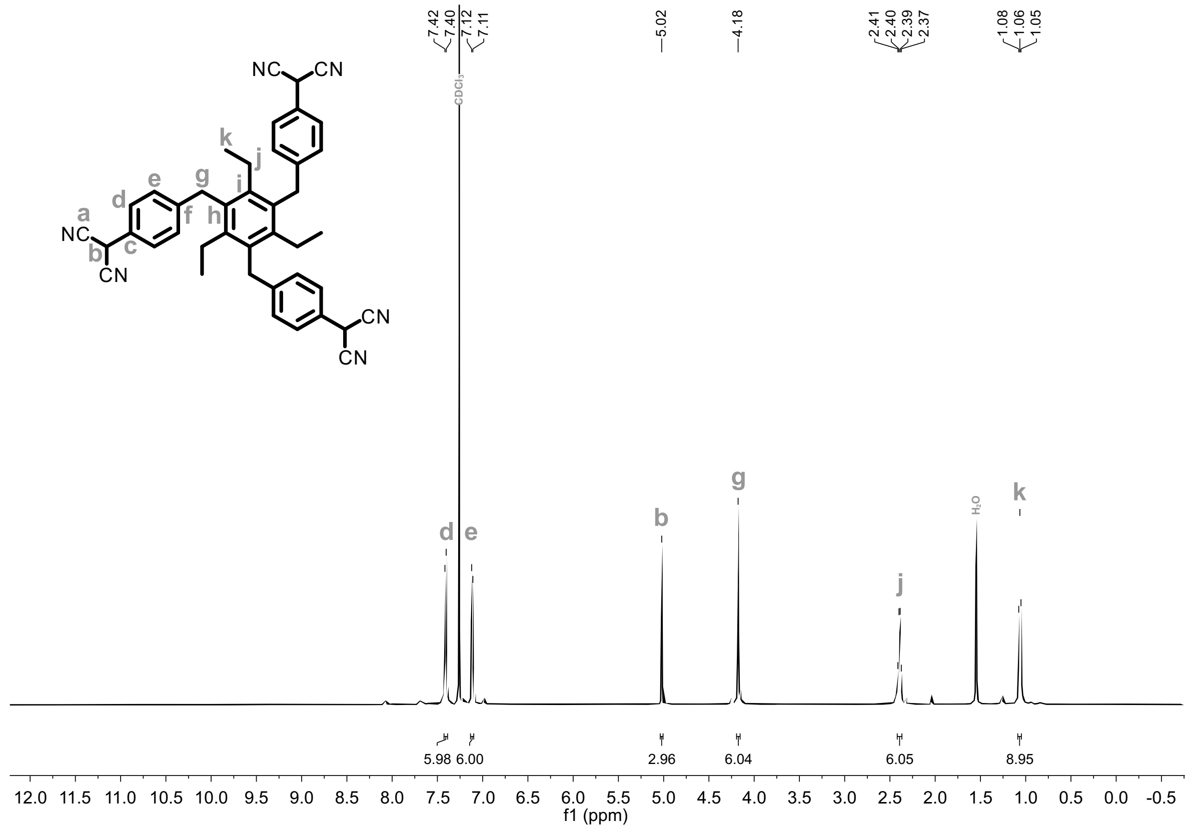

Figure S45: ^{1}H NMR spectrum (600 MHz, $CDCl_3$, 298 K) of **C**.

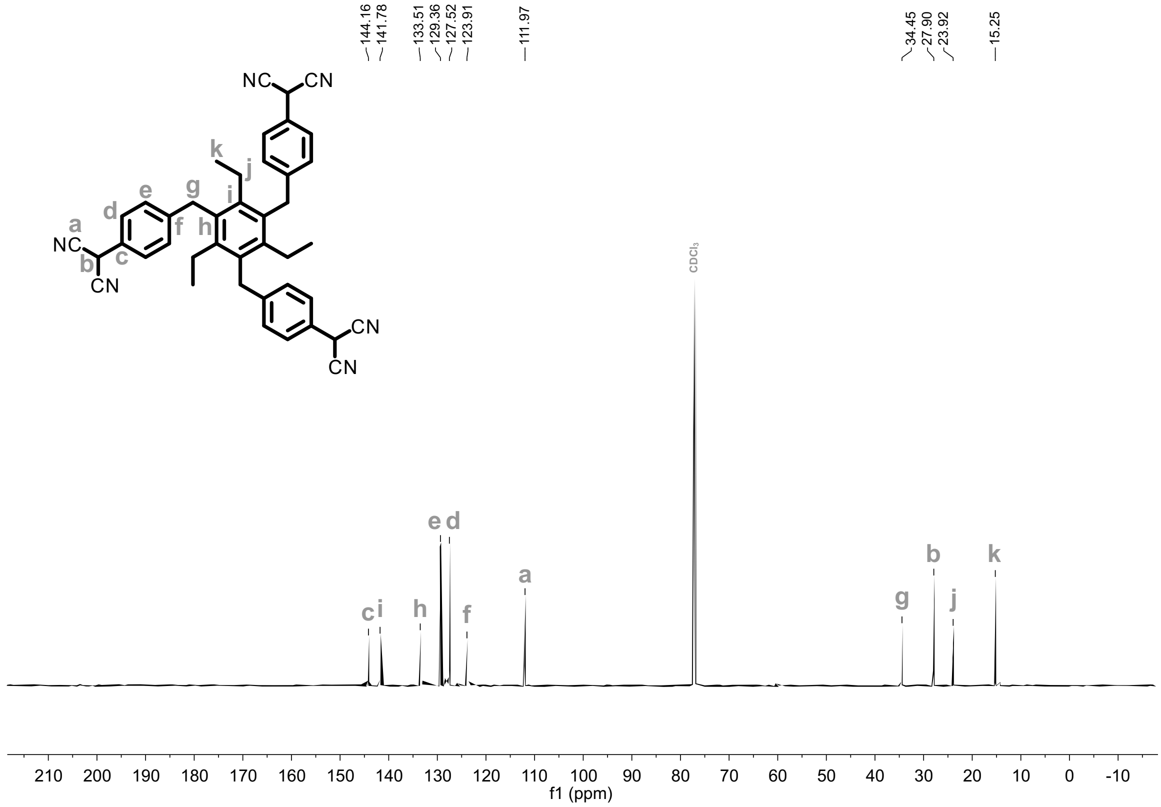

Figure S46: ^{13}C NMR spectrum (151 MHz, $CDCl_3$, 298 K) of **C**.

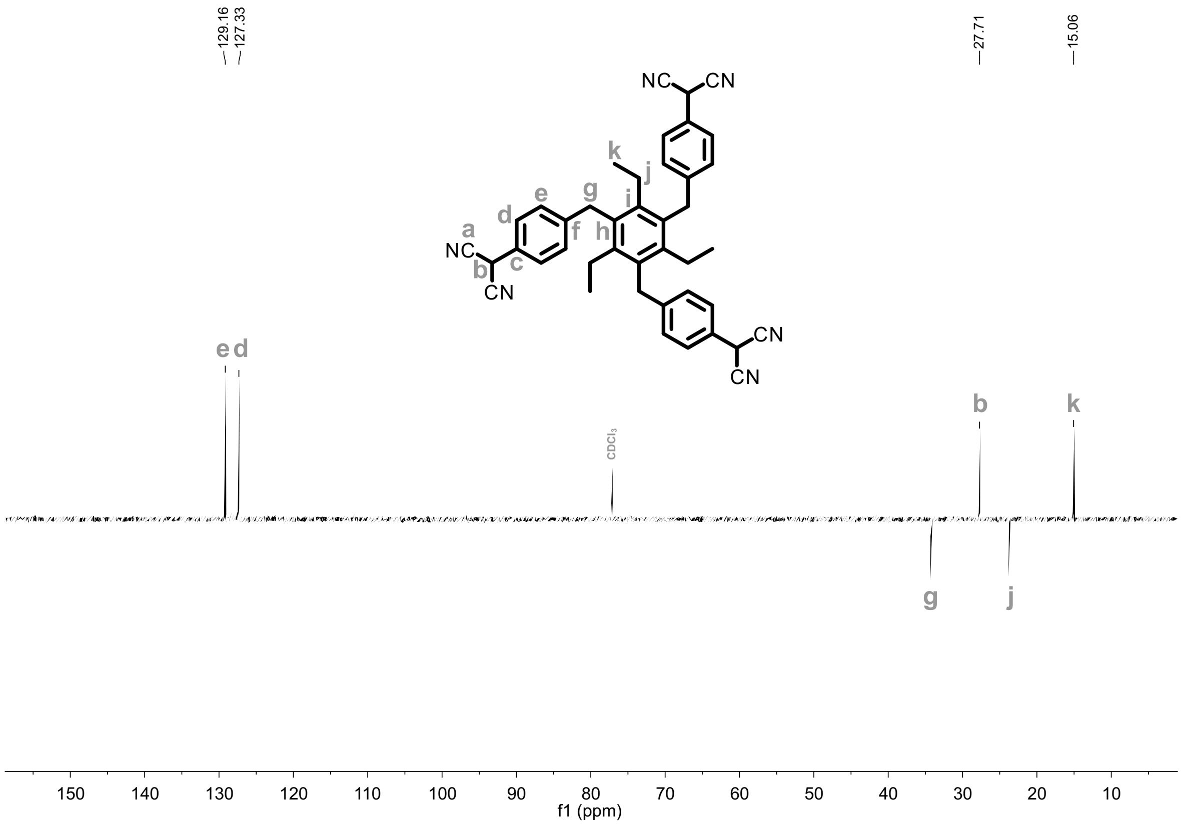

Figure S47: ^{13}C DEPT-135 NMR spectrum (151 MHz, $CDCl_3$, 298 K) of **C**.

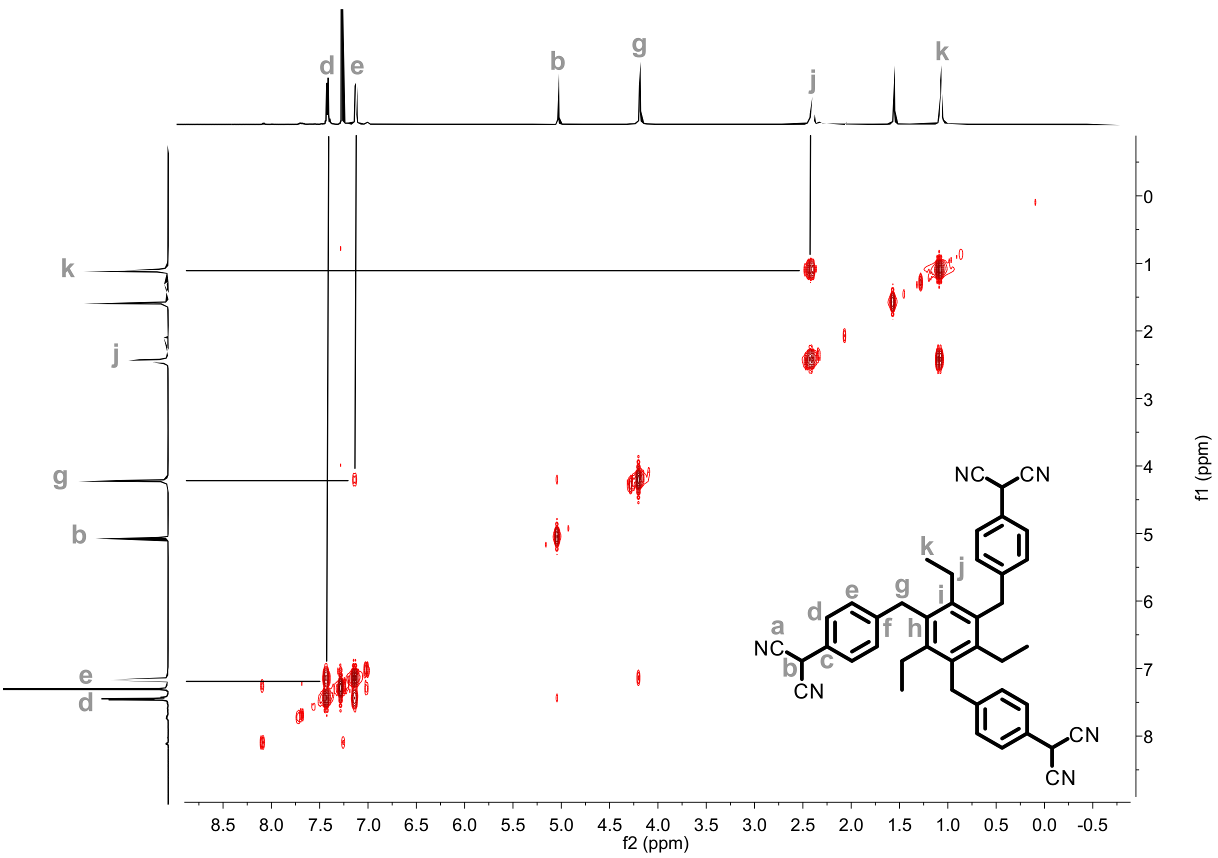

Figure S48: ^{1}H-^{1}H COSY NMR spectrum (600 MHz, $CDCl_3$, 298 K) of **C**.

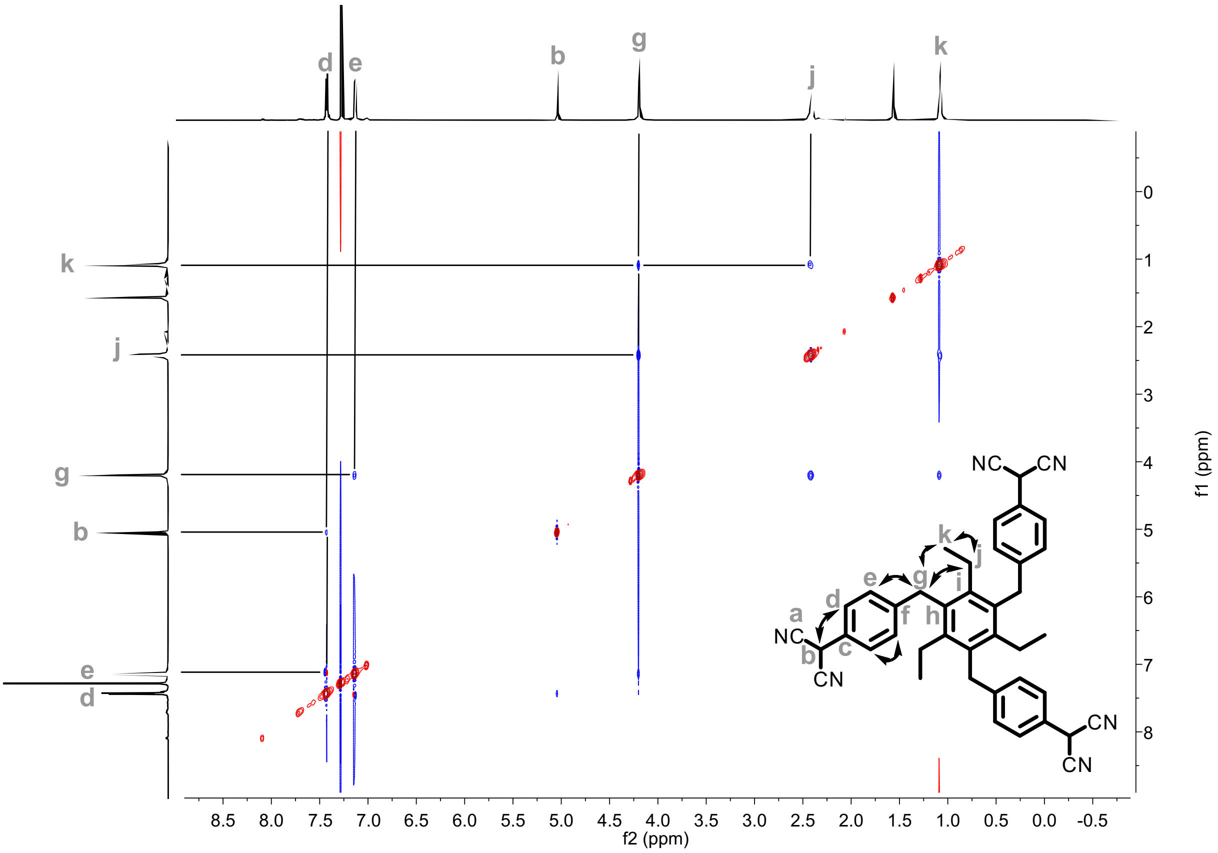

Figure S49: ^{1}H-^{1}H NOESY NMR spectrum (600 MHz, $CDCl_3$, 298 K) of **C**.

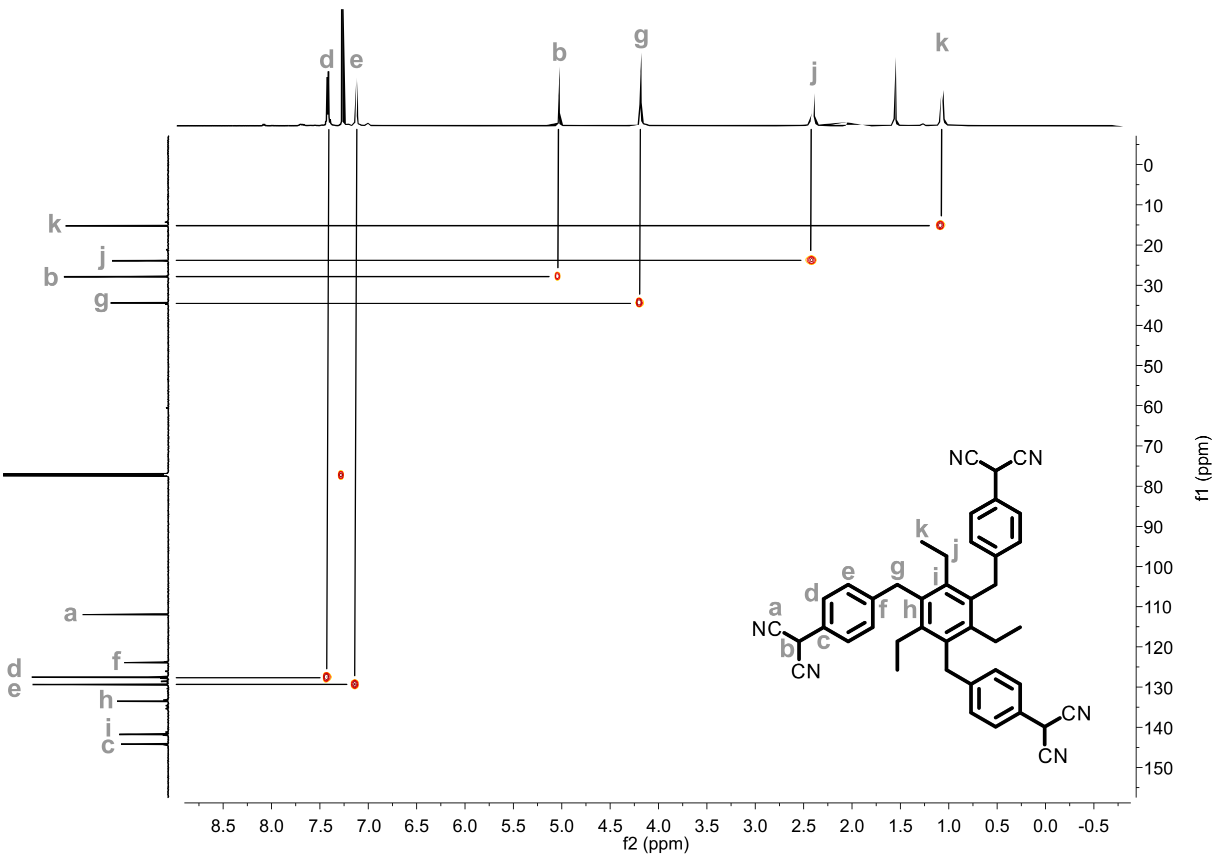

Figure S50: ^{1}H-^{13}C HSQC NMR spectrum (151 MHz, $CDCl_3$, 298 K) of **C**.

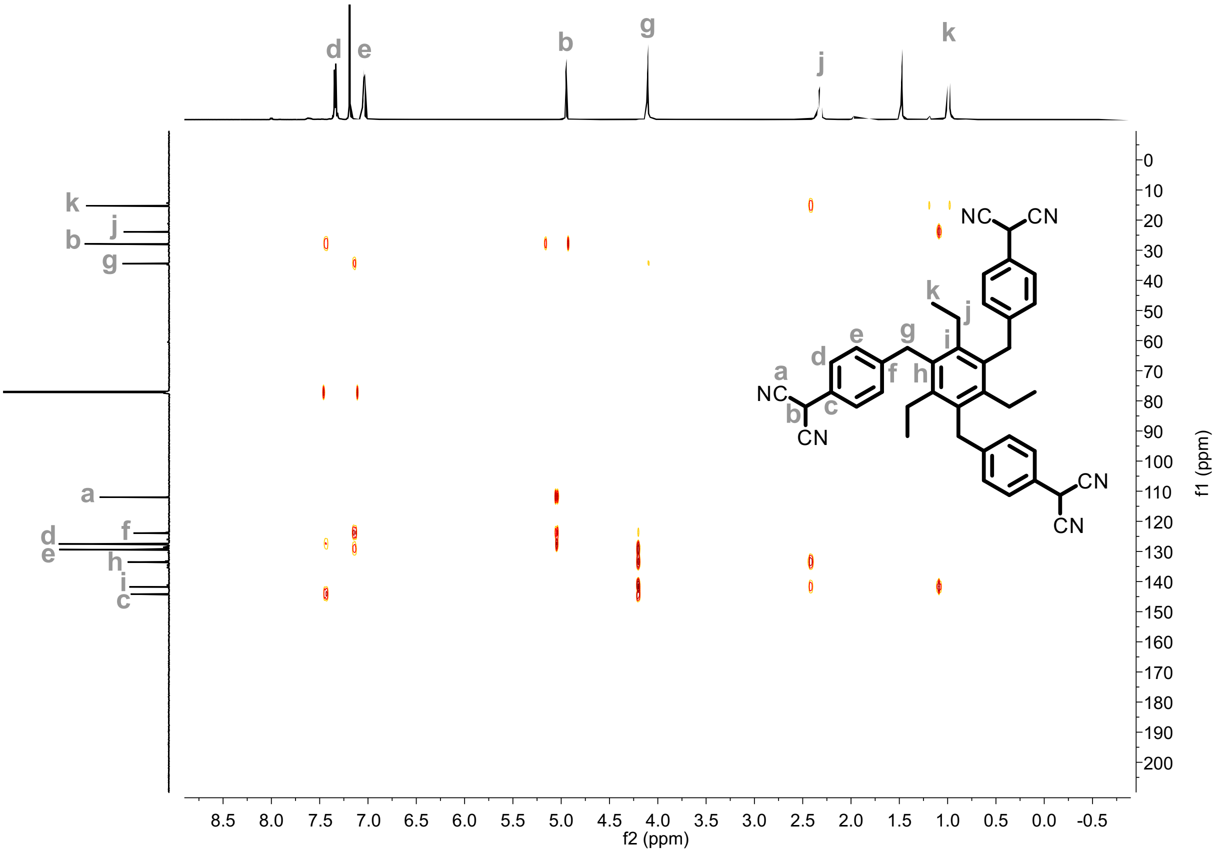

Figure S51: ^{1}H-^{13}C HMBC NMR spectrum (151 MHz, $CDCl_3$, 298 K) of **C**.

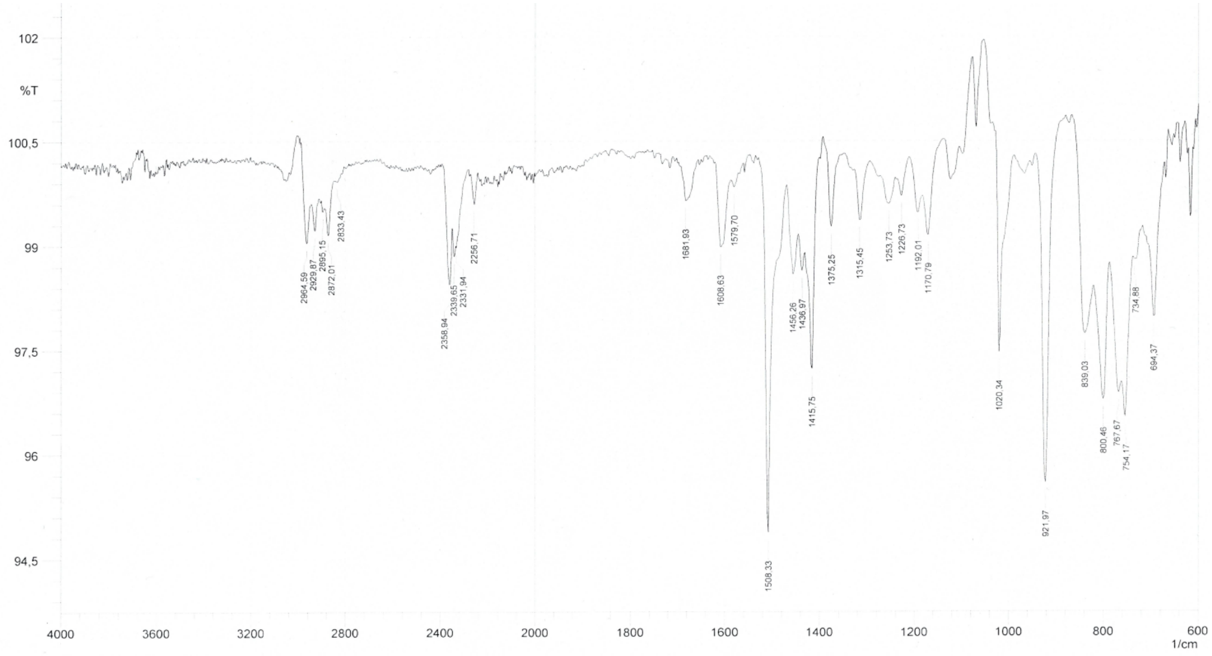

Figure S52: IR spectrum of **C**.

Acquisition Parameter

Source Type	ESI	Ion Polarity	Positive	Set Nebulizer	0.3 bar
Focus	Not active	Set Capillary	3500 V	Set Dry Heater	200 °C
Scan Begin	50 m/z	Set End Plate Offset	-500 V	Set Dry Gas	3.5 l/min
Scan End	1600 m/z	Set Collision Cell RF	500.0 Vpp	Set Divert Valve	Source

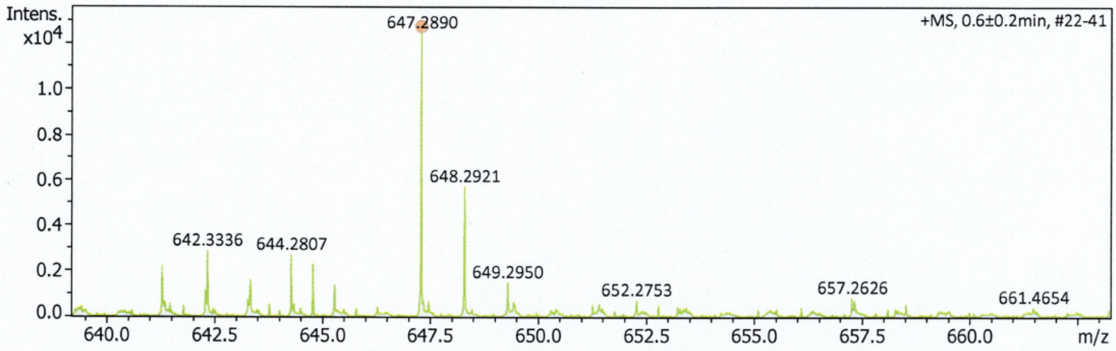

Meas. m/z	#	Ion Formula	m/z	err [ppm]	Mean err [ppm]	rdb (neutral)	N-Rule	e⁻ Conf	mSigma	Std I	Std Mean m/z	Std I VarNorm	Std m/z Diff	Std Comb Dev
647.2890	1	C41H40N2NaO4	647.2880	-1.4	-1.1	23.0	ok	even	10.4	13.8	n.a.	n.a.	n.a.	n.a.
647.2890	2	C42H36N6Na	647.2894	0.6	0.6	28.0	ok	even	14.7	19.7	n.a.	n.a.	n.a.	n.a.

Figure S53: HRMS (ESI) spectrum of **C**.

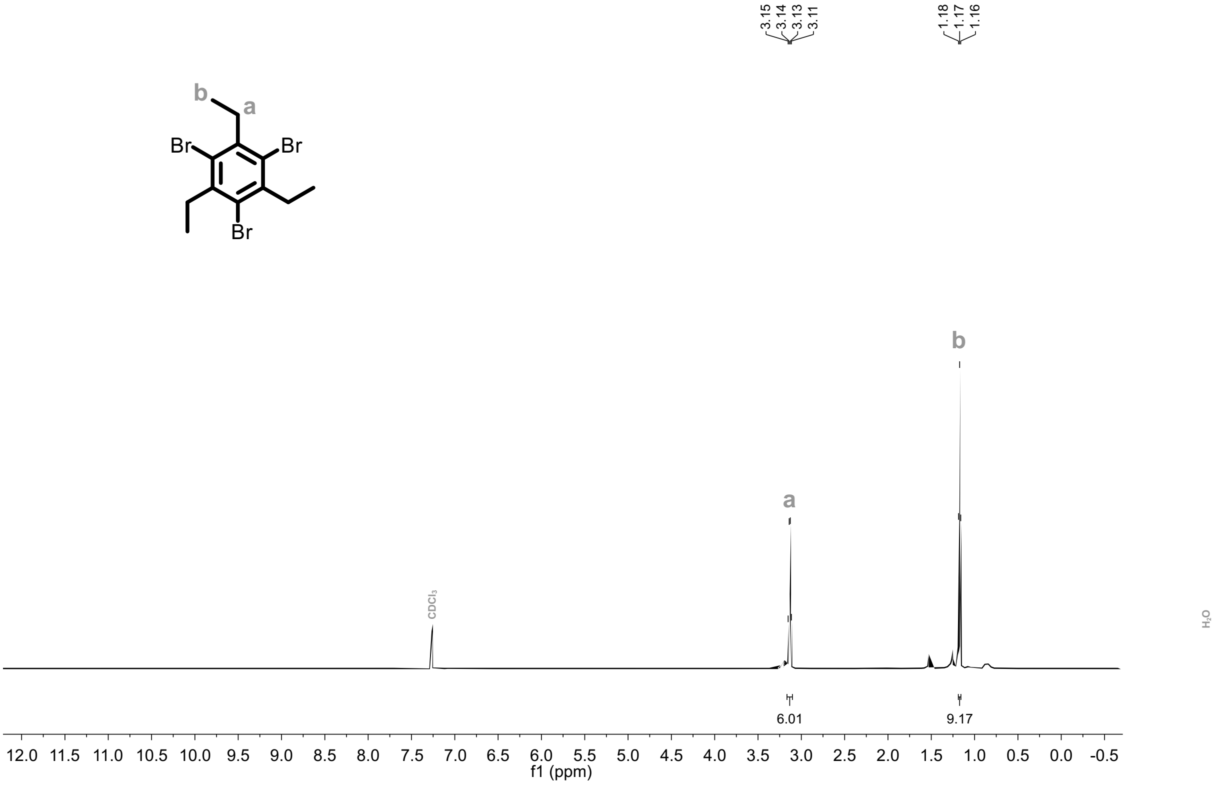

Figure S54: ^{1}H NMR spectrum (600 MHz, $CDCl_3$, 298 K) of **4**.

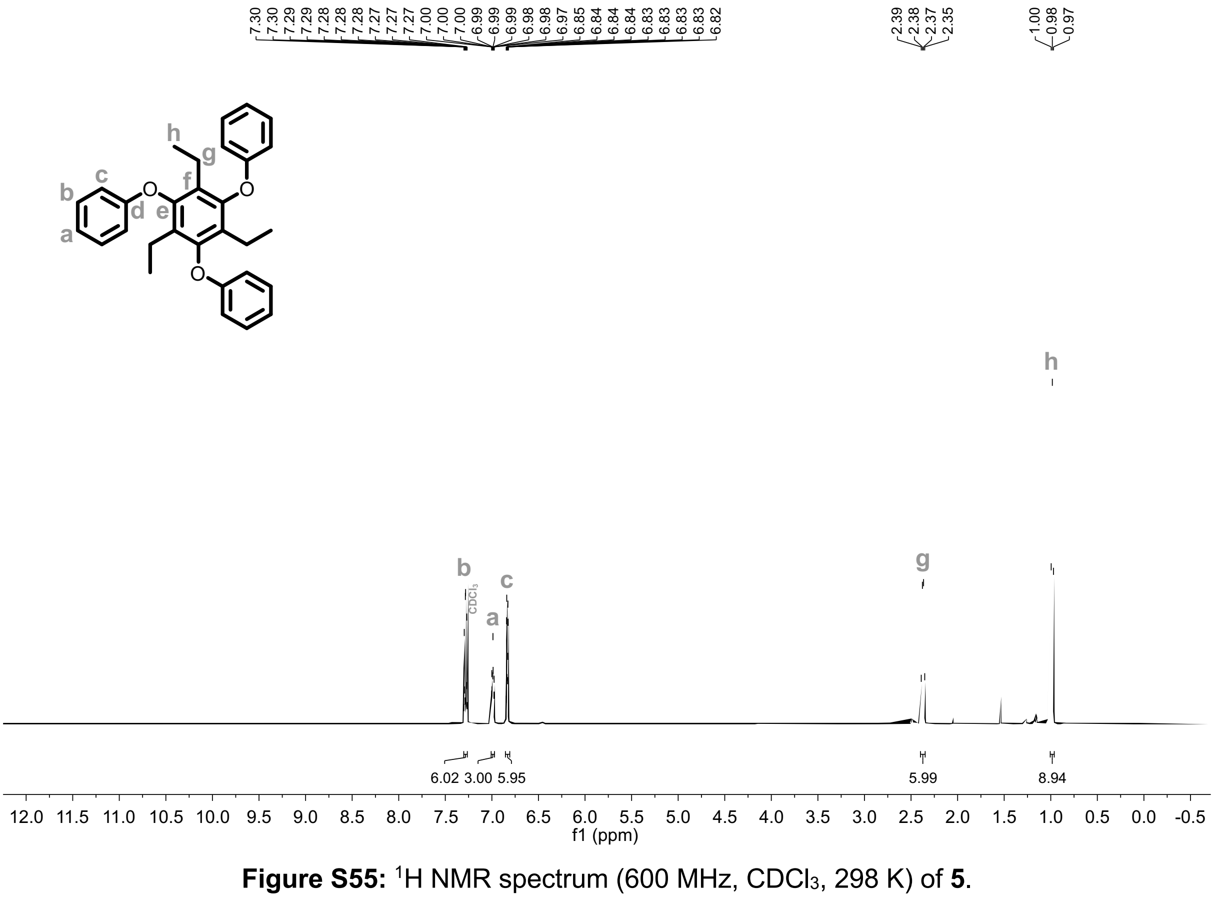

Figure S55: ^{1}H NMR spectrum (600 MHz, $CDCl_3$, 298 K) of **5**.

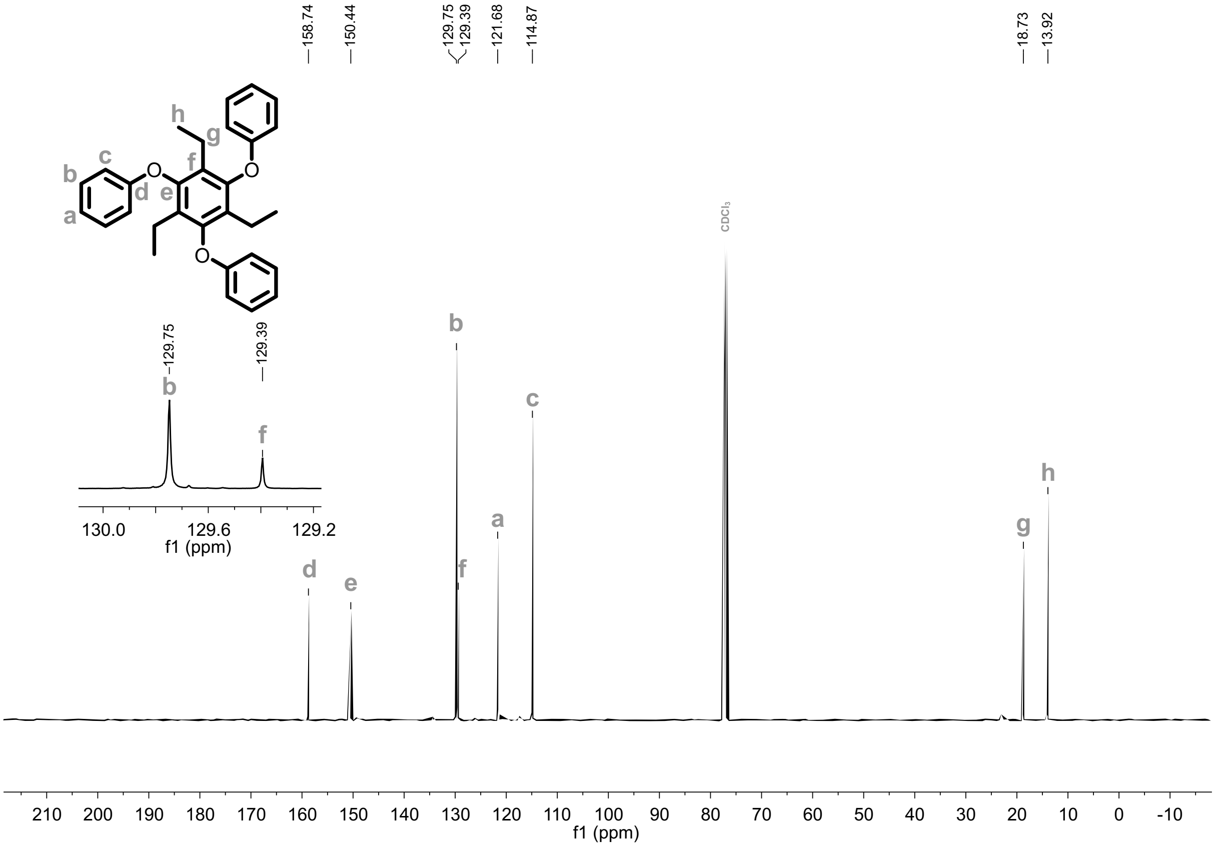

Figure S56: $^{13}C\{^{1}H\}$ NMR spectrum (151 MHz, $CDCl_3$, 298 K) of **5**.

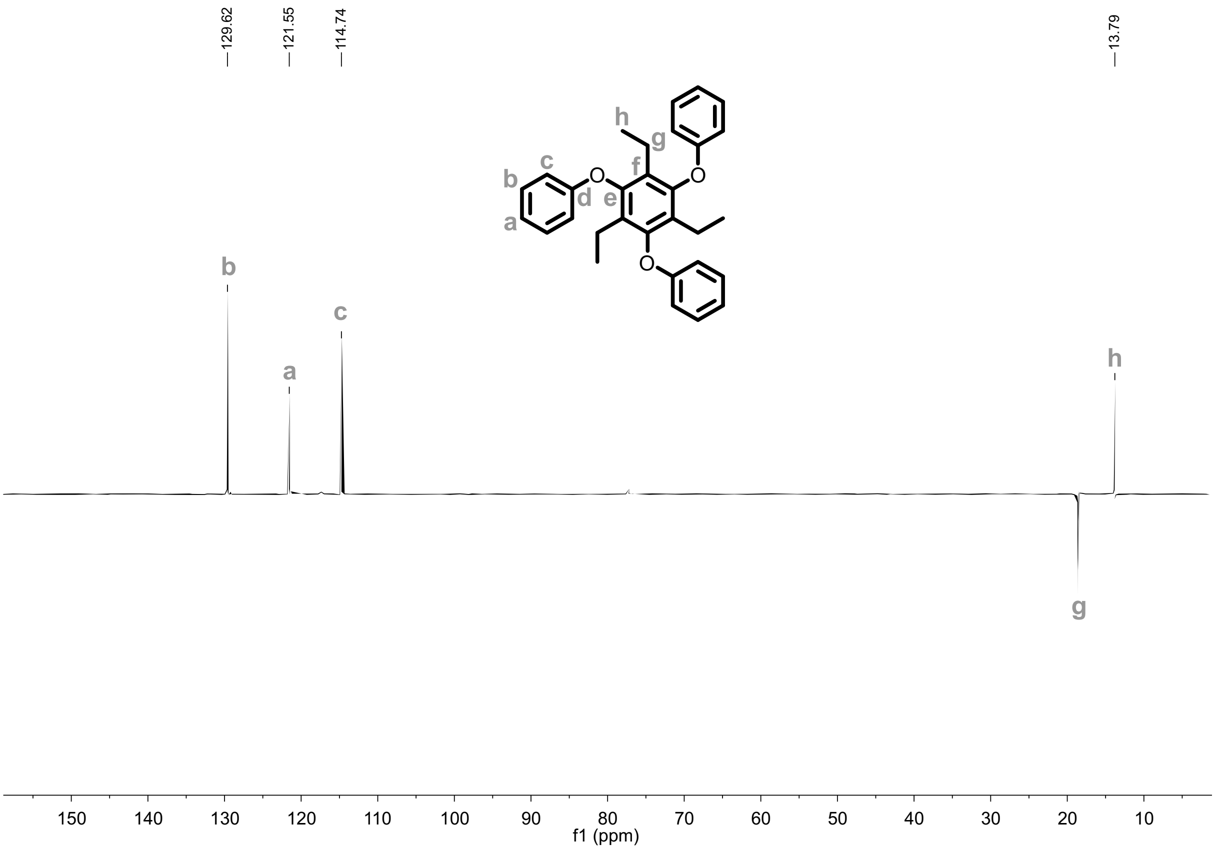

Figure S57: ^{13}C DEPT-135 NMR spectrum (151 MHz, $CDCl_3$, 298 K) of **5**.

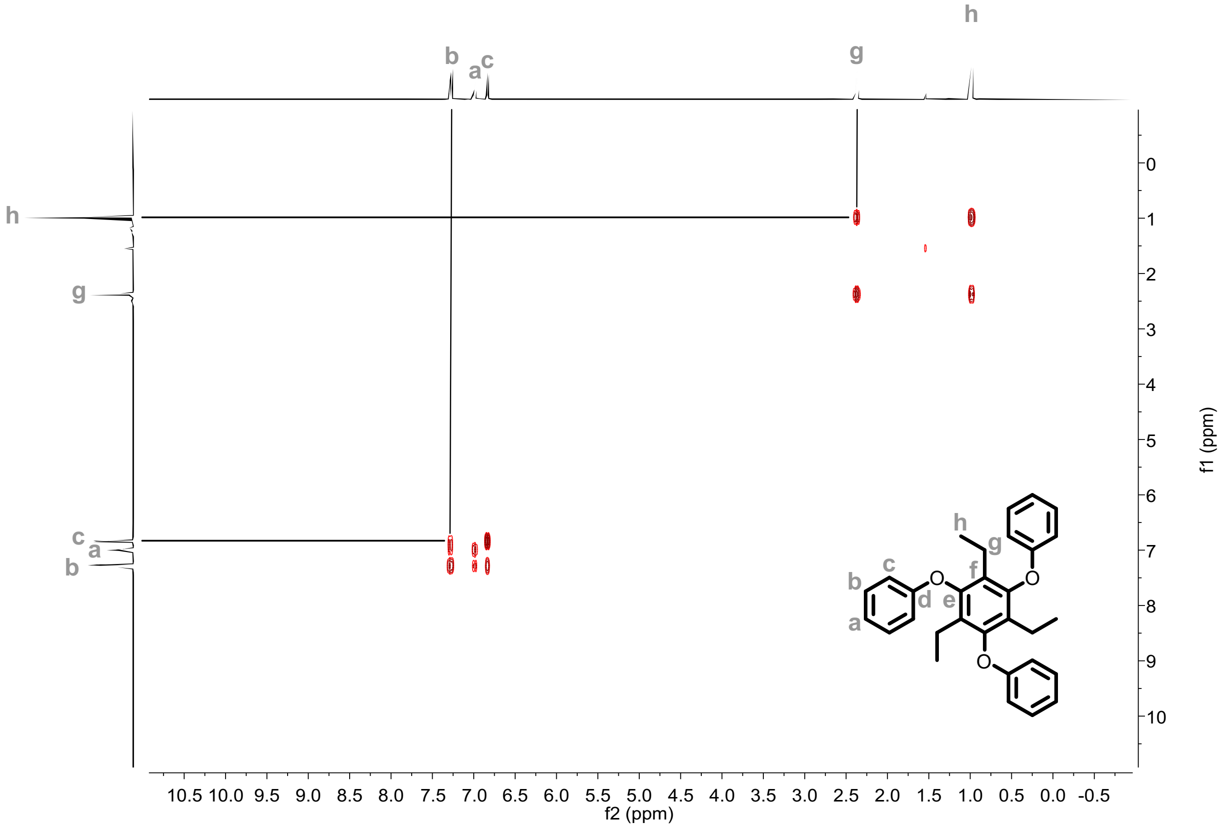

Figure S58: ^{1}H-^{1}H COSY NMR spectrum (600 MHz, $CDCl_3$, 298 K) of **5**.

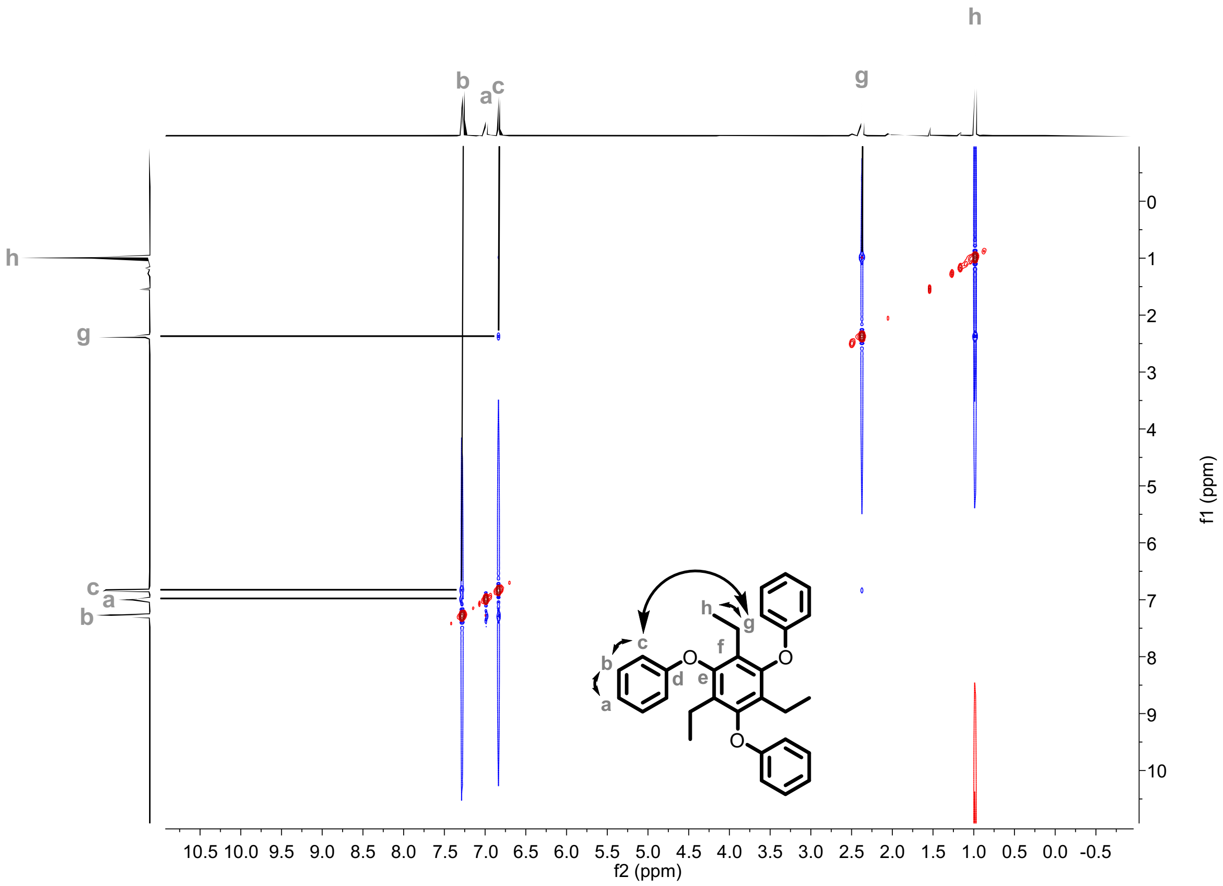

Figure S59: ^{1}H-^{1}H NOESY NMR spectrum (600 MHz, $CDCl_3$, 298 K) of **5**.

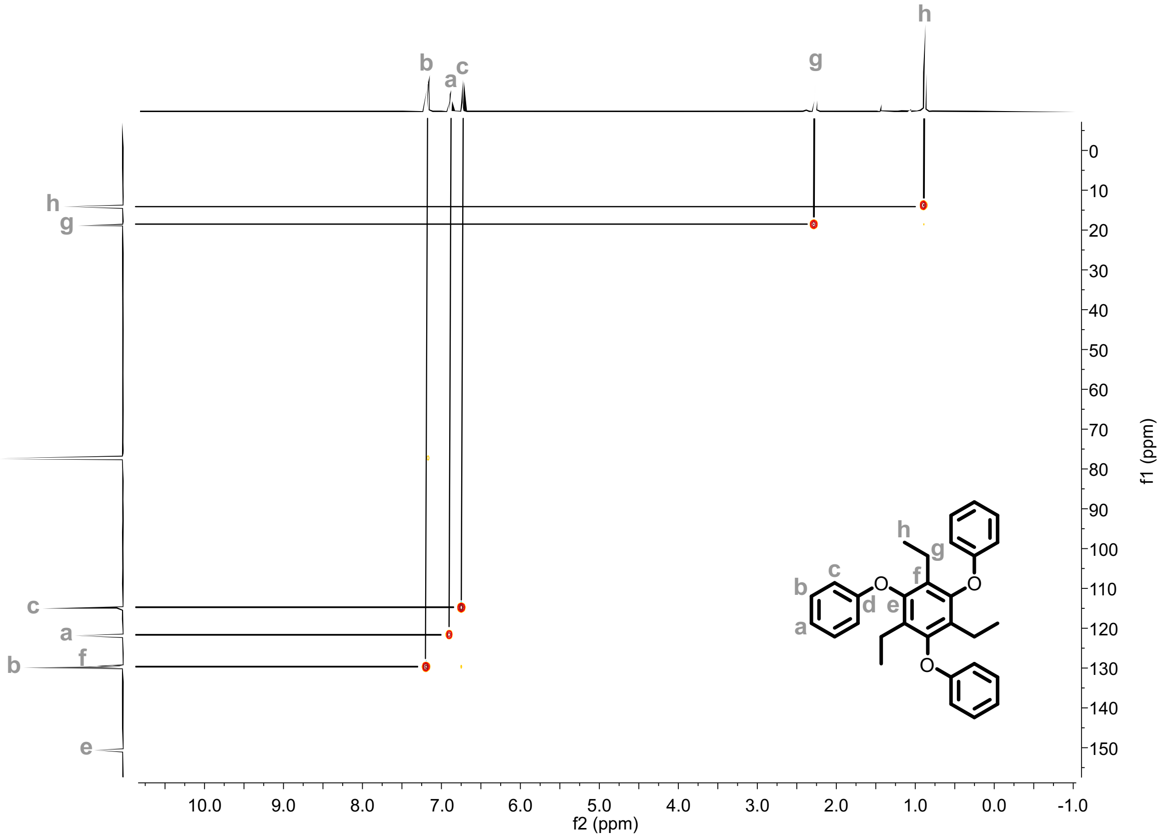

Figure S60: ^{1}H-^{13}C HSQC NMR spectrum (151 MHz, $CDCl_3$, 298 K) of **5**.

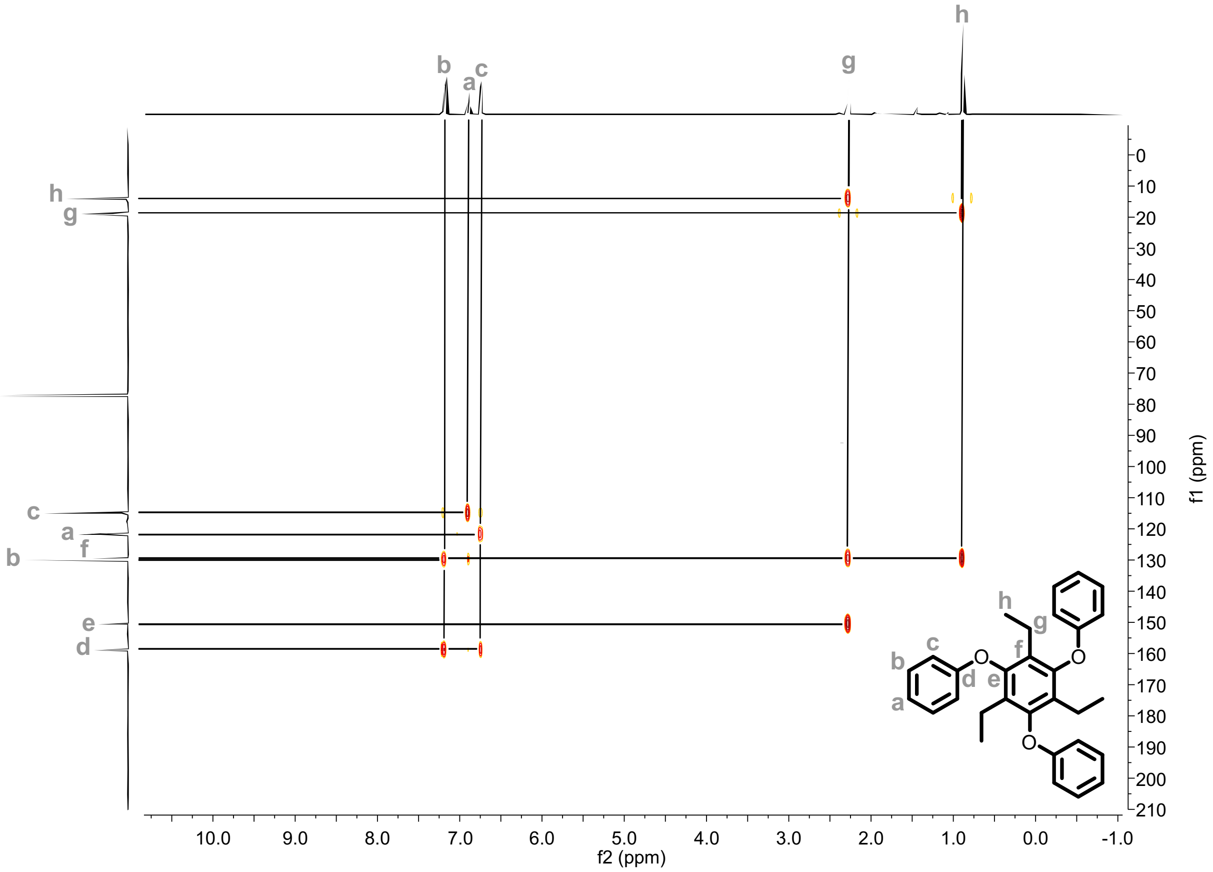

Figure S61: ^{1}H-^{13}C HMBC NMR spectrum (151 MHz, $CDCl_3$, 298 K) of **5**.

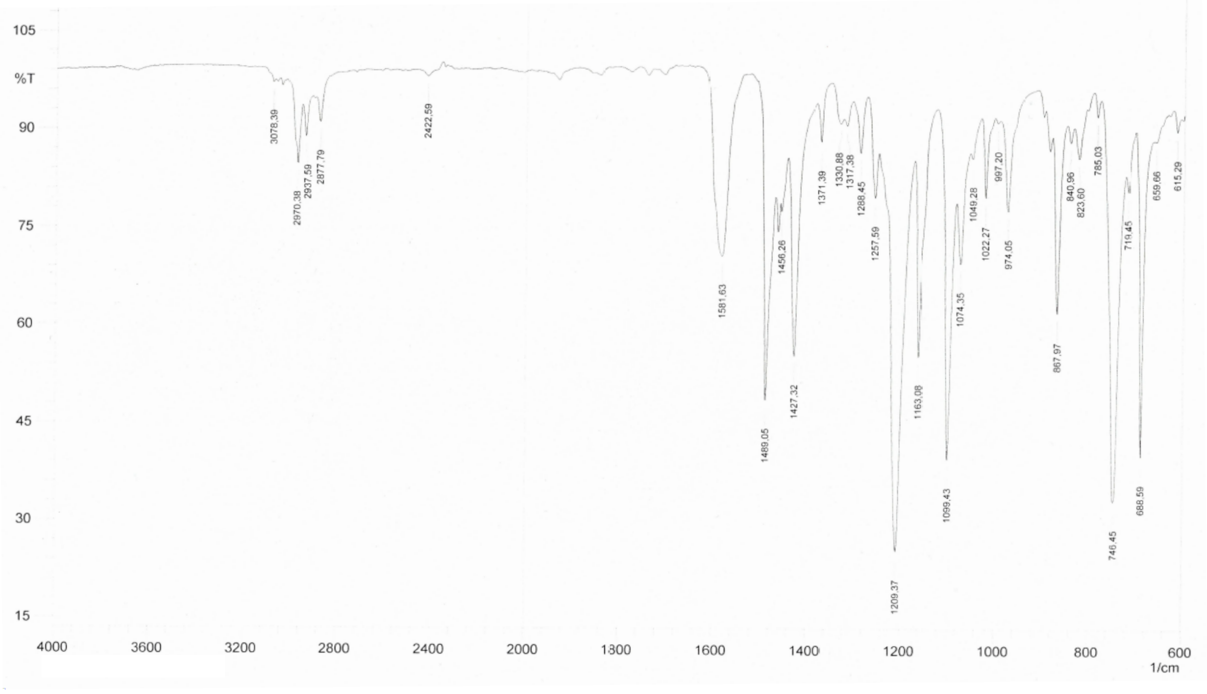

Figure S62: IR spectrum of **5**.

Acquisition Parameter

Source Type	ESI	Ion Polarity	Positive	Set Nebulizer	0.3 bar
Focus	Not active	Set Capillary	3500 V	Set Dry Heater	200 °C
Scan Begin	50 m/z	Set End Plate Offset	-500 V	Set Dry Gas	3.5 l/min
Scan End	1600 m/z	Set Collision Cell RF	500.0 Vpp	Set Divert Valve	Source

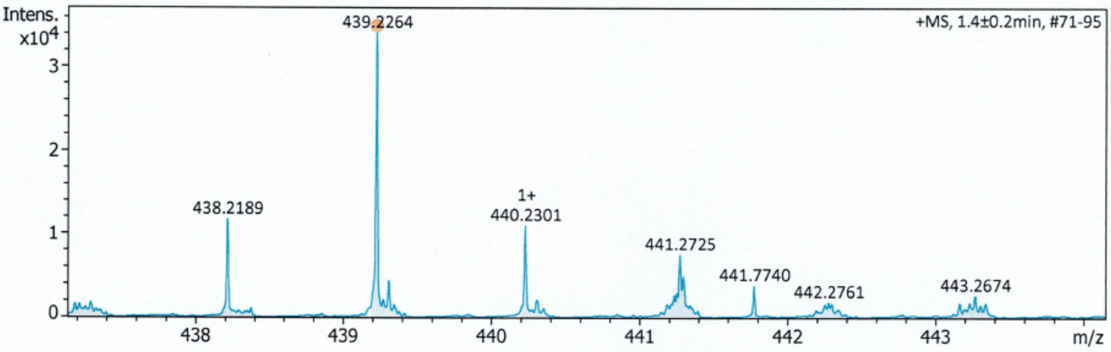

Meas. m/z	#	Ion Formula	m/z	err [ppm]	Mean err [ppm]	rdb (neutral)	N-Rule	e⁻ Conf	mSigma	Std I	Std Mean m/z	Std I VarNorm	Std m/z Diff	Std Comb Dev
439.2264	1	C30H31O3	439.2268	0.9	0.3	16.0	ok	even	11.8	17.3	n.a.	n.a.	n.a.	n.a.

Figure S63: HRMS (ESI) spectrum of **5**.

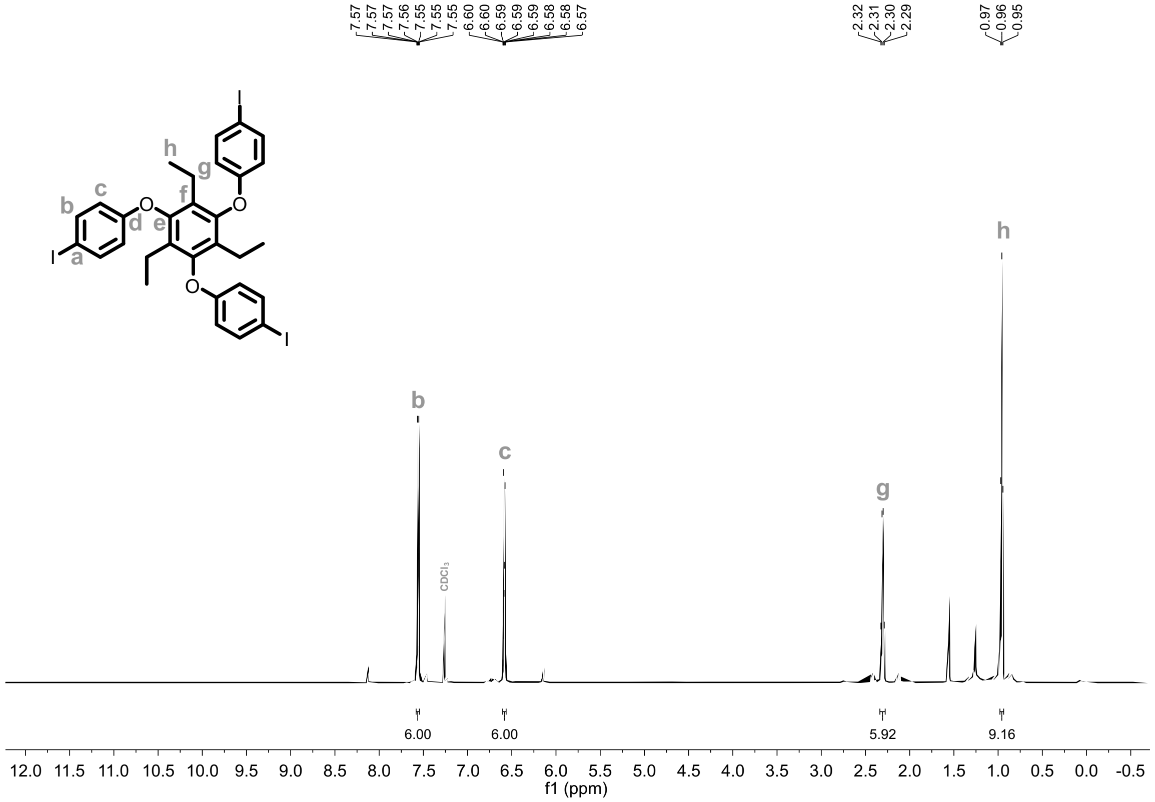

Figure S64: ^{1}H NMR spectrum (600 MHz, $CDCl_3$, 298 K) of **6**.

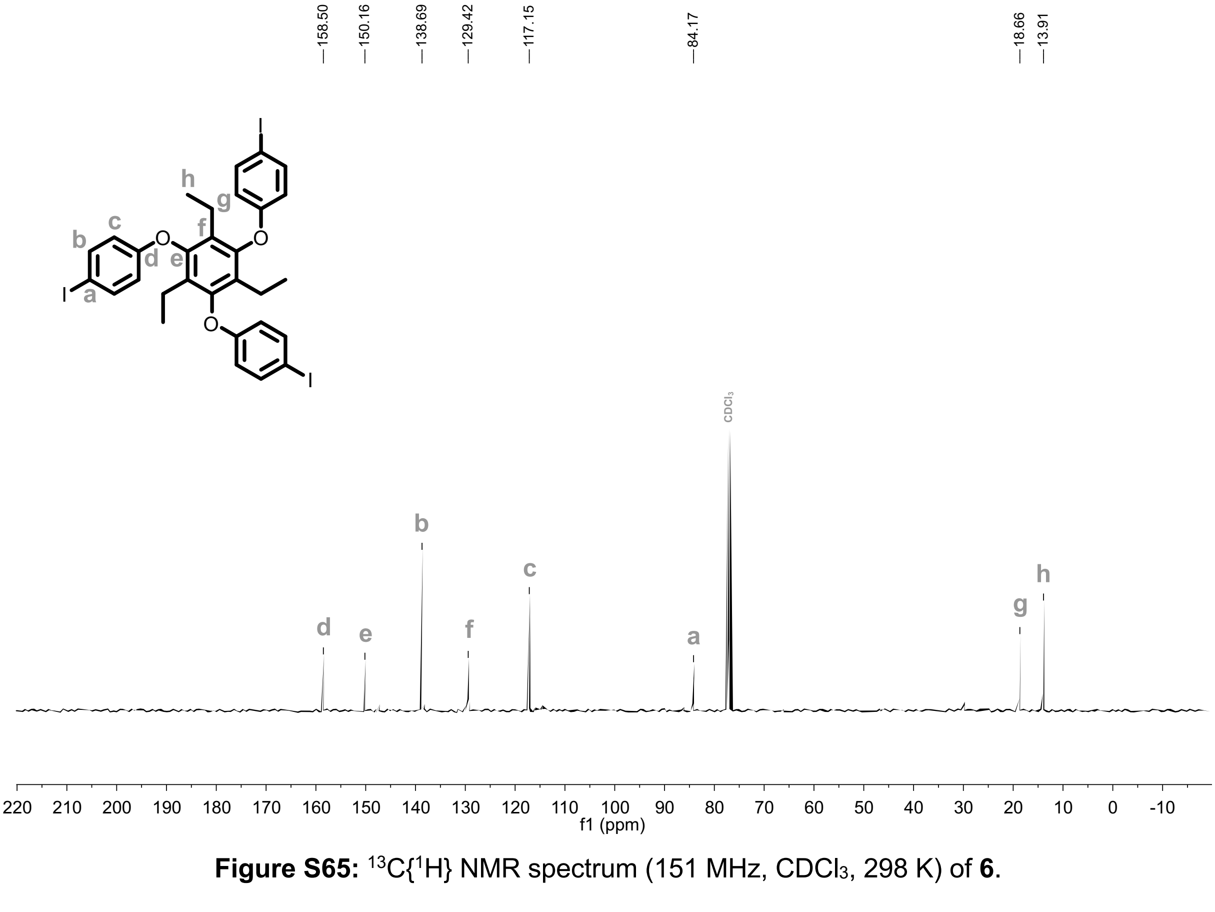

Figure S65: $^{13}C\{^{1}H\}$ NMR spectrum (151 MHz, $CDCl_3$, 298 K) of **6**.

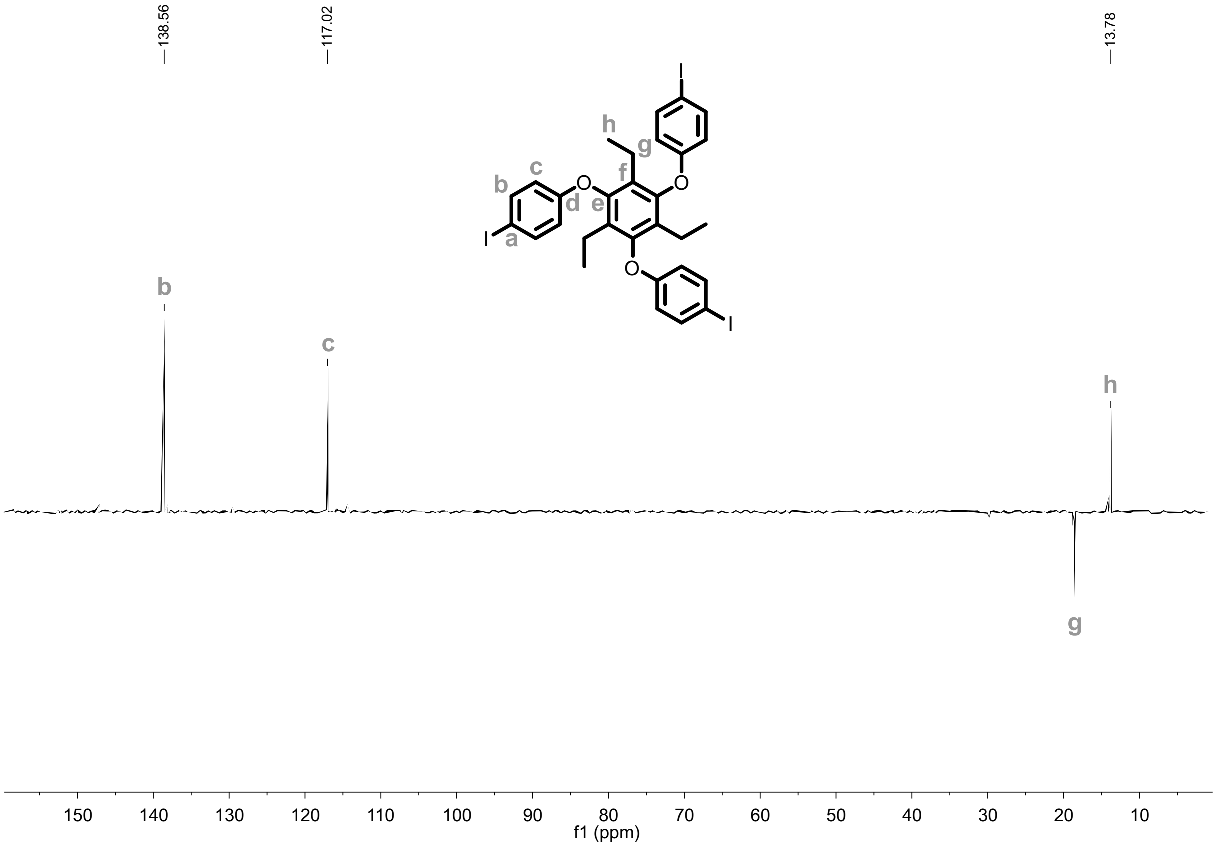

Figure S66: ^{13}C DEPT-135 NMR spectrum (151 MHz, $CDCl_3$, 298 K) of **6**.

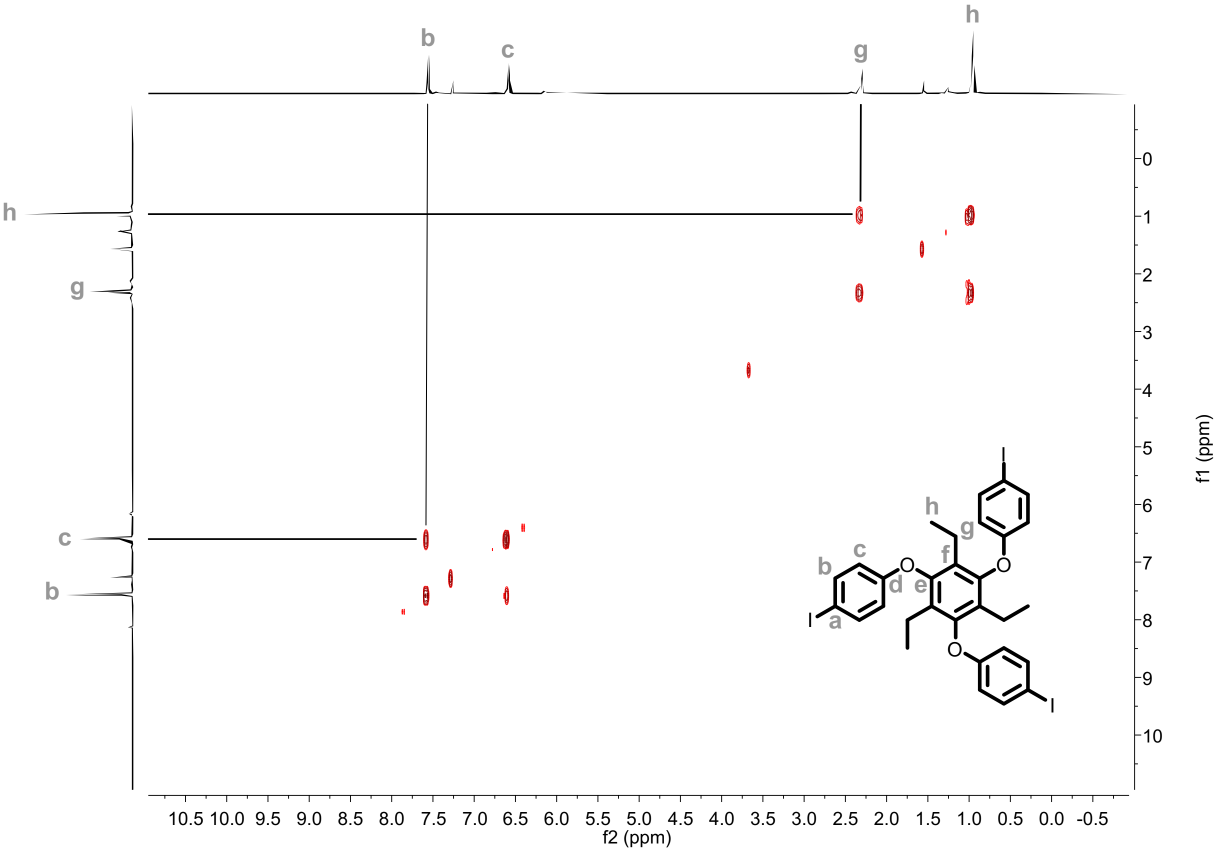

Figure S67: ^{1}H-^{1}H COSY NMR spectrum (600 MHz, $CDCl_3$, 298 K) of **6**.

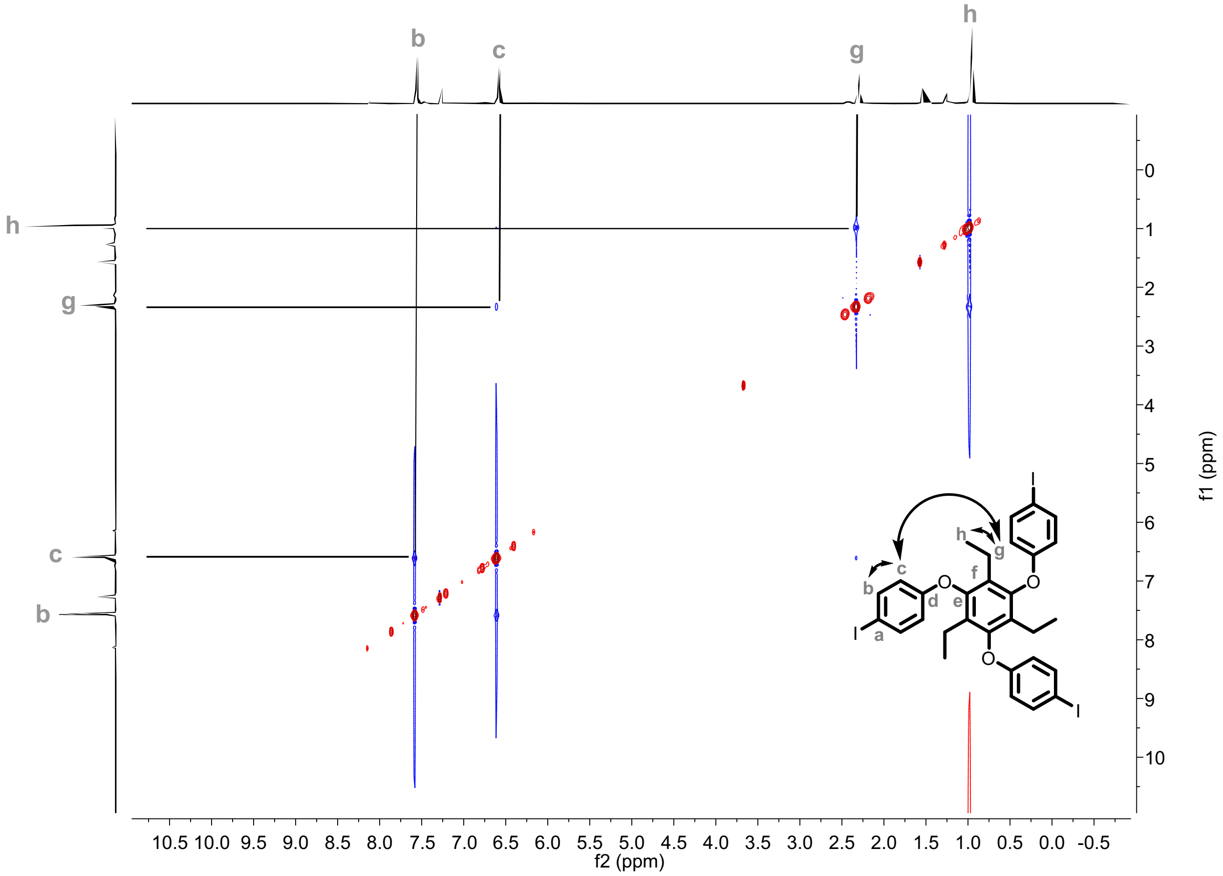

Figure S68: ^{1}H-^{1}H NOESY NMR spectrum (600 MHz, $CDCl_3$, 298 K) of **6**.

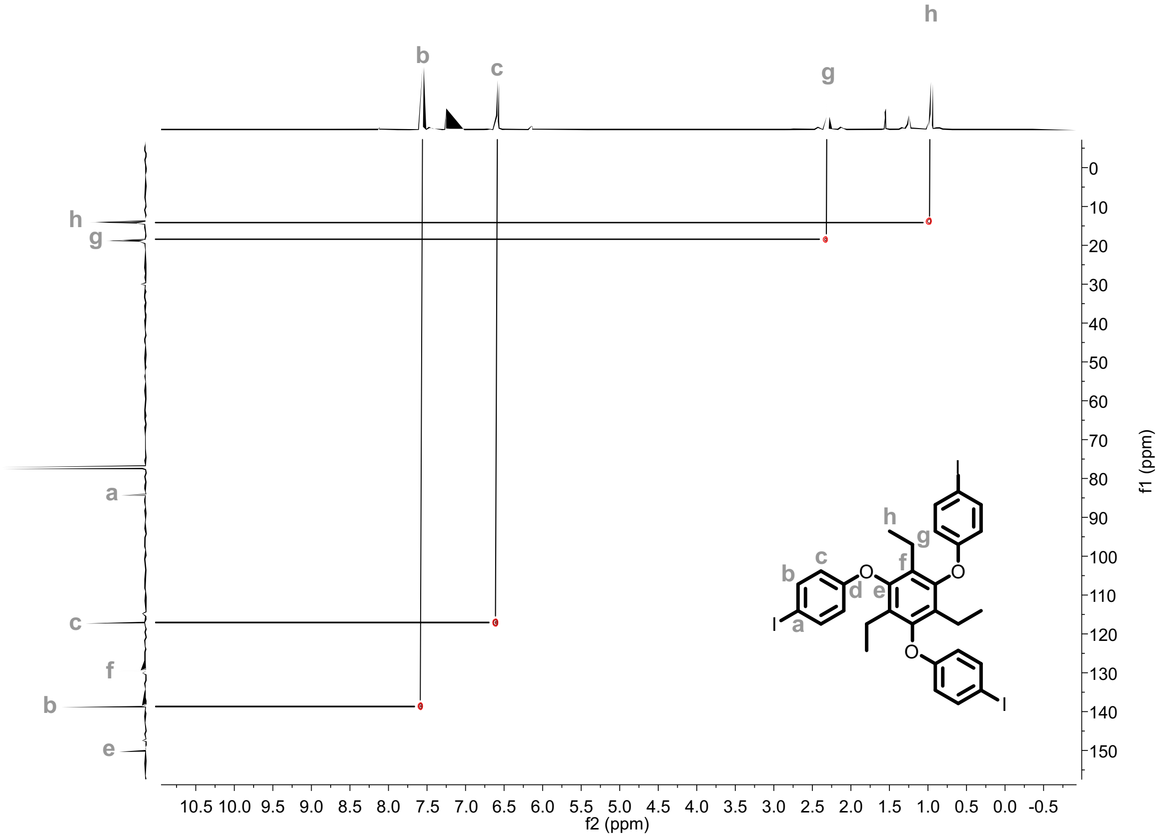

Figure S69: ^{1}H-^{13}C HSQC NMR spectrum (151 MHz, $CDCl_3$, 298 K) of **6**.

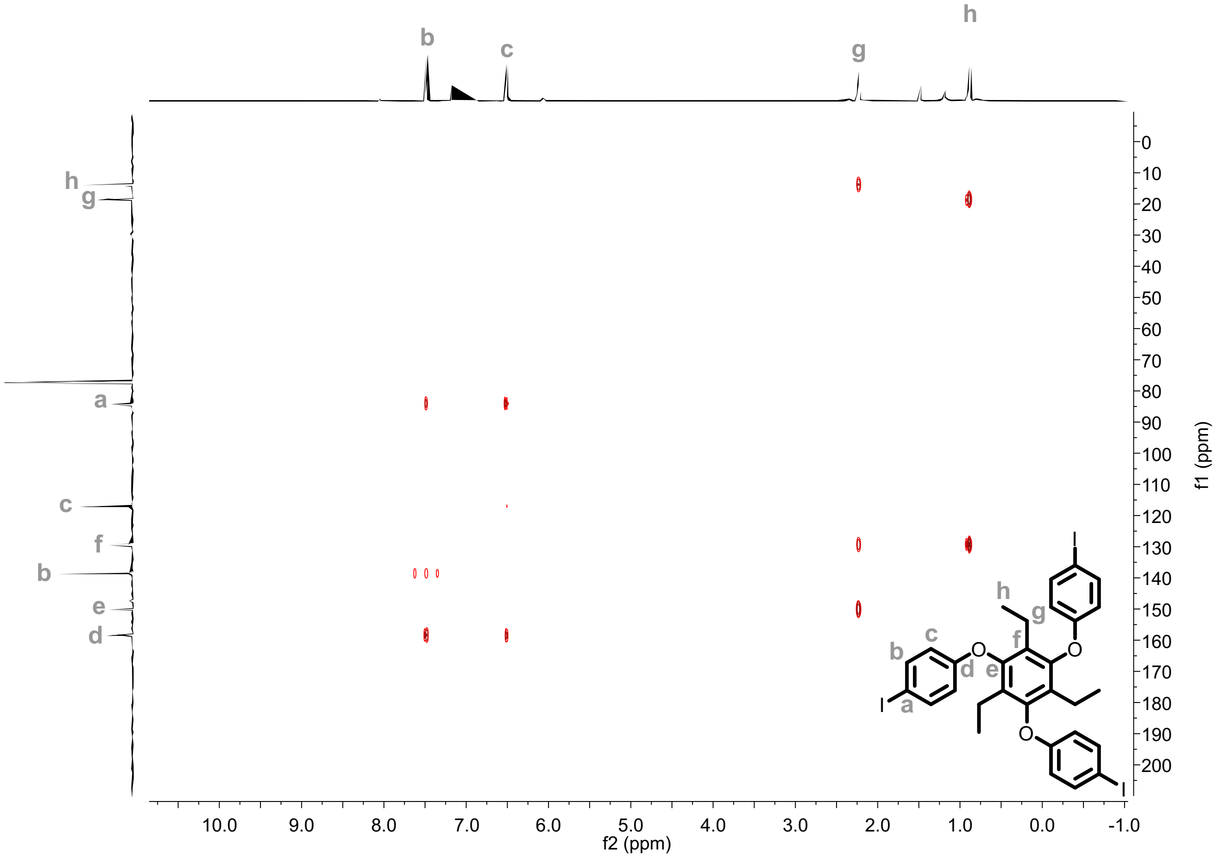

Figure S70: ^{1}H-^{13}C HMBC NMR spectrum (151 MHz, $CDCl_3$, 298 K) of **6**.

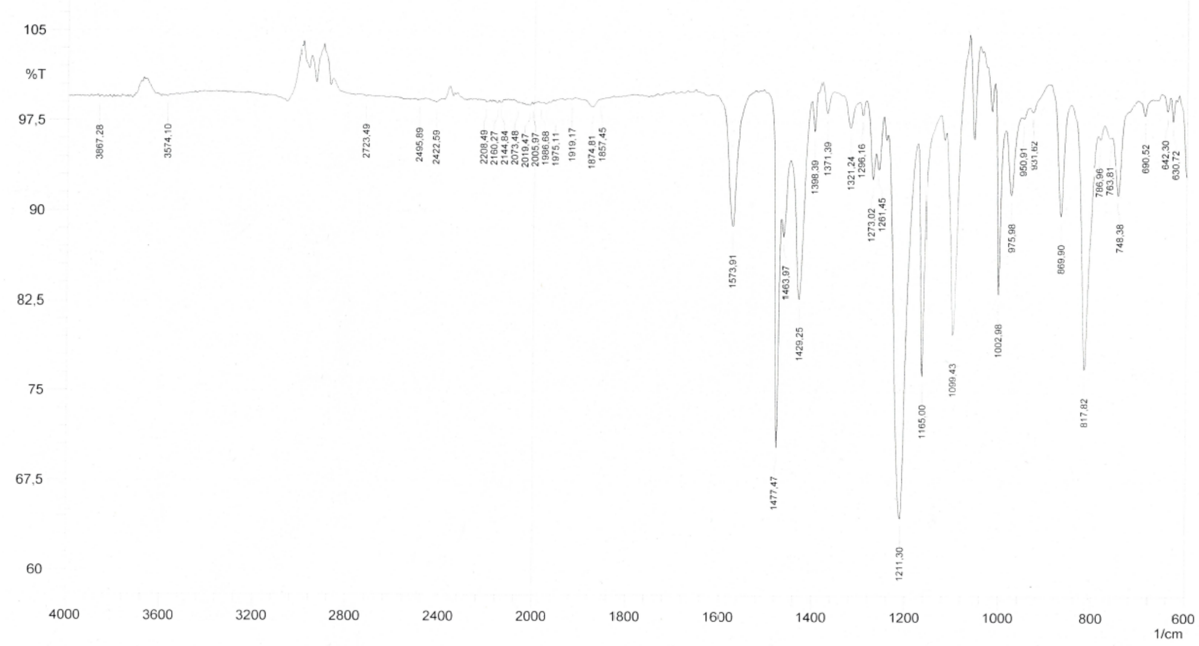

Figure S71: IR spectrum of **6**.

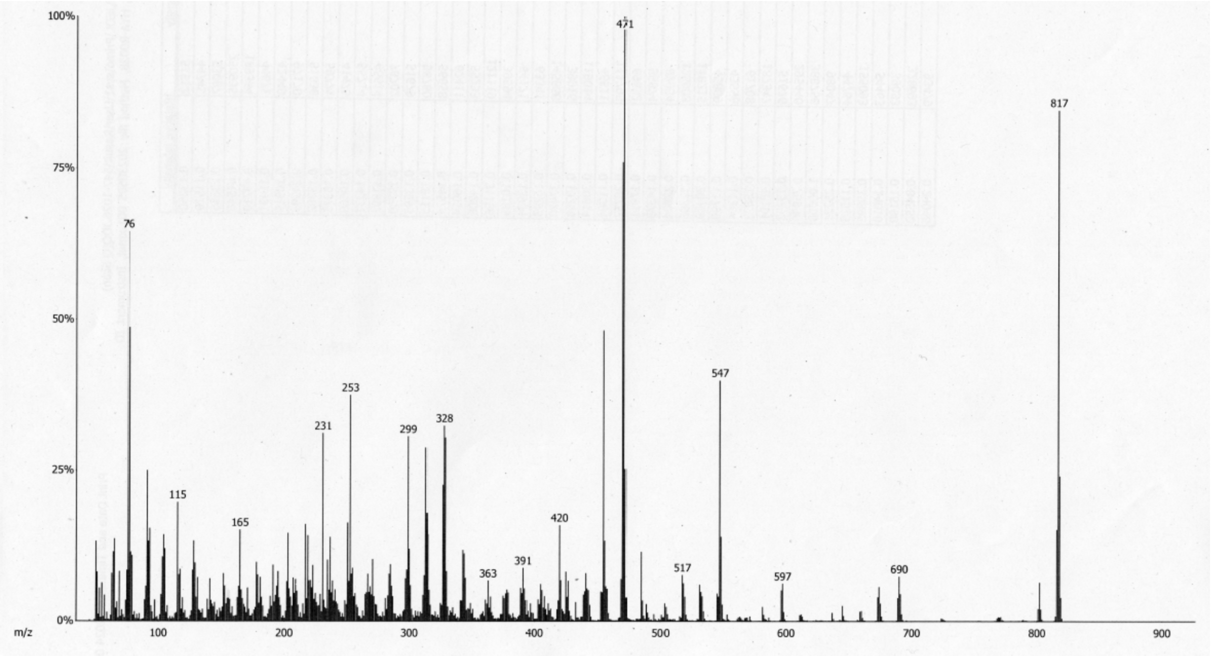

Figure S72: MS (EI) spectrum of **6**.

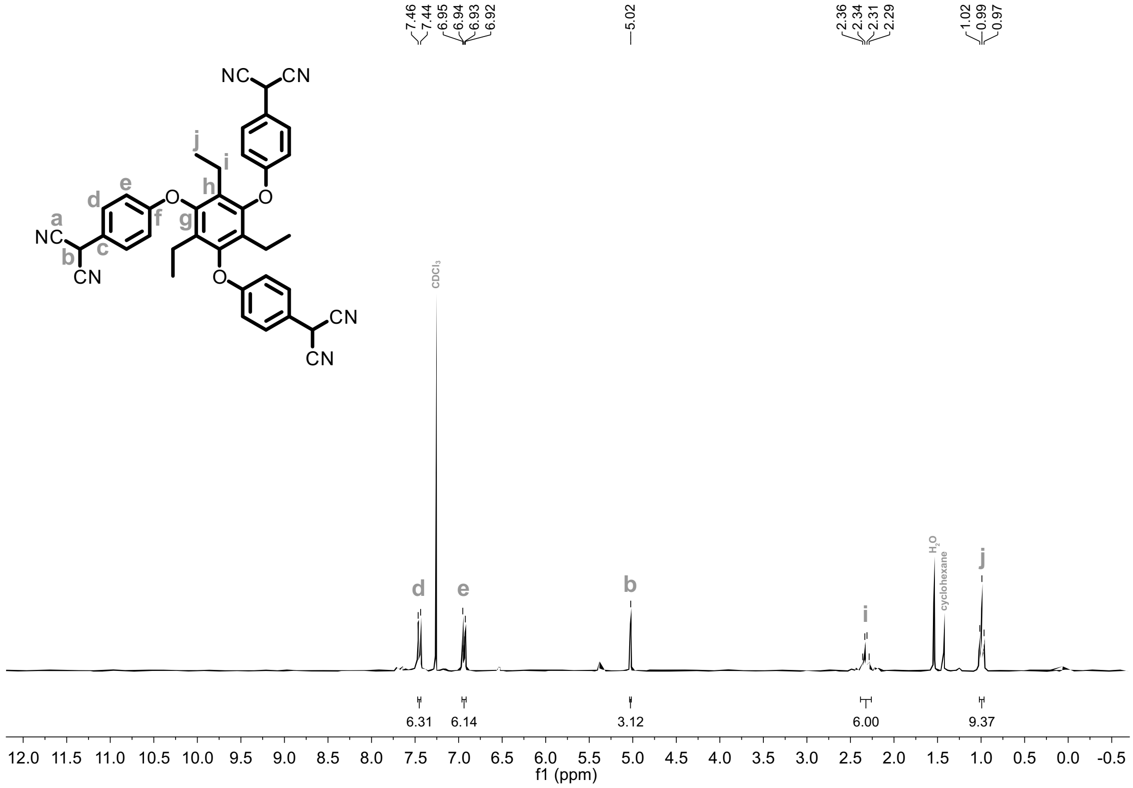

Figure S73: ^{1}H NMR spectrum (600 MHz, $CDCl_3$, 298 K) of **O**.

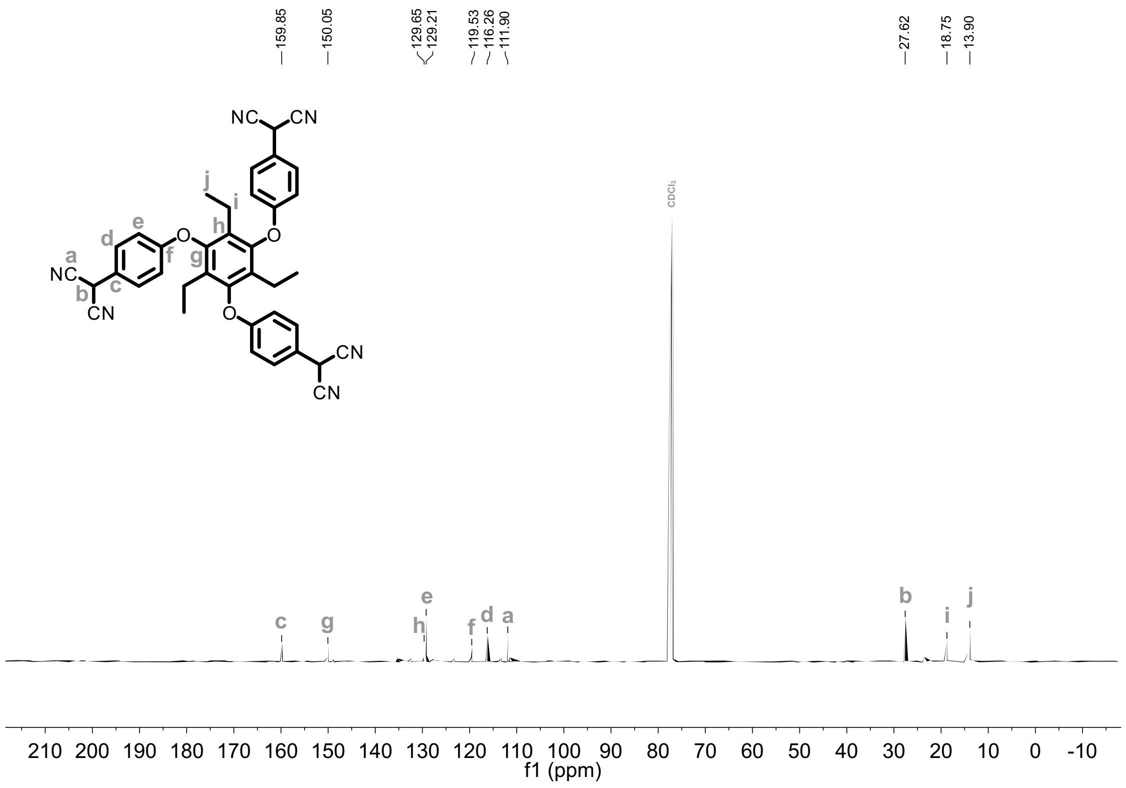

Figure S74: $^{13}C\{^{1}H\}$ NMR spectrum (151 MHz, $CDCl_3$, 298 K) of **O**.

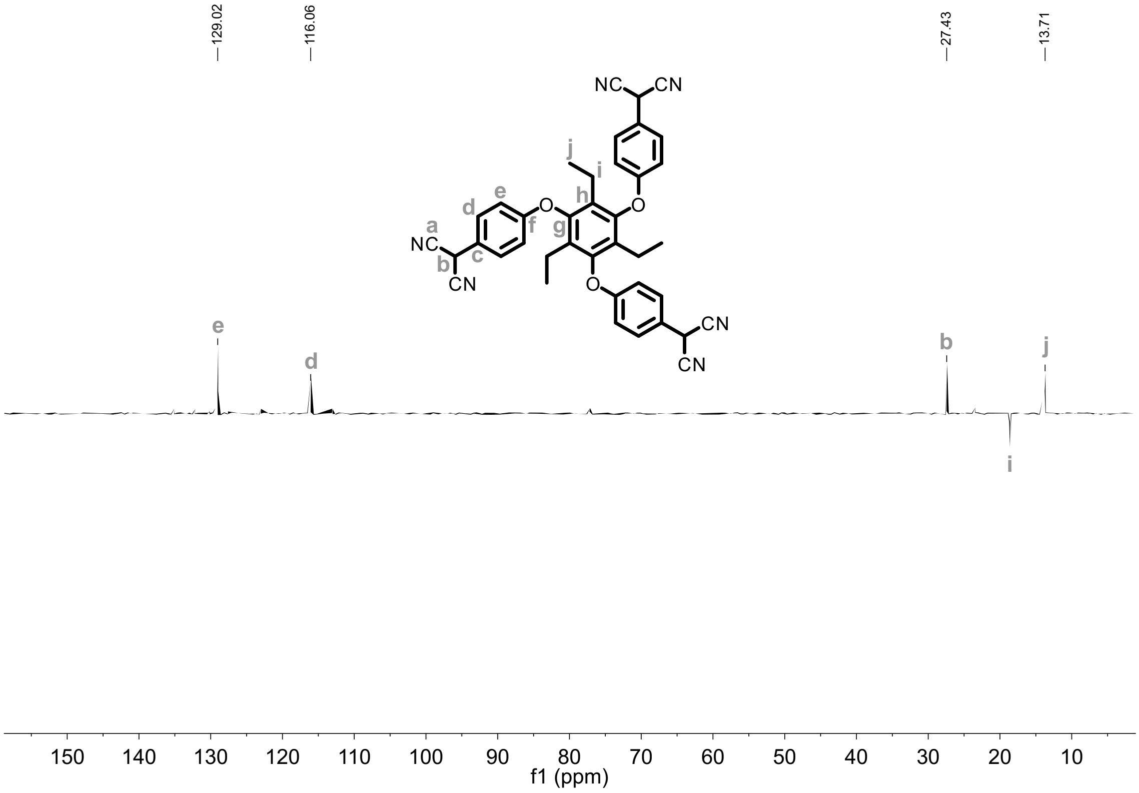

Figure S75: ^{13}C DEPT-135 NMR spectrum (151 MHz, $CDCl_3$, 298 K) of **O**.

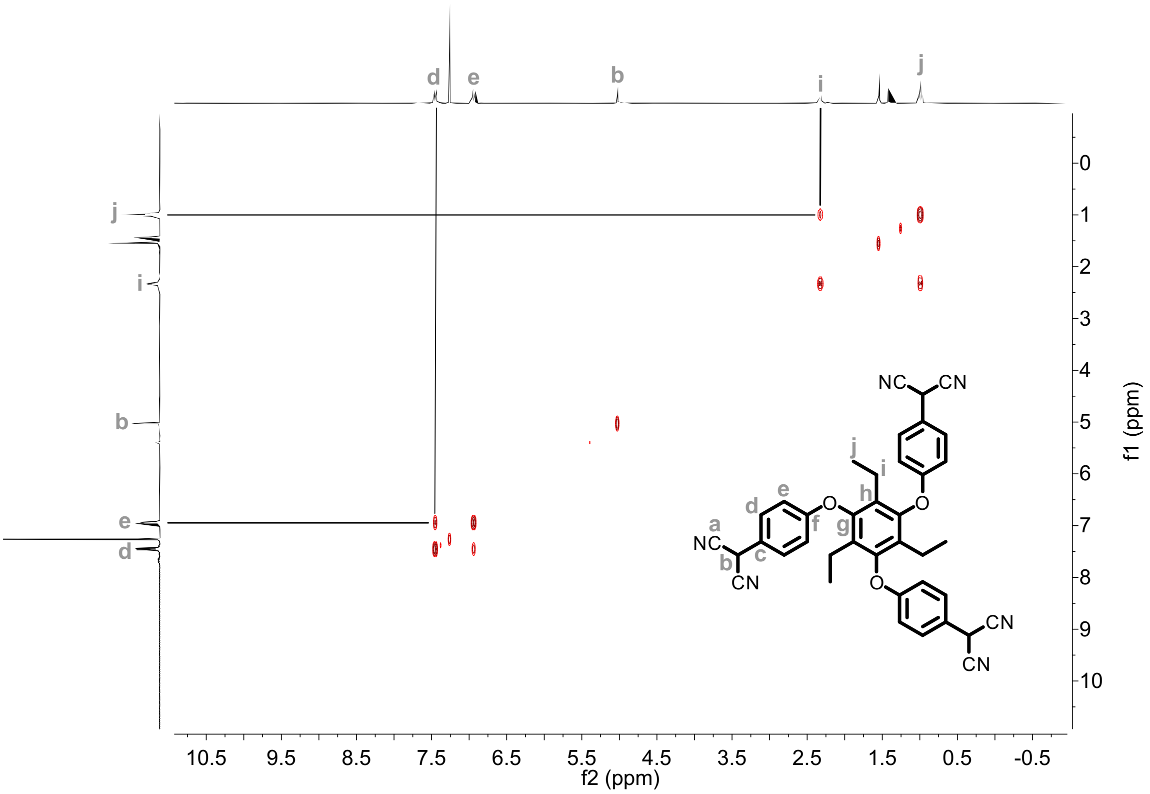

Figure S76: ^{1}H-^{1}H COSY NMR spectrum (600 MHz, $CDCl_3$, 298 K) of **O**.

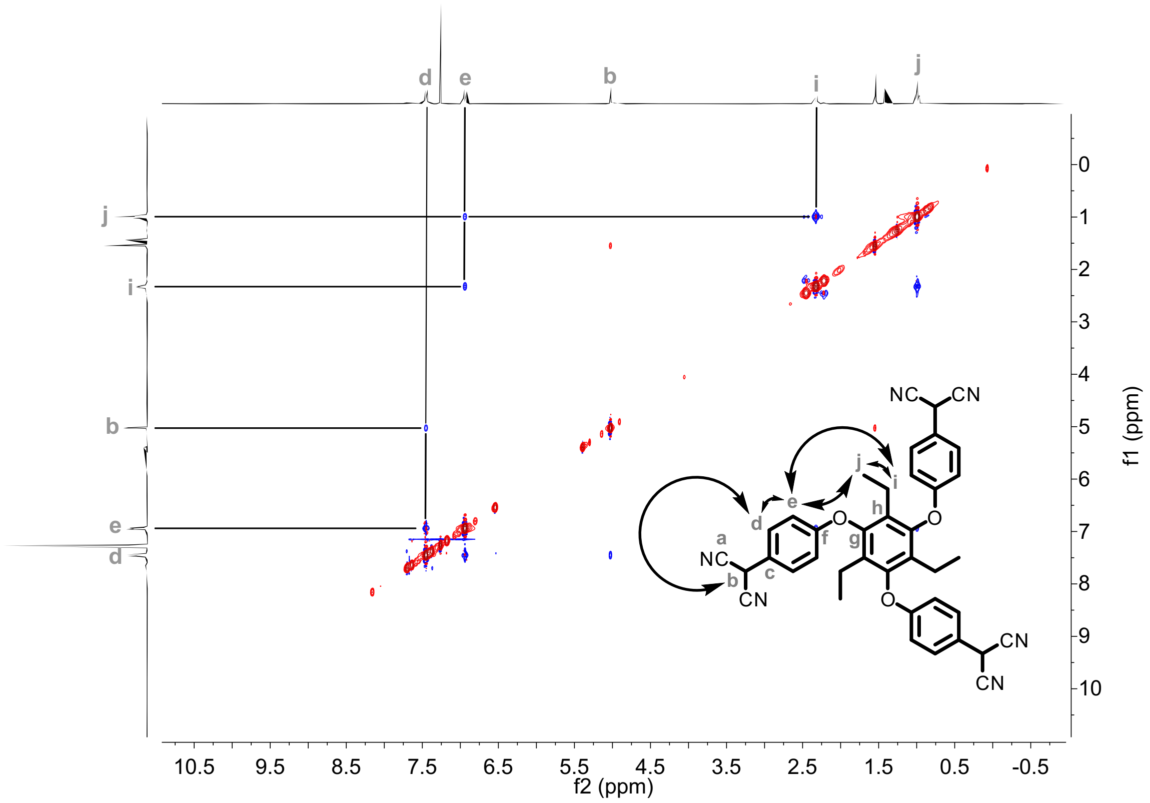

Figure S77: ^{1}H-^{1}H NOESY NMR spectrum (600 MHz, $CDCl_3$, 298 K) of **O**.

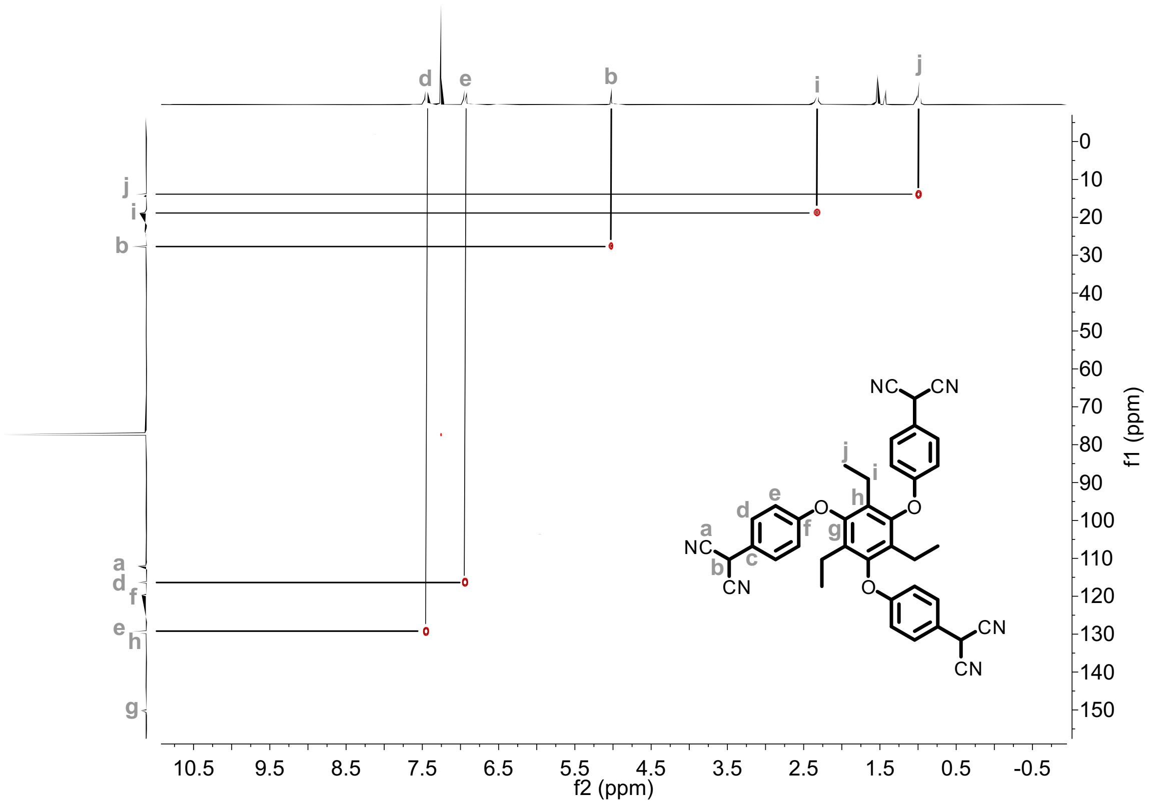

Figure S78: ¹H-¹³C HSQC NMR spectrum (151 MHz, $CDCl_3$, 298 K) of **O**.

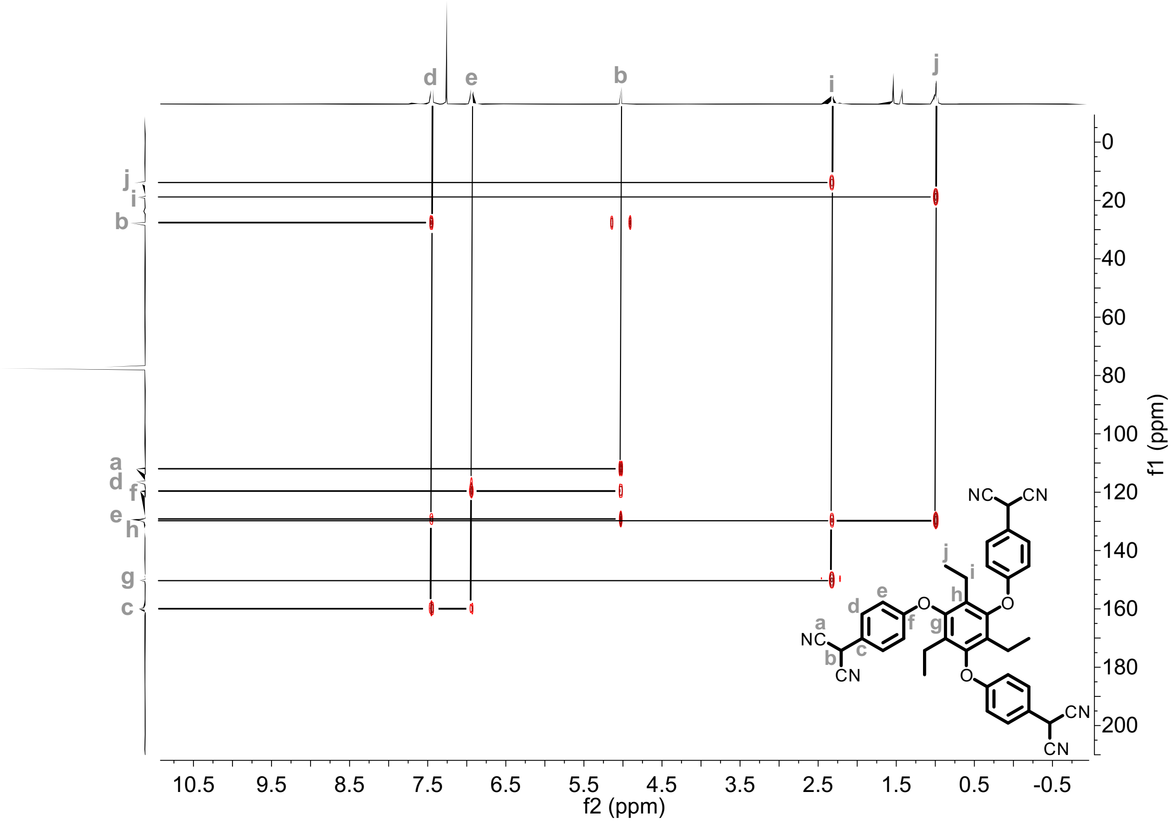

Figure S79: ¹H-¹³C HMBC NMR spectrum (151 MHz, $CDCl_3$, 298 K) of **O**.

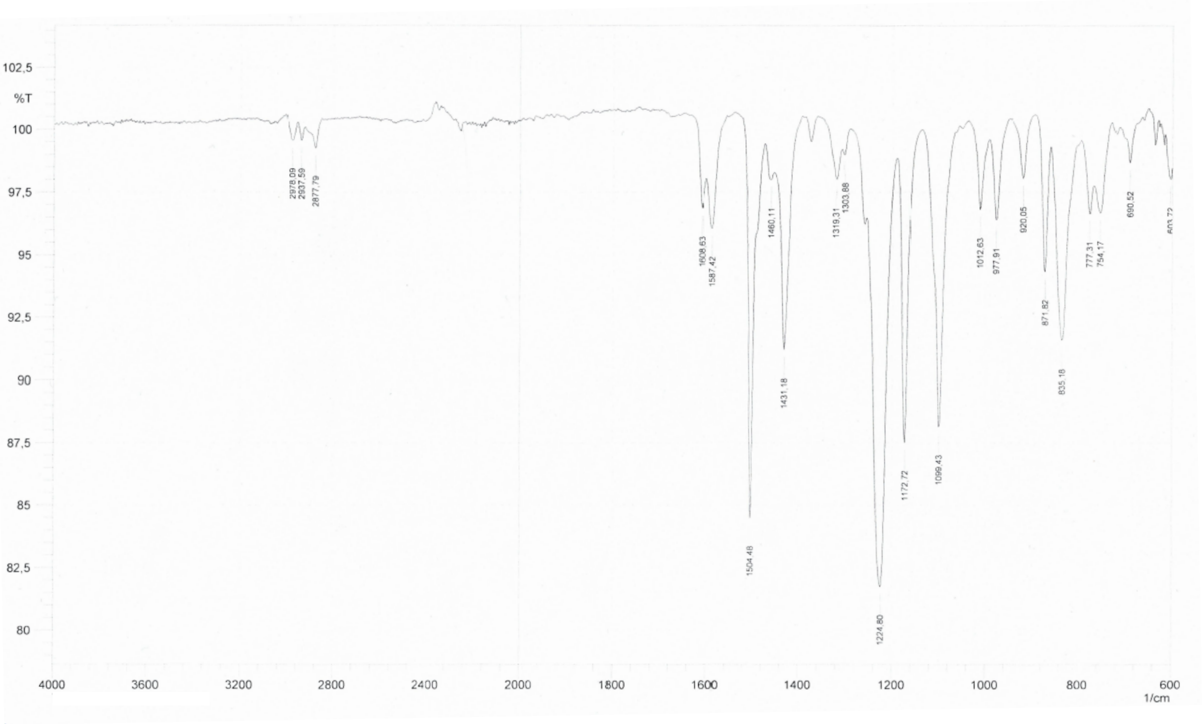

Figure S80: IR spectrum of **O**.

Acquisition Parameter

Source Type	ESI	Ion Polarity	Positive	Set Nebulizer	0.3 bar
Focus	Not active	Set Capillary	3500 V	Set Dry Heater	200 °C
Scan Begin	50 m/z	Set End Plate Offset	-500 V	Set Dry Gas	3.5 l/min
Scan End	1600 m/z	Set Collision Cell RF	500.0 Vpp	Set Divert Valve	Source

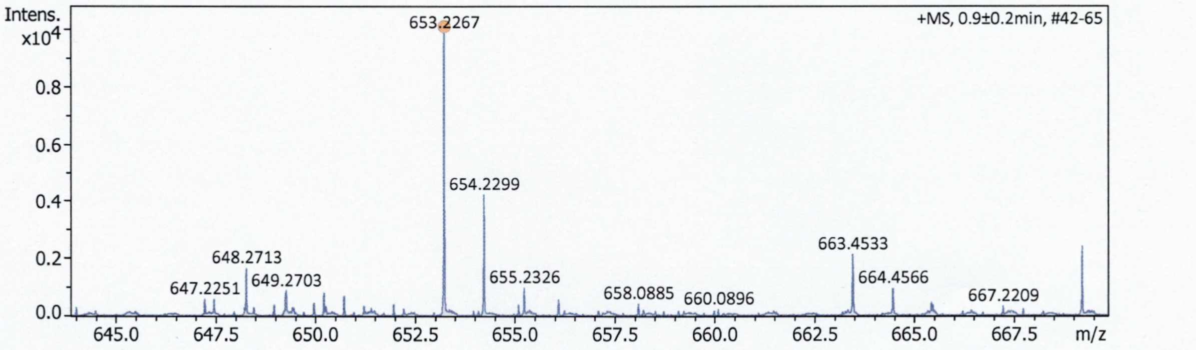

Meas. m/z	#	Ion Formula	m/z	err [ppm]	Mean err [ppm]	rdb (neutral)	N-Rule	e⁻ Conf	mSigma	Std I	Std Mean m/z	Std I VarNorm	Std m/z Diff	Std Comb Dev
653.2267	1	C38H34N2NaO7	653.2258	-1.3	-1.2	23.0	ok	even	2.6	3.3	n.a.	n.a.	n.a.	n.a.
653.2267	2	C39H30N6NaO3	653.2272	0.8	0.7	28.0	ok	even	11.1	13.9	n.a.	n.a.	n.a.	n.a.

Figure S81: HRMS (ESI) spectrum of **O**.

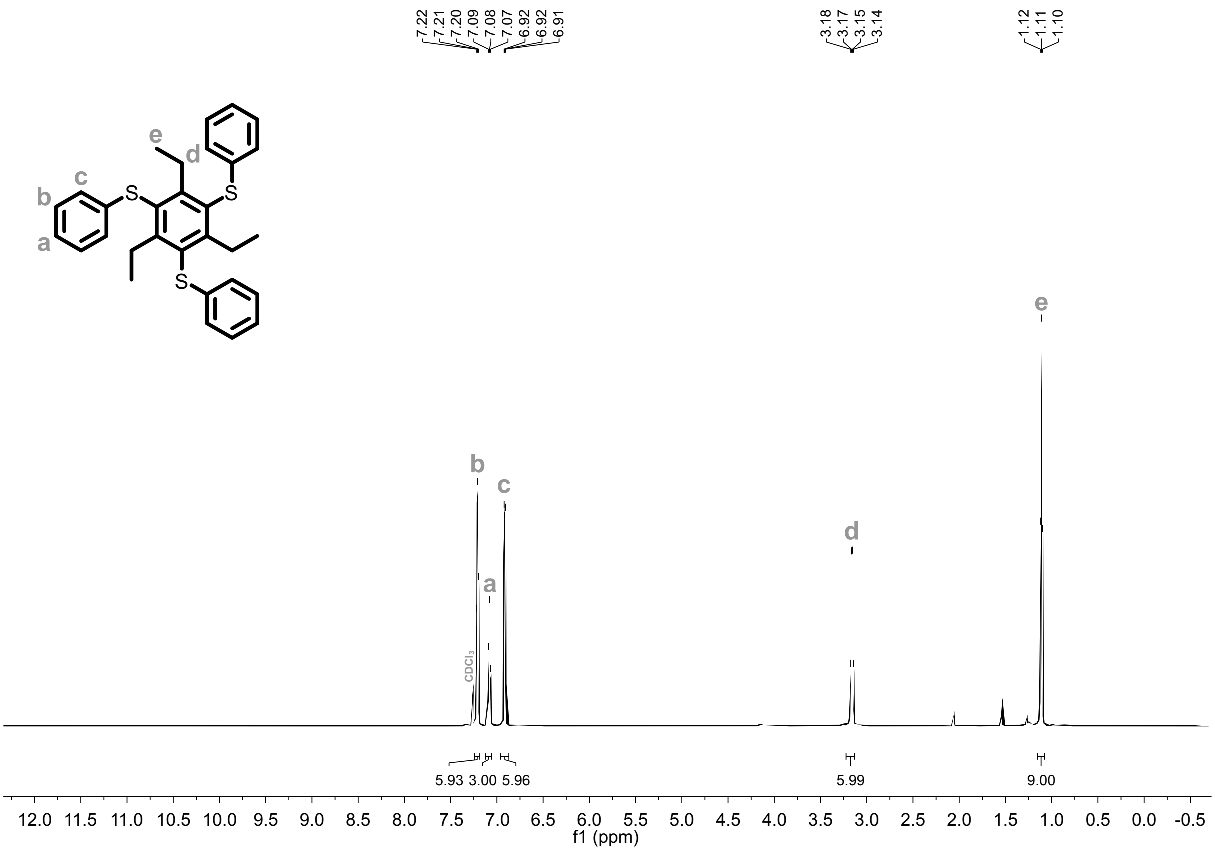

Figure S82: ^{1}H NMR spectrum (600 MHz, $CDCl_3$, 298 K) of **7**.

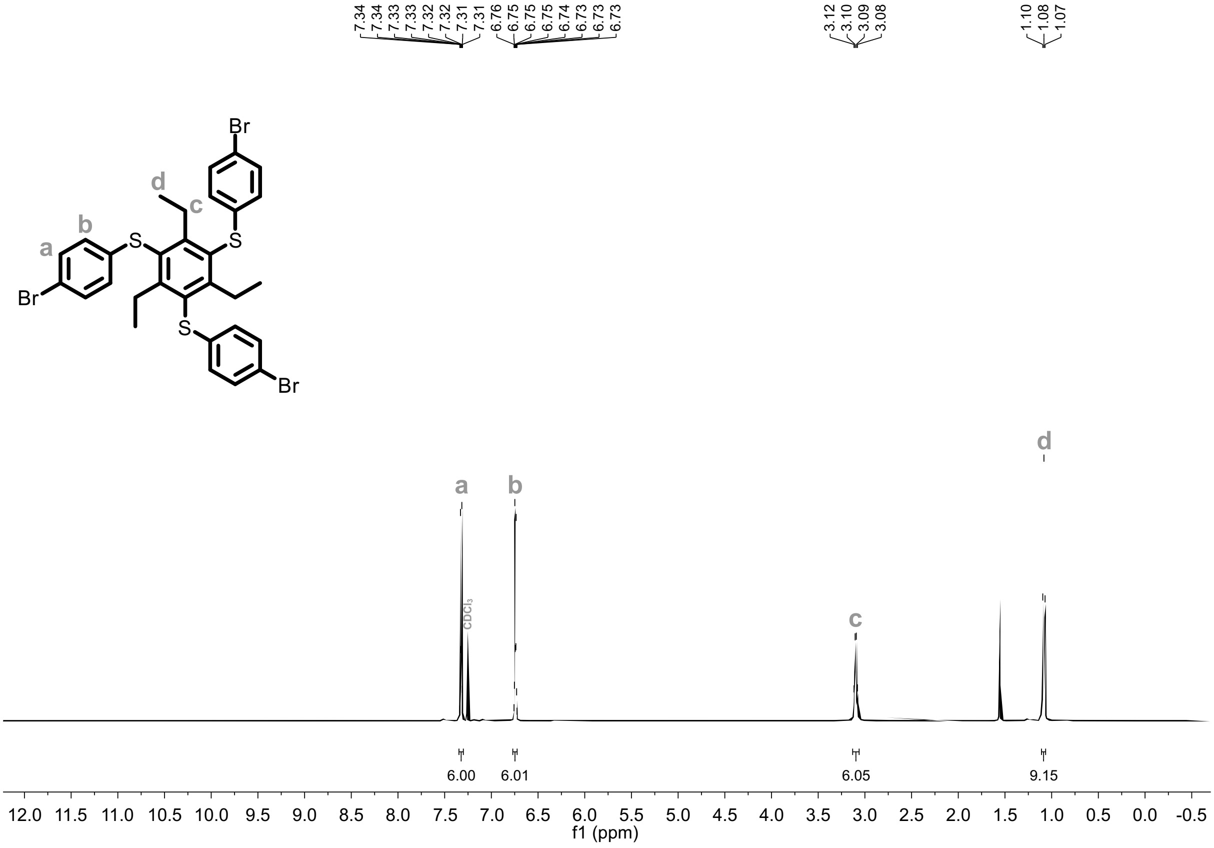

Figure S83: ^{1}H NMR spectrum (600 MHz, $CDCl_3$, 298 K) of **8**.

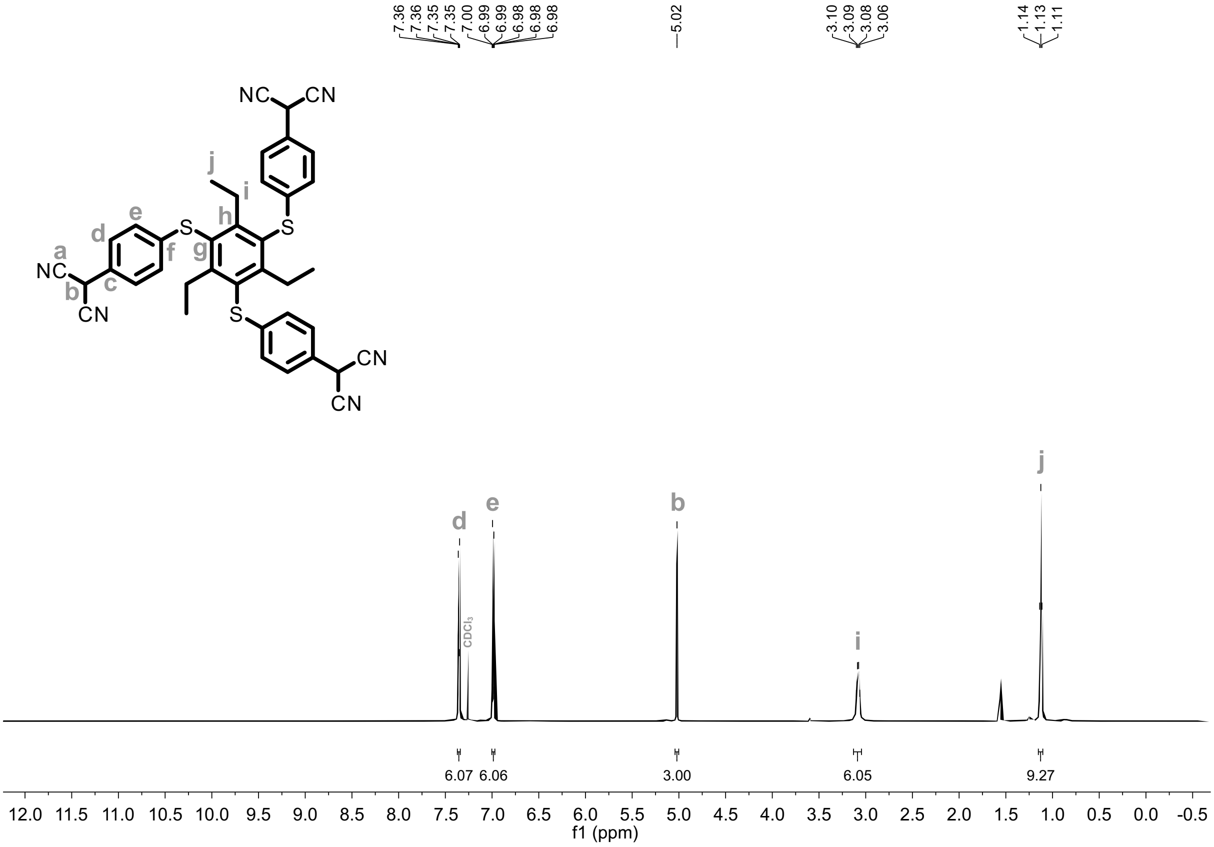

Figure S84: ^{1}H NMR spectrum (600 MHz, $CDCl_3$, 298 K) of **S**.

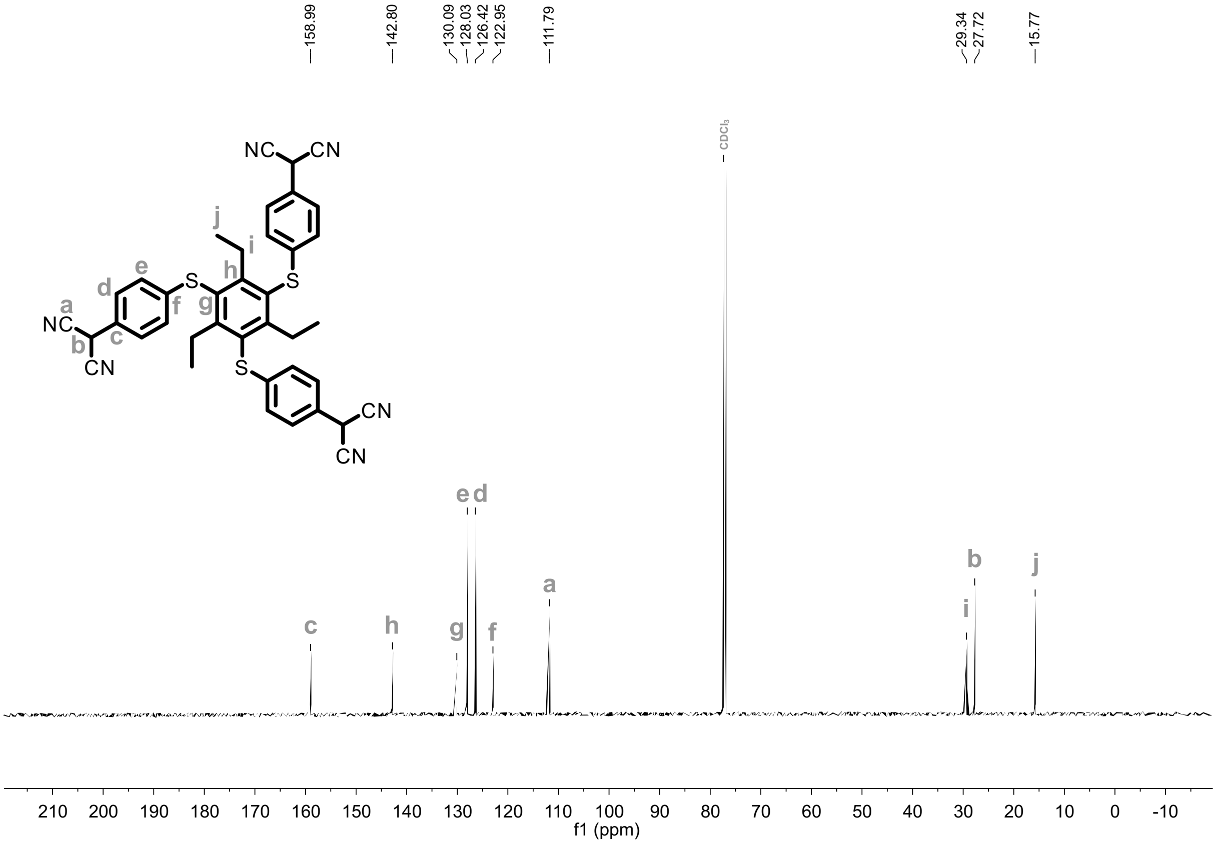

Figure S85: $^{13}C\{^{1}H\}$ NMR spectrum (151 MHz, $CDCl_3$, 298 K) of **S**.

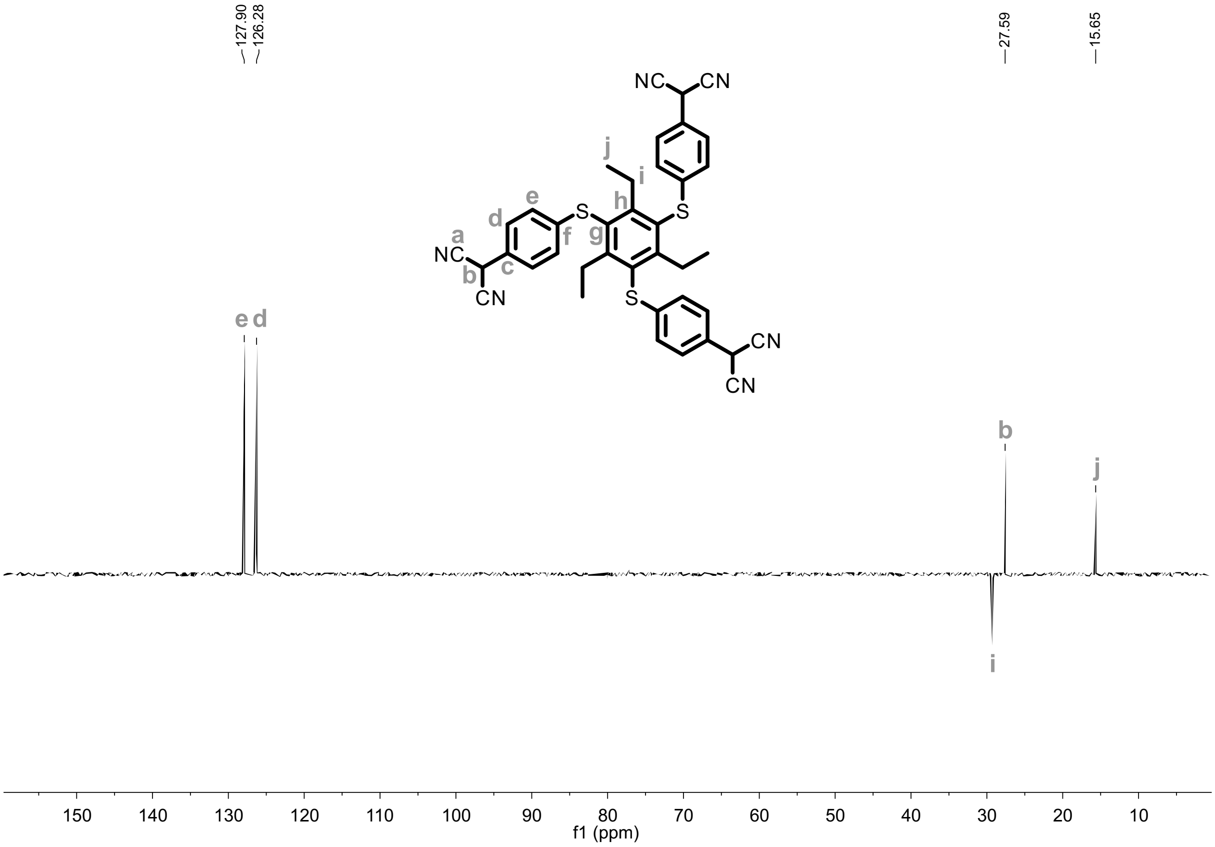

Figure S86: ^{13}C DEPT-135 NMR spectrum (151 MHz, $CDCl_3$, 298 K) of **S**.

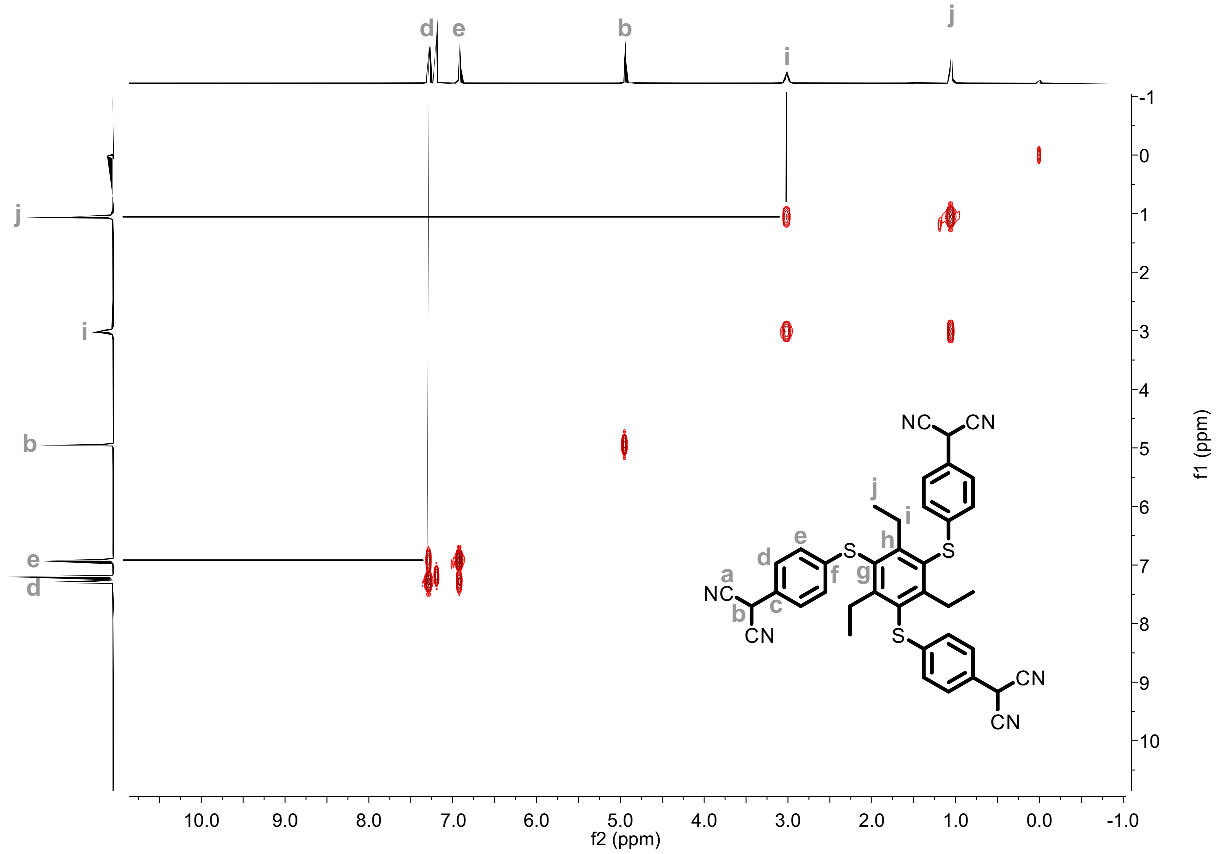

Figure S87: ^{1}H-^{1}H COSY NMR spectrum (600 MHz, $CDCl_3$, 298 K) of **S**.

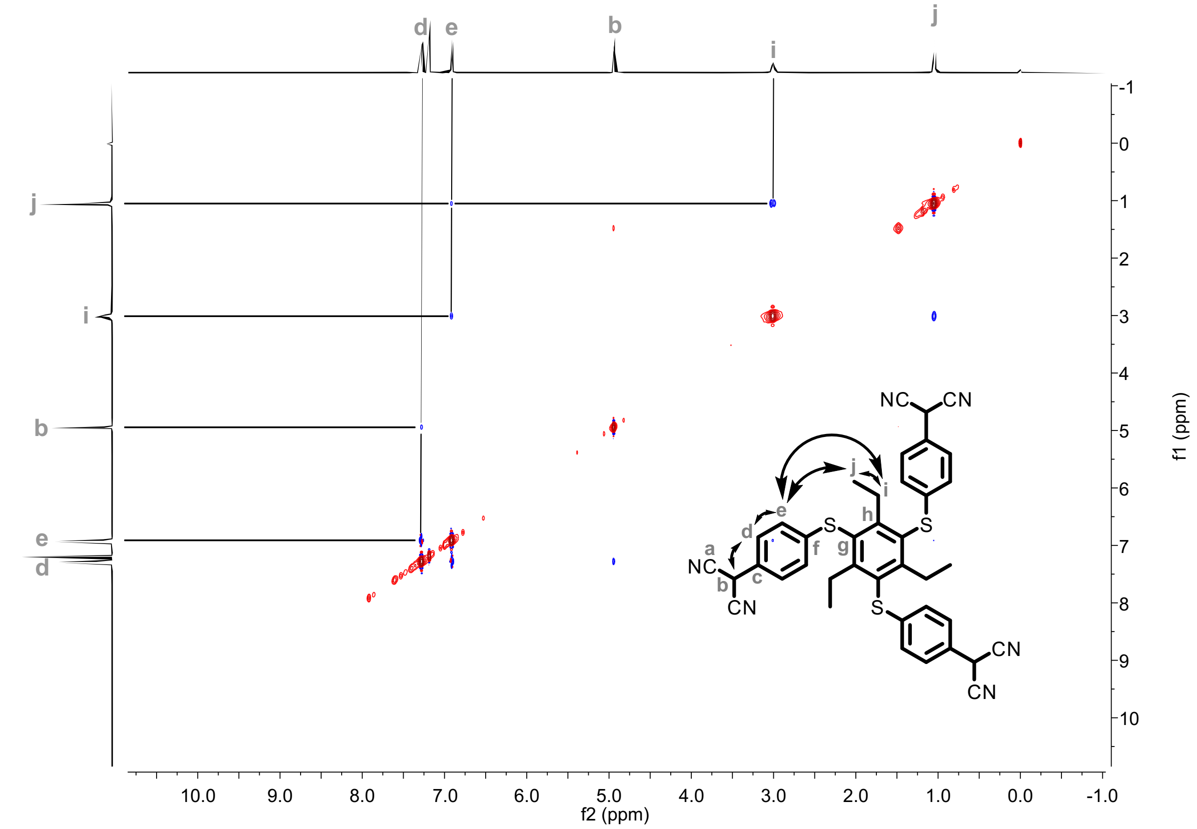

Figure S88: ^{1}H-^{1}H NOESY NMR spectrum (600 MHz, $CDCl_3$, 298 K) of **S**.

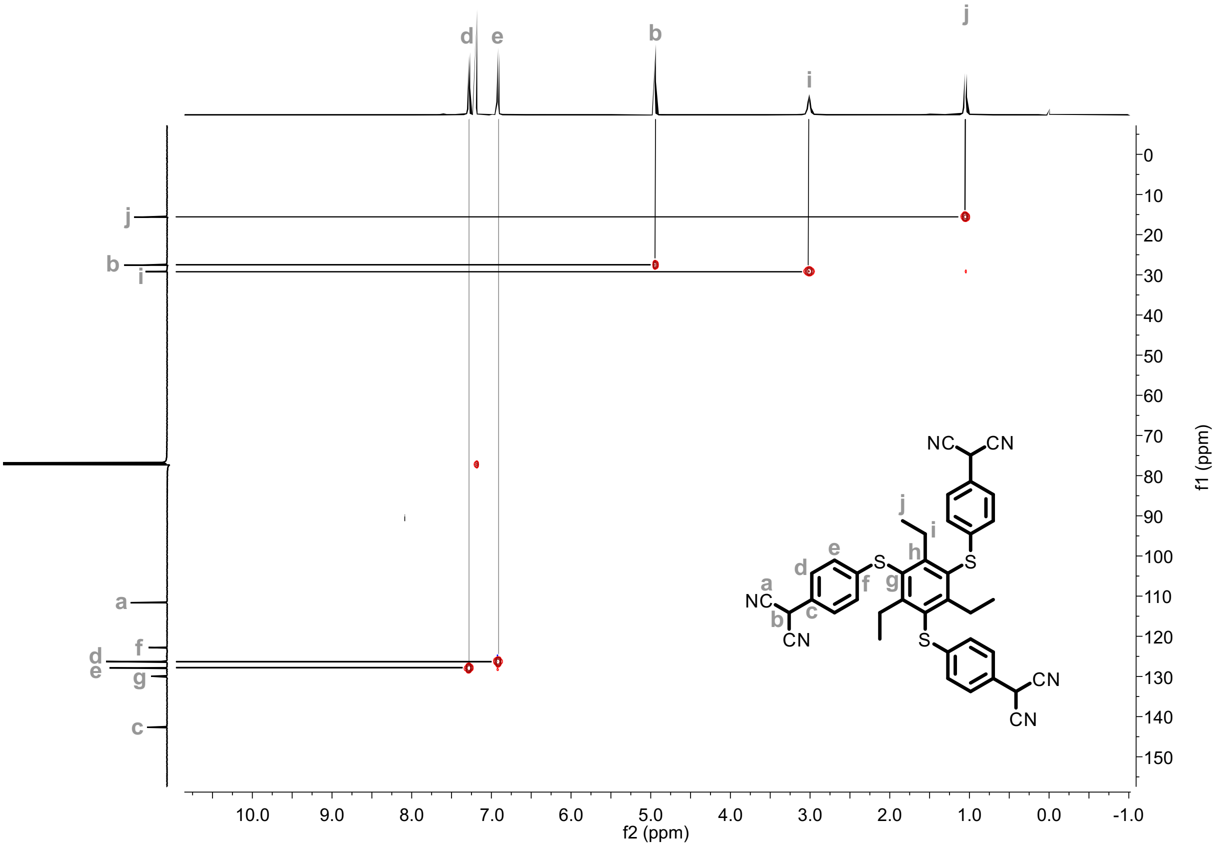

Figure S89: ^{1}H-^{13}C HSQC NMR spectrum (151 MHz, $CDCl_3$, 298 K) of **S**.

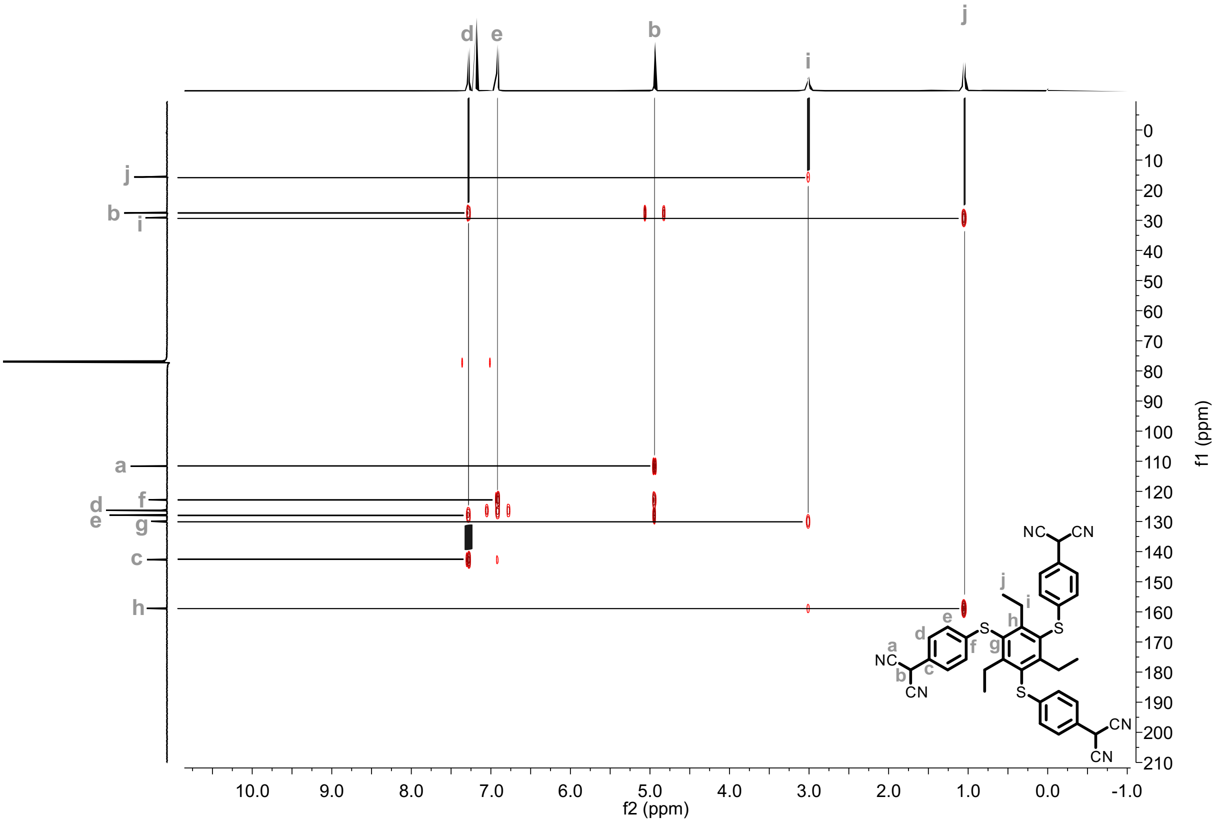

Figure S90: ^{1}H-^{13}C HMBC NMR spectrum (151 MHz, $CDCl_3$, 298 K) of **S**.

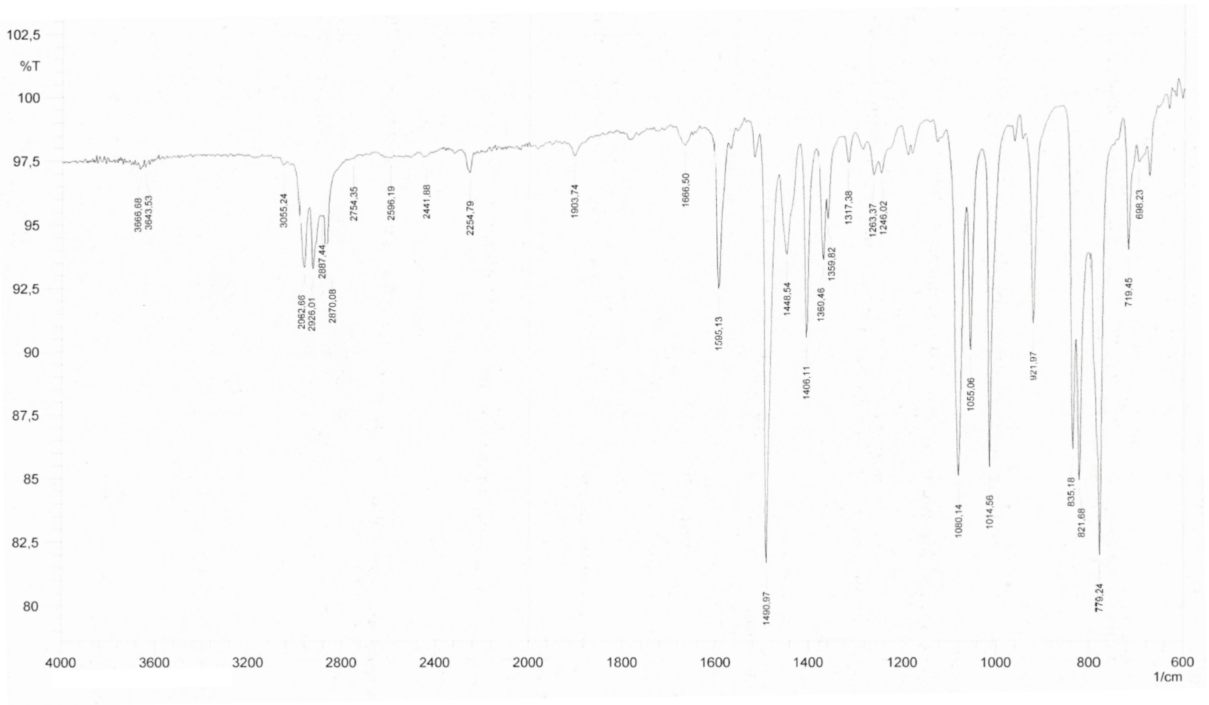

Figure S91: IR spectrum of **S**.

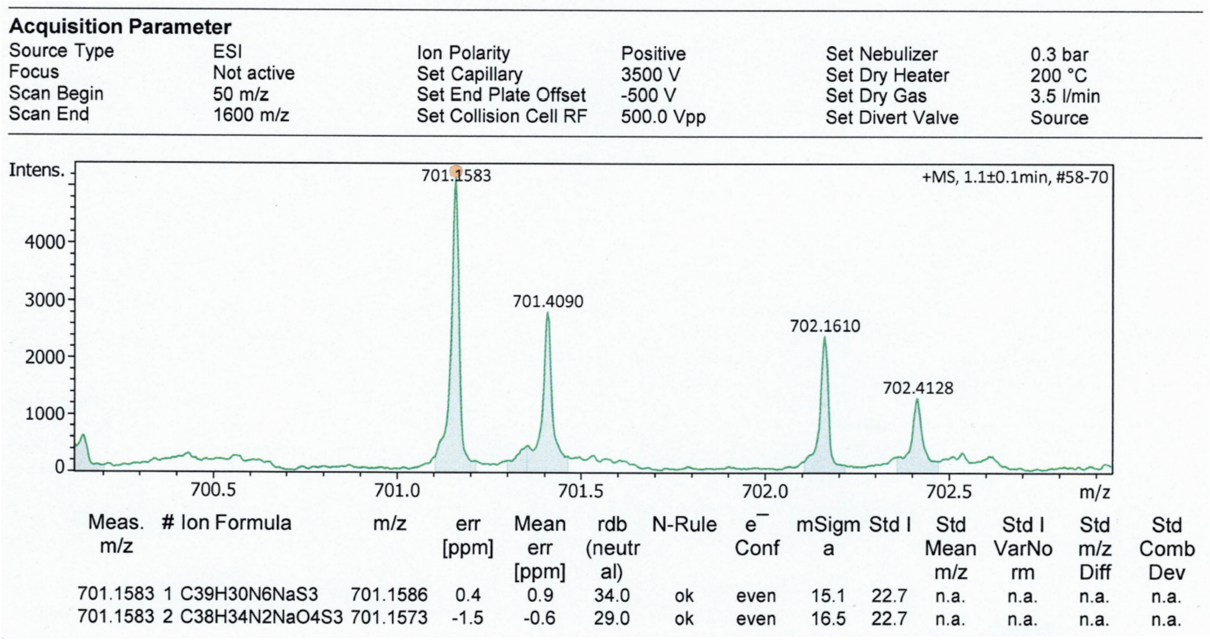

Figure S92: HRMS (ESI) spectrum of **S**.

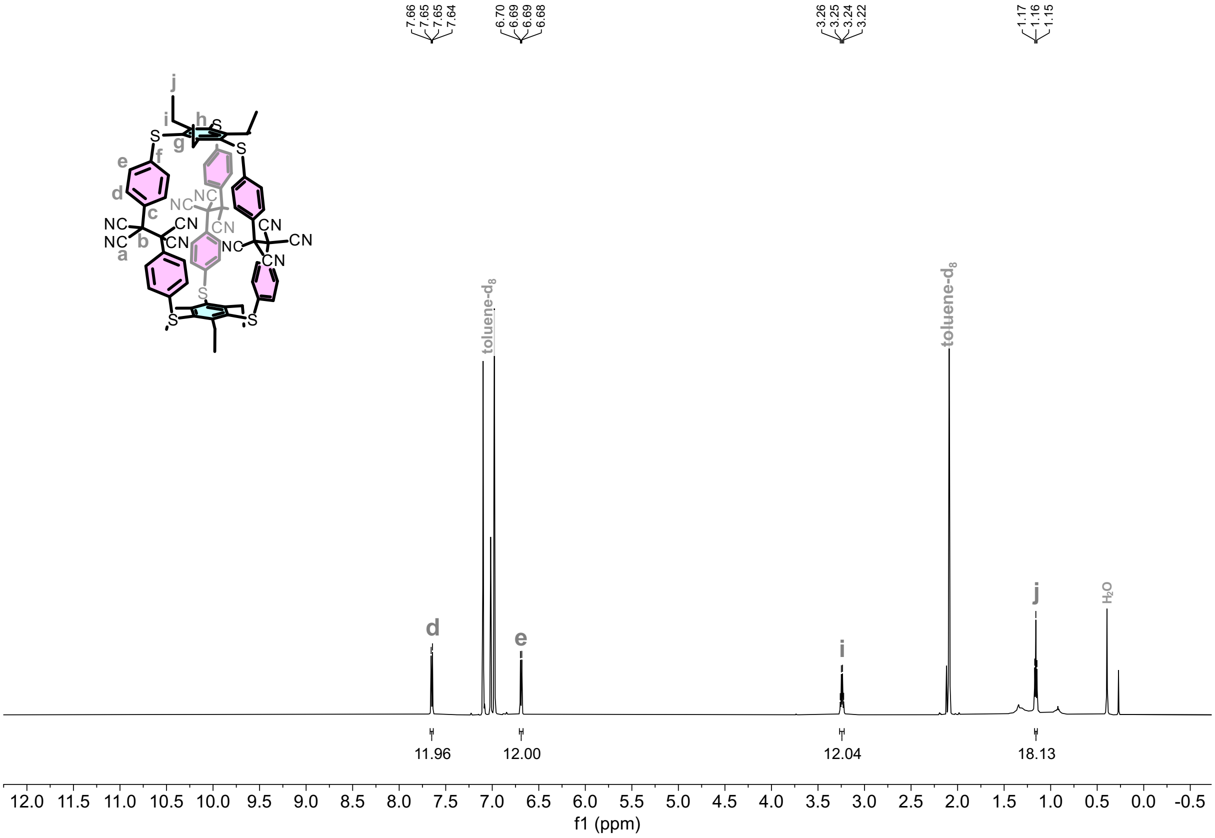

Figure S93: ^{1}H NMR spectrum (600 MHz, toluene-d$_8$, 298 K) of **S^2**.

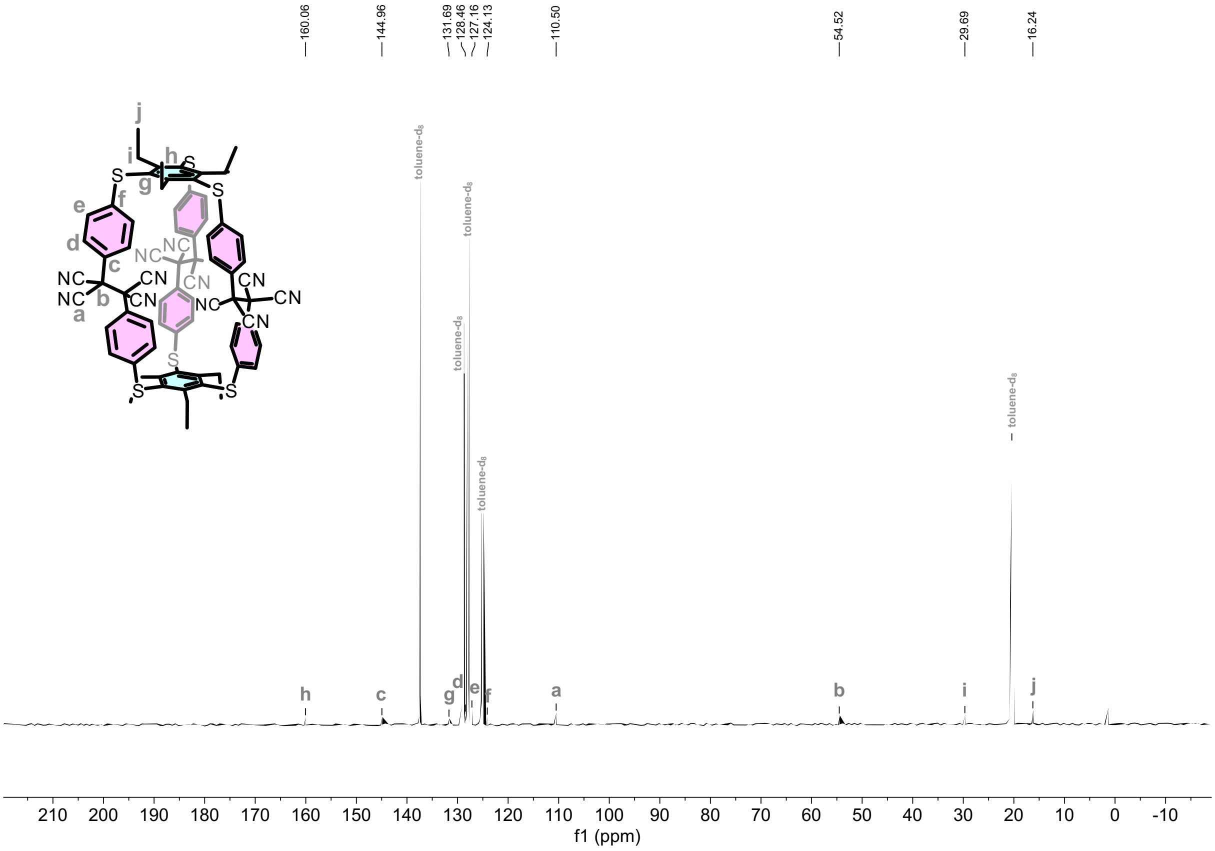

Figure S94: $^{13}C\{^{1}H\}$ NMR spectrum (151 MHz, toluene-d8, 298 K) of **S²**.

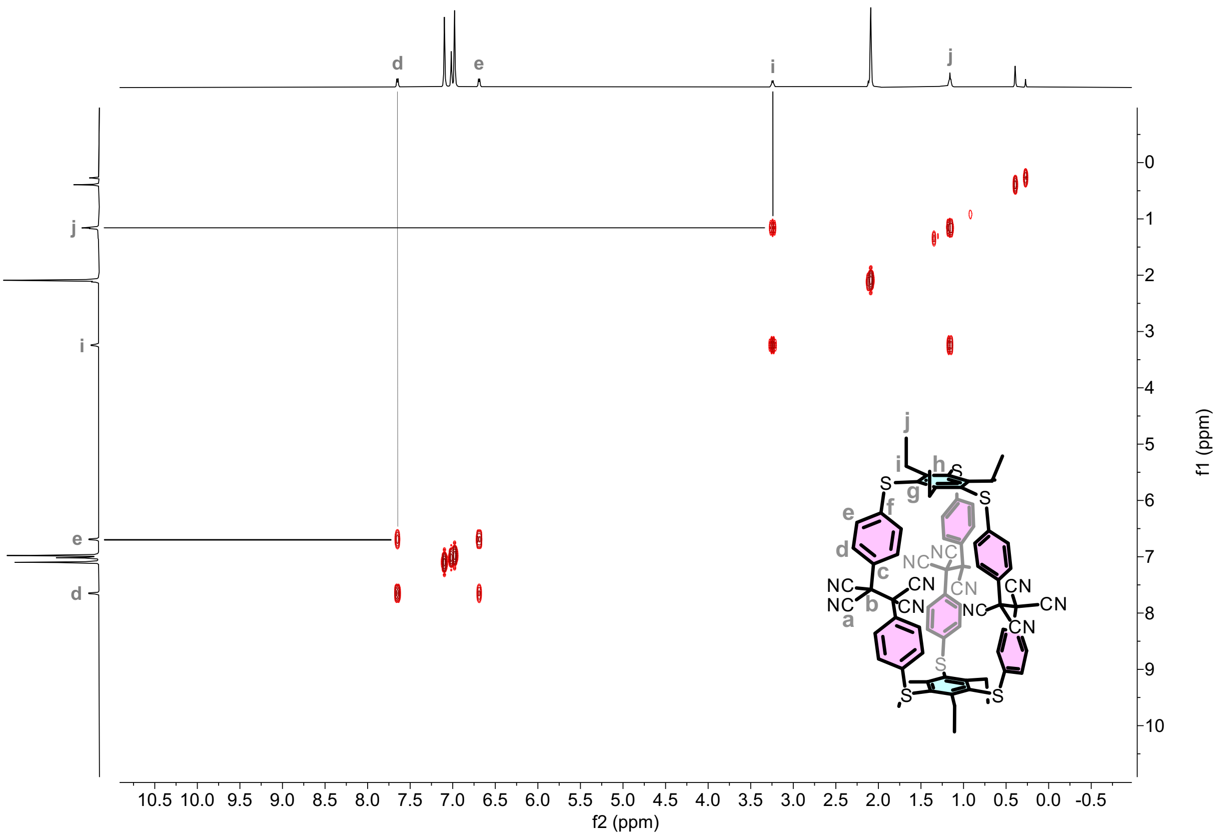

Figure S95: ^{1}H-^{1}H COSY NMR spectrum (600 MHz, toluene-d8, 298 K) of **S²**.

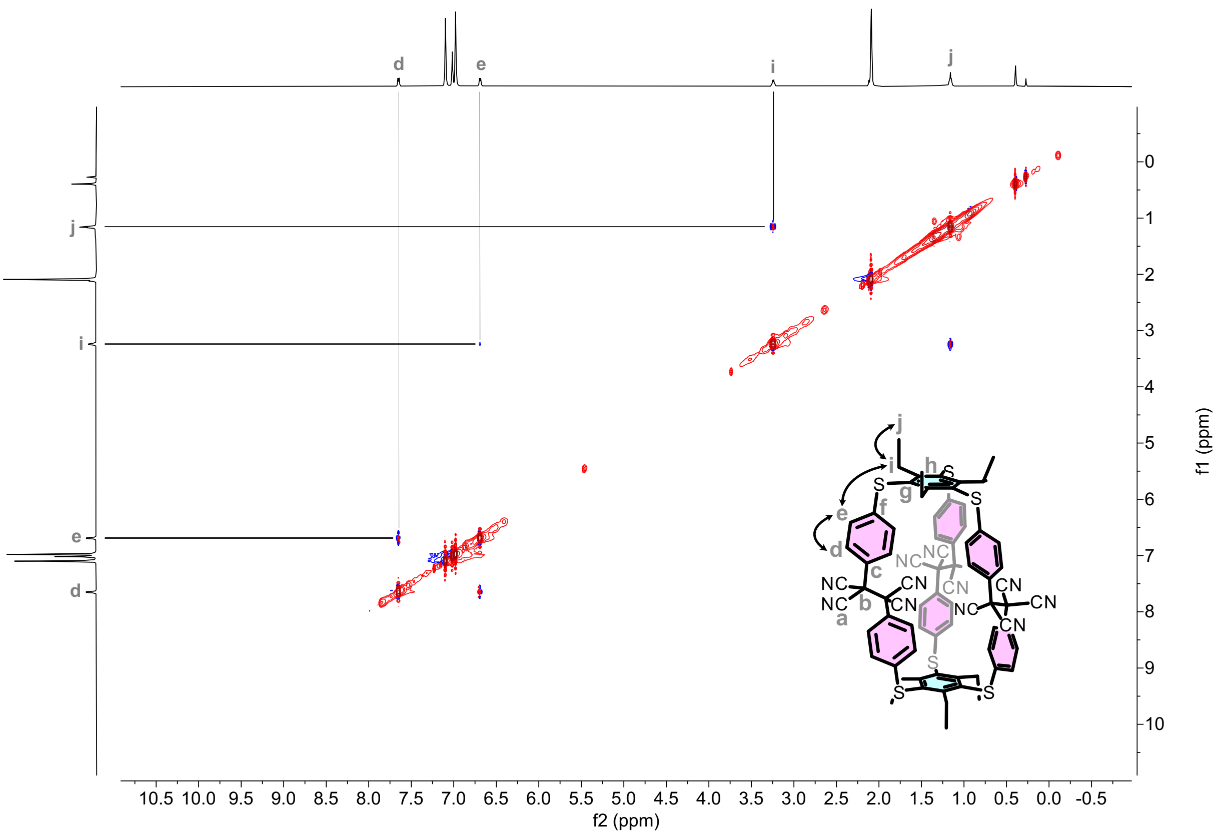

Figure S96: ^{1}H-^{1}H NOESY NMR spectrum (600 MHz, toluene-d₈, 298 K) of **S²**.

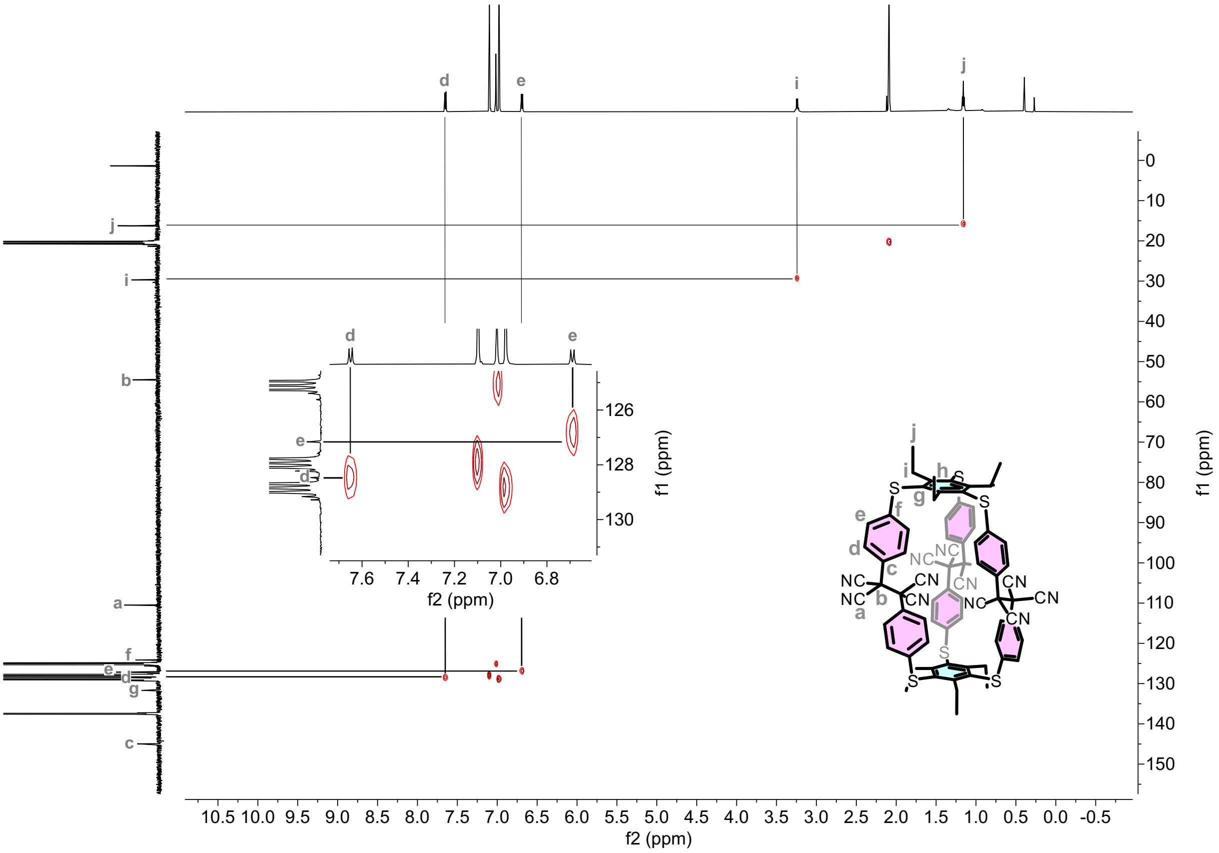

Figure S97: ^{1}H-^{13}C HSQC NMR spectrum (151 MHz, toluene-d₈, 298 K) of **S²**.

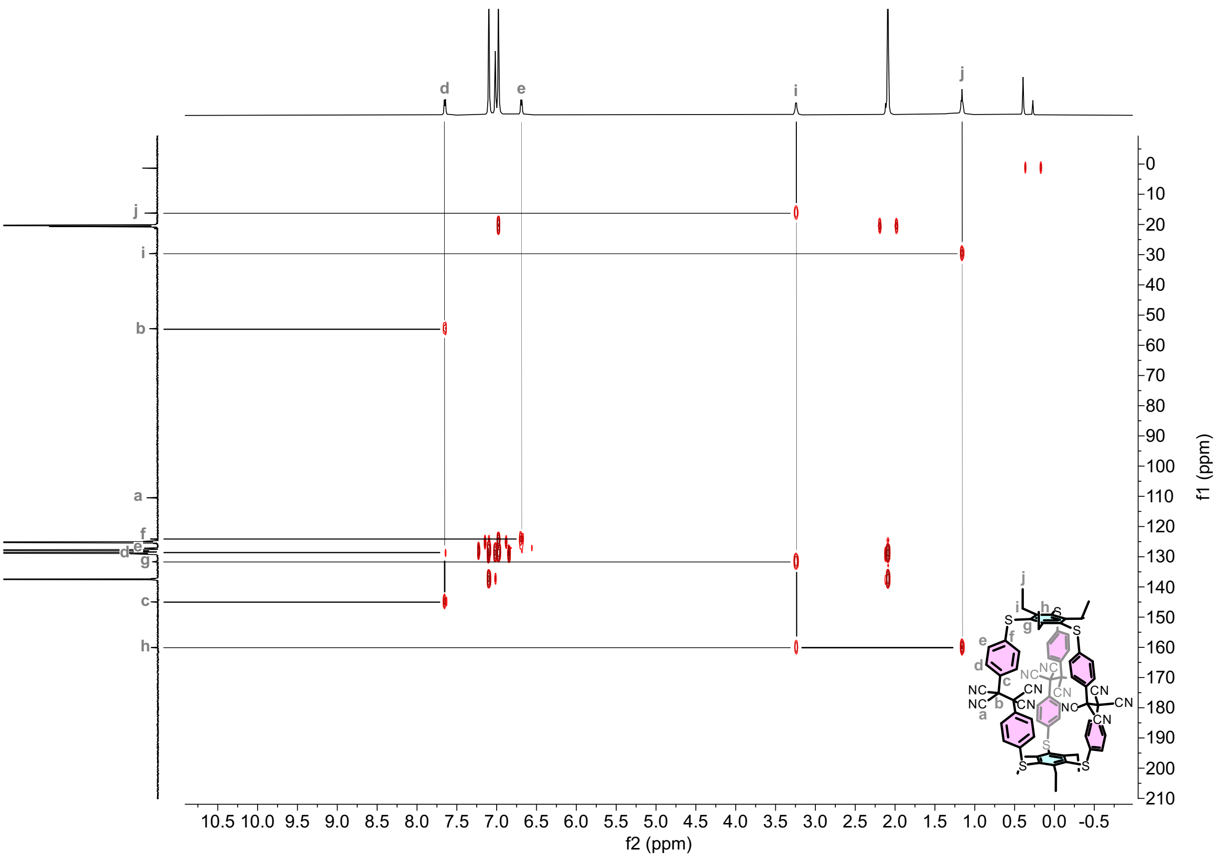

Figure S98: ^{1}H-^{13}C HMBC NMR spectrum (151 MHz, toluene-d_8, 298 K) of $\mathbf{S^2}$.

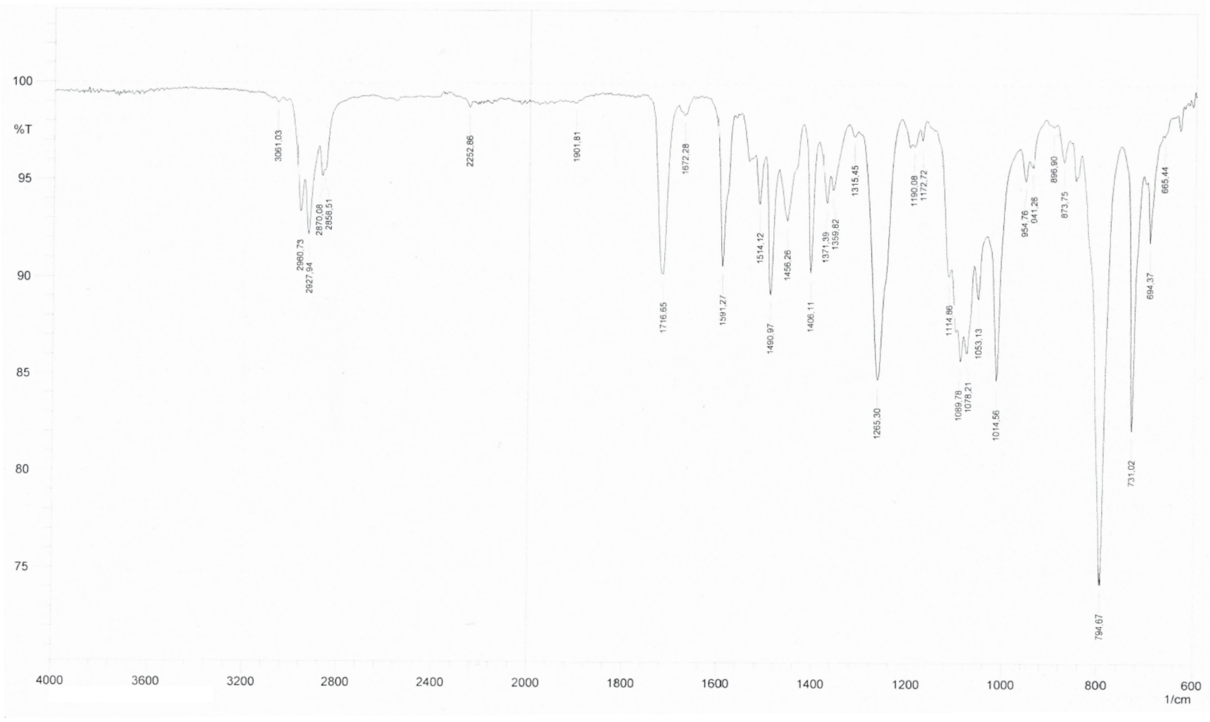

Figure S99: IR spectrum of $\mathbf{S^2}$.

Acquisition Parameter

Source Type	ESI	Ion Polarity	Positive	Set Nebulizer	0.3 Bar
Focus	Active	Set Capillary	4000 V	Set Dry Heater	180 °C
Scan Begin	300 m/z	Set End Plate Offset	-500 V	Set Dry Gas	4.0 l/min
Scan End	2800 m/z	Set Collision Cell RF	2500.0 Vpp	Set Divert Valve	Source

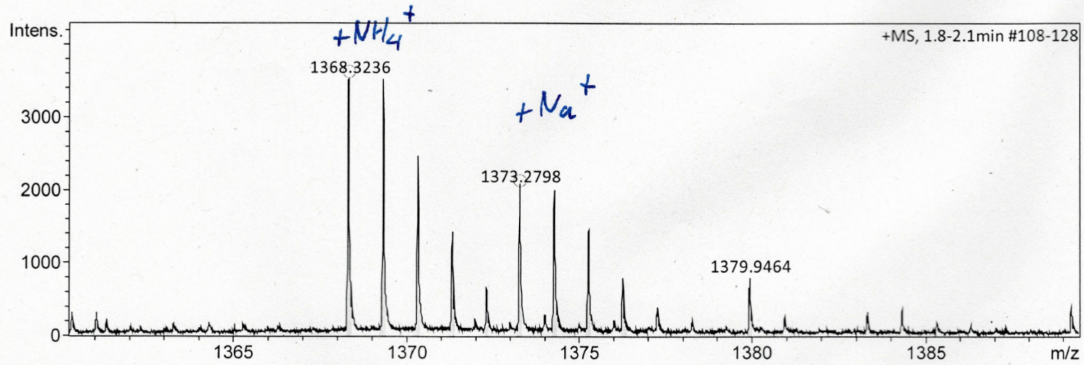

Meas. m/z	#	Ion Formula	m/z	err [ppm]	mSigma	# mSigma	Score	rdb	e⁻ Conf	N-Rule
1368.3236	1	C78H58N13S6	1368.3257	1.5	35.1	1	49.56	56.5	even	ok
	2	C77H62N9O4S6	1368.3244	0.5	41.3	2	100.00	51.5	even	ok
	3	C76H66N5O8S6	1368.3230	-0.4	49.0	3	85.66	46.5	even	ok
	4	C75H70NO12S6	1368.3217	-1.4	57.7	4	28.38	41.5	even	ok
1373.2798	1	C77H58N8NaO4S6	1373.2797	-0.1	71.8	1	100.00	52.5	even	ok
	2	C76H62N4NaO8S6	1373.2784	-1.0	72.0	2	48.78	47.5	even	ok
	3	C78H54N12NaS6	1373.2811	0.9	73.1	3	52.82	57.5	even	ok

Figure S100: HRMS (ESI) spectrum of **S^2**.

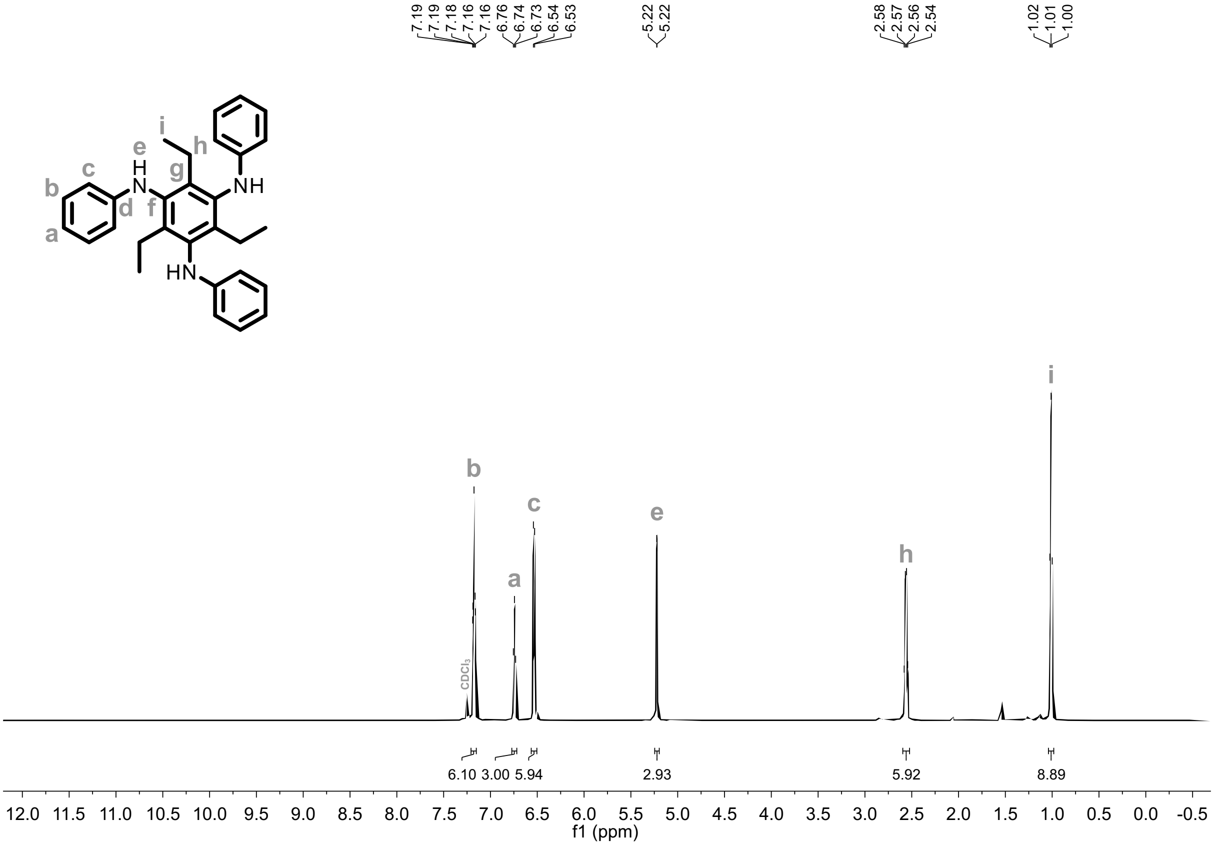

Figure S101: ^{1}H NMR spectrum (600 MHz, CDCl$_3$, 298 K) of **9**.

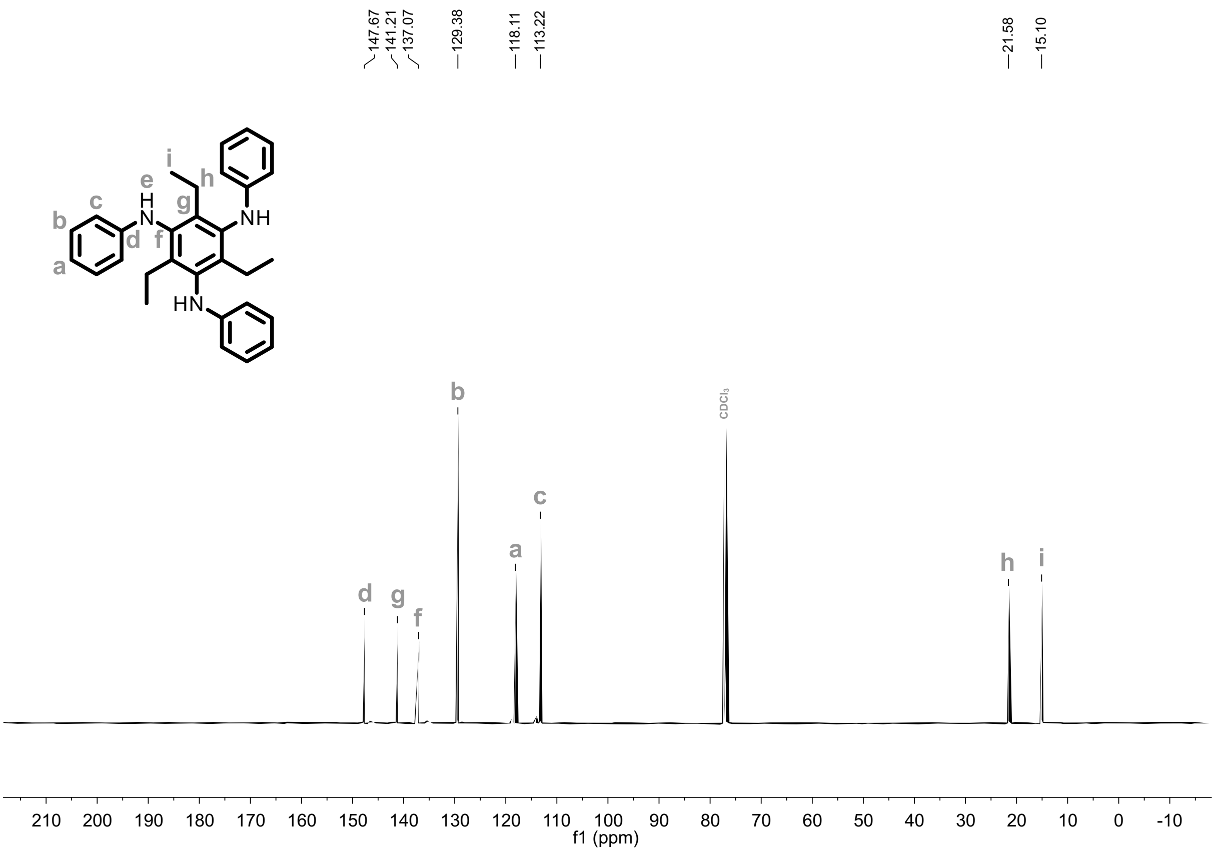

Figure S102: $^{13}C\{^{1}H\}$ NMR spectrum (151 MHz, $CDCl_3$, 298 K) of **9**.

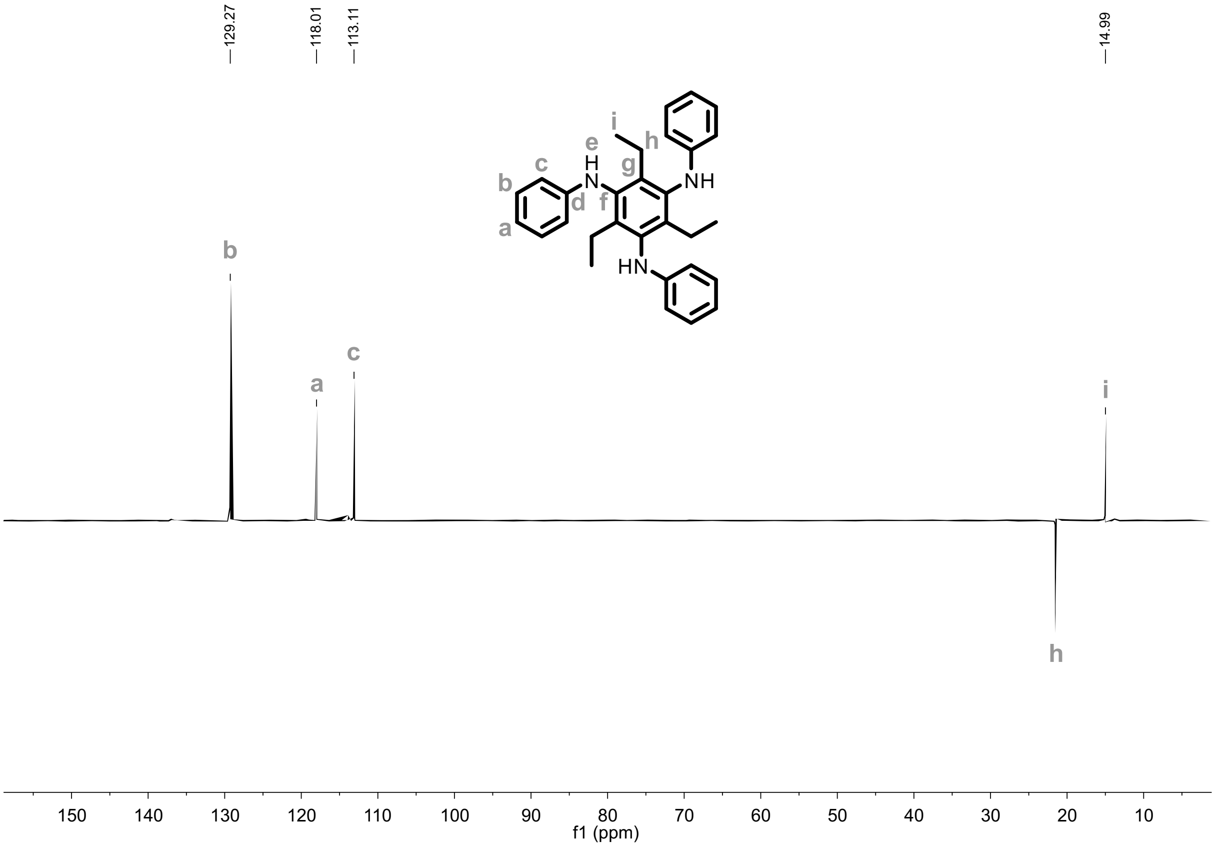

Figure S103: ^{13}C DEPT-135 NMR spectrum (151 MHz, $CDCl_3$, 298 K) of **9**.

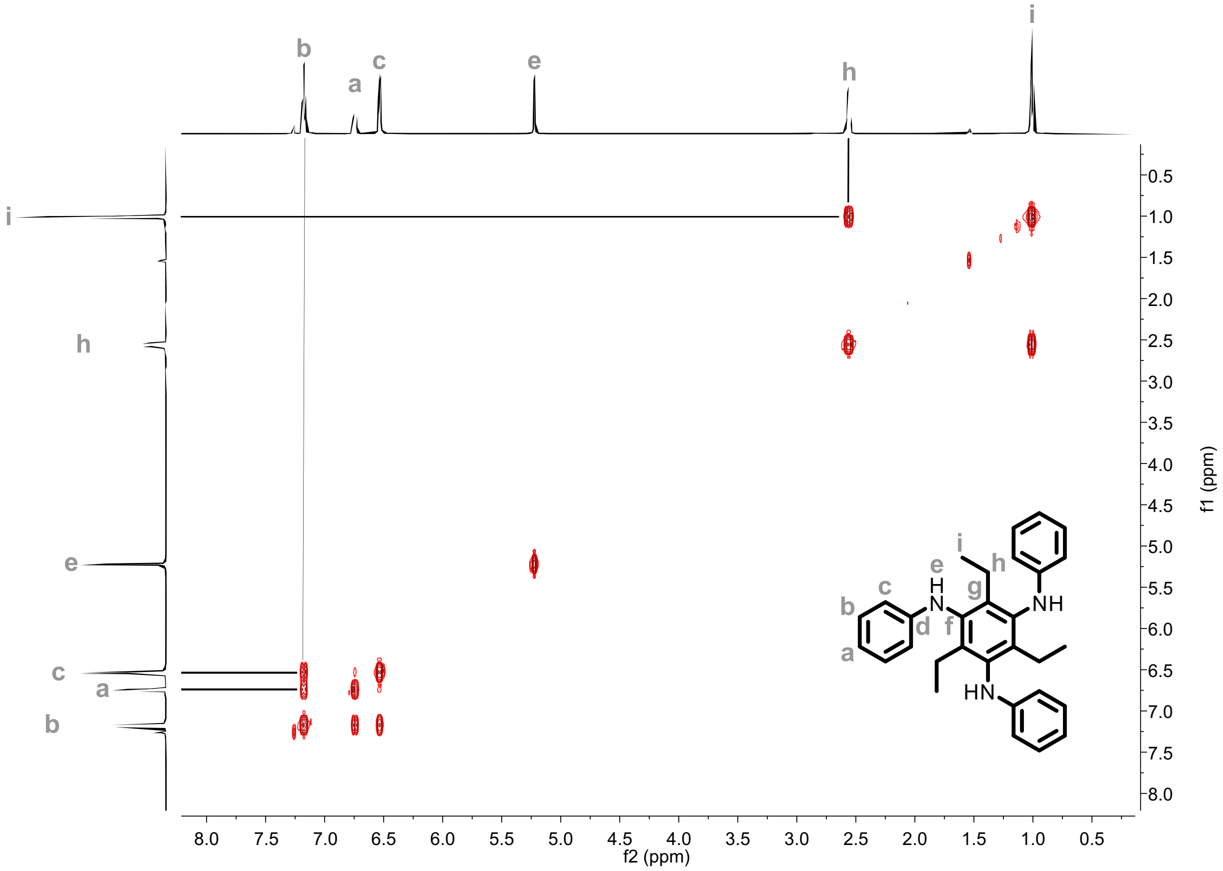

Figure S104: ^{1}H-^{1}H COSY NMR spectrum (600 MHz, $CDCl_3$, 298 K) of **9**.

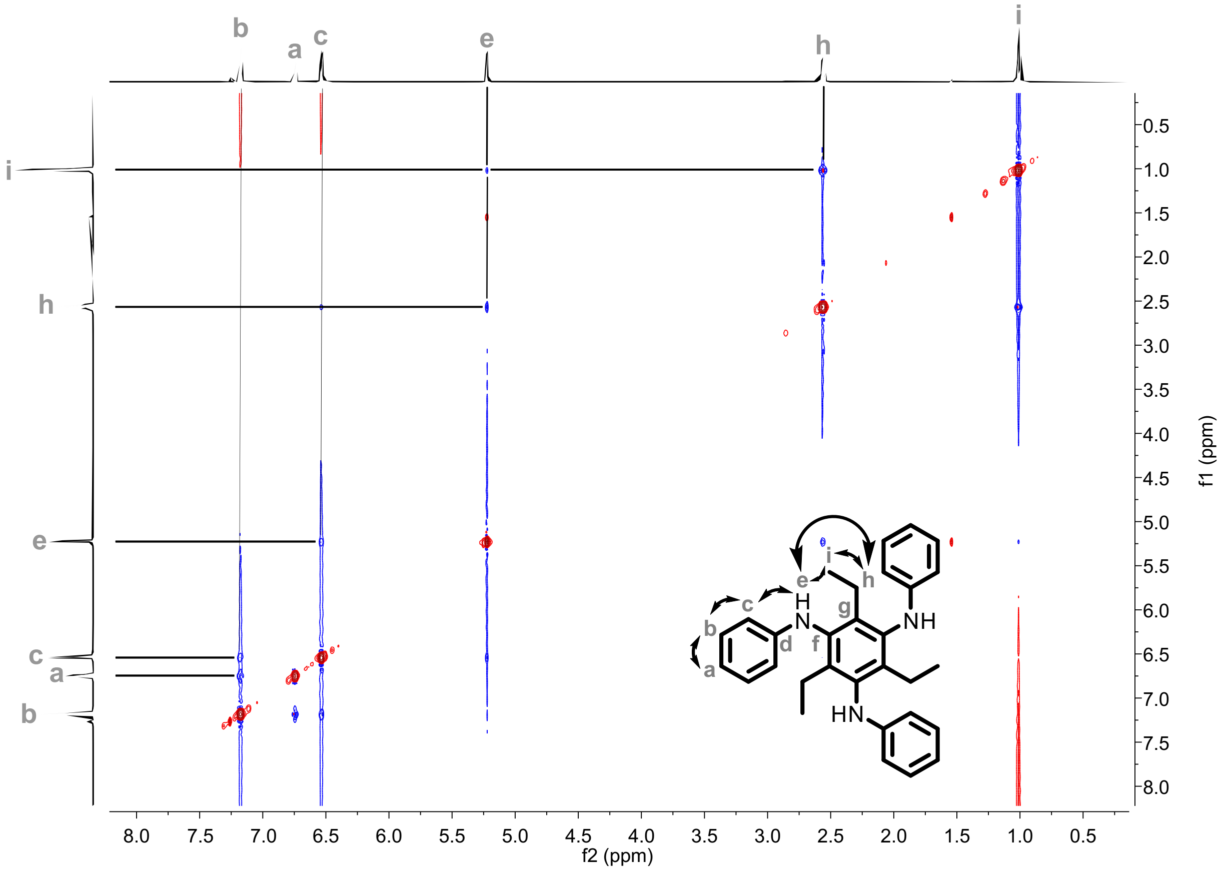

Figure S105: ^{1}H-^{1}H NOESY NMR spectrum (600 MHz, $CDCl_3$, 298 K) of **9**.

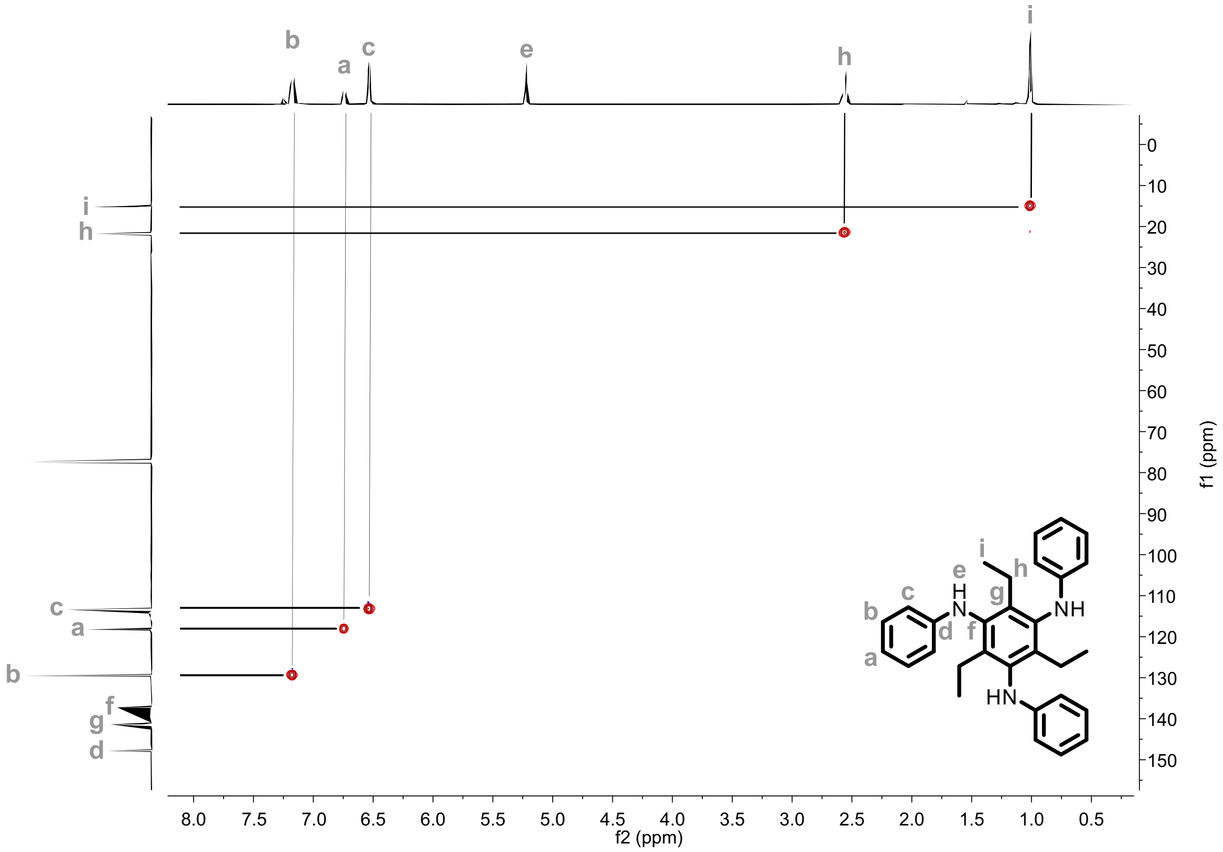

Figure S106: ^{1}H-^{13}C HSQC NMR spectrum (151 MHz, $CDCl_3$, 298 K) of **9**.

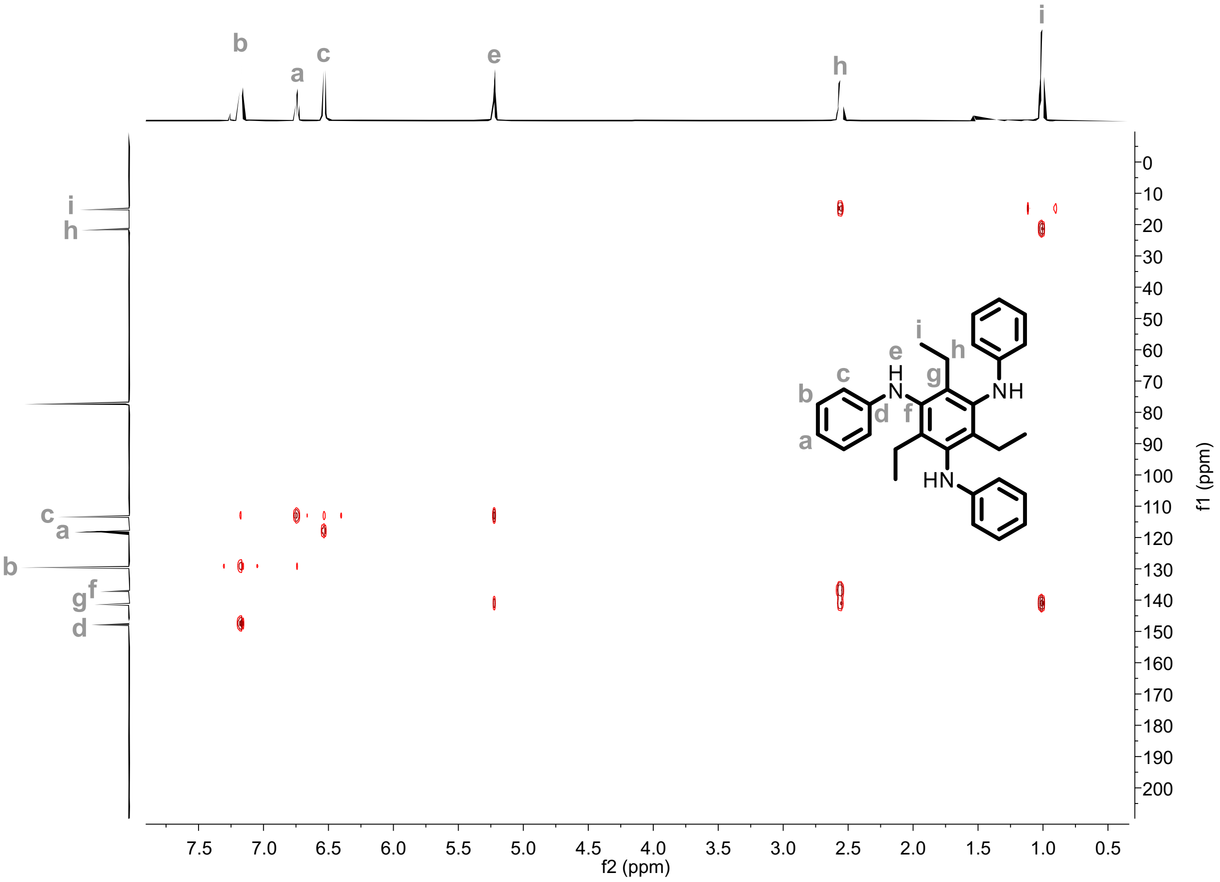

Figure S107: ^{1}H-^{13}C HMBC NMR spectrum (151 MHz, $CDCl_3$, 298 K) of **9**.

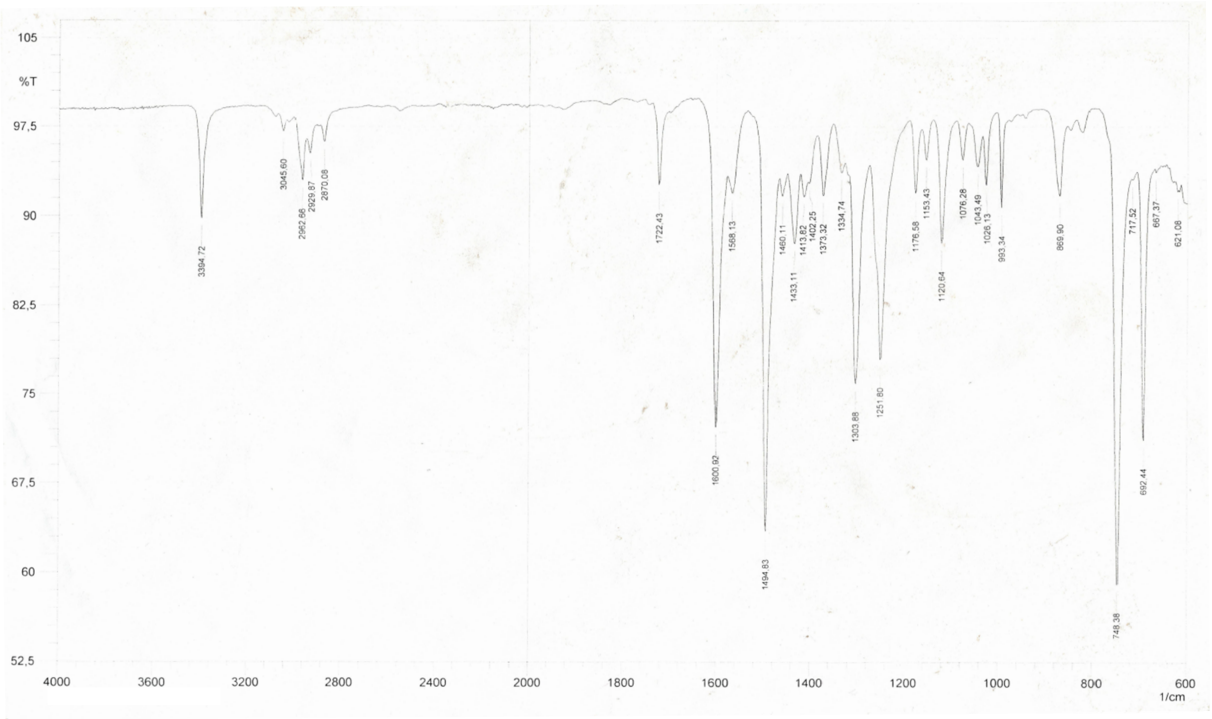

Figure S108: IR spectrum of **9**.

Acquisition Parameter

Source Type	ESI	Ion Polarity	Positive	Set Nebulizer	0.3 bar
Focus	Not active	Set Capillary	3500 V	Set Dry Heater	200 °C
Scan Begin	50 m/z	Set End Plate Offset	-500 V	Set Dry Gas	3.5 l/min
Scan End	1600 m/z	Set Collision Cell RF	500.0 Vpp	Set Divert Valve	Source

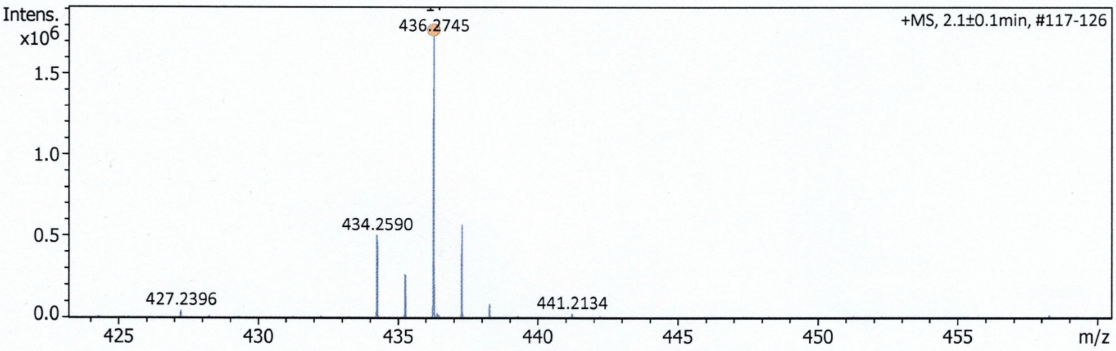

Meas. m/z	#	Ion Formula	m/z	err [ppm]	Mean err [ppm]	rdb (neutral)	N-Rule	e⁻ Conf	mSigma	Std I	Std Mean m/z	Std I VarNorm	Std m/z Diff	Std Comb Dev
436.2745	1	C30H34N3	436.2747	0.5	0.3	16.0	ok	even	8.1	12.0	n.a.	n.a.	n.a.	n.a.

Figure S109: HRMS (ESI) spectrum of **9**.

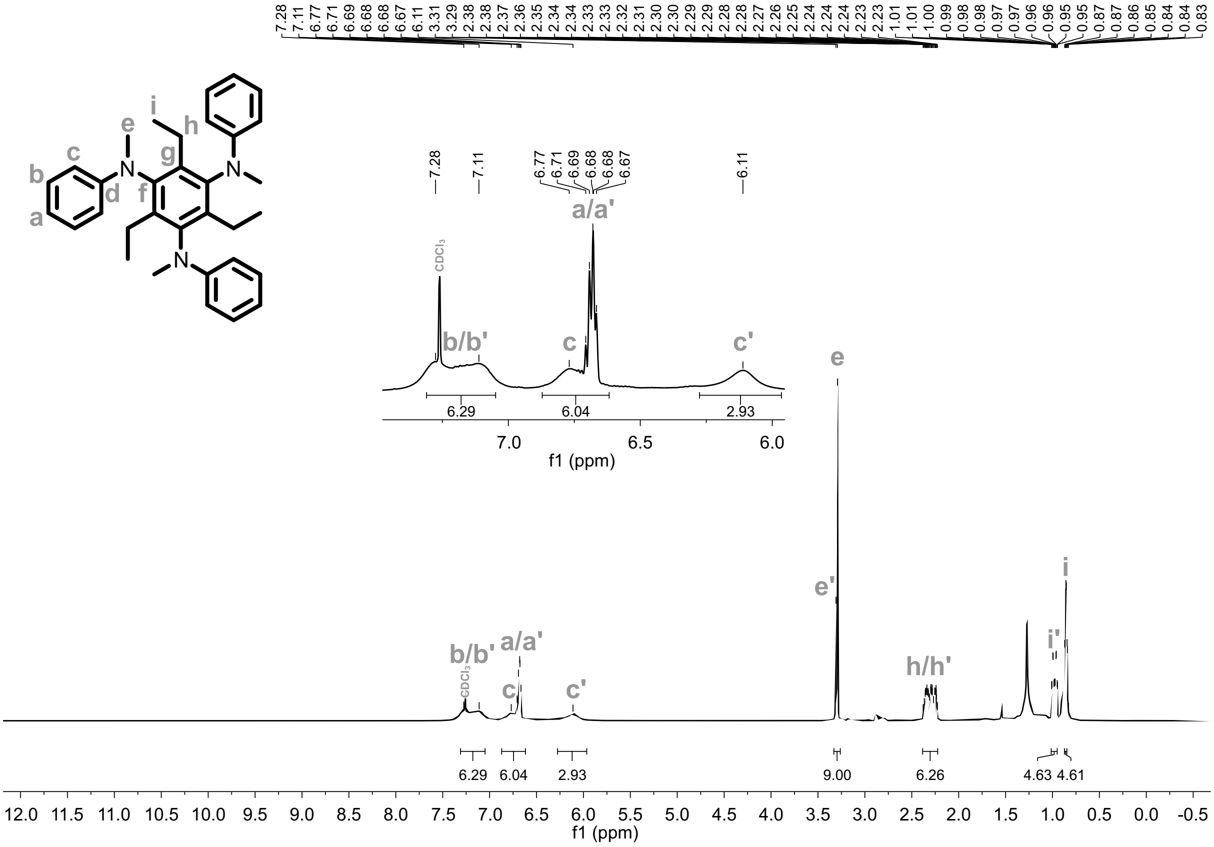

Figure S110: ^{1}H NMR spectrum (600 MHz, $CDCl_3$, 298 K) of **10**.

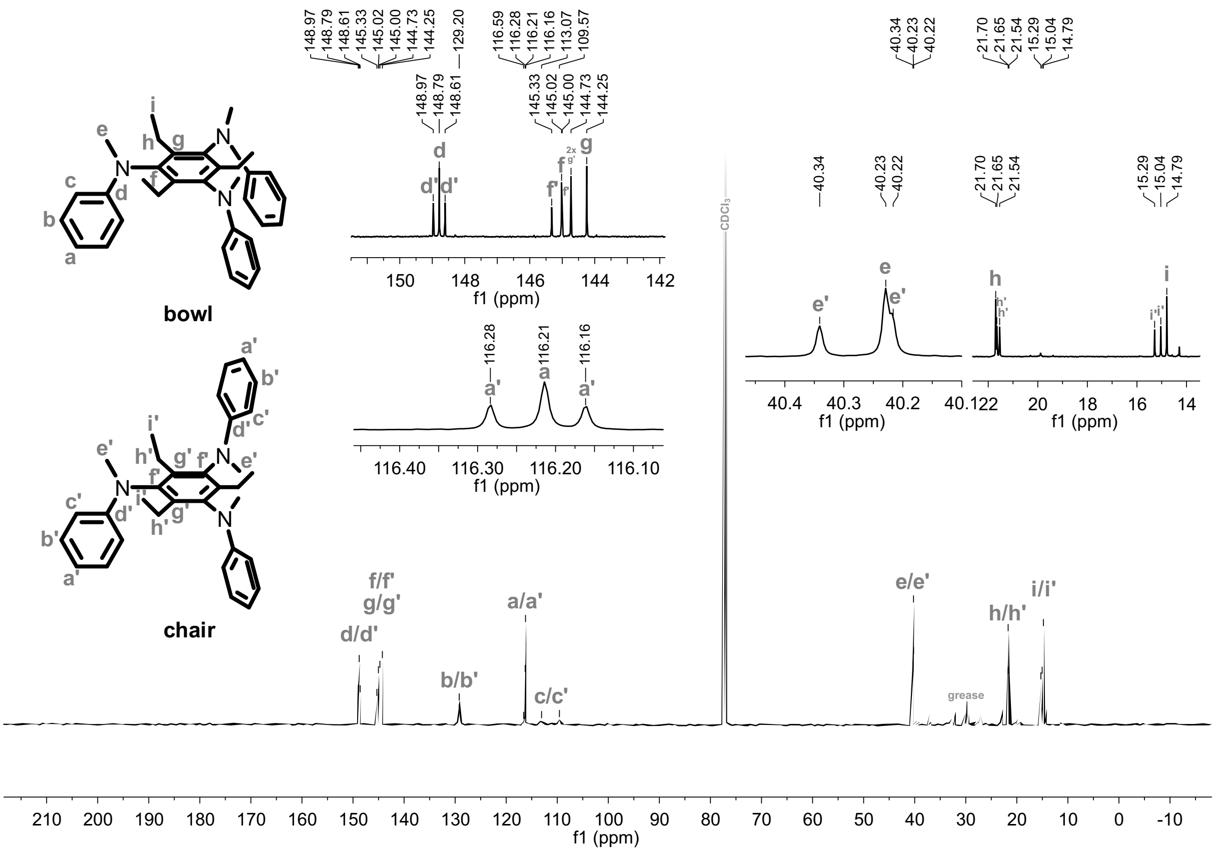

Figure S111: $^{13}C\{^{1}H\}$ NMR spectrum (151 MHz, $CDCl_3$, 298 K) of **10**.

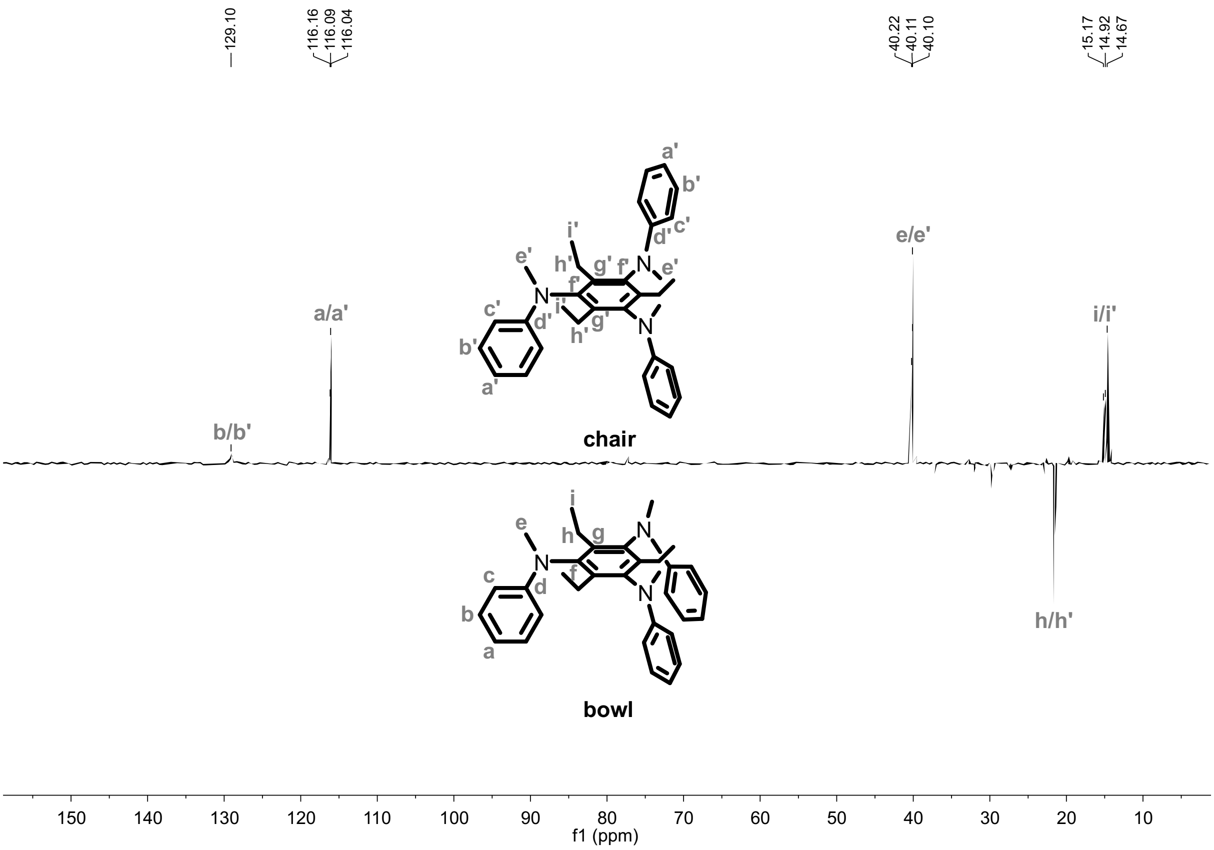

Figure S112: ^{13}C DEPT-135 NMR spectrum (151 MHz, $CDCl_3$, 298 K) of **10**.

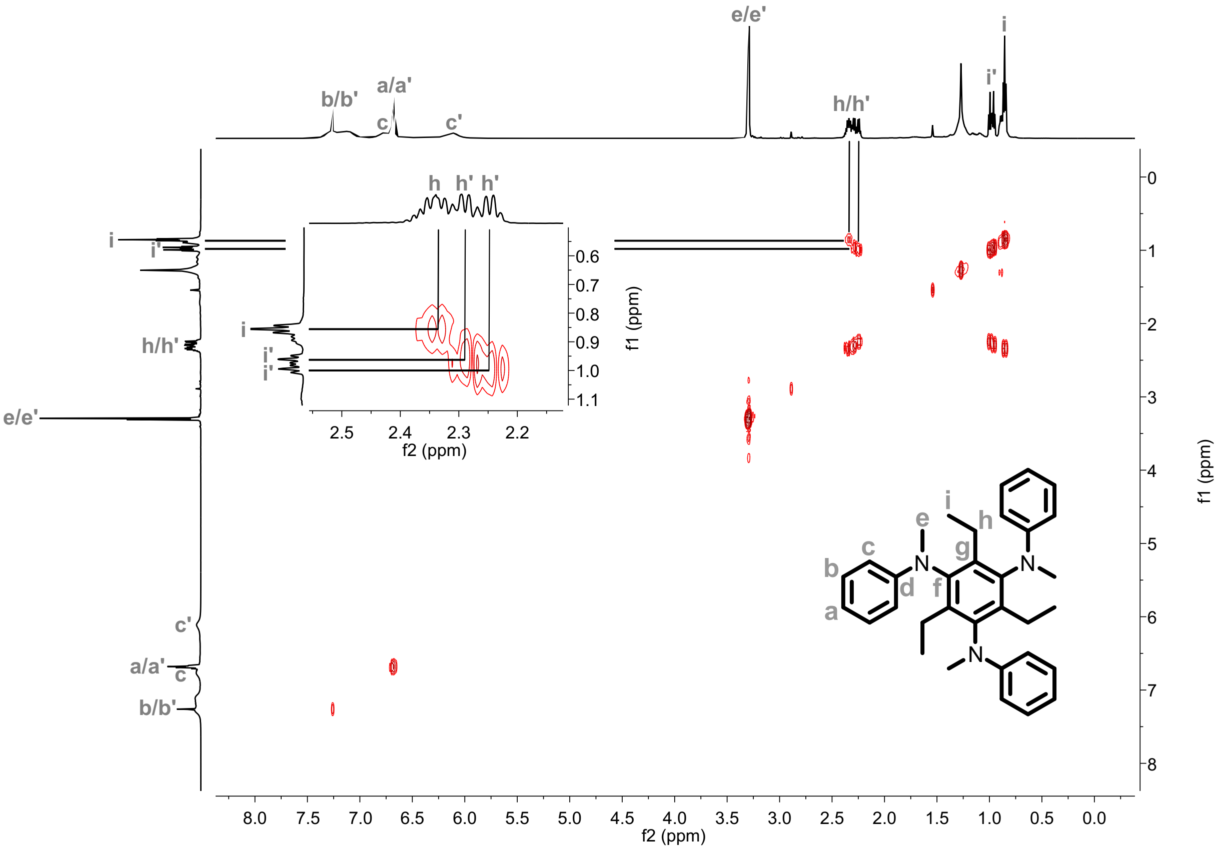

Figure S113: ^{1}H-^{1}H COSY NMR spectrum (600 MHz, $CDCl_3$, 298 K) of **10**.

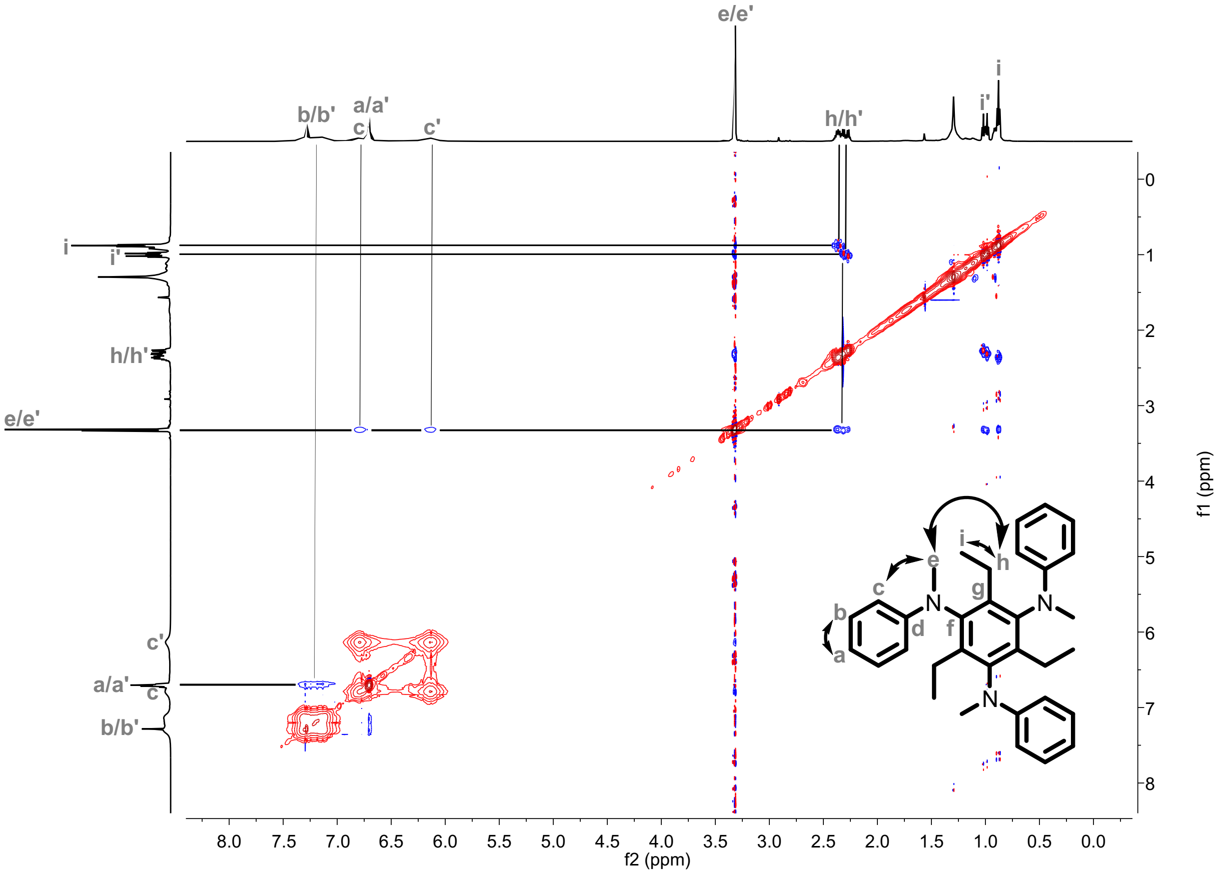

Figure S114: ^{1}H-^{1}H NOESY NMR spectrum (600 MHz, $CDCl_3$, 298 K) of **10**.

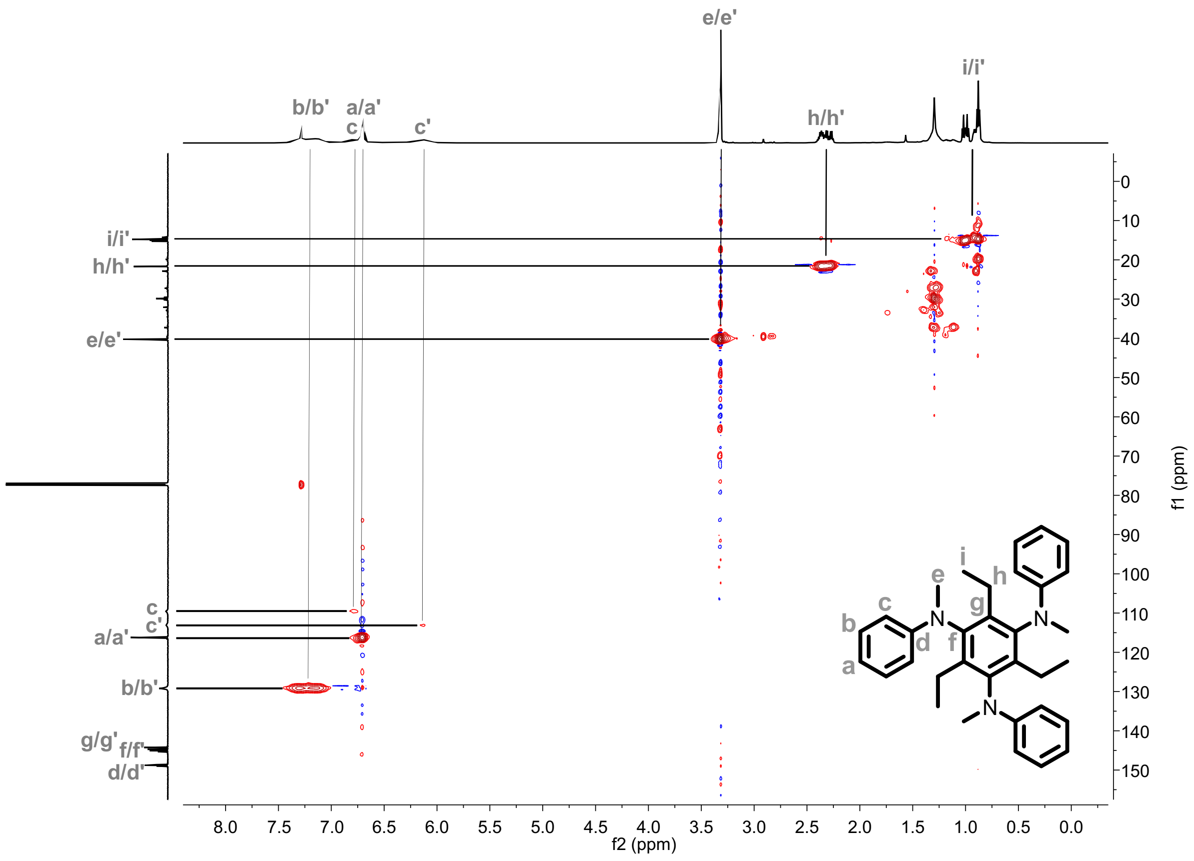

Figure S115: ^{1}H-^{13}C HSQC NMR spectrum (151 MHz, $CDCl_3$, 298 K) of **10**.

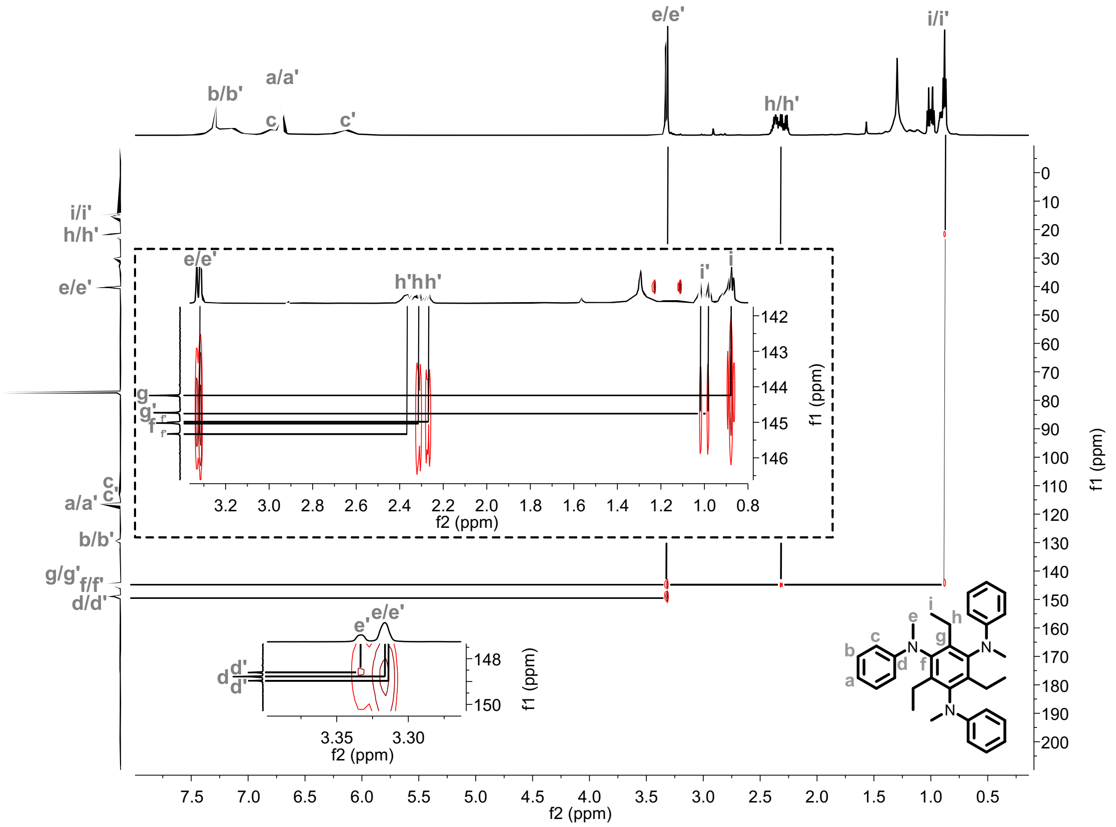

Figure S116: ^{1}H-^{13}C HMBC NMR spectrum (151 MHz, $CDCl_3$, 298 K) of **10**.

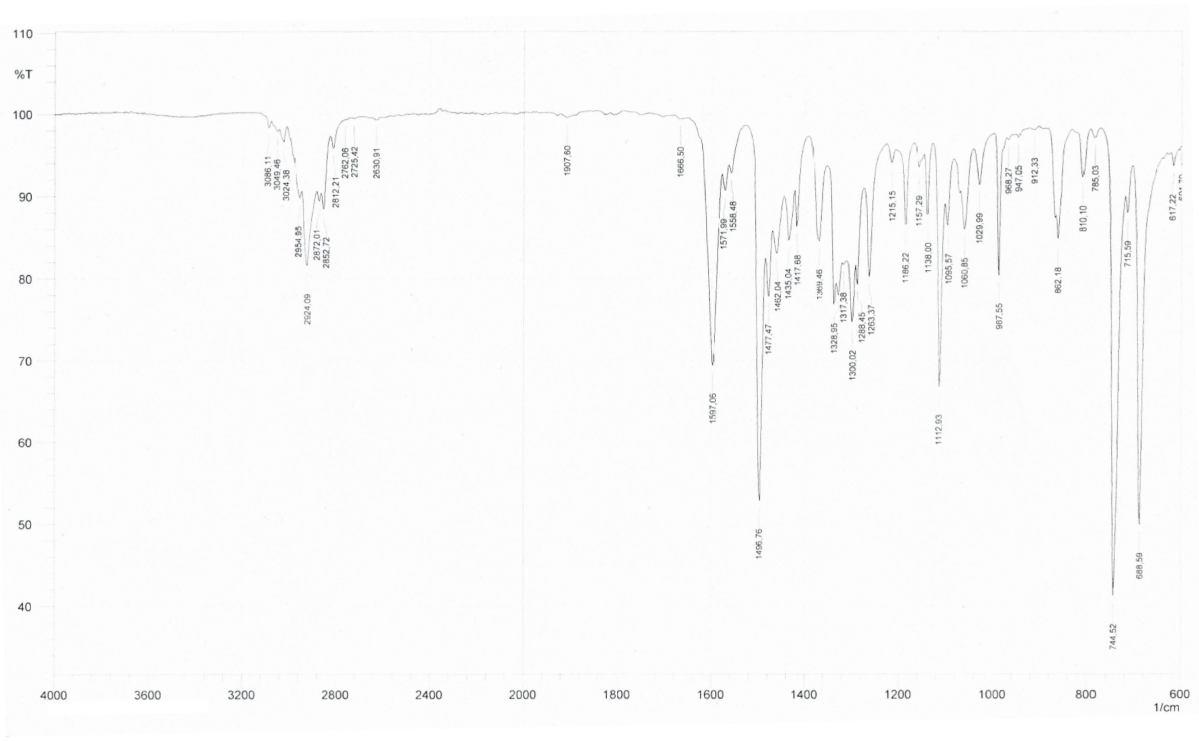

Figure S117: IR spectrum of **10**.

Acquisition Parameter

Source Type	ESI	Ion Polarity	Positive	Set Nebulizer	0.3 bar
Focus	Not active	Set Capillary	3500 V	Set Dry Heater	200 °C
Scan Begin	50 m/z	Set End Plate Offset	-500 V	Set Dry Gas	3.5 l/min
Scan End	1600 m/z	Set Collision Cell RF	500.0 Vpp	Set Divert Valve	Source

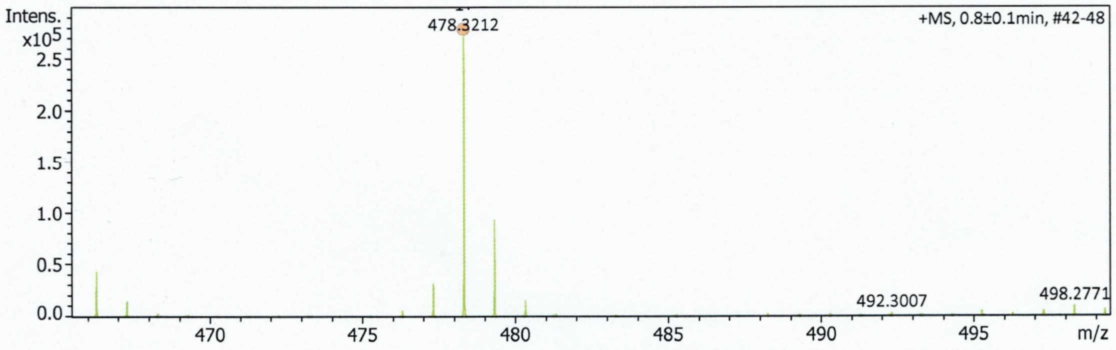

Meas. m/z	#	Ion Formula	m/z	err [ppm]	Mean err [ppm]	rdb (neutral)	N-Rule	e⁻ Conf	mSigma	Std I	Std Mean m/z	Std I VarNorm	Std m/z Diff	Std Comb Dev
478.3212	1	C33H40N3	478.3217	1.0	0.7	16.0	ok	even	15.4	23.8	n.a.	n.a.	n.a.	n.a.

Figure S118: HRMS (ESI) spectrum of **10**.

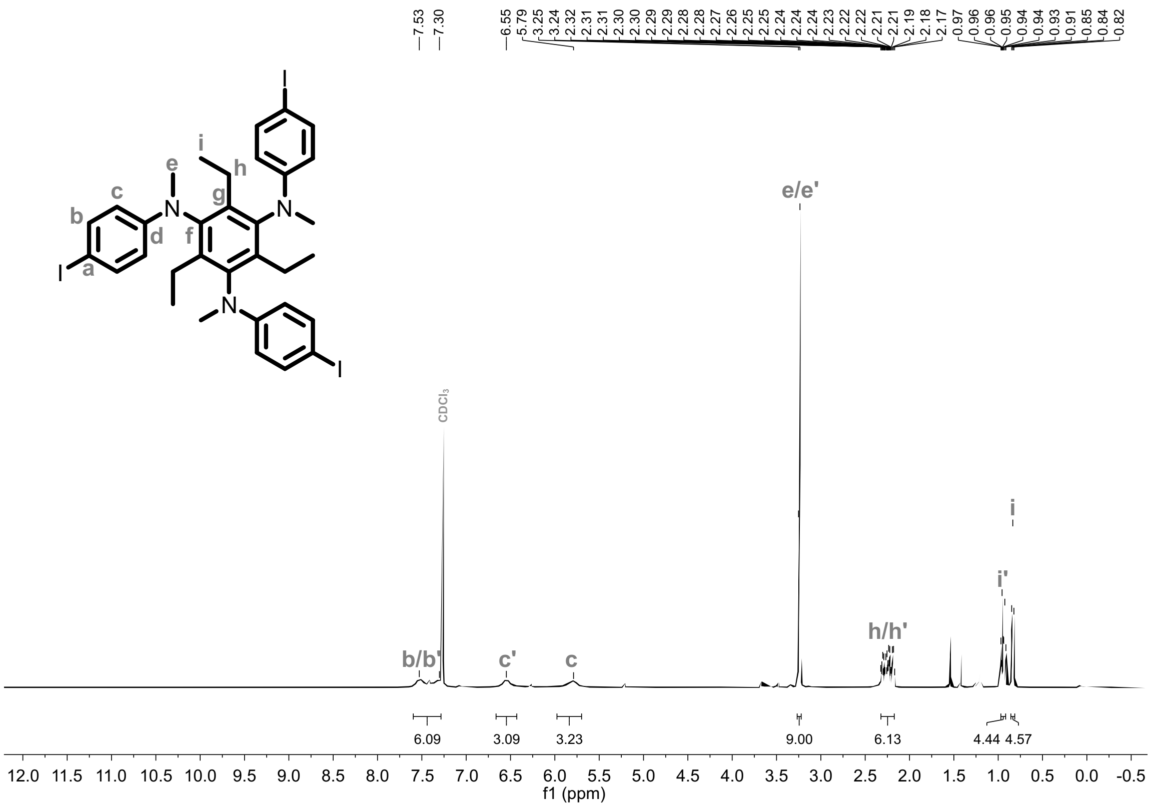

Figure S119: ^{1}H NMR spectrum (600 MHz, $CDCl_3$, 298 K) of **11**.

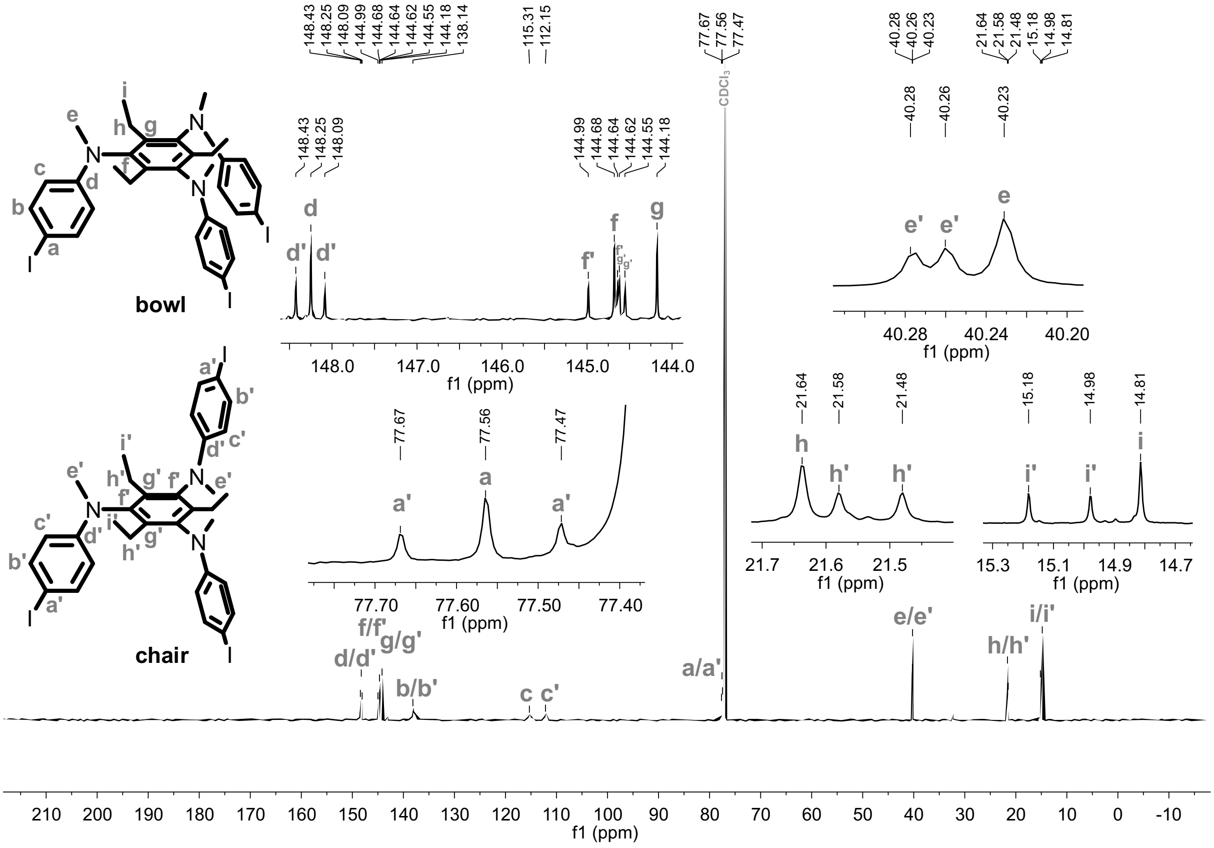

Figure S120: $^{13}C\{^{1}H\}$ NMR spectrum (151 MHz, $CDCl_3$, 298 K) of **11**.

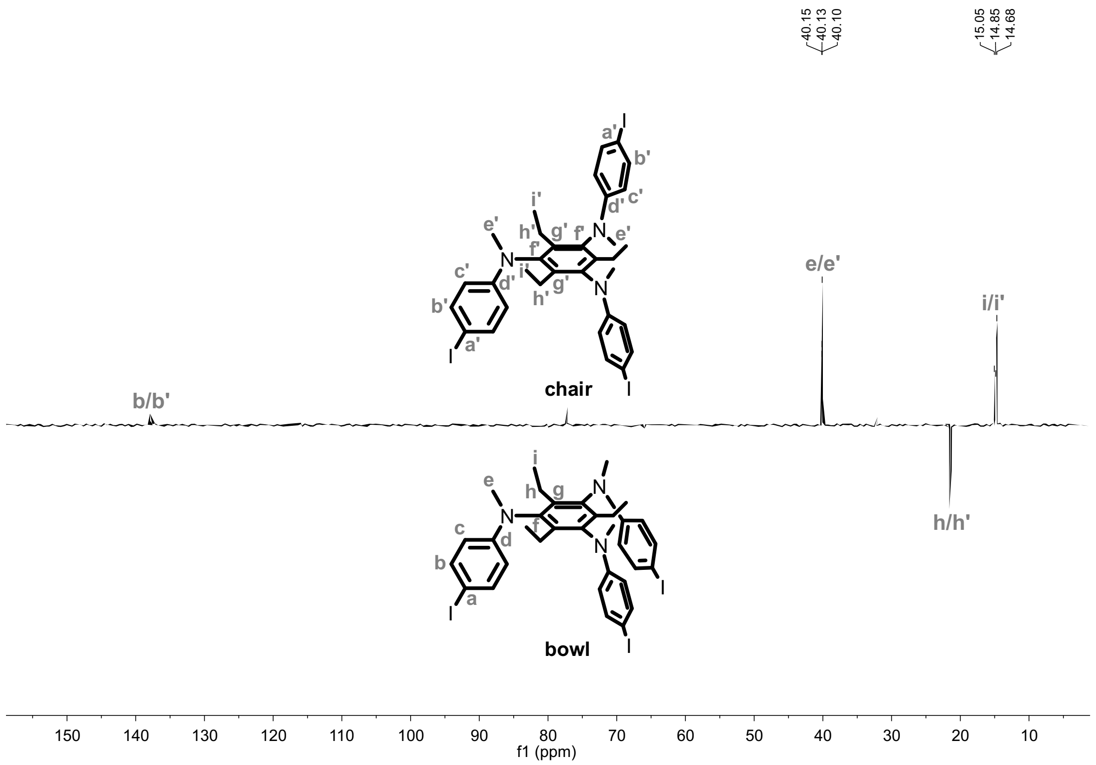

Figure S121: ^{13}C DEPT-135 NMR spectrum (151 MHz, $CDCl_3$, 298 K) of **11** (due the broadening of the $\mathbf{H_c}$ and $\mathbf{H_{c'}}$ signals, they were not visible in the spectrum).

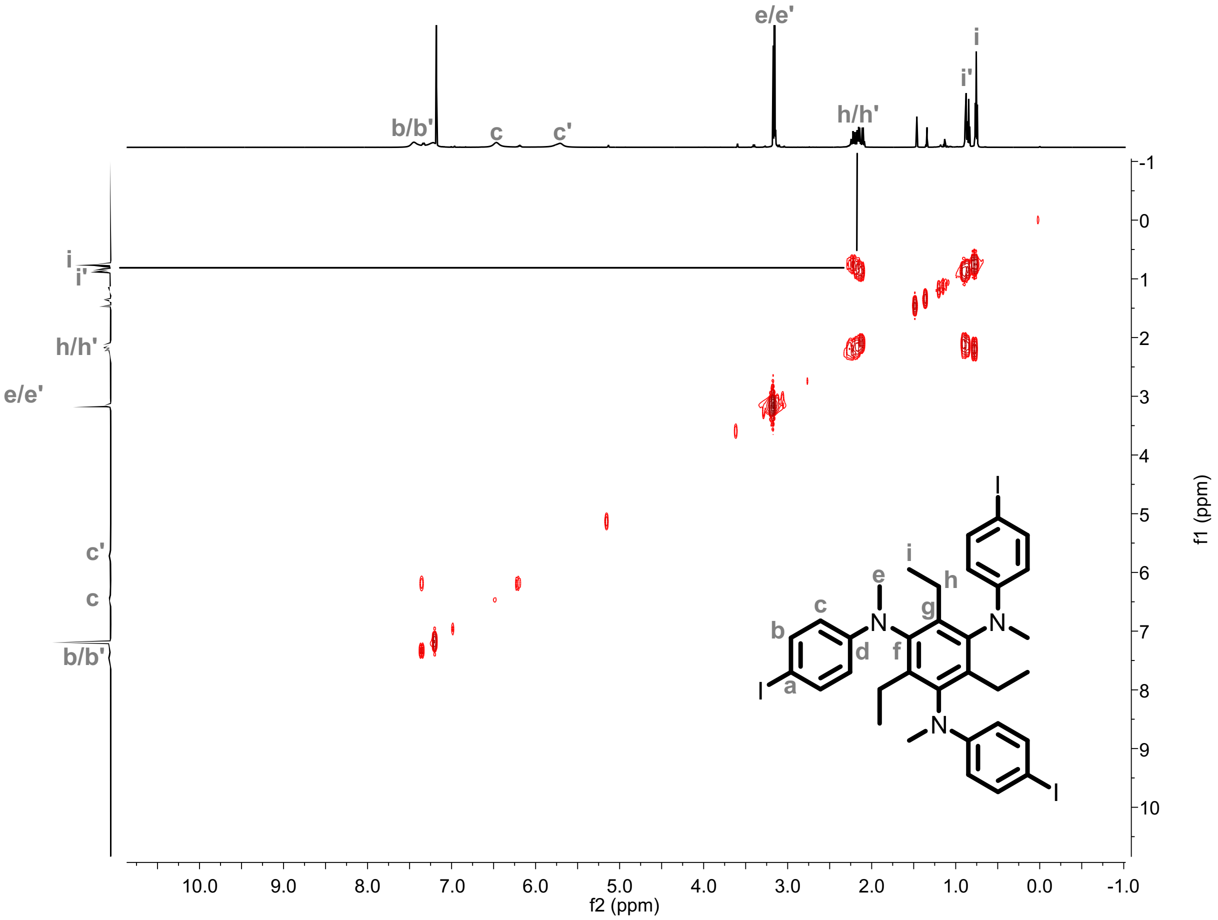

Figure S122: ^{1}H-^{1}H COSY NMR spectrum (600 MHz, $CDCl_3$, 298 K) of **11**.

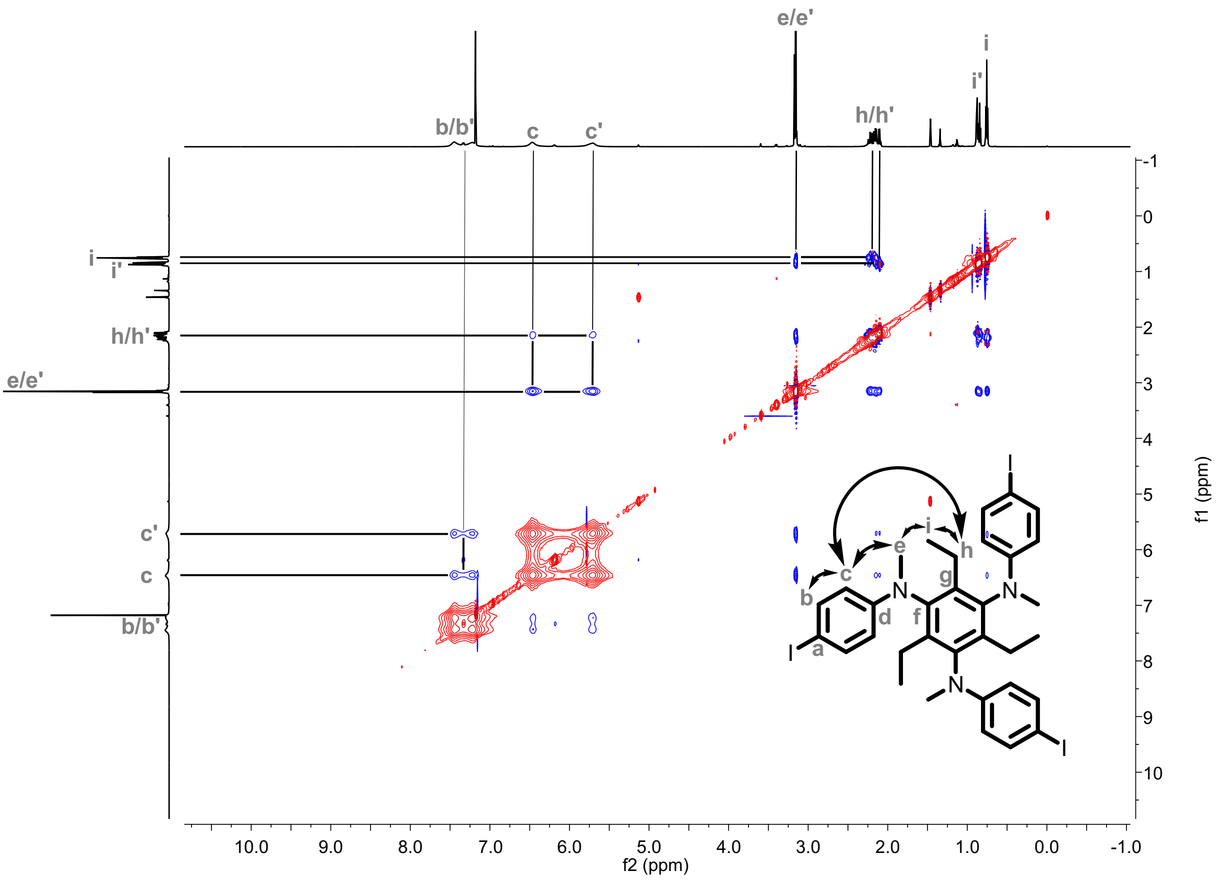

Figure S123: ^{1}H-^{1}H NOESY NMR spectrum (600 MHz, $CDCl_3$, 298 K) of **11**.

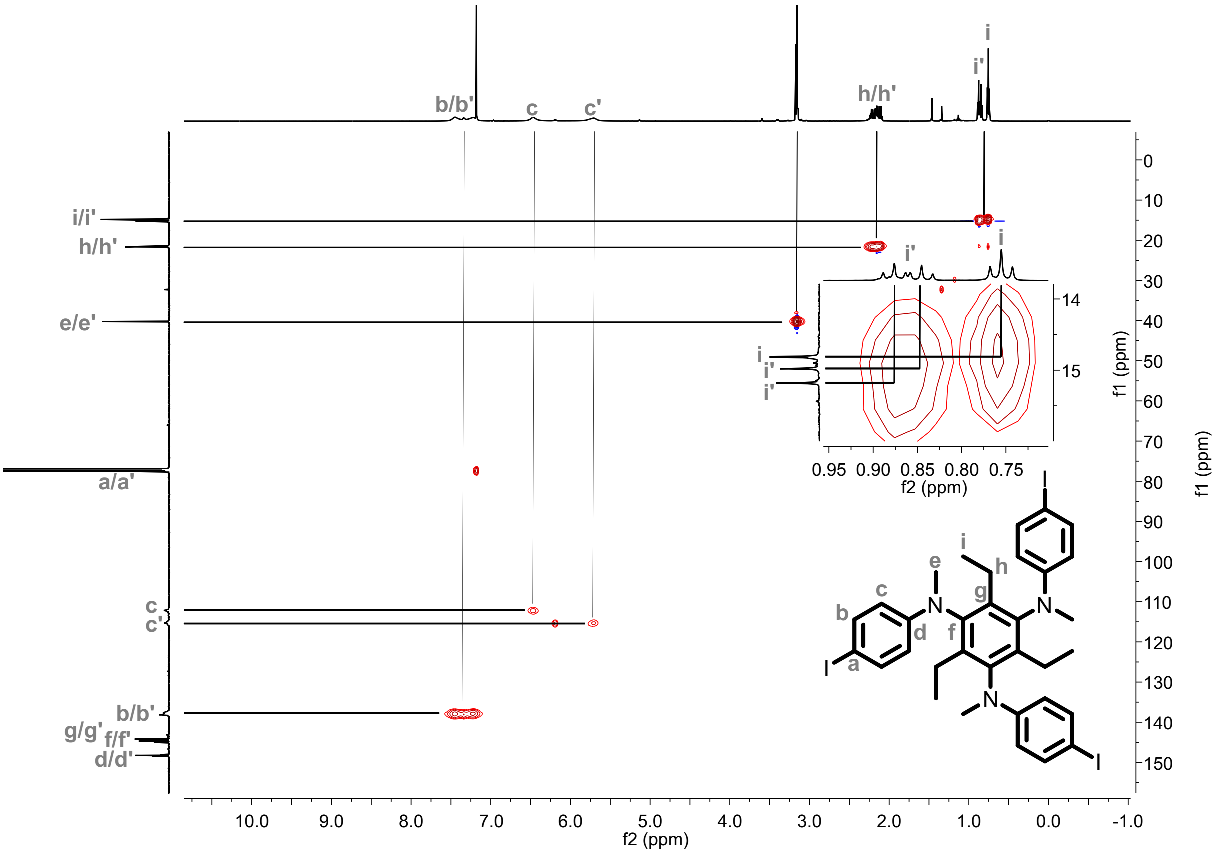

Figure S124: ^{1}H-^{13}C HSQC NMR spectrum (151 MHz, $CDCl_3$, 298 K) of **11**.

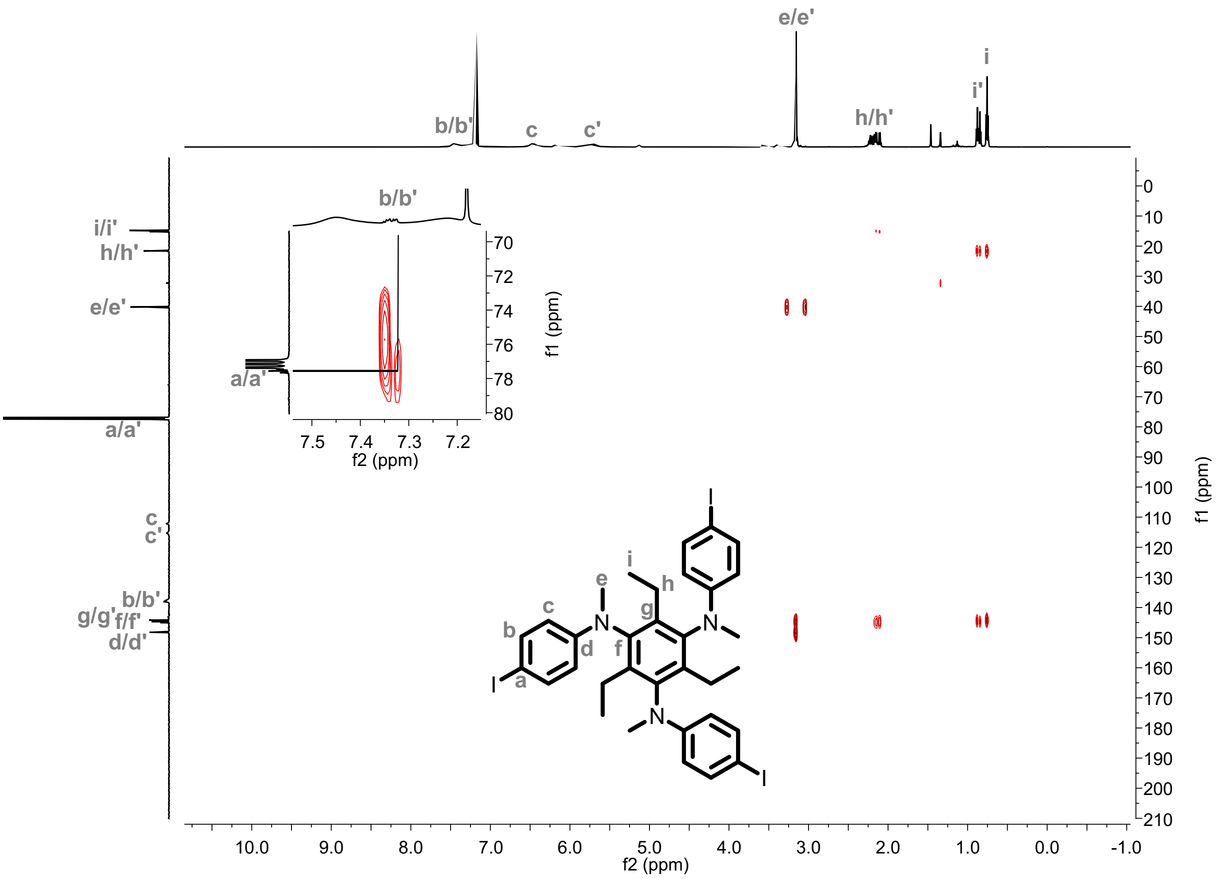

Figure S125: ^{1}H-^{13}C HMBC NMR spectrum (151 MHz, $CDCl_3$, 298 K) of **11**.

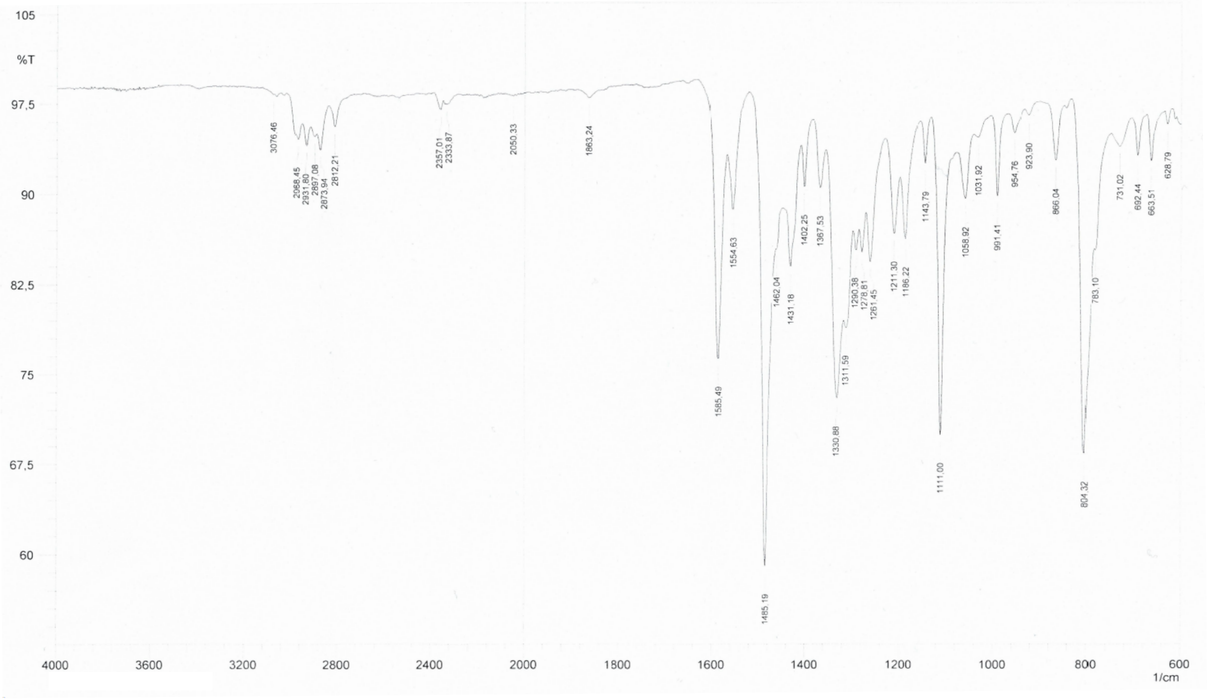

Figure S126: IR spectrum of **11**.

Acquisition Parameter

Source Type	ESI	Ion Polarity	Positive	Set Nebulizer	0.3 bar
Focus	Not active	Set Capillary	3500 V	Set Dry Heater	200 °C
Scan Begin	50 m/z	Set End Plate Offset	-500 V	Set Dry Gas	3.5 l/min
Scan End	1600 m/z	Set Collision Cell RF	500.0 Vpp	Set Divert Valve	Source

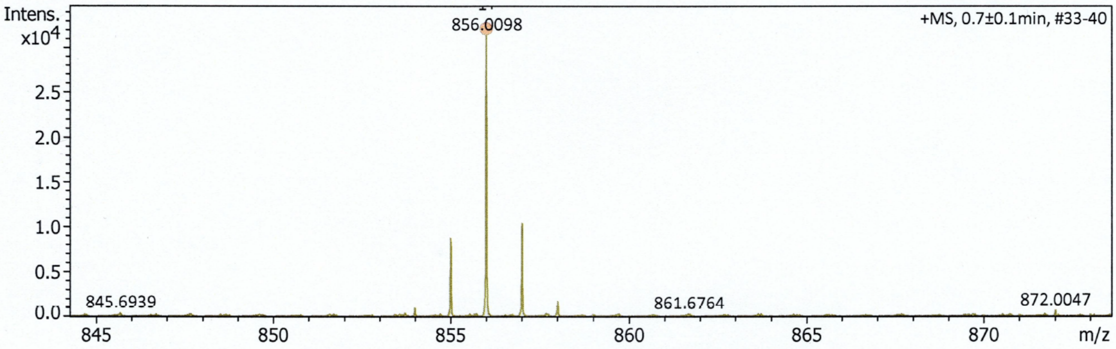

Meas. m/z	#	Ion Formula	m/z	err [ppm]	Mean err [ppm]	rdb (neutral)	N-Rule	e⁻ Conf	mSigma	Std I	Std Mean m/z	Std I VarNorm	Std m/z Diff	Std Comb Dev
856.0098	1	C33H37I3N3	856.0116	2.2	2.0	25.0	ok	even	20.8	32.3	n.a.	n.a.	n.a.	n.a.

Figure S127: HRMS (ESI) spectrum of **11**.

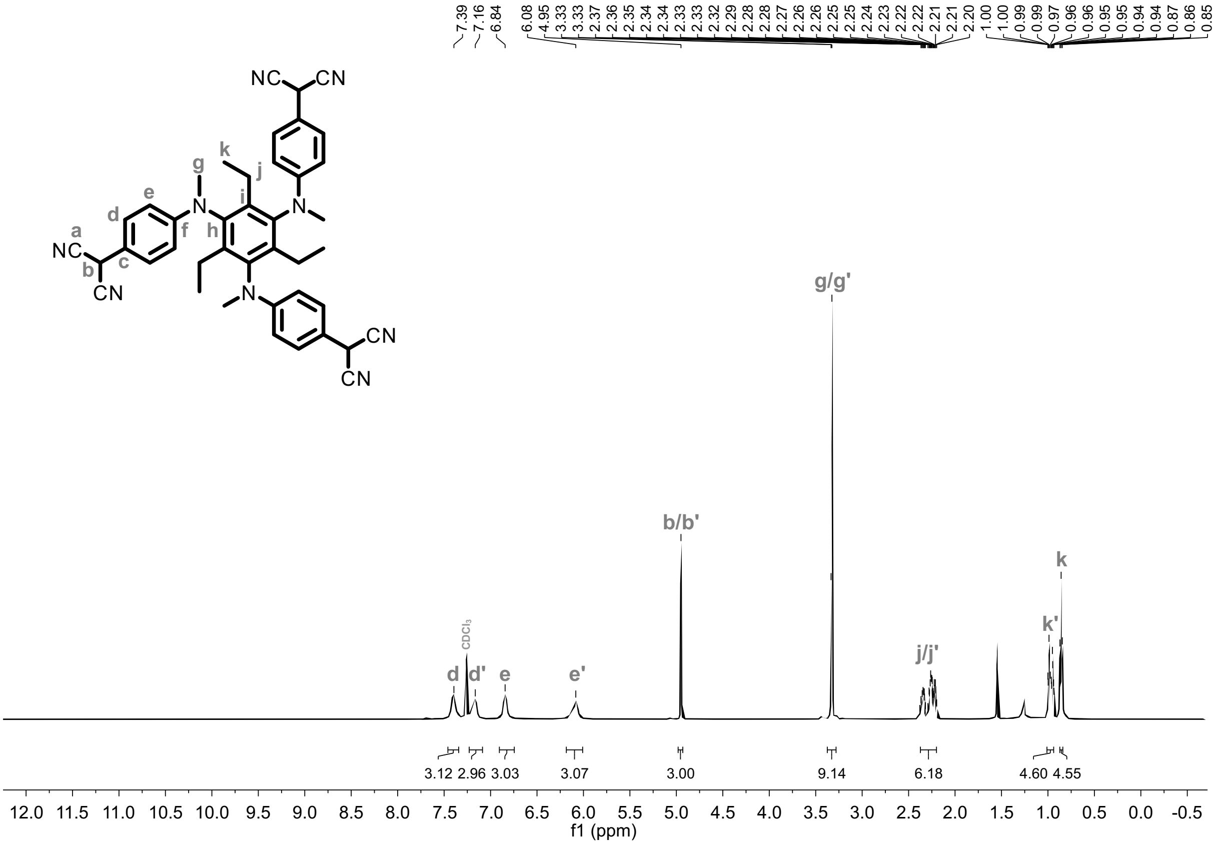

Figure S128: ^{1}H NMR spectrum (600 MHz, $CDCl_3$, 298 K) of **N**.

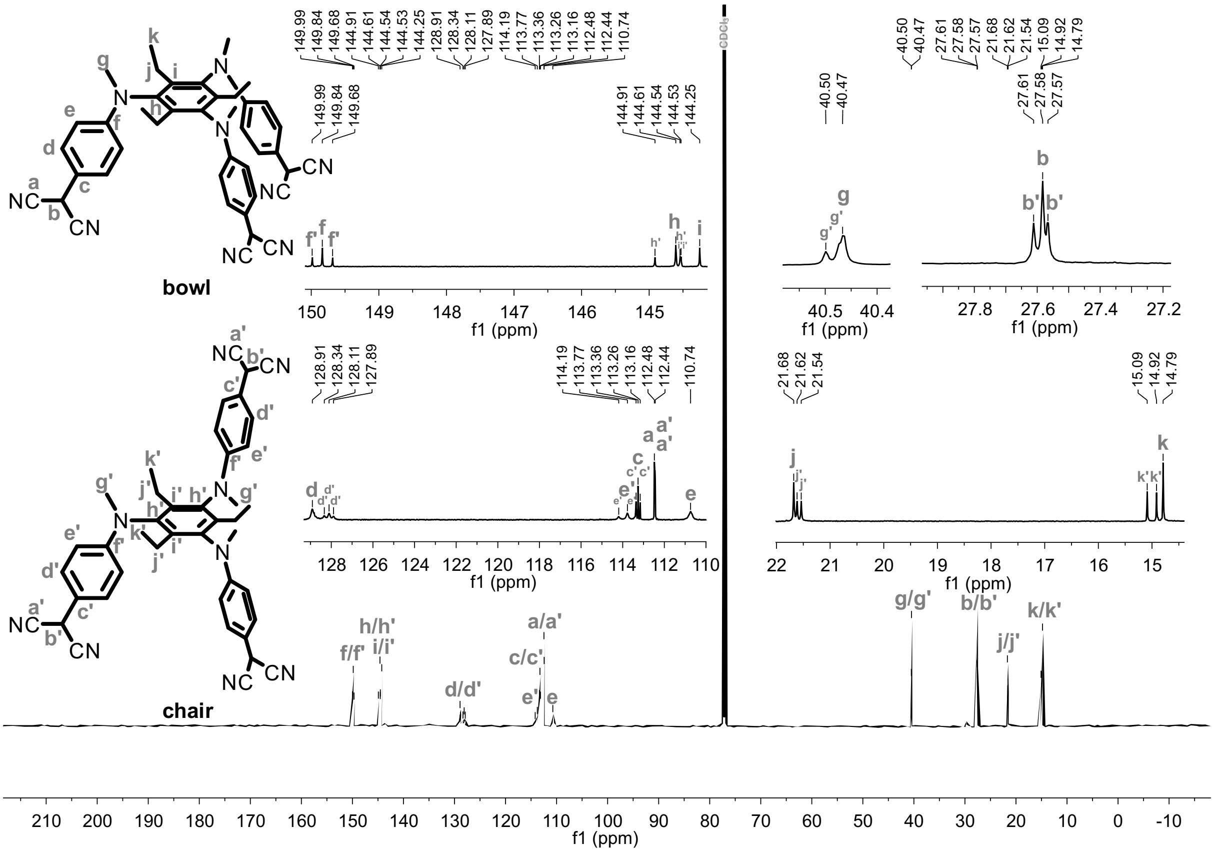

Figure S129: $^{13}C\{^{1}H\}$ NMR spectrum (151 MHz, $CDCl_3$, 298 K) of **N**.

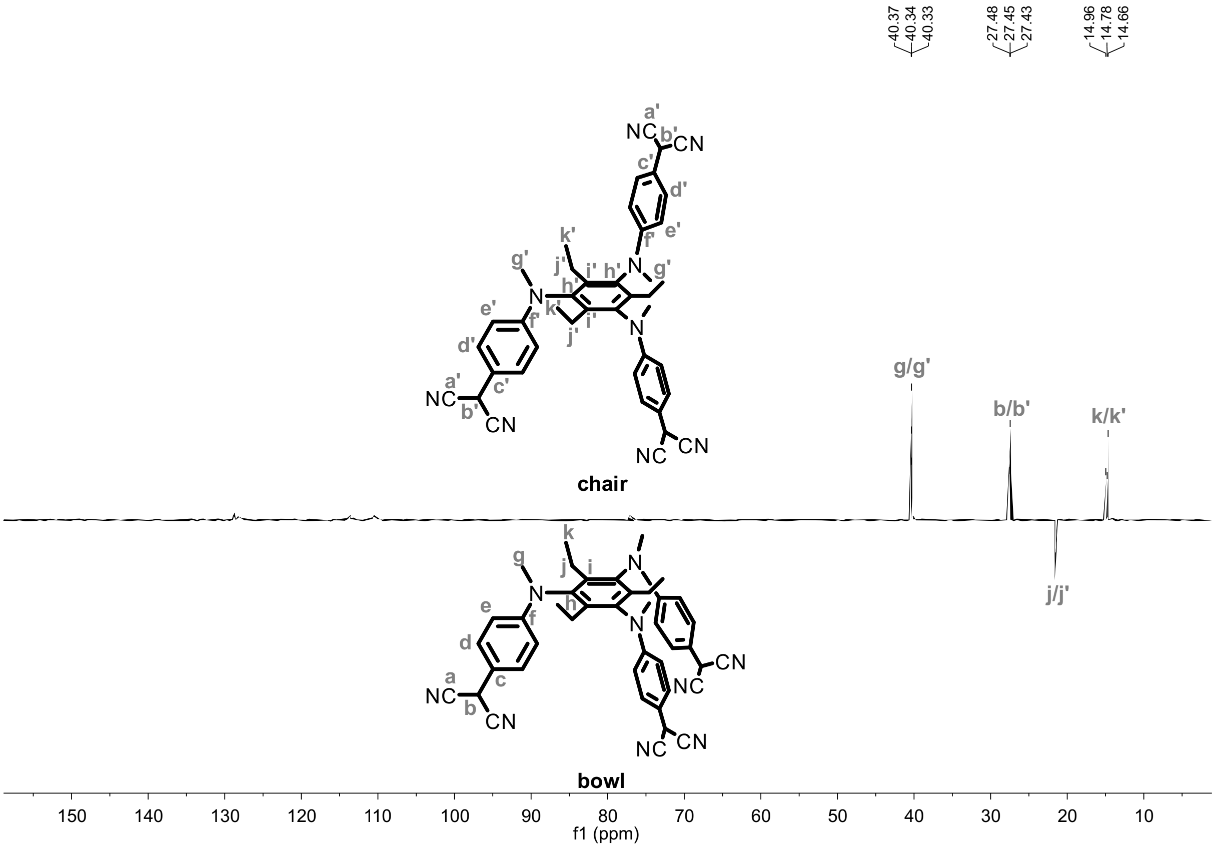

Figure S130: ^{13}C DEPT-135 NMR spectrum (151 MHz, $CDCl_3$, 298 K) of **N**.

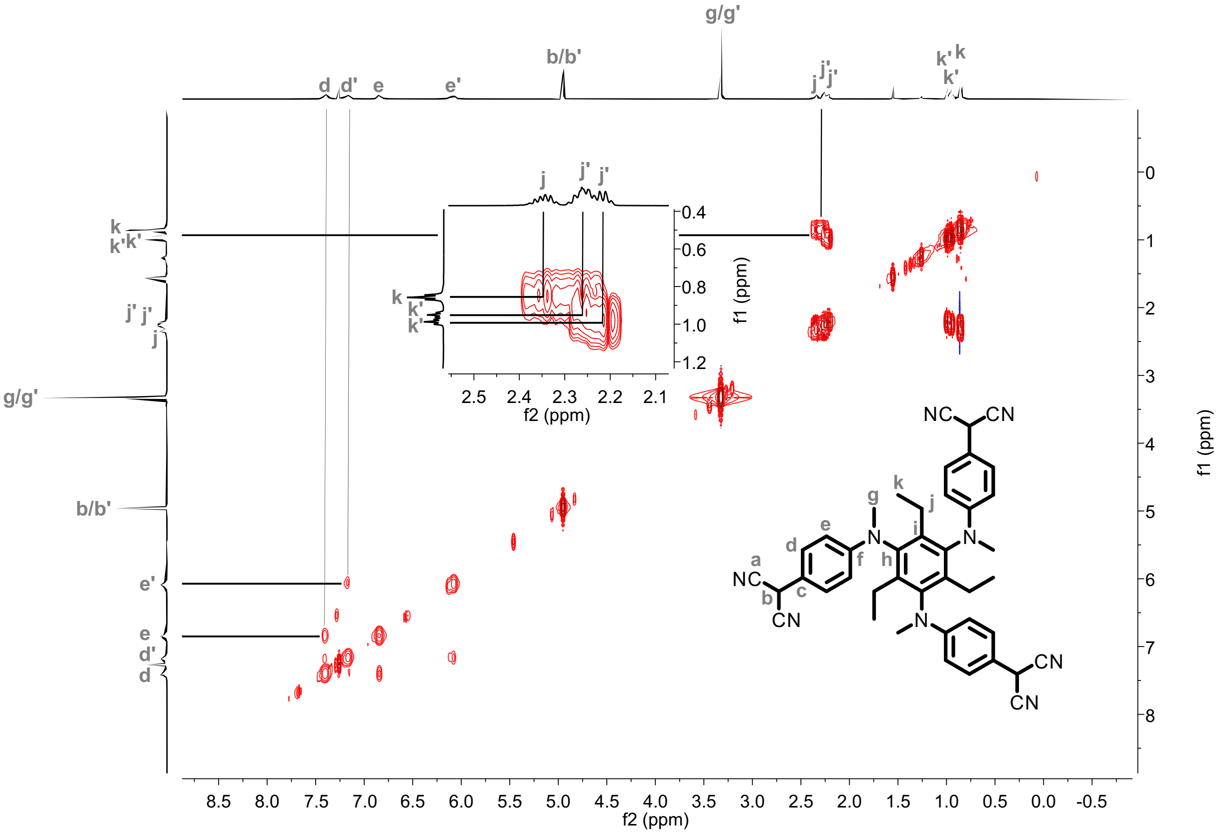

Figure S131: ^{1}H-^{1}H COSY NMR spectrum (600 MHz, $CDCl_3$, 298 K) of **N**.

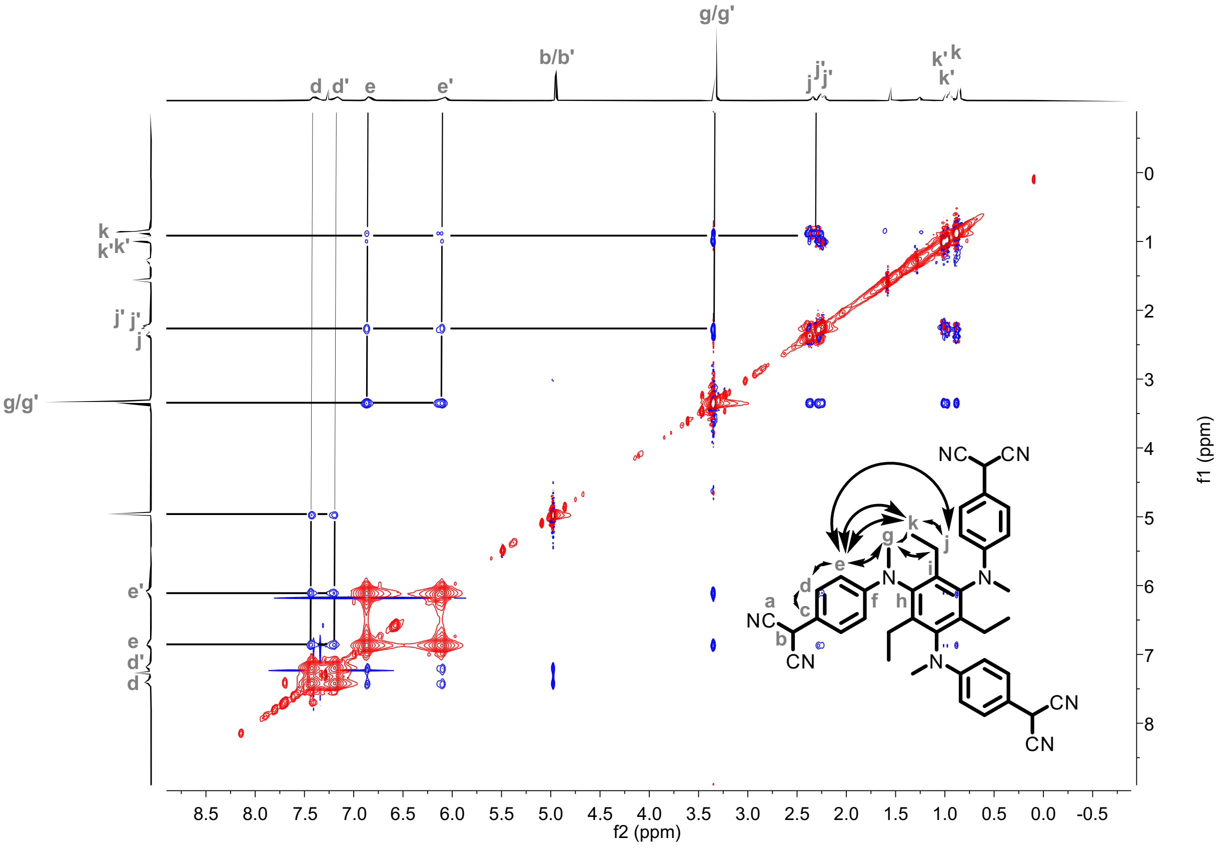

Figure S132: ^{1}H-^{1}H NOESY NMR spectrum (600 MHz, $CDCl_3$, 298 K) of **N**.

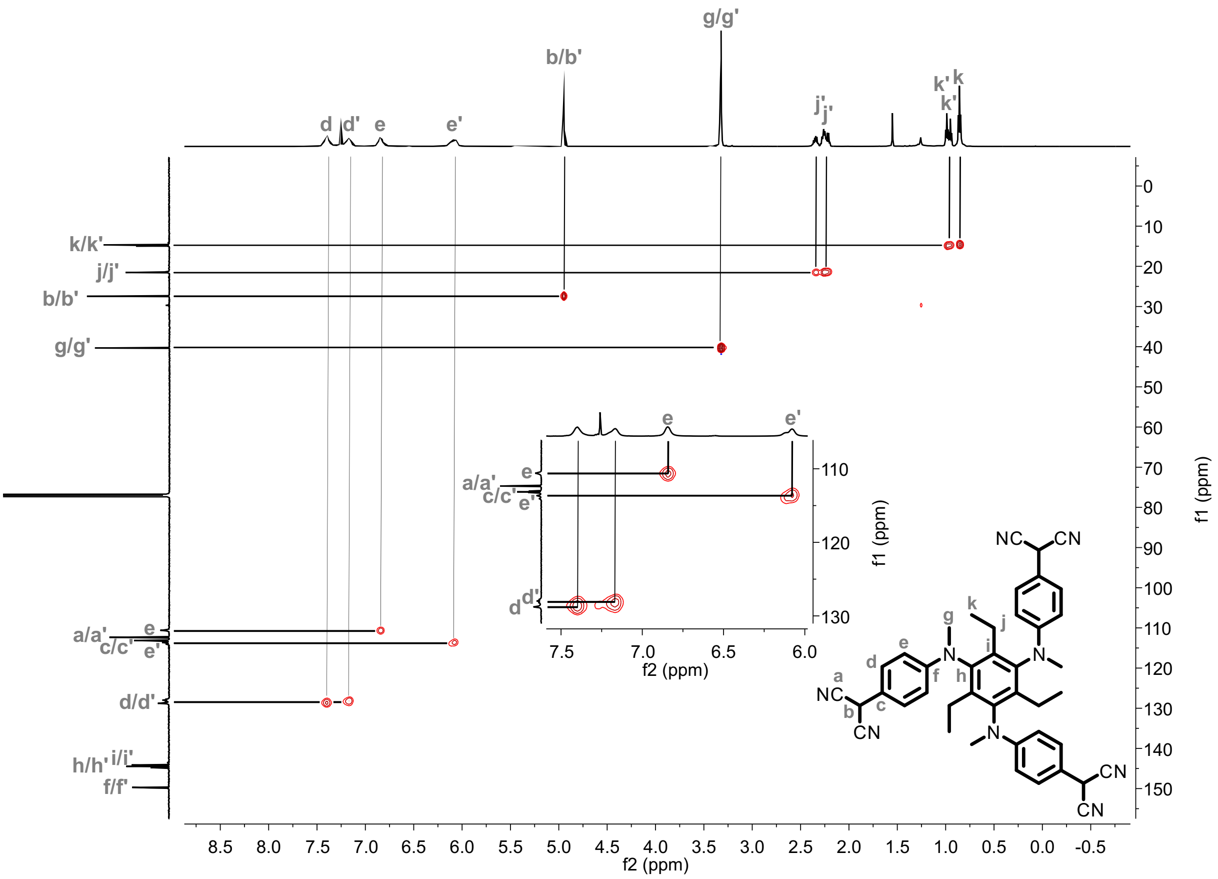

Figure S133: ^{1}H-^{13}C HSQC NMR spectrum (151 MHz, $CDCl_3$, 298 K) of **N**.

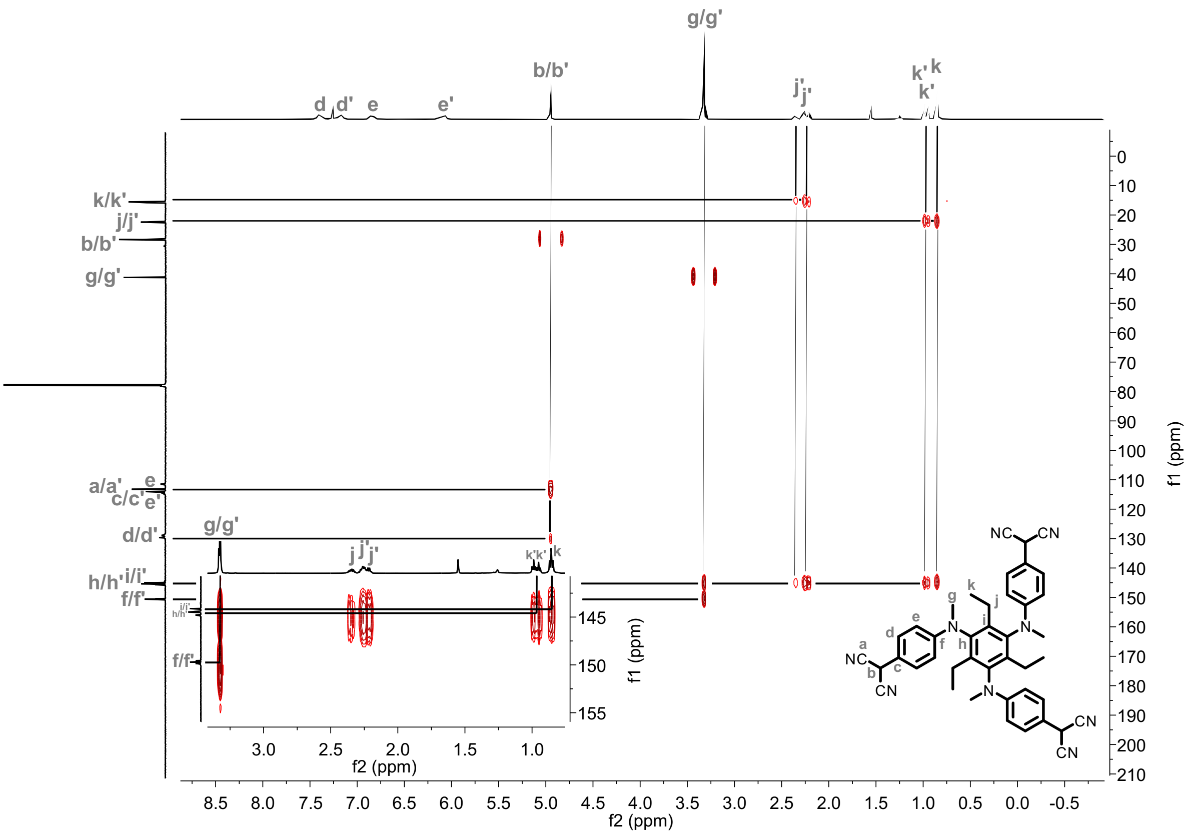

Figure S134: ^{1}H-^{13}C HMBC NMR spectrum (151 MHz, $CDCl_3$, 298 K) of **N**.

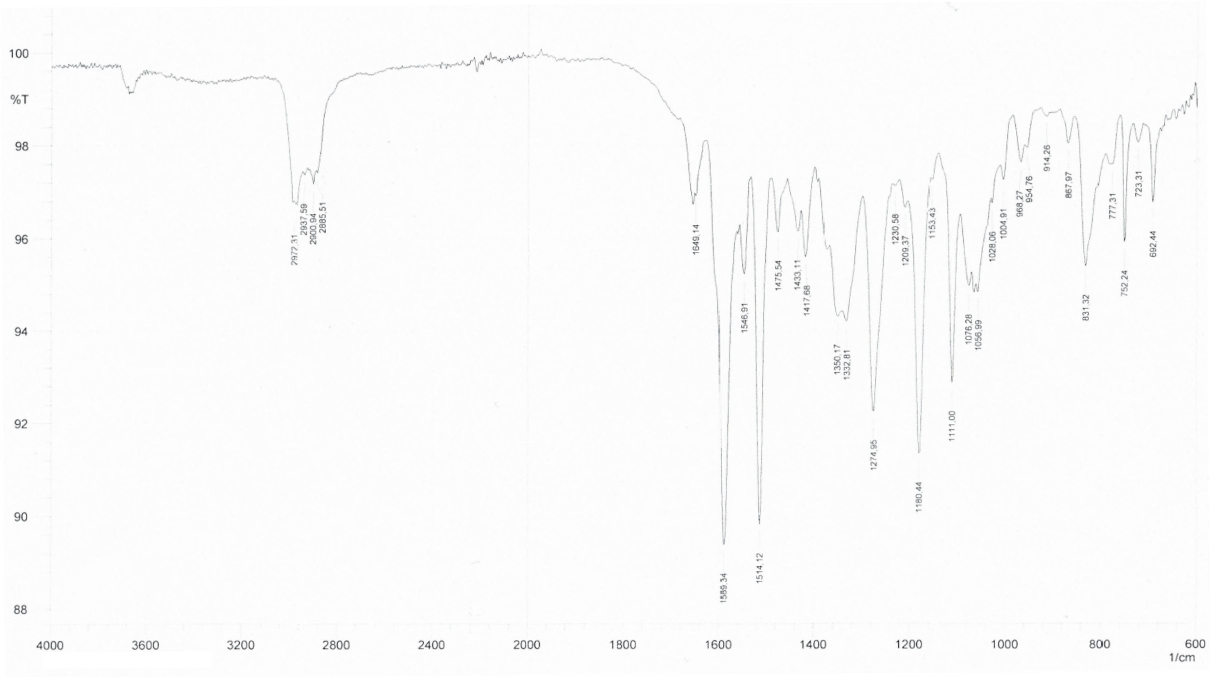

Figure S135: IR spectrum of **N**.

Acquisition Parameter

Source Type	ESI	Ion Polarity	Positive	Set Nebulizer	0.3 Bar
Focus	Not active	Set Capillary	4000 V	Set Dry Heater	180 °C
Scan Begin	50 m/z	Set End Plate Offset	-500 V	Set Dry Gas	4.0 l/min
Scan End	1500 m/z	Set Collision Cell RF	600.0 Vpp	Set Divert Valve	Source

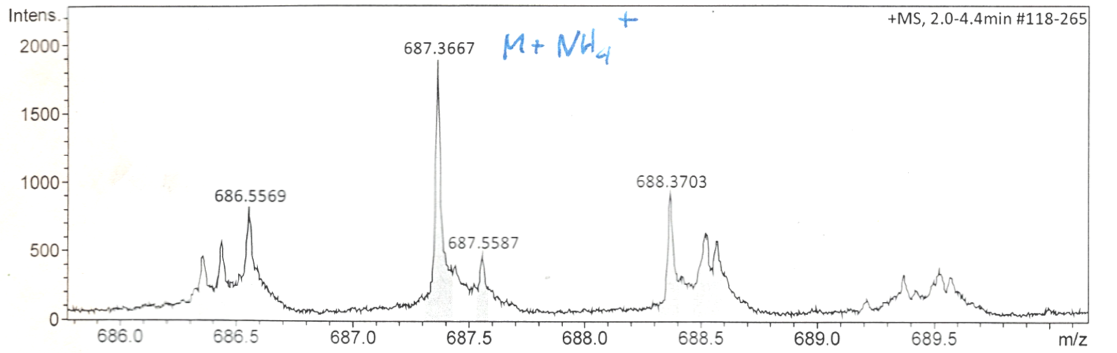

Meas. m/z	#	Ion Formula	m/z	err [ppm]	mSigma	# mSigma	Score	rdb	e⁻ Conf	N-Rule
687.3667	1	C41H47N6O4	687.3653	-2.0	60.2	1	51.03	21.5	even	ok
	2	C42H43N10	687.3667	-0.1	61.0	2	100.00	26.5	even	ok
	3	C45H51O6	687.3680	1.9	67.3	3	42.24	20.5	even	ok

Figure S136: HRMS (ESI) spectrum of **N**.

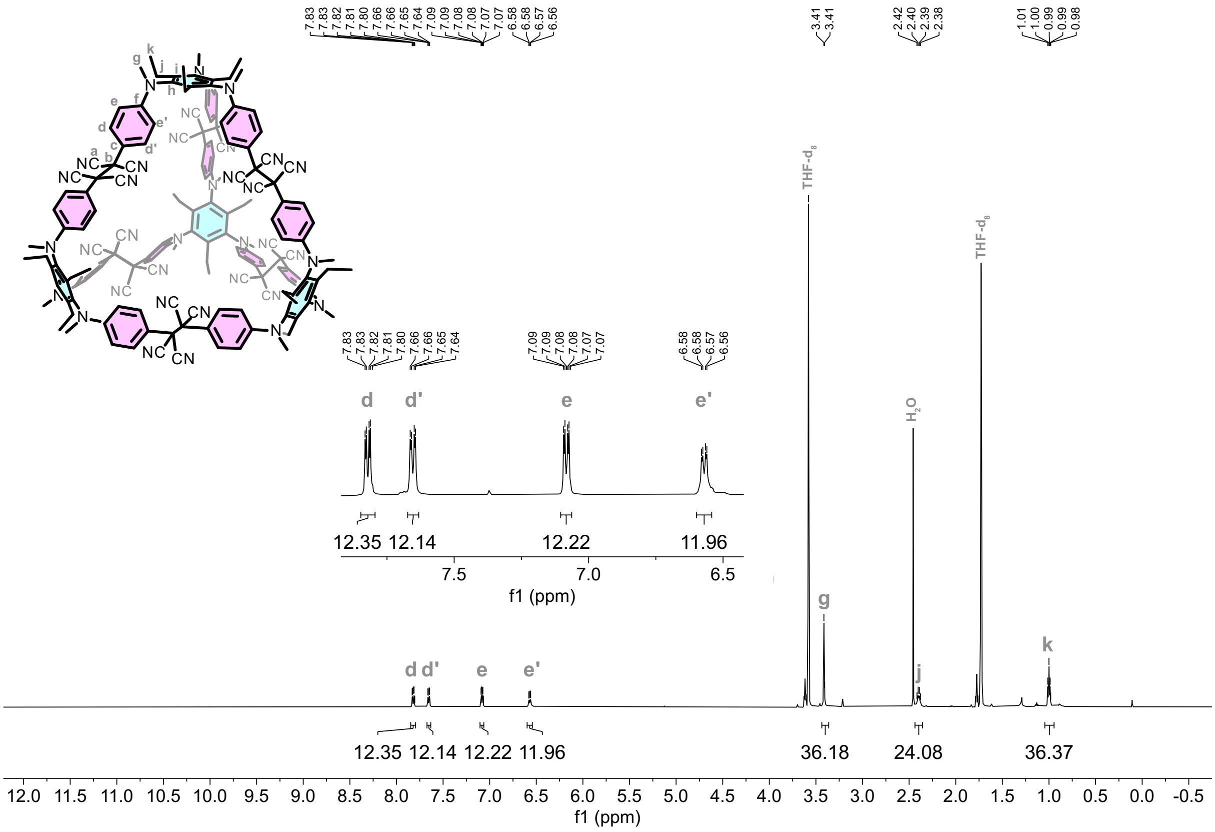

Figure S137: ^{1}H NMR spectrum (600 MHz, THF-d_8, 298 K) of **N[4]**.

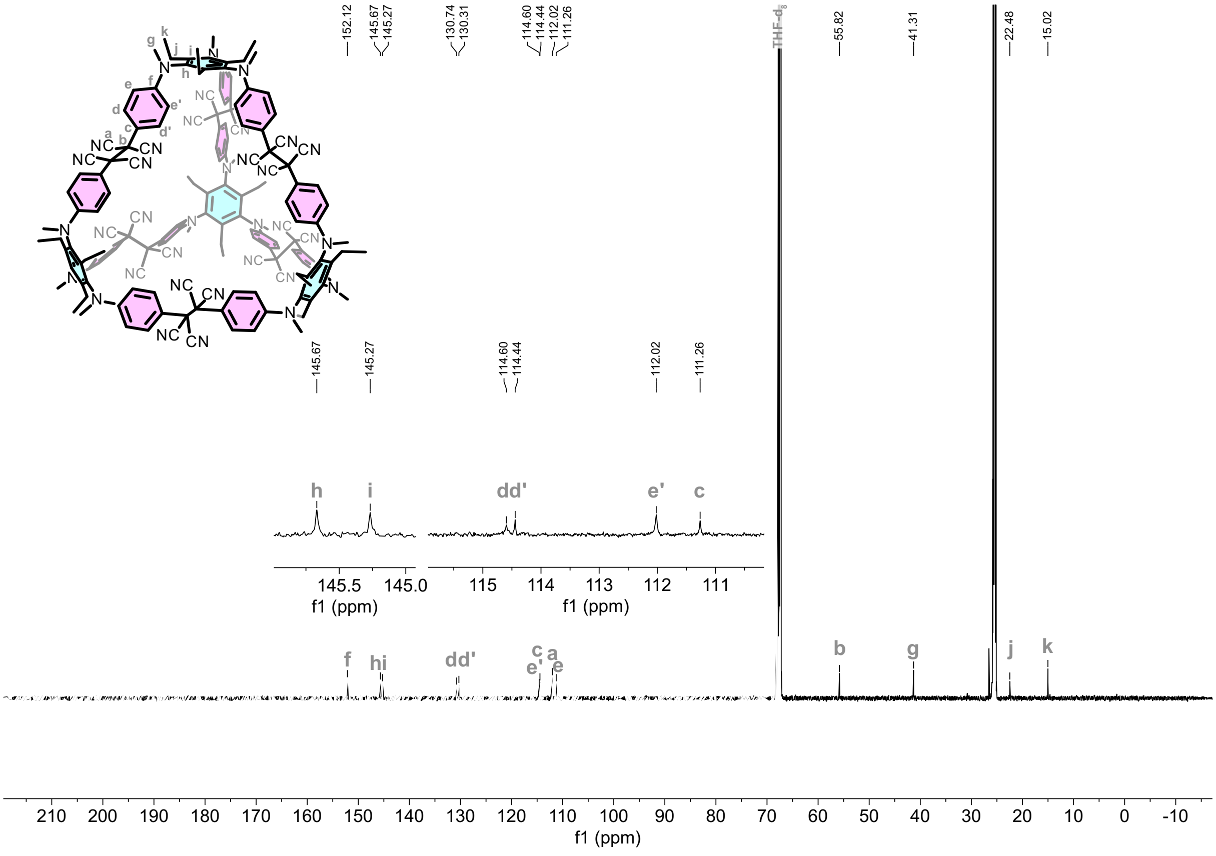

Figure S138: ^{13}C{^{1}H} NMR spectrum (151 MHz, THF-d_8, 298 K) of **N[4]**.

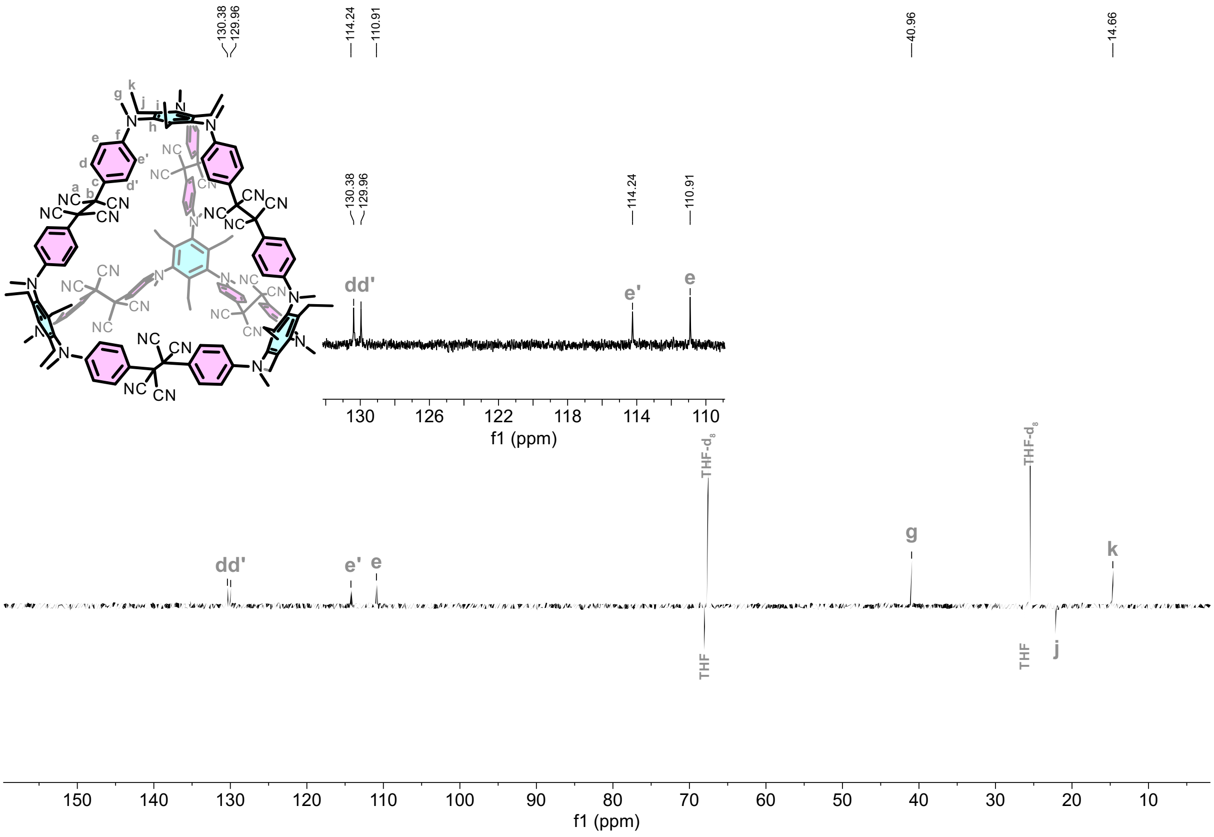

Figure S139: ^{13}C DEPT-135 NMR spectrum (151 MHz, THF-d$_8$, 298 K) of **N^4**.

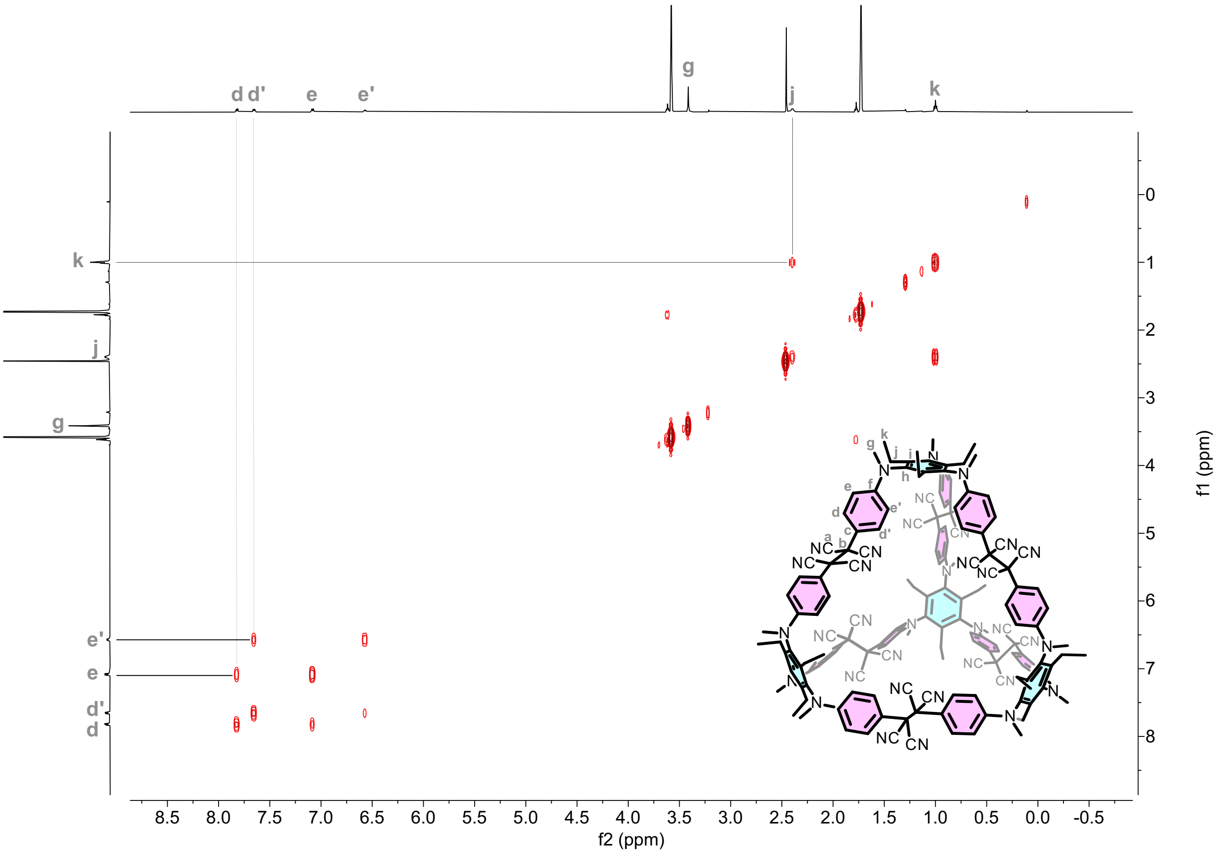

Figure S140: ^{1}H-^{1}H COSY NMR spectrum (600 MHz, THF-d$_8$, 298 K) of **N^4**.

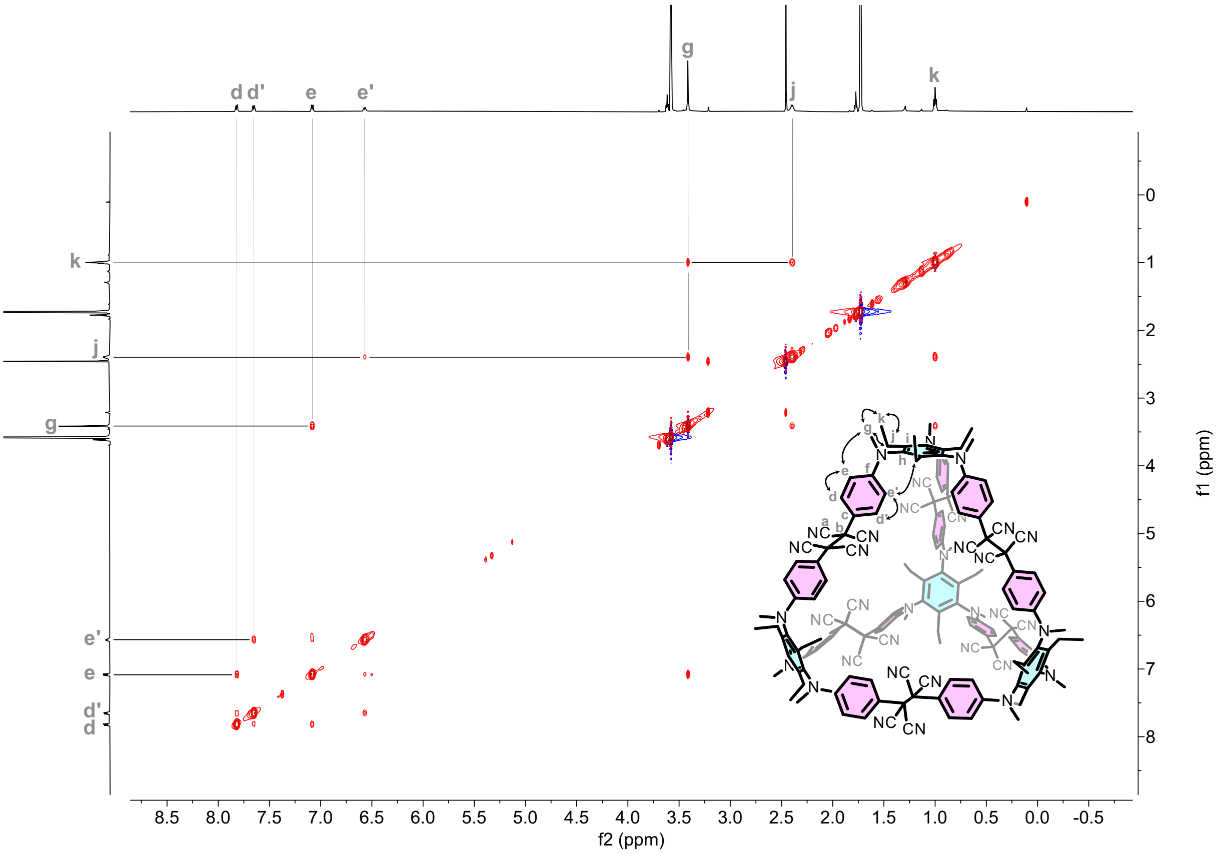

Figure S141: ^{1}H-^{1}H NOESY NMR spectrum (600 MHz, THF-d₈, 298 K) of $\mathbf{N^4}$.

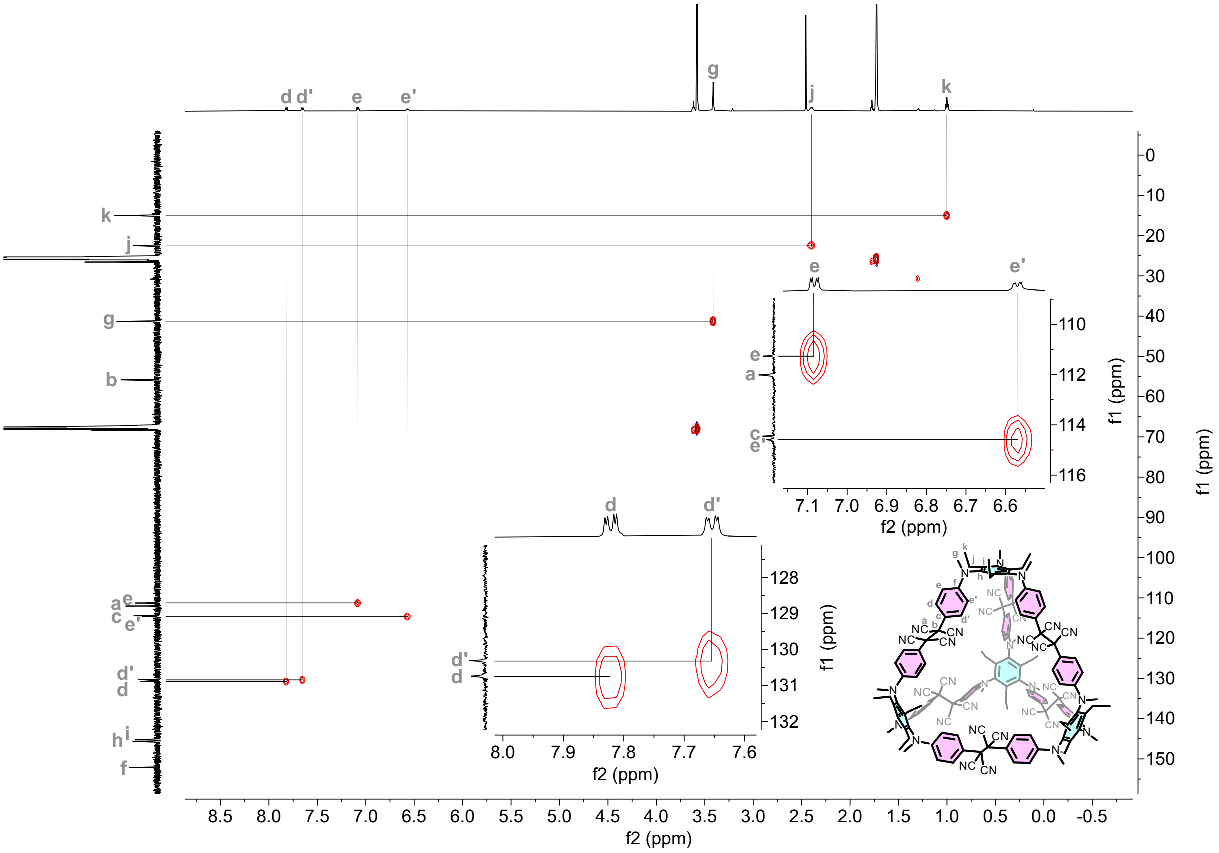

Figure S142: ^{1}H-^{13}C HSQC NMR spectrum (151 MHz, THF-d₈, 298 K) of $\mathbf{N^4}$.

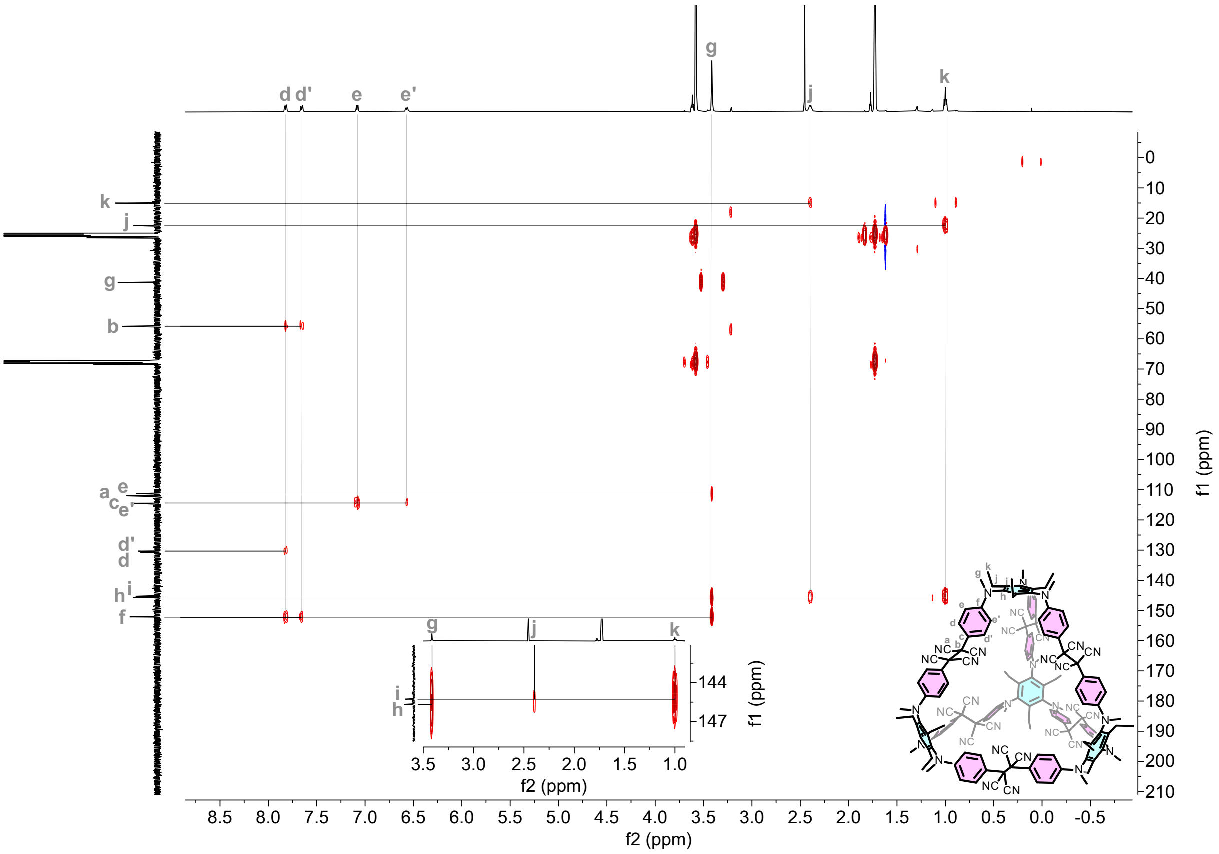

Figure S143: ^{1}H-^{13}C HMBC NMR spectrum (151 MHz, THF-d_8, 298 K) of $\mathbf{N^4}$.

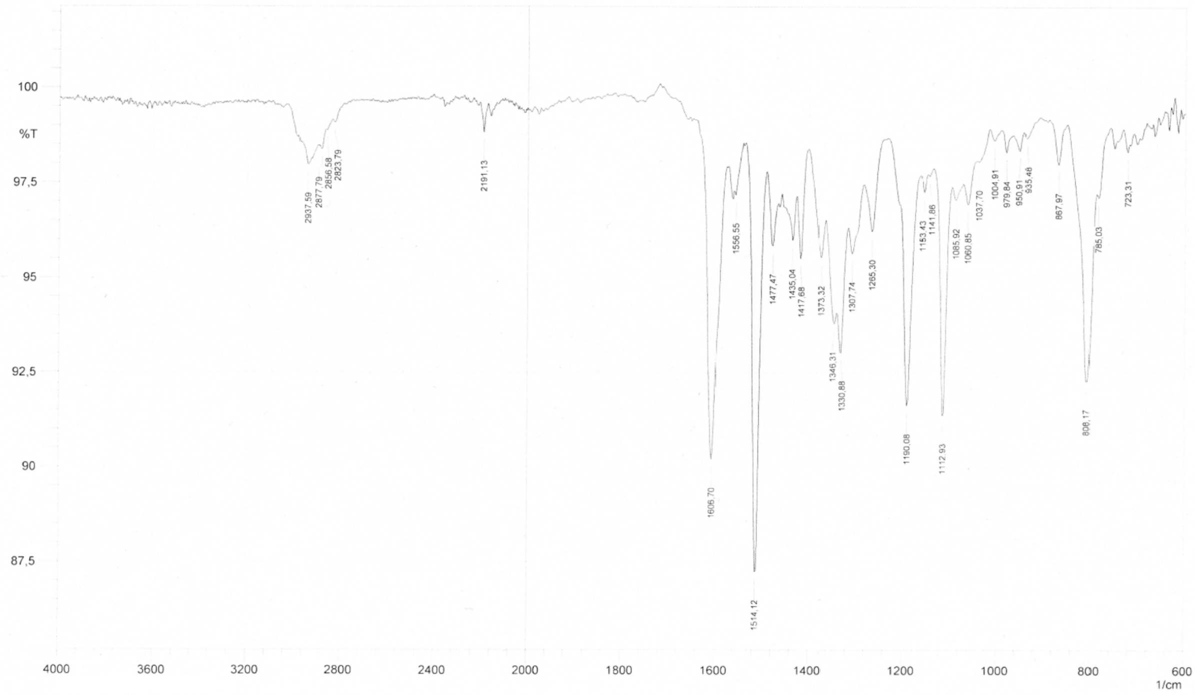

Figure S144: IR spectrum of $\mathbf{N^4}$.

13 References

[1] J. C. Lauer, W.-S. Zhang, F. Rominger, R. R. Schröder, M. Mastalerz, *Shape-Persistent [4+4] Imine Cages with a Truncated Tetrahedral Geometry*, *Chem. Eur. J.* **2018**, *24*, 1816-1820.

[2] S. Lee, A. Yang, T. P. Moneypenny, II, J. S. Moore, *Kinetically Trapped Tetrahedral Cages via Alkyne Metathesis*, *J. Am. Chem. Soc.* **2016**, *138*, 2182-2185.

[3] T. P. Moneypenny, II, A. Yang, N. P. Walter, T. J. Woods, D. L. Gray, Y. Zhang, J. S. Moore, *Product Distribution from Precursor Bite Angle Variation in Multitopic Alkyne Metathesis: Evidence for a Putative Kinetic Bottleneck*, *J. Am. Chem. Soc.* **2018**, *140*, 5825-5833.

[4] R. Evans, Z. Deng, A. K. Rogerson, A. S. McLachlan, J. J. Richards, M. Nilsson, G. A. Morris, *Quantitative Interpretation of Diffusion-Ordered NMR Spectra: Can We Rationalize Small Molecule Diffusion Coefficients?*, *Angew. Chem. Int. Ed.* **2013**, *52*, 3199-3202; *Angew. Chem.* **2013**, *125*, 3281-3284.

[5] D. J. Metz, A. Glines, *Density, viscosity, and dielectric constant of tetrahydrofuran between -78 and 30.degree*, *J. Phys. Chem.* **1967**, *71*, 1158-1158.

[6] O. V. Dolomanov, L. J. Bourhis, R. J. Gildea, J. A. K. Howard, H. Puschmann, *OLEX2: a complete structure solution, refinement and analysis program*, *J. Appl. Crystallogr.* **2009**, *42*, 339-341.

[7] G. Sheldrick, *SHELXT - Integrated space-group and crystal-structure determination*, *Acta Crystallogr. A* **2015**, *71*, 3-8.

[8] G. Sheldrick, *Crystal structure refinement with SHELXL*, *Acta Crystallogr. C* **2015**, *71*, 3-8.

[9] A. Spek, *PLATON SQUEEZE: a tool for the calculation of the disordered solvent contribution to the calculated structure factors*, *Acta Crystallogr. C* **2015**, *71*, 9-18.

[10] I. Badía-Domínguez, A. Pérez-Guardiola, J. C. Sancho-García, J. T. López Navarrete, V. Hernández Jolín, H. Li, D. Sakamaki, S. Seki, M. C. Ruiz Delgado, *Formation of Cyclophane Macrocycles in Carbazole-Based Biradicaloids: Impact of the Dicyanomethylene Substitution Position*, *ACS Omega* **2019**, *4*, 4761-4769.
